# Oxidative Addition of Aryl Electrophiles into a Red-Light-Active Bismuthinidene

**DOI:** 10.1021/jacs.3c06651

**Published:** 2023-08-21

**Authors:** Mauro Mato, Paolo Cleto Bruzzese, Fumiya Takahashi, Markus Leutzsch, Edward J. Reijerse, Alexander Schnegg, Josep Cornella

**Affiliations:** † Max-Planck-Institut für Kohlenforschung, Kaiser-Wilhelm-Platz 1, Mülheim an der Ruhr 45470, Germany; ‡ Max Planck Institute for Chemical Energy Conversion, Stiftstrasse 34−36, Mülheim an der Ruhr 45470, Germany

## Abstract

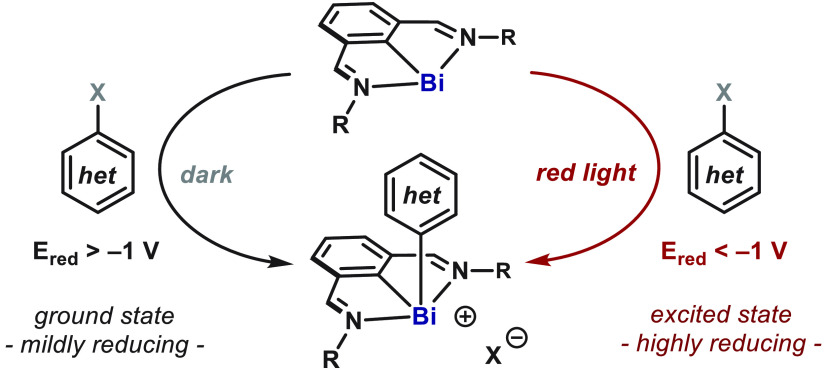

The oxidative addition of aryl electrophiles is a fundamental organometallic reaction widely applied in the field of transition metal chemistry and catalysis. However, the analogous version based on main group elements still remains largely underexplored. Here, we report the ability of a well-defined organobismuth(I) complex to undergo formal oxidative addition with a wide range of aryl electrophiles. The process is facilitated by the reactivity of both the ground and excited states of *N*,*C*,*N*-bismuthinidenes upon absorption of low-energy red light.

The oxidative addition of organic electrophiles into transition metal complexes is one of the most important processes in organometallic chemistry (Figure 1A),^1^ as it commonly represents the initial step in metal-catalyzed cross-coupling reactions.^2^ Recent years have witnessed the rapid development of transition-metal-like reactivity by main group compounds (MG), which have complemented and expanded the canonical behavior of d-block elements.^3−5^ Despite the advances in this area, several challenges still remain when mimicking fundamental organometallic steps toward the activation of strong bonds for organic synthesis. For example, while the S_N_2-type oxidative addition of alkyl (pseudo)halides is a well-established process for MG elements,^5,6^ the oxidative addition of aryl electrophiles into well-defined MG complexes has not been generalized, and the reported examples are limited to highly electron-poor (hetero)aryl fluorides (Figure 1B).^5,7^ This limitation stems from the lack of d-orbital reactivity, which is traditionally responsible in transition metals for the precoordination to the π-system, followed by a 3-centered transition state (TS) to cleave the C(sp^2^)–X bond.^1^ Highly reducing elemental metals [e.g., Mg(0)] have long been known to mimic such reactivity through an alternative single-electron transfer followed by fragmentation and radical recombination (Figure 1A, right).^8^ However, aside from particular exceptions involving activated elemental p-block elements [e.g., In(0), Bi(0), etc.],^9^ formal oxidative additions of well-defined MG complexes into aryl (pseudo)halides are largely underdeveloped.

**Figure 1:**
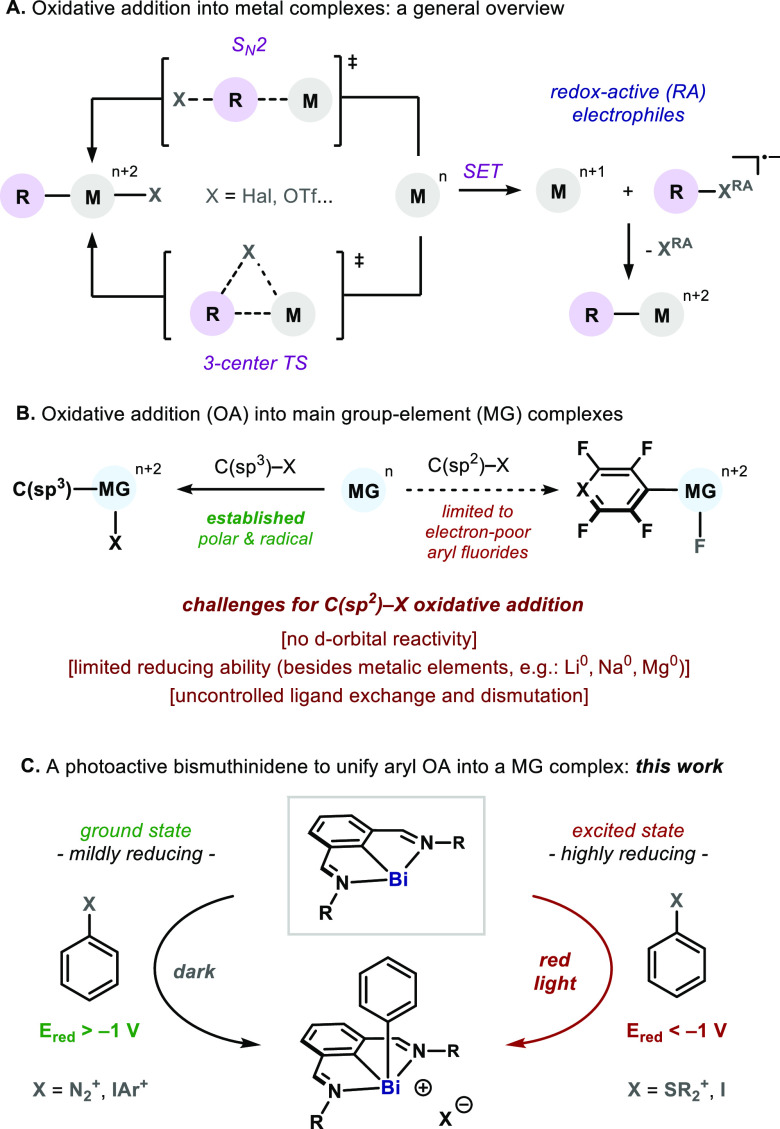
(A) General types of oxidative additions into metal complexes. (B) Background and limitations of C–X oxidative additions into main group elements. (C) Photoactive bismuthinidenes unlock aryl oxidative addition into a single MG complex.

The ability of bismuth complexes to maneuver through different oxidation states has recently been established as a feasible platform for the development of new redox processes and catalytic methodologies.^10^ Of particular interest is the ability of *N*,*C*,*N*-bismuthinidenes^6b,11^ to undergo oxidative addition with redox-active *alkyl* electrophiles via SET.^12^ The radical nature of these processes led us to consider the reactivity of Bi(I) complexes with redox-active *aryl* electrophiles, such as aryl diazonium or iodonium salts (Figure 1C, left). However, bismuthinidenes such as **1** are only mildly reducing in the ground state (ca. *E*_1/2_ = −0.5 V vs SCE), thus preventing the reduction of more challenging electrophiles, for example, aryl halides. Yet, accessing excited states of intensely colored *N*,*C*,*N*-bismuthinidenes—which absorb visible light down to the red region—results in a highly reducing complex able to activate a broader variety of electrophiles, thereby leading to a wide range of aryl-bismuth(III) complexes (Figure 1C, right).^13,7d,10,12^ This work introduces *N*,*C*,*N*-bismuthinidenes as unique photoactive species, which sets an entry point to new photoinduced events^14^ and exploits the low-energy bands (red) of visible light.^15^

Initially, we evaluated the reactivity of *N*,*C*,*N*-bismuthinidenes with aryl diazonium salts **2** (Scheme 1).^16^ As a result of the mildly reducing nature of bismuthinidenes in the ground state [*E*_1/2_ (**1**) = −0.47 V vs SCE], they undergo an energetically favorable SET to **2** followed by a fast fragmentation, which leads to stable aryl bismuthonium complexes **3** after N_2_ extrusion. The intermediacy of aryl-radical fragments is supported by the observation of small amounts of product **4**, which would be the result of the reaction between an aryl radical with the *d*_*3*_-MeCN solvent via D-atom abstraction.^17^*N*,*C*,*N*-Bismuthinidenes supported by either imines or oxazolines were engaged successfully (**3a**–**c**). The method was suitable for the introduction of electronically distinct aryl rings (**3d**–**e**) and also heteroaryl units (**3g**–**i**), thus highlighting the broad functional group tolerance of the process. Not surprisingly, the presence of a Br in the aryl unit could be tolerated, thereby indicating orthogonal reactivity to common electrophilic sites of oxidative addition (**3f**). Electron-rich aryl bismuthonium cations, such as **3d**, were found to be surprisingly stable, even in solution under air or light irradiation. In addition to diazonium salts, we also investigated the reactivity of **1** with diaryliodonium salt **6**; smooth conversion to 4-methoxyphenyl bismuthonium **7a** was observed with the concomitant formation of 4-iodoanisole. Similarly to **2a**, **6** reacts immediately at room temperature in the absence of light. This is consistent with the redox behavior of **2a** and **6**, which display a cathodic peak with an onset potential at −0.3 V and −0.4 V vs SCE, respectively (Figure 2A, right).^17^

**Scheme 1:**
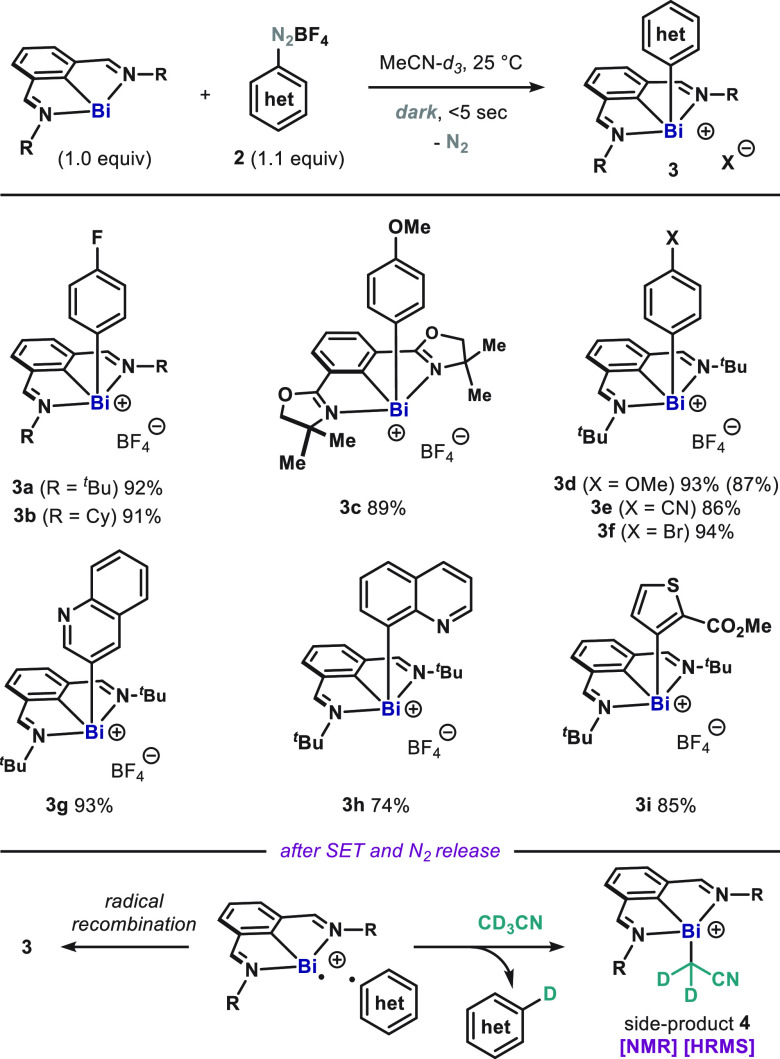
Scope of the Oxidative Addition of Aryl Diazonium Salts into Ground-State Bismuth(I) Yields determined by ^1^H NMR using 1,3,5-trimethoxybenzene as internal standard. Isolated yields are in parentheses.

**Figure 2:**
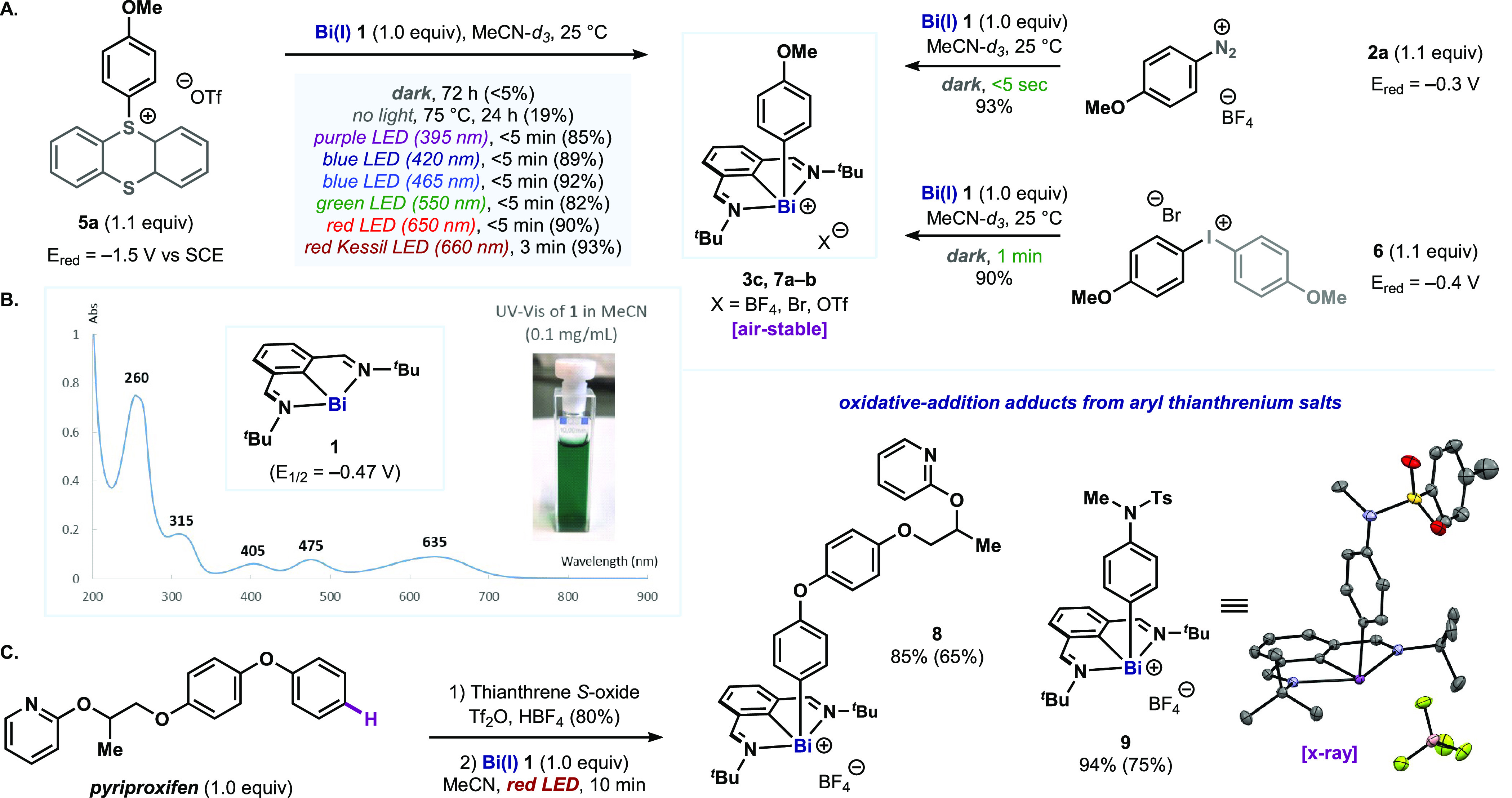
(A) Oxidative addition of aryl electrophiles to **1** in the dark and under light irradiation. (B) Absorption spectrum and physical appearance of **1**. (C) Formal C–H bismuthation of arenes through sequential regioselective thianthrenation and red-light-promoted oxidative addition. Yields determined by ^1^H NMR using 1,3,5-trimethoxybenzene as internal standard. Isolated yields are in parentheses.

Recently, aryl thianthrenium salts have been established as versatile arylelectrophile reagents.^18^ They are easily accessible via regioselective C–H thianthrenation and exhibit very fast fragmentation rates upon one-electron reduction.^19^ However, they display more negative reduction potentials than aryl diazonium salts, which should be out of reach of the mildly reducing capabilities of ground-state **1**. Indeed, the reaction of **1** and arylthianthrenium **5a** (*E*_red_ = −1.5 V vs SCE) did not afford any significant conversion after 3 days in the dark and only sluggish reactivity under thermal conditions (Figure 2A). This lack of reactivity, together with the intense dark green color displayed by *N*,*C*,*N*-bismuthinidenes, prompted us to study the photochemical behavior of **1**. Absorption spectroscopy revealed that **1** is able to garner light throughout the visible range and tailing down to the NIR region (Figure 2B). It is worth highlighting that strong light absorption, a reversible redox behavior, and stability toward photochemical decomposition provide **1** with some of the key features of most photoredox catalysts.^20^ Encouraged by this analogy, we hypothesized that the reducing capabilities of **1** could be significantly improved in the excited state. Upon blue light irradiation, the reaction of **1** with **5a** resulted in complete conversion to **7b** in less than 5 min (as judged by the drastic color change of the reaction mixture, from dark green to pale yellow). Interestingly, when the light source was changed, oxidative addition adduct **7b** was obtained in excellent yields regardless of the wavelength: from 395 to 660 nm (Figure 2A, left). Most interestingly, we probed the possibility of driving a formal oxidative addition into a main group element complex by using low-energy red light (660 nm, 1.88 eV, or 43 kcal/mol). The versatility of this reactivity is illustrated with the synthesis of complex **8** (Figure 2C): regioselective C–H thianthrenation of pyriproxifen, followed by red-light-promoted oxidative addition to **1**, afforded the product of formal oxidative C–H bismuthation in high yield. Similarly, **9** was obtained as a pure white crystalline solid after simple filtration. The connectivity and structure of the products could be confirmed by single-crystal X-ray diffraction analysis of **9**.^17^ In situ EPR was chosen to investigate the putative intermediacy of aryl-radical fragments. The reaction of **5a** with **1** under light irradiation in the presence of DMPO (5,5-dimethyl-1-pyrroline *N*-oxide). as spin-trapping agent resulted in the identification of paramagnetic adduct **14**.^21^ Furthermore, the reaction of aryl diazonium salt **2b** with **1** in the presence of B_2_pin_2_ afforded borylated compound **13**, which is consistent with the formation of an aryl radical (Scheme 2).

**Scheme 2:**
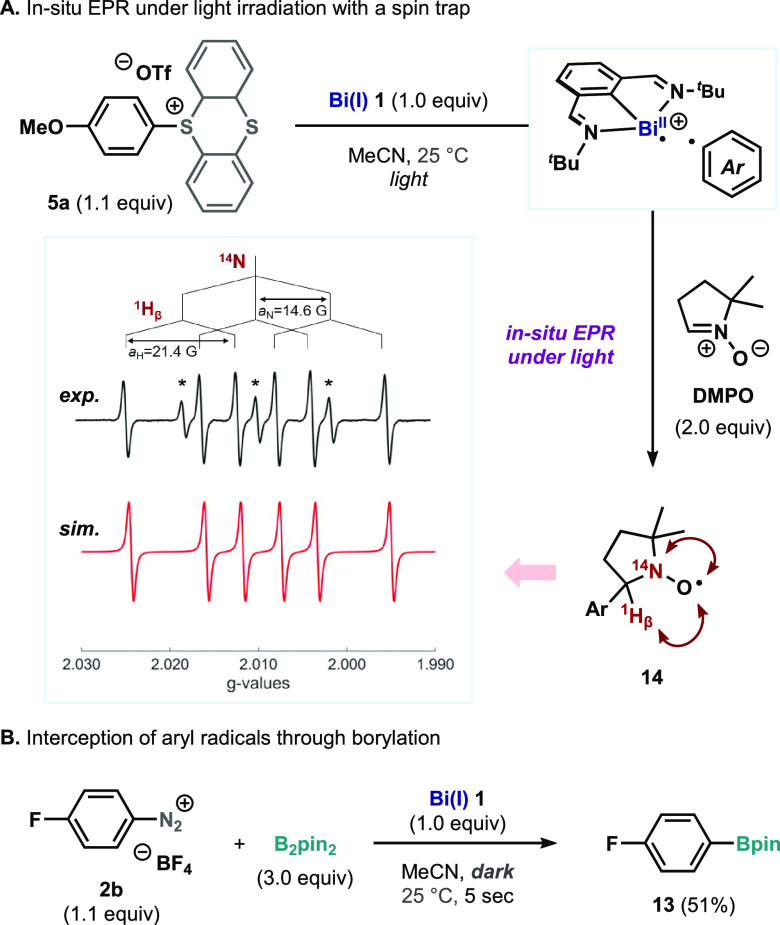
Interception and Detection of Aryl Radical Intermediates The signal marked with an asterisk (*) belongs to a known decomposition product of **14**.^21^

In an attempt to investigate the limits of this reactivity and emulate the classical behavior of transition metal complexes, we turned our attention to the possibility of engaging aryl halides. We started studying the reactivity between 4-iodobenzonitrile (*E*_red_ = −1.7 V vs SCE) and bismuthinidene **1** (Figure 3A). As expected, <10% conversion was observed in the dark or under ambient light, even at high temperatures. However, upon green or red light irradiation, **1** underwent smooth oxidative addition with **10** to afford aryl bismuthonium iodide **12a** in less than 24 h. Complex **12a** could be obtained as a yellow crystalline solid, which was analyzed by X-ray diffraction, thereby revealing that the iodide acts as a noncoordinating anion in the outer sphere. However, when the same reaction was carried out under blue light, a complex mixture of products was obtained because of decomposition of **12a** under these conditions. The ability of **12a** to absorb blue light but to remain unreactive transparent under red light illustrates the advantages in selectivity of using low-energy light in terms of functional group tolerance and stability. All these reactions follow a zero-order kinetic profile, and hence, the time to reach full conversion is only dependent on the number of photons absorbed (scale of the reaction, light penetration, etc.).^17^ We examined several red-light sources for the oxidative addition of 1-iodonaphthalene (*E*_red_ = −2.0 V vs SCE; Figure 3B). Red LED strips (maximum power output of ca. 20 W) afforded **12b** in 91% yield after 84 h (3.5 days). Conversely, two 660 nm Kessil lamps (maximum power output ca. 80 W) drove the reaction to completion in 24 h. Finally, monochromatic focused irradiation with a 660 nm laser allowed us to confirm unequivocally the ability of low-energy light to promote the reaction. On/off experiments highlight the requirement of continuous illumination.

**Figure 3:**
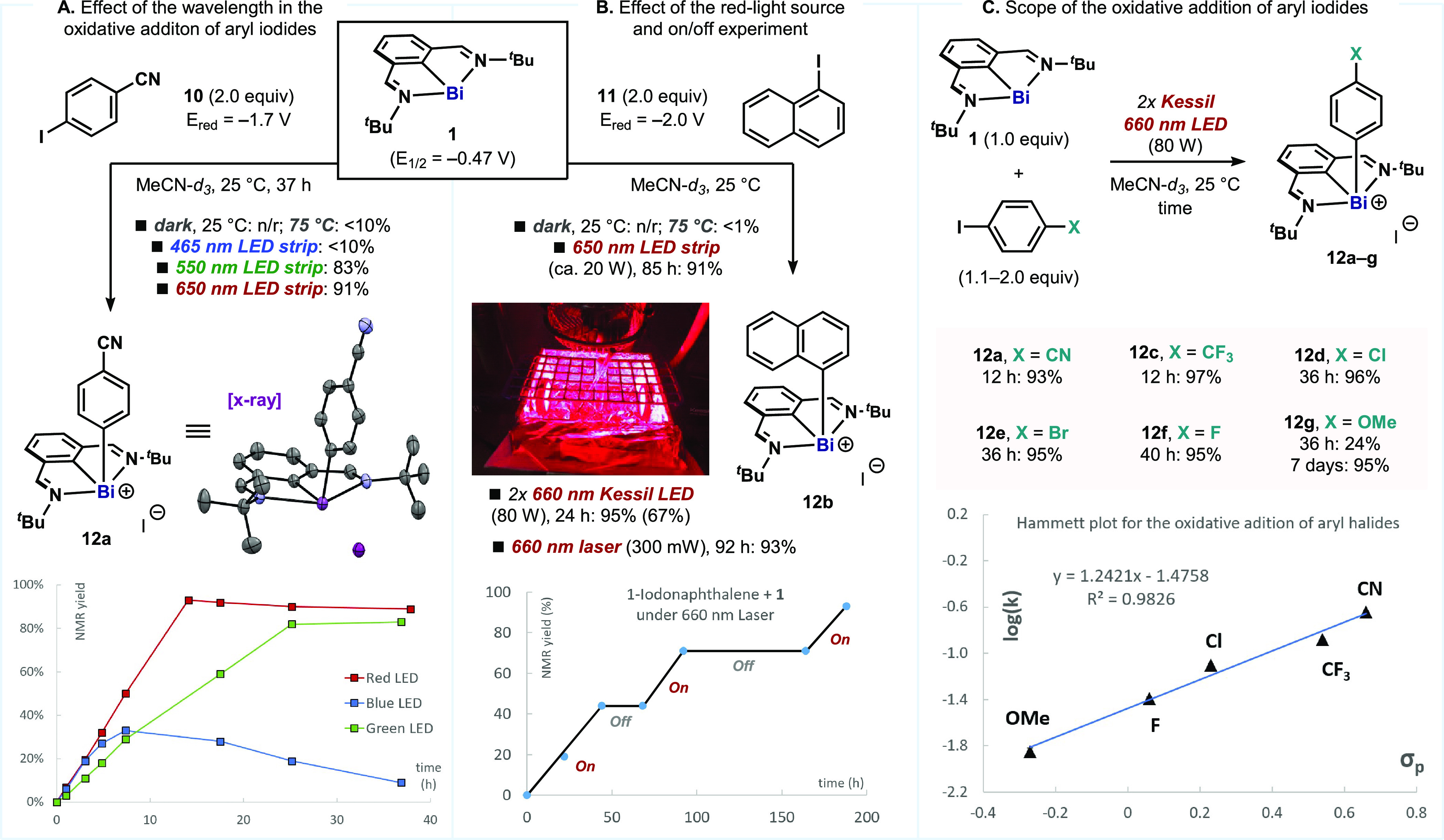
(A) Selective oxidative addition of 4-iodobenzonitrile into bismuthinidene **1**. (B) Effect of the red light source on the oxidative addition. (C) Scope and electronic effects of the red-light-promoted oxidative addition. Maximum power output of the light source as stated by the provider indicated in watts (W). Yields determined by ^1^H NMR using 1,3,5-trimethoxybenzene as internal standard. Isolated yields are in parentheses.

Finally, we explored the range of electronic properties of the aryl iodides that can be activated (Figure 3C). We found a clear correlation with Hammett σ_p_ values,^22^ where the process was found to be faster for electron-poor aryl groups. Nevertheless, adjustment of the reaction time allows activation of a wide range of aryl iodides with distinct electronic properties, from 4-iodobenzonitrile (**12a**, <24 h) to 4-iodoanisole (**12g**, 7 days). This electronic trend perfectly complements the reactivity of aryl thianthrenium salts: whereas electron-poor aryl halides react faster with **1**, aryl thianthrenium salts are easily accessible from electron-rich arenes.^18^ Although speculative at this point, the lower reaction rate of aryl iodides could be attributed to a slower fragmentation of these substrates^19,23^ or to a mechanistic divergence between the two processes (e.g., stepwise vs concerted SET/fragmentation).^24^

Preliminary orbital analysis by quantum chemical calculations using the ORCA package^25^ suggests that the absorption band in the red region corresponds to a metal-to-ligand charge transfer (MLCT) transition.^20,26^ The electron promoted to a ligand-centered orbital is responsible for the enhanced reducing ability of the complex (Scheme 3A).^27^ Presumably, this absorption can be attributed to a composite of not only S_0_ → S_*n*_, but also direct S_0_ → T_*n*_ transitions.^17^ Such spin-forbidden processes can be allowed in heavy-atom-containing molecules^28^ because of spin–orbit coupling,^29^ an effect that is particularly strong for bismuth,^30^ and has been largely exploited in the field of materials science.^31^ DFT analysis also allowed us to determine the Bi(I)*/Bi(II) redox potential of the highly reducing ^3^MLCT excited state of **1** to be −1.79 V vs SCE (Scheme 3B), which is consistent with the reduction potential of the aryl electrophiles that can be activated (ca. – 2 V).^23^

**Scheme 3:**
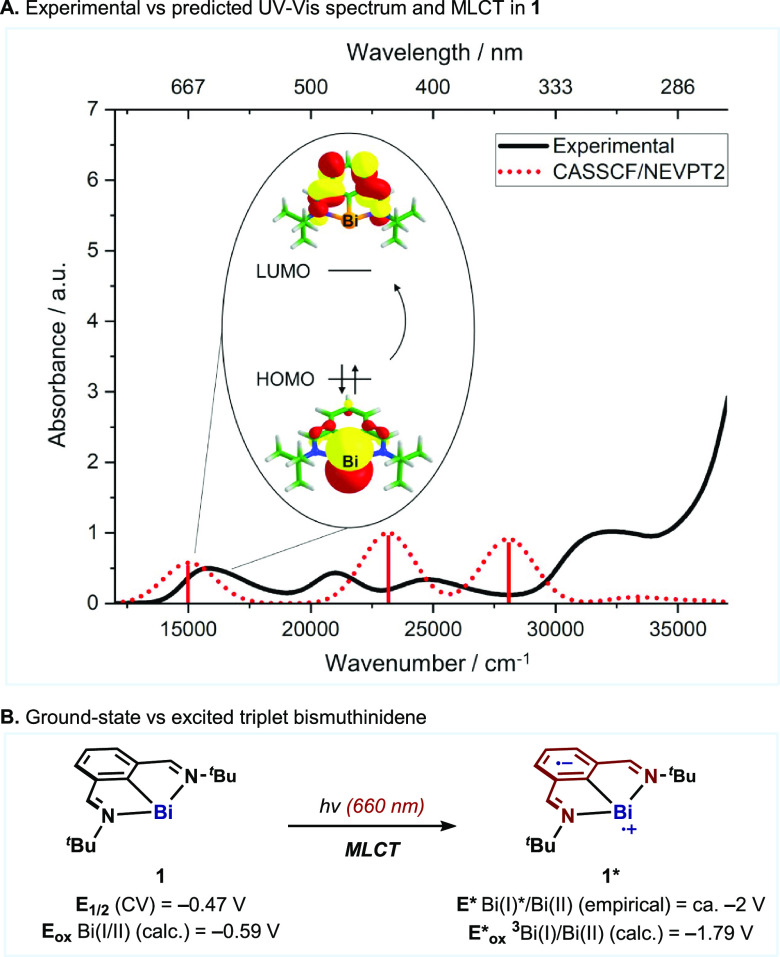
Theoretical and Experimental Analysis of the Excited States of **1**, with Potentials in V vs SCE

In summary, we present *N*,*C*,*N*-bismuthinidenes as a platform that unifies aryl oxidative additions into a well-defined main-group-element complex, which exploits both ground- and excited-state reactivity. Whereas aryl diazonium or iodonium salts react readily in the absence of light, aryl iodides and thianthrenium salts smoothly undergo formal oxidative addition under red light irradiation. This latter reactivity comes as a result of the increased reducing capability of the excited state of the bismuth(I) complexes upon absorption of low-energy visible light. Although exhaustive photophysical analysis of this system is still required to fully elucidate the mechanistic picture, these results pave the way for employing excited-state bismuthinidenes to explore unknown reactivity. Extension of this concept is currently being explored in our laboratories.

## Supporting Information Available

Additional experimental details, materials, methods (including photographs of the experimental setup), and full characterization data (^1^H, ^13^C, ^19^F NMR and HRMS) for all new compounds (PDF)
