# Supplementary material for: Oxidative Addition of Aryl Electrophiles into a Red-Light-Active Bismuthinidene
Source: J Am Chem Soc. 2023 Aug 21;145(34):18742–7. doi: 10.1021/jacs.3c06651 (PMC10472430; doi:10.1021/jacs.3c06651)
Supplement: Supplementary file 1 — ja3c06651_si_001.pdf [file ja3c06651_si_001.pdf]

Supporting Information

Oxidative Addition of Aryl Electrophiles into a Red Light-Active Bismuthinidene

Mauro Mato,[a] Paolo Cleto Bruzzese,[b] Fumiya Takahashi,[a] Markus Leutzsch,[a] Edward J. Reijerse,[b] Alexander Schnegg,[b] Josep Cornella[a,*]

[a] Max-Planck-Institut für Kohlenforschung, Kaiser-Wilhelm-Platz 1, Mülheim an der Ruhr, 45470, Germany. [b] Max-Planck-Institut für Chemische Energiekonversion, Stiftstrasse 34–36, Mülheim an der Ruhr, 45470, Germany

cornella@kofo.mpg.de

Table of contents

1. General considerations 3
2. Light sources and photoreaction set-up 4
3. Synthesis of bismuthinidenes 7
4. Preparation of starting materials 10
   4.1. General procedure A for the preparation of aryl diazonium salts 10
   4.2. General procedure B for the preparation of aryl thianthrenium salts 13
   4.3. Characterization of aryl thianthrenium salts 14
   4.4. Other electrophiles 15
5. Aryl oxidative additions into bismuth(I) 16
   5.1. General procedures for the aryl oxidative additions into bismuth(I) 16
   5.2. Characterization data of the oxidative addition adducts 19
   5.3. Solid-state structure of aryl bismuth(III) complexes 32
6. Electrochemical data 33
7. Interception of aryl radical intermediates 37
8. Photophysical properties 41
9. Computational study 46
10. Kinetic analysis and other mechanistic experiments 53
   10.1. Influence of the wavelength in the oxidative addition of aryl iodides 53
   10.2. On/off experiments with monochromatic 660 nm light 58
   10.3. Hammett correlation in the oxidative addition of aryl iodides 59
   10.4. Using all visible-light spectrum for the OA of aryl thianthrenium 61
11. Crystal data and structure refinement 62
12. DFT coordinates 79
13. NMR spectra 87
14. References 137

1. General considerations

Unless otherwise stated, all manipulations were performed under argon using standard Schlenk-line techniques or in an argon-filled glovebox.

Instruments

NMR data were recorded using a Bruker AVIII HD 300 MHz, Bruker AVIII HD 400 MHz, Bruker AVIII 500 MHz or Bruker AVNeo 600 MHz NMR spectrometer (at 298-300 K, unless stated otherwise). ^{1}H and ^{13}C chemical shifts are reported in ppm relative to the solvent residual peaks as an internal reference. For ^{1}H NMR the following residual proton peaks of the deuterated solvents were used: $CDCl_3$, $\delta_H(CHCl_3)$ 7.260; THF-d_8, $\delta_H((CD_2)_3CHDO)$ 3.580; CD_3CN, $\delta_H(CHD_2CN)$ 1.940; DMSO-d_6, $\delta_H(CD_2HSOCD_3)$ 2.500; DMF-d_7, $\delta_H(CHON(CD_3)_2)$ 8.03. For ^{13}C NMR: $CDCl_3$, δ 77.16; THF-d_8, δ 67.57; CD_3CN, δ 1.32; DMSO-d_6, δ 39.52; DMF-d_7, δ 163.15/34.89/29.76. ^{13}C spectra were acquired with broadband ^{1}H decoupling unless mentioned otherwise. Chemical shifts (δ) are given in ppm, relative to deuterated solvent residual peak, and coupling constants (*J*) provided in Hz. ^{19}F NMR shifts are reported relative to the ^{19}F resonances of $CFCl_3$. ^{19}F data at 470 and 282 MHz NMR is generally reported with ^{1}H decoupling, and ^{19}F data at 565 MHz NMR is reported without ^{1}H decoupling due to hardware limitations. Mass spectra were acquired using the following instruments: EI - Finnigan MAT 8200 (70 eV); ESI-MS - Bruker ESQ 3000. Accurate mass determinations: Bruker APEX III FT-MS (7 T magnet) or Finnigan MAT 95. Chromatographic purifications were performed by flash column chromatography using Merck silica gel 60 (40-63 µm) or by preparative TLC using PLC Silica gel 60 F_{254}, 1 mm, 20x20 cm (Sigma-Aldrich).

Solvents and reagents

Anhydrous MeCN and THF (and their deuterated versions) were distilled from CaH_2, degassed through 3 freeze-pump-thaw cycles, and stored over 3 Å molecular sieves under argon. Alternatively, small amounts (<50 mL) of freshly purchased solvents (which are reasonably free of water) could also be first submitted to 3 freeze-pump-thaw cycles, and then left under 3 Å molecular sieves overnight. We found this treatment to be enough for carrying out the low-valent bismuth chemistry described herein. Molecular sieves were activated at 200 °C under high vacuum for 3 days. Unless otherwise stated, solvents used for the preparation of the ligands, catalysts or starting materials were also anhydrous, but not degassed nor stored over molecular sieves. Anhydrous $BiCl_3$ (99.9%, trace metal basis) was purchased from Alfa Aesar and stored in the glovebox. Unless otherwise noted, all reagents were obtained from commercial suppliers and used without further purification.

2. Light sources and photoreaction set-up

LED Strips:

Blue (465 nm, 100 cm/140 LEDs, max. output ca. 19 W; max. luminous flow 400 lumen), green (550 nm, 100 cm/140 LEDs, max. output ca. 20 W; max. luminous flow 1360 lumen) and red (650 nm , 100 cm/140 LEDs, max. output ca. 21 W; max. luminous flow 1000 lumen) LED-strip irradiation was performed with 100 cm of 24V DC LED strips strapped around a 15-cm glass crystalizing dish. These LED strips were purchased from LEDs24. White LEDs are LED light 24 V from LUMOtech®. The reactions were run in vials or NMR tubes assembled inside the dish surrounded by the LED strip, while the temperature was maintained close to room temperature by using a cooling fan placed on top of the reactor. The reactor was set up on top of a stirring plate. The walls of the reactor were covered with aluminum foil.

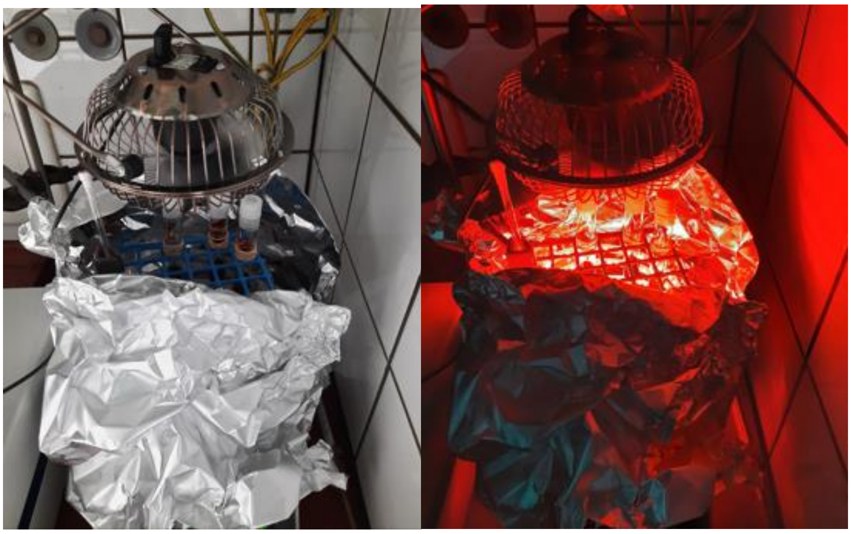

Figure S1. Lights off and on in a representative red-LED strip irradiation set up.

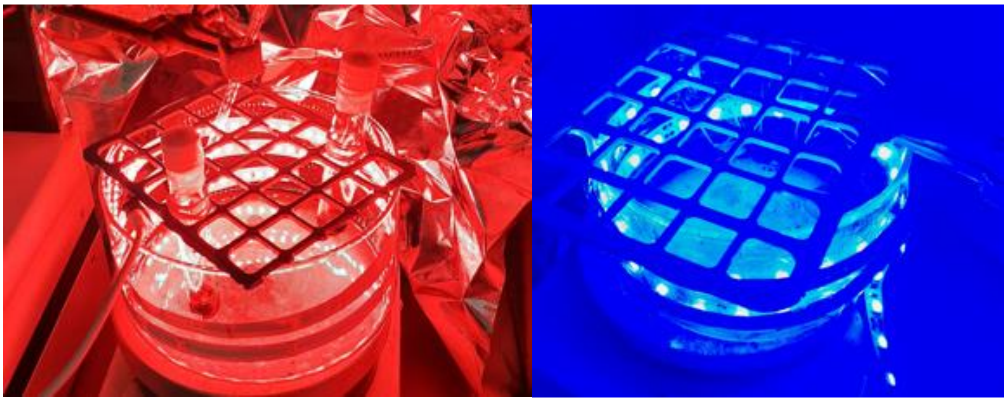

Figure S2. Red-light and blue-light strip irradiation set up.

Purple (395 nm), dark-blue (420 nm) and blue (465 nm) irradiation was performed using a standardized Penn PhD photoreactor M2, at 50% intensity. These light sources were used only for comparison purposes, when specified, and not as standard method for this study.

Different red-light sources

Red-light irradiation was performed either with the 650 nm LED strip, with two 660 nm Kessil lamps at full intensity, or with a 300 mW 600 nm monochromatic laser.

Kessil lamps: Unless mentioned otherwise, the reactions carried out under red-light irradiation were performed using two standardized 660 nm LED PR160L lamps purchased from Kessil.

The inside of the reactor box was fully covered with aluminum foil. The two Kessil lamps were assembled opposite to one another through two holes in the walls of the reactor. As suggested by the manufacturer, the two lamps were assembled at ca. 12 cm from each other. The reaction vessels will be then located at ca. 6 cm from each lamp, receiving an average intensity of 159 mW/cm^2 (see https://kessil.com/products/science_PR160L.php for light-intensity maps and other details). Unless stated otherwise, both PR160L Kessil lamps were used at the 100% intensity setting. The reactor was set up on top of a stirring plate, and the temperature of the reactor was lowered by using a cooling fan placed on top (note: the temperature of the reaction mixtures was found to be around 40 °C using this set up, so thermal control experiments at higher temperatures but without light irradiation were run whenever necessary, resulting in no reactivity). Finally, during extended reaction times, the lateral of the reactor was covered with aluminum foil.

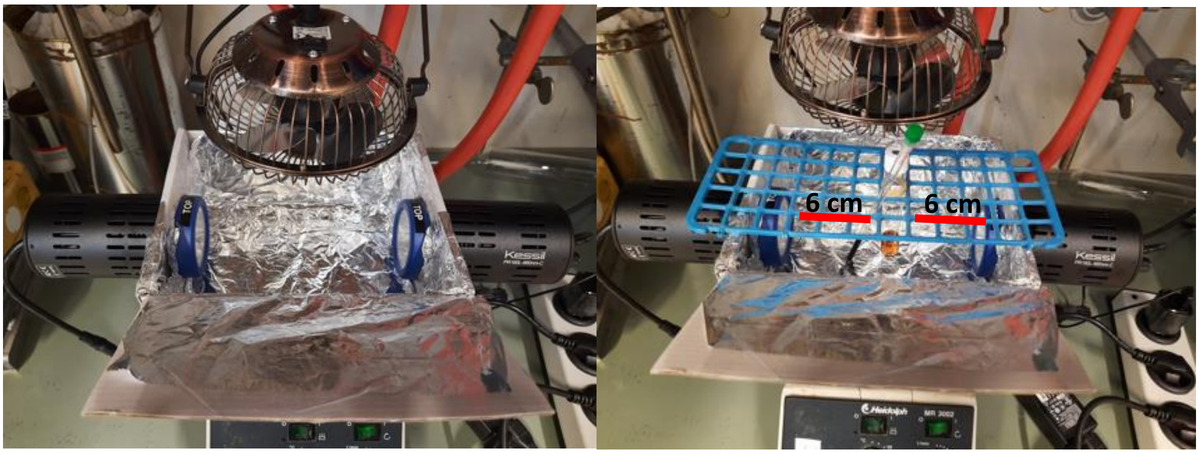

Figure S3. Set up with 2x Kessil 660 nm LED PR160L lamps. The reactions sit at ca. 6 cm from each LED.

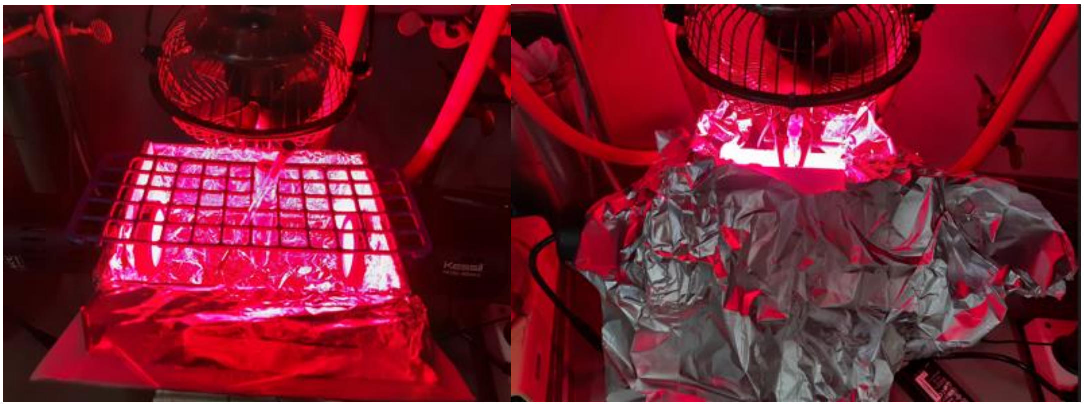

Figure S4. Kessil LED set up with lights on, uncovered (left) or covered with aluminum foil.

Monochromatic 660 nm laser: In a room with the lights off, a monochromatic 660 nm red laser was pointed to the reaction vessel (usually an NMR tube; the focused light covered ca. 10% of the length of the height solution, relying on diffusion for the reaction to proceed to completion). The output of the focused red light is 300 mW.

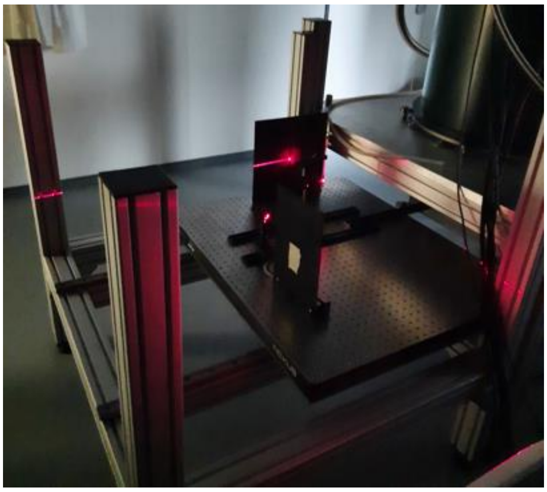

Figure S5. 300 mW 660 nm laser pointed to the reaction NMR tube.

3. Synthesis of bismuthinidenes

The key bismuthinidene used in this study (**1**) was prepared by scaling up a reported procedure, in two steps from the corresponding aryl bromide **S1**.[1,2]

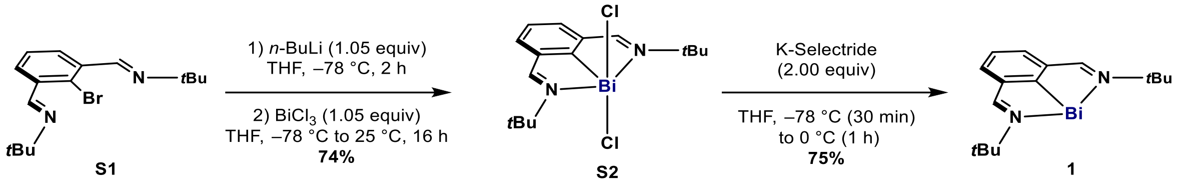

Dichlorobismuthine **S2**: A heat gun-dried 250 mL Schlenk flask with a magnetic stirring bar was charged with (1*E*,1'*E*)-1,1'-(2-bromo-1,3-phenylene)bis(*N*-tert-butylmethanimine) (**S1**) (3.00 g, 9.28 mmol, 1.00 equiv), and it was dissolved in dry THF (100 mL, ca. 0.1 M). The mixture was cooled down to –78 °C in an acetone/dry ice bath, and to this mixture was added *n*-BuLi dropwise (2.5 M in hexanes, 3.90 mL, 1.05 equiv). The resulting solution was stirred at –78 °C for 2 h. After this time, this solution was transferred in a single portion via cannula to a 250 mL Schlenk flask containing a solution of $BiCl_3$ (3.07 g, 9.74 mmol, 1.05 equiv) in dry THF (60 mL, ca. 0.15 M) under Ar, also pre-cooled to –78 °C. After stirring for 5 min at –78 °C, the cooling bath was removed and the mixture was allowed to stir at room temperature for 16 h. After this time a yellow suspension was obtained, and the rest of the procedure was carried out under air. THF was removed in vacuum, and the obtained residue was re-suspended in 200 mL of HPLC-grade CH_2Cl_2. This was filtered through a plug of Celite®, which was washed with 2 x 100 mL of CH_2Cl_2. The volume of d CH_2Cl_2 was reduced to ca. 75 mL in vacuum, and then, 100 mL of hexanes were added. A precipitate was obtained, which was filtered through a filtering plate, washed twice (2 x 25 mL) with pentane, and dried in high vacuum, to afford **S2** (3.60 g, 74%) as a white solid, which was carried to the next step without further purification. **^{1}H NMR** (300 MHz, $CDCl_3$) δ 9.61 (s, 2H), 8.17 (d, *J* = 7.5 Hz, 2H), 7.85 (dd, *J* = 7.8, 7.2 Hz, 1H), 1.60 (s, 18H).[1,2]

Bismuthinidene **1**: A heat gun-dried 250 mL Schlenk flask with a magnetic stirring bar was charged with **S2** (1.80 g, 3.44 mmol, 1.00 equiv), which was dissolved under argon in anhydrous THF (60 mL, 0.06 M), and cooled down to –78 °C in an acetone/dry ice bath. To this mixture was added dropwise K-Selectride (1.0 M in THF, 6.90 mL, 2.00 equiv) and the resulting dark green mixture was stirred at –78 °C for 30 min and then warmed to 0 °C in an ice/water bath, and stirred for 1 h. Then, THF was removed slowly in vacuum, and the resulting residue was dried in high vacuum for 2 h. The crude was redissolved in anhydrous pentane (ca. 75 mL, in three portions), and filtered through a heat gun-dried glass funnel under argon, into another 250 mL Schlenk flask. The volume of the filtrate was reduced to ca. 20 mL in vacuum, and the solution was cooled to –20 °C overnight. After this time, **1** was obtained as dark green crystalline needles, from which the mother liquor was removed via cannula before drying in high vacuum. The product (1.15 g, 75%) was stored in an argon-filled glovebox. **^{1}H NMR** (300 MHz, THF-d_8) δ 9.81 (s, 2H), 7.94 (d, *J* = 7.4 Hz, 2H), 7.08 (t, *J* = 7.4 Hz, 1H), 1.57 (s, 18H).[1,2]

The bismuthinidene used for the synthesis of **3c** was prepared according to a reported procedure.[3]

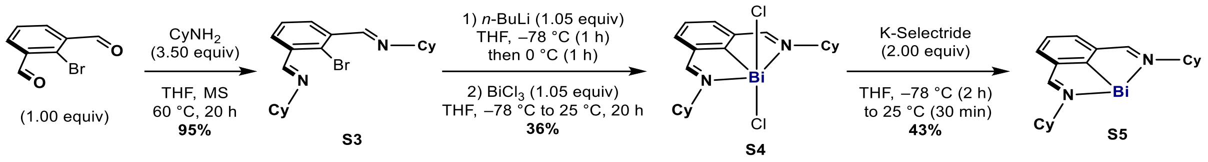

(*E*)-1-(2-Bromo-3-((*E*)-(cyclohexylimino)methyl)phenyl)-N-cyclohexylmethanimine (**S3**).[4] A 250 mL two-necked round-bottomed flask with a magnetic stirring bar and a reflux condenser was charged, under argon, with 2-bromoisophthalaldehyde (3.00 g, 14.1 mmol, 1.00 equiv), cyclohexylamine (4.90 g, 49.0 mmol, 3.50 equiv) and activated 3 Å molecular sieves. To this were added 80 mL of dry THF, and the resulting mixture was stirred at 60 °C for 20 h. After cooling down to room temperature, the mixture was filtered through a pad of Celite®, which was washed with another 100 mL of THF. After removing the solvent and drying the resulting solid in high vacuum for 2 days (to remove residual cyclohexylamine), 5.05 g of the desired bisimine **S3** (95%) was obtained as a pale orange solid. The product was used in the next step without further purification. **^{1}H NMR** (300 MHz, $CDCl_3$) δ 8.74 (s, 2H), 8.01 (d, *J* = 7.7 Hz, 2H), 7.32 (t, *J* = 7.7 Hz, 1H), 3.30 (tt, *J* = 10.3, 4.1 Hz, 2H), 1.90 – 1.51 (m, 16H), 1.45 – 1.24 (m, 6H). **^{13}C NMR** (75 MHz, $CDCl_3$) δ 157.63, 135.60, 130.67, 127.42, 126.75, 69.80, 34.28, 25.58, 24.66.

Biscyclohexyl dichlorobismuthine **S4**: A heat gun-dried 100 mL Schlenk flask with a magnetic stirring bar was charged with (*E*)-1-(2-Bromo-3-((*E*)-(cyclohexylimino)methyl)phenyl)-N-cyclohexylmethanimine (**S3**) (1.60 g, 4.26 mmol, 1.00 equiv), and it was dissolved in dry THF (50 mL). The mixture was cooled down to –78 °C in an acetone/dry ice bath, and to this mixture was added *n*-BuLi dropwise (2.5 M in hexanes, 1.79 mL, 1.05 equiv). The resulting solution was stirred at –78 °C for 1 h. After this time, the solution was warmed up to 0 °C, and stirred at this temperature for 1 h. Then, this solution was transferred in a single portion via cannula to a 250 mL Schlenk flask containing a solution of $BiCl_3$ (1.41 g, 4.48 mmol, 1.05 equiv) in dry THF (30 mL, 0.12 M) under Ar at –78 °C. After stirring for 30 min at –78 °C, the cooling bath was removed and the mixture was allowed to stir at room temperature for 20 h. After this time a yellow suspension was obtained, and the rest of the procedure was carried out under air. THF was removed in vacuum, and the obtained residue was redisolved in 100 mL of HPLC-grade CH_2Cl_2. This was filtered through a plug of Celite®, which was washed with 2 x 100 mL of CH_2Cl_2. The volume of CH_2Cl_2 was reduced to ca. 30 mL in vacuum, and then, 30 mL of hexanes were added. A precipitate was obtained, which was filtered through a filtering plate, washed twice (2 x 25 mL) with pentane, and dried in high vacuum, to afford **S4** (0.90 g, 36%) as a white solid, which was carried to the next step without further purification. **^{1}H NMR** (600 MHz, THF-*d₈*) δ 9.70 (d, *J* = 1.0 Hz, 2H), 8.15 (d, *J* = 7.5 Hz, 2H), 7.76 (t, *J* = 7.5 Hz, 1H), 3.63 (ttd, *J* = 11.2, 4.2, 1.1 Hz, 2H), 2.13 – 2.05 (m, 4H), 1.93 – 1.85 (m, 4H), 1.73 – 1.70 (m, 6H), 1.42 (tdd, *J* = 16.4, 9.7, 3.4 Hz, 4H), 1.32 (tt, *J* = 12.6, 3.5 Hz, 2H). **^{13}C NMR** (151 MHz, THF-d_8) δ 209.82, 170.91, 147.98, 135.98, 129.10, 68.79, 36.17, 26.02, 25.55. **HRMS (ESI)**: calculated for $C_{20}H_{27}BiClN_2$ $[M-Cl]^+$: 539.16613; found: 539.16586.

Biscyclohexyl bismuthinidene **S5**: A heat gun-dried 100 mL Schlenk flask with a magnetic stirring bar was charged with **S4** (0.53 g, 0.92 mmol, 1.00 equiv), which was dissolved under argon in anhydrous THF (15 mL, 0.075 M), and cooled down to –78 °C in an acetone/dry ice bath. To this mixture was added dropwise K-Selectride (1.0 M in THF, 1.84 mL, 2.00 equiv) and the resulting dark green mixture

was stirred at –78 °C for 30 min and then stirred at room temperature for 1 h. Then, THF was removed in vacuum. The crude was redissolved in ca. 20 mL of anhydrous pentane, and filtered through a heat gun-dried glass funnel under argon, into another Schlenk flask. The volume of the filtrate was reduced to ca. 5 mL in vacuum, and the solution was cooled to –20 °C overnight. After this time, **S5** was obtained as very dark green crystals, from which the mother liquor was removed via cannula before drying in high vacuum. The product (200 mg, 43%) was stored in an argon-filled glovebox. **^{1}H NMR** (600 MHz, THF-*d₈*) δ 9.76 (d, *J* = 0.9 Hz, 2H), 7.92 (d, *J* = 7.4 Hz, 2H), 7.07 (t, *J* = 7.4 Hz, 1H), 3.74 – 3.67 (m, 2H), 2.14 – 2.05 (m, 4H), 1.91 – 1.83 (m, 4H), 1.73 – 1.67 (m, 6H), 1.54 – 1.46 (m, 4H), 1.37 – 1.30 (m, 2H). **^{13}C NMR** (151 MHz, THF-*d₈*) δ 198.14, 166.49, 145.75, 133.82, 122.47, 69.54, 40.89, 26.48, 26.18. **HRMS (ESI)**: calculated for $C_{20}H_{27}BiN_2$ $[M]^+$: 504.19727; found: 504.19735.

4. Preparation of starting materials

4.1. General procedure A for the preparation of aryl diazonium salts

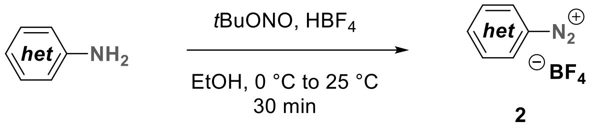

Under air, a 25 mL round bottom flask with a magnetic stirring bar was charged with the corresponding aniline (1 equiv) and aqueous HBF_4 (48% in water, 2 equiv), and both were dissolved in absolute ethanol (2-3 mL, ca. 1.5 M). The mixture was stirred until complete homogeneity and cooled down to 0 °C with a water/ice bath. Then, *t*BuONO (90%, 2 equiv) was added dropwise, and the resulting reaction mixture was stirred at room temperature for 30 min. At this point, in most reactions the product crashed out from the mixture. Then, ca. 10 mL of diethyl ether were added to fully precipitate the aryl diazonium salt. The solid was transferred to a frit, and washed three times with diethyl ether. This afforded pure material, which was dried in high vacuum prior to use.

Safety notes

- Aryl diazonium salts are highly energetic compounds. Even though all the compounds used in this studied are well reported, widely used (even some commercially available) and generally safe to work with at small scale, special care should be taken while preparing or handling them.
- Whereas the preparation of mesityl diazonium tetrafluoroborate **2h** appeared successful, it decomposed (non-violently, it turned into an oil) upon standing overnight under Ar at room temperature. Thus, special care should be taken if this, or other sterically crowded aryl diazonium salts were to be prepared or handled.
- In our experience, besides the aforementioned exception, the rest of the compounds were stable while stored in glass vials under argon, in the absence of light at room temperature for several months.

Characterization of aryl diazonium salts

The following aryl diazonium salts were prepared. All of them are well-known compounds, and characterization data matched the reported ones. For reference, ^{1}H NMR data is included below.[5]

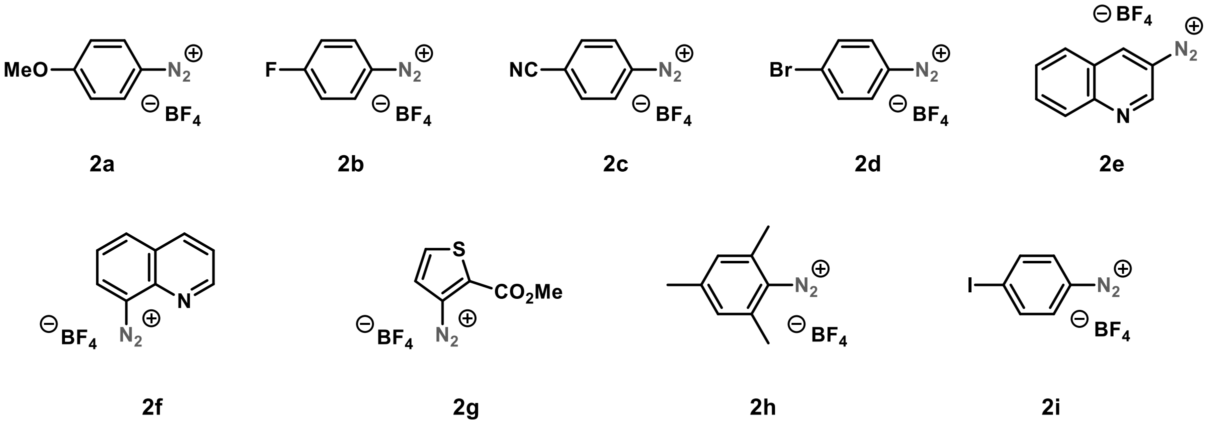

Methoxybenzene-4-diazonium tetrafluoroborate (**2a**)

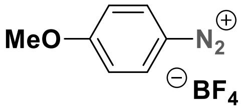

The title product was obtained as a white solid in 95% yield (1.1 g) from the corresponding aniline (5.0 mmol), following General Procedure A. Characterization data matched previously reported ones.[5]

^{1}H NMR (300 MHz, CD_3CN) δ 8.49 – 8.38 (m, 2H), 7.43 – 7.32 (m, 2H), 4.09 (s, 3H).

Fluorobenzene-4-diazonium tetrafluoroborate (**2b**)

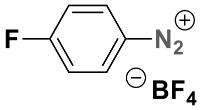

The title product was obtained as a white crystalline solid in 88% yield (0.65 g) from the corresponding aniline (3.5 mmol), following General Procedure A. Characterization data matched previously reported ones.[5]

^{1}H NMR (300 MHz, DMSO-d_6) δ 8.87 – 8.77 (m, 2H), 7.95 – 7.85 (m, 2H).

Benzonitrile-4-diazonium tetrafluoroborate (**2c**)

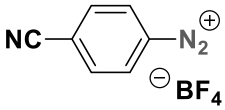

The title product was obtained as a white solid in 81% yield (0.35 g) from the corresponding aniline (2.0 mmol), following General Procedure A. Characterization data matched previously reported ones.[5]

^{1}H NMR (300 MHz, DMSO-d_6) δ 8.84 (d, J = 8.6 Hz, 2H), 8.46 (d, J = 8.6 Hz, 2H).

Bromobenzene-4-diazonium tetrafluoroborate (**2d**)

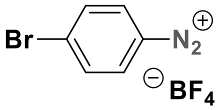

The title product was obtained as a white solid in 87% yield (0.48 g) from the corresponding aniline (2.0 mmol), following General Procedure A. Characterization data matched previously reported ones.[5]

^{1}H NMR (300 MHz, DMSO-d_6) δ 8.62 – 8.55 (m, 2H), 8.30 – 8.21 (m, 2H).

Quinoline-3-diazonium tetrafluoroborate (**2e**)

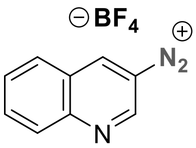

The title product was obtained as a beige solid in 80% yield (0.39 g) from the corresponding aniline (2.0 mmol), following General Procedure A. Characterization data matched previously reported ones.[5]

^{1}H NMR (300 MHz, DMSO-d_6) δ 9.97 (d, J = 2.5 Hz, 1H), 9.74 (d, J = 2.4 Hz, 1H), 8.48 (d, J = 8.2 Hz, 1H), 8.35 – 8.32 (m, 2H), 8.06 – 8.02 (m, 1H).

Quinoline-8-diazonium tetrafluoroborate (**2f**)

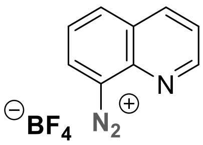

The title product was obtained as a light orange solid in 82% yield (0.40 g) from the corresponding aniline (2.0 mmol), following General Procedure A. Characterization data matched previously reported ones.[5]

^{1}H NMR (300 MHz, DMSO-d_6) δ 9.43 – 9.37 (m, 1H), 9.35 (d, *J* = 6.2 Hz, 1H), 9.02 – 8.97 (m, 1H), 8.89 (d, *J* = 8.2 Hz, 1H), 8.18 (d, *J* = 8.4 Hz, 1H), 8.07 – 8.03 (m, 1H).

2-(Methoxycarbonyl)thiophene-3-diazonium tetrafluoroborate (**2g**)

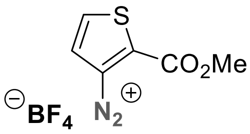

The title product was obtained as a yellow solid in 92% yield (0.47 g) from the corresponding aniline (2.0 mmol), following General Procedure A. Characterization data matched previously reported ones.[5]

^{1}H NMR (300 MHz, DMSO-d_6) δ 8.36 (d, *J* = 5.5 Hz, 1H), 8.28 (d, *J* = 5.5 Hz, 1H), 4.03 (s, 3H).

Iodobenzene-4-diazonium tetrafluoroborate (**2i**)

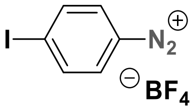

The title product was obtained as a white solid in 82% yield (0.52 g) from the corresponding aniline (2.0 mmol), following General Procedure A. Characterization data matched previously reported ones.[5]

^{1}H NMR (300 MHz, DMSO-d_6) δ 8.43 (d, *J* = 8.8 Hz, 2H), 8.34 (d, *J* = 8.9 Hz, 2H).

4.2. General procedure B for the preparation of aryl thianthrenium salts

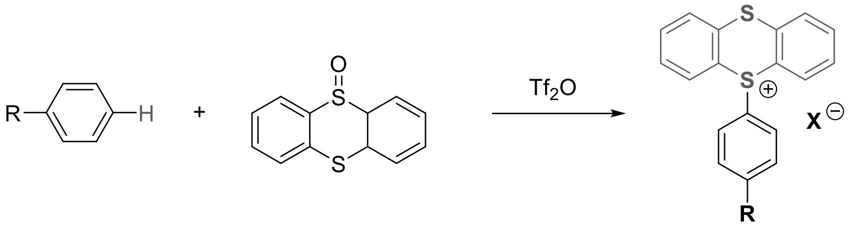

Procedure B1 with OTf counteranion: Following a published report,[6] a two-neck round-bottom flask was charged, under argon, with thianthrene-*S*-oxide (TTO) (1.0 equiv) and the corresponding arene (1.0 equiv), and both were dissolved in dry CH_2Cl_2 (ca. 0.15 M). The reaction mixture was cooled to −40 °C before trifluoromethanesulfonic anhydride (1.2 equiv) was added dropwise. The reaction mixture was stirred at −40 °C for 30 min and the allowed to stir at room temperature overnight. The mixture was diluted in CH_2Cl_2 (ca. 30 mL/mmol) and quenched with saturated aqueous $NaHCO_3$ (ca. 30 mL/mmol). The aqueous phase was extracted twice with CH_2Cl_2. The combined organic fractions were dried over anhydrous Na_2SO_4, filtered, and concentrated under reduced pressure. Further purification was achieved by dissolving the products in CH_2Cl_2 (ca. 5 mL/mmol), filtration, and precipitation with diethyl ether (ca. 25 mL/mmol).

Procedure B2 with BF_4 counteranion: Following a published report,[7] a two-neck round-bottom flask was charged under argon with an arene (1.0 equiv), which was dissolved in dry acetonitrile (ca. 0.15 M). After cooling down to 0 °C in a water/ice bath, $HBF_4{\cdot}OEt_2$ (1.2 equiv + 1.0 extra equiv per basic functional group) was added while stirring. After 5 min, thianthrene-*S*-oxide (TTO) (1.0 equiv) was added in one portion to the solution at 0 °C. Subsequently, trifluoromethanesulfonic anhydride (3.0 equiv) was added in one portion, which results in a change in color to deep purple. The resulting solution was left stirring, while warming up to room temperature overnight, resulting in the slow purple color fading away. The mixture was diluted in CH_2Cl_2 (ca. 30 mL/mmol) and quenched with water (ca. 15 mL/mmol) and saturated aqueous Na_2CO_3 (ca. 15 mL/mmol). The biphasic mixture was poured into a separatory funnel and shaken energetically before the phases were separated. The organic fraction was washed with aqueous $NaBF_4$ (5% w/w, 2 x ca. 20 mL) and with water (2 x ca. 20 mL). Then, the organic fraction was dried over anhydrous $MgSO_4$, filtered, and the solvent was removed in vacuum. Further purification was achieved by dissolving the products in CH_2Cl_2 (ca. 5 mL/mmol), filtration, and precipitation with diethyl ether (ca. 25 mL/mmol).

4.3. Characterization of aryl thianthrenium salts

5-(4-Methoxyphenyl)-5H-thianthren-5-ium trifluoromethanesulfonate (**5a**)

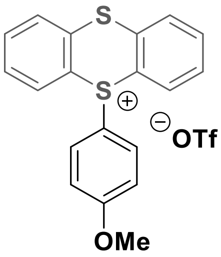

The title product was obtained as a white solid in 88% yield (1.25 g) following General Procedure B1, from anisole (324 mg, 3.00 mmol), thianthrene oxide (1.00 equiv) and Tf_2O (1.50 equiv).

^{1}H NMR (400 MHz, CD_3CN) δ 8.30 (dd, *J* = 7.9, 1.4 Hz, 2H), 7.98 – 7.93 (m, 2H), 7.88 (td, *J* = 7.7, 1.4 Hz, 2H), 7.83 – 7.77 (m, 2H), 7.23 – 7.17 (m, 2H), 7.07 – 7.01 (m, 2H), 3.82 (s, 3H).

^{13}C NMR (101 MHz, CD_3CN) δ 163.62, 136.03, 134.88, 134.32, 130.57, 130.55, 129.95, 121.12 (q, *J* = 320.2 Hz) 119.41, 116.32, 113.15, 55.84.

^{19}F NMR (282 MHz, CD_3CN) δ -79.27.

HRMS (ESI): calculated for $C_{19}H_{15}OS_2$ [M-OTf]$^+$: 323.05588; found: 323.05567.

5-(4-(4-(2-(Pyridin-2-yloxy)propoxy)phenoxy)phenyl)-5*H*-thianthren-5-ium tetrafluoroborate (**5b**)

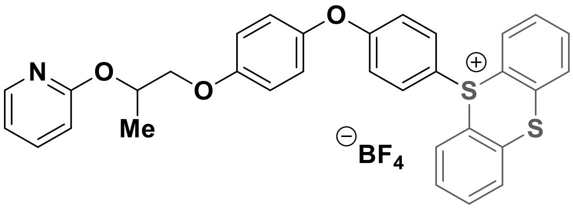

The title product was obtained as a white solid in 80% yield (250 mg) following General Procedure B2, from pyriproxifen (161 mg, 0.50 mmol), thianthrene oxide (1.00 equiv), Tf_2O (2.00 equiv) and $HBF_4{\cdot}Et_2O$ (2.20 equiv).

^{1}H NMR (400 MHz, CD_3CN) δ 8.34 – 8.30 (m, 2H), 8.15 (ddd, *J* = 5.0, 2.0, 0.8 Hz, 1H), 7.98 (ddd, *J* = 7.9, 1.5, 0.4 Hz, 2H), 7.90 (td, *J* = 7.7, 1.4 Hz, 2H), 7.81 (ddd, *J* = 8.0, 7.5, 1.4 Hz, 2H), 7.66 (ddd, *J* = 8.4, 7.1, 2.0 Hz, 1H), 7.19 – 7.12 (m, 2H), 7.00 – 6.93 (m, 7H), 6.73 (dt, *J* = 8.3, 0.9 Hz, 1H), 5.62 – 5.53 (m, 1H), 4.22 – 4.11 (m, 2H), 1.42 (d, *J* = 6.5 Hz, 3H).

^{13}C NMR (101 MHz, CD_3CN) δ 163.14, 162.98, 156.42, 147.82, 146.86, 139.17, 136.25, 135.00, 134.52, 130.63, 129.95, 121.83, 119.12, 118.30, 117.34, 116.98, 116.07, 115.02, 111.21, 70.93, 69.23, 15.97.

^{19}F NMR (282 MHz, CD_3CN) δ -151.8.

HRMS (ESI): calculated for $C_{32}H_{26}NO_3S_2$ [M-BF_4]$^+$: 536.13486; found: 536.13492.

5-(4-((*N*,4-Dimethylphenyl)sulfonamido)phenyl)-5*H*-thianthren-5-ium tetrafluoroborate (5c)

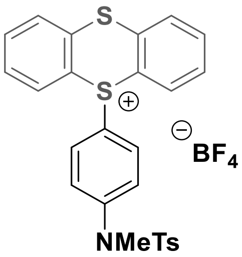

The title product was obtained as a white solid in 73% yield (410 mg) following General Procedure B2, from *N*,4-dimethyl-N-phenylbenzenesulfonamide (261 mg, 1.00 mmol), thianthrene oxide (1.00 equiv), Tf_2O (2.00 equiv) and $HBF_4 \cdot Et_2O$ (2.20 equiv).

^{1}H NMR (400 MHz, CD_3CN) δ 8.40 (dd, *J* = 7.8, 1.4 Hz, 2H), 8.01 (dd, *J* = 7.9, 1.4 Hz, 2H), 7.93 (td, *J* = 7.7, 1.4 Hz, 2H), 7.85 (td, *J* = 7.7, 1.4 Hz, 2H), 7.38 – 7.29 (m, 4H), 7.29 – 7.24 (m, 2H), 7.10 – 7.05 (m, 2H), 3.12 (s, 3H), 2.41 (s, 3H).

^{13}C NMR (101 MHz, CD_3CN) δ 145.84, 144.83, 136.61, 135.25, 135.11, 132.96, 130.75, 130.00, 129.72, 128.73, 127.50, 126.65, 120.92, 118.63, 37.01, 20.58.

^{19}F NMR (282 MHz, CD_3CN) δ -151.7.

HRMS (ESI): calculated for $C_{26}H_{22}NO_2S_3$ $[M-BF_4]^+$: 476.08072; found: 476.08061.

4.4. Other electrophiles

The rest of the electrophiles employed in this study (listed below) are commercially available. They were purchased and used without further purification.

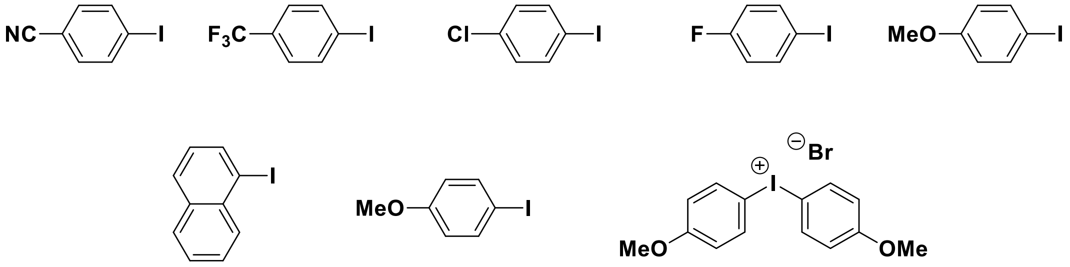

5. Aryl oxidative additions into bismuth(I)

5.1. General procedures for the aryl oxidative additions into bismuth(I)

General procedure C1 for the oxidative addition of aryl diazonium salts

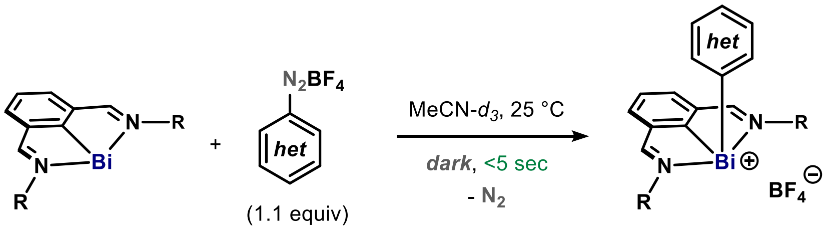

In an argon-filled glovebox, an oven-dried Schlenk flask or a glass vial with a magnetic stirring bar was charged with a bismuthinidene (1.0 equiv), and it was dissolved in anhydrous degassed MeCN-d_3 (0.1 M). To this stirred solution was added dropwise another solution of the corresponding aryl diazonium salt (1.1 equiv, 0.1 M in MeCN-d_3). Immediate evolution of nitrogen gas was observed, and color changed from dark green to light yellow/orange upon addition of the full equivalent of electrophile (aryl diazonium salts can also be added as solids in a single portion, but addition of a solution allows for a titration-like control of the addition of 1.0 equiv, due to the significant color change when all bismuth(I) has been consumed). Then, for characterization purposes, the resulting solution was transferred into an NMR tube, which was closed and taken out of the glovebox. Due to the formation of minor side products (mainly from direct reaction of aryl radical with the solvent before recombination with bismuth(II), see section **7** for details), the accurate yield of the oxidative addition was determined by NMR by repeating the reaction in the presence of 1 equiv of 1,3,5-trimethoxybenzene as internal standard. Unless otherwise stated, full conversion was observed for the starting bismuth(I) complex.

For preparative scale (**3d**), the reaction was carried out in non-deuterated MeCN. The resulting solution of oxidative-addition complex was taken out of the glovebox and the solvent was removed in vacuum (rotary evaporator), giving a light yellow solid.

For the oxidative addition of bis-(4-methoxyphenyl) iodonium bromide **6**, the same procedure was followed. HRMS and NMR spectra matched that for the ones obtained from the (4-methoxyphenyl) diazonium tetrafluoroborate (with a small shift in ^{1}H NMR spectra due to the change of counteranion).

General procedure C2 for the red-light promoted oxidative addition of aryl thianthrenium salts

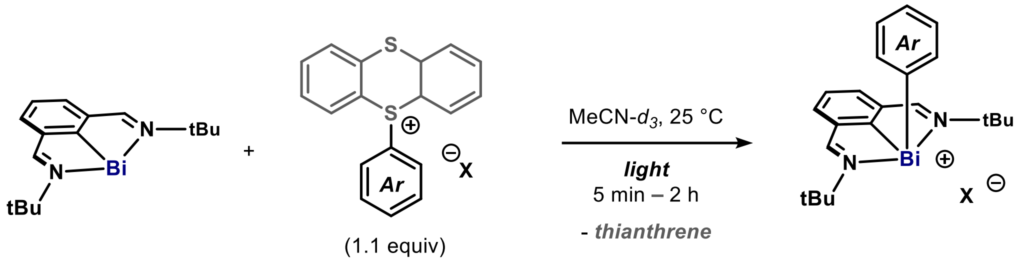

In an argon-filled glovebox, an oven-dried glass vial with a magnetic stirring bar was charged with bismuthinidene **1** (1.0 equiv) and the corresponding aryl thianthrenium salt (1.1 equiv). Both solids were dissolved in anhydrous, degassed MeCN-d_3 (for characterization) or MeCN (for preparative scale), before the vial was closed (or the MeCN-d_3 solution transferred to an NMR tube for characterization), taken out of the glovebox and further sealed with parafilm. The resulting homogeneous solution was stirred under red-light irradiation (unless stated otherwise, 2x 660 nm LED PR160L lamps purchased from Kessil, see section 2 for details) until full conversion of the starting bismuth(I) complex (which happened after ca. 5–120 min, depending on the substrate). Full conversion of bismuth(I) was determined visually, by the disappearance of its dark green color to give light yellow/orange solutions. The yield of the oxidative addition was determined by NMR by repeating the reaction in the presence of 1 equiv of 1,3,5-trimethoxybenzene as internal standard. Unless otherwise stated, full conversion was observed for the starting bismuth(I) complex.

For preparative scale, the reaction was carried out in non-deuterated MeCN, in a 10-mL screw-cap culture tube. The tube was further sealed with parafilm, taken out of the glovebox, and stirred under red-light irradiation (unless stated otherwise, 2x 660 nm LED PR160L lamps at 100% intensity, purchased from Kessil, see section 2 for details). Full conversion of bismuth(I) was determined visually, by the disappearance of its dark green color to give light yellow/orange solutions. Then, the solvent was removed in vacuum, and the product was precipitated by adding diethyl ether and sonication. The resulting powder was filtered through a small frit, and washed 5 times with diethyl ether, to remove most of the thianthrene byproduct. Then, the resulting off-white solid was dried in high vacuum and, if necessary, submitted to crystallization for X-ray analysis.

General procedure C3 for the red-light promoted oxidative addition of aryl iodides

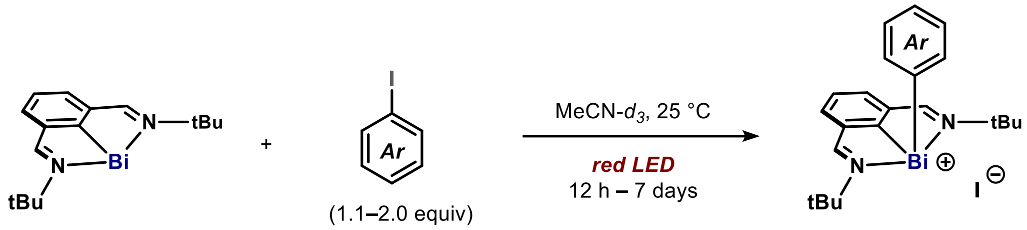

In an argon-filled glovebox, a glass vial with a magnetic stirring bar was charged with bismuthinidene **1** (1.0 equiv) and the corresponding aryl iodide (1.1–2.0 equiv). Both reagents were dissolved in anhydrous, degassed MeCN-d_3 (for characterization) and the solution was transferred into an NMR tube which was closed, taken out of the glovebox and further sealed with parafilm. The resulting homogeneous solution was left red-light irradiation (unless stated otherwise, 2x 660 nm LED PR160L lamps at 100% intensity, purchased from Kessil, see section 2 for details) until full conversion of the starting bismuth(I) complex (which happened after 12 h to 7 days, depending on the substrate and on the scale of the reaction). Full conversion of bismuth(I) was determined visually, by the disappearance of its dark green color to give light yellow/orange solutions. The yield of the oxidative addition was determined by NMR by repeating the reaction in the presence of 1 equiv of 1,3,5-trimethoxybenzene as internal standard. Unless otherwise stated, full conversion was observed for the starting bismuth(I) complex.

For preparative scale, the reaction was carried out in non-deuterated MeCN, in a 10-mL screw-cap culture tube. The tube was further sealed with parafilm, taken out of the glovebox, and stirred under red-light irradiation (unless stated otherwise, 2x 660 nm LED PR160L lamps at 100% intensity, purchased from Kessil, see section 2 for details). Full conversion of bismuth(I) was determined visually, by the disappearance of its dark green color to give light yellow/orange solutions. Then, the solvent was removed in vacuum, and the product was precipitated by adding diethyl ether and sonication. The resulting powder was filtered through a small frit, and washed 5 times with diethyl ether, to remove most of the unreacted excess aryl iodide. Then, the resulting yellow solid was dried in high vacuum and, if necessary, submitted to crystallization for X-ray analysis.

Note on scale and conversion: In contrast to the reactions described in procedures **C1** and **C2**, (which are very fast due to the highly favorable fragmentation rate)[9] this reaction is slower and directly dependent to the number of photons absorbed. These reactions follow a zero-order kinetic profile, and do not depend on the concentration of reagents (see Section 10). Thus, the use of a red-light source of the appropriate intensity, and controlling the scale of the reaction (which was kept between 0.03 and 0.20 mmol in this study) is required to get full conversion in reasonable timeframes. Furthermore, the oxidative addition adducts are sensitive to high-energy light irradiation (e.g.: blue LED).

For kinetic details on the influence of light color on selectivity and light intensity in reaction rate, see section **9**.

5.2. Characterization data of the oxidative addition adducts

From aryl diazonium salts

[(2,6-(*t*BuNCH)$_2$C$_6$H$_3$)Bi(4-fluorophenyl)(tetrafluoroborate)] (3a)

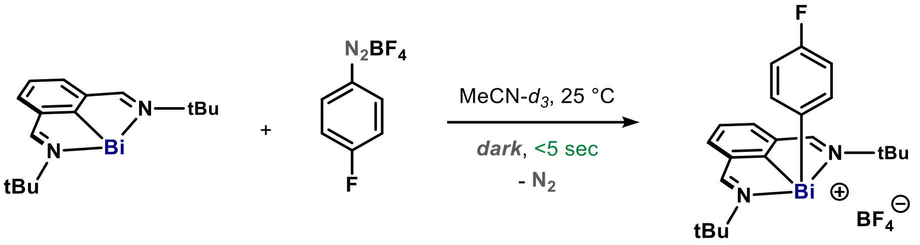

The title compound was obtained from bismuthinidene **1** (0.030 mmol, 1.0 equiv) and 4-fluorobenzene diazonium tetrafluoroborate (0.033 mmol, 1.1 equiv) in 0.6 mL of MeCN-*d*$_3$ (ca. 0.05 M) immediately after addition at room temperature (full conversion of bismuth(I), 92% NMR yield).

^{1}H NMR (600 MHz, CD$_3$CN) δ 9.71 (s, 2H), 8.33 (d, *J* = 7.6 Hz, 2H), 8.10 – 8.06 (m, 1H), 8.06 – 8.03 (m, 2H), 7.21 – 7.17 (m, 2H), 1.29 (s, 18H).

^{13}C NMR (151 MHz, CD$_3$CN) δ 187.60, 174.05, 168.61, 164.09 (d, *J* = 247.6 Hz), 149.40, 141.49 (d, *J* = 7.7 Hz), 137.59, 131.81, 120.21 (d, *J* = 20.6 Hz), 62.35, 30.96.

^{19}F NMR (565 MHz, CD$_3$CN) δ -111.17 (tt, *J* = 9.4, 5.9 Hz), -151.6.

HRMS (ESI Positive): calculated for $C_{22}H_{27}BiFN_2$ [M-BF$_4$]$^+$: 547.1956; found: 547.1956.

Full assignment for a representative oxidative addition adduct (see NMR section for details)

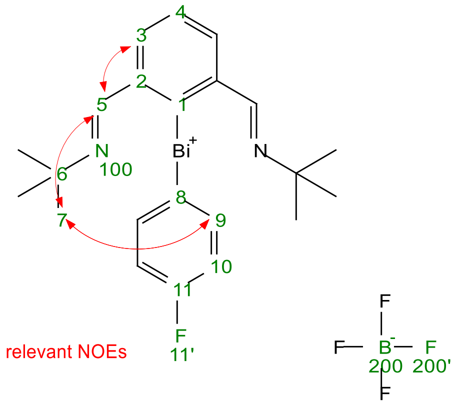

Atom	J	δ (ppm)	HSQC	COSY	HMBC	NOESY
1 C		187.596			3, 5	
2 C		149.395			4, 5	
3 C		137.587	3		3, 5	
H	7.60(4)	8.326	3	4	1, 3, 5	5
4 C		131.812	4			
H	7.60(3)	8.078	4	3	2	
5 C		168.609	5		3	
H		9.707	5		1, 2, 3, 6, 100	3, 7
6 C		62.346			5, 7	
7 C		30.964	7		7	
H3		1.294	7		6, 7, 100	5, 9
8 C		174.051			10	
9 C	5.90(11')	141.490	9		9	
H		8.050	9	10	9, 11	7
10 C	9.40(11')	120.212	10		10	
H		7.189	10	9	8, 10, 11	
11 C		164.091			9, 10	
11' F	9.40(10), 5.90(9)	-111.170				
100 N		-70.829			5, 7	
200 B		-1.171				
200' F		-151.546				

Both sites of the cation (Ar-F and Ligand) can be correlated in the 2D NOESY.

[(2,6-(CyNCH)$_2$C$_6$H$_3$)Bi(4-fluorophenyl)(tetrafluoroborate)] (**3b**)

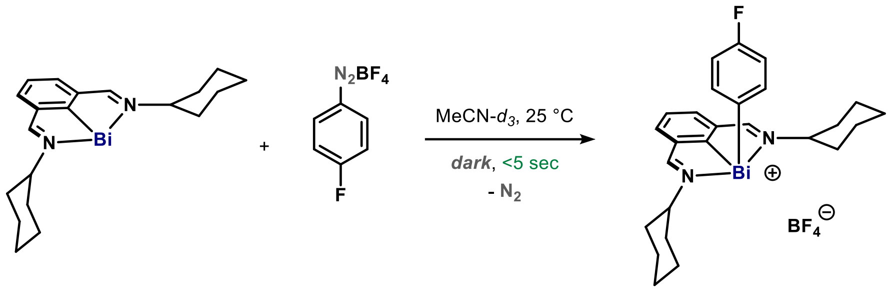

The title compound was obtained following General Procedure **C1** from bismuthinidene **S5** (16 mg, 0.030 mmol, 1.0 equiv) and 4-fluorobenzene diazonium tetrafluoroborate (6.9 mg, 0.033 mmol, 1.1 equiv) in 0.6 mL of MeCN-*d*$_3$ (0.05 M) immediately after addition (91% NMR yield).

^{1}H NMR (600 MHz, CD$_3$CN) δ 9.69 (d, *J* = 1.1 Hz, 2H), 8.28 (d, *J* = 7.6 Hz, 2H), 8.03 (dd, *J* = 7.8, 7.4 Hz, 1H), 8.05 – 7.99 (m, 2H), 7.21 – 7.17 (m, 2H), 3.52 (ttd, *J* = 11.1, 4.1, 1.1 Hz, 2H), 1.99 – 1.91 (m, 2H), 1.86 – 1.80 (m, 2H), 1.67 (m, 4H), 1.52 – 1.43 (m, 4H), 1.42 – 1.35 (m, 2H), 1.34 – 1.25 (m, 2H), 1.19 (qt, *J* = 12.8, 3.8 Hz, 2H), 1.11 (tdd, *J* = 12.7, 11.3, 3.7 Hz, 2H).

^{13}C NMR (151 MHz, CD$_3$CN) δ 186.43, 173.25, 170.87, 164.07 (d, *J* = 247.4 Hz), 147.89, 141.40 (d, *J* = 7.7 Hz), 137.26, 131.75, 120.11 (d, *J* = 20.6 Hz), 68.46, 36.87, 35.86, 25.74, 25.47, 25.36.

^{19}F NMR (565 MHz, CD$_3$CN) δ -111.26 (tt, *J* = 9.4, 5.9 Hz), -151.6.

HRMS (ESI Positive): calculated for $C_{26}H_{31}BiFN_2$ [M-BF$_4$]$^+$: 599.22698; found: 599.22687.

[(Phebox)Bi(4-methoxyphenyl)(tetrafluoroborate)] (**3c**)

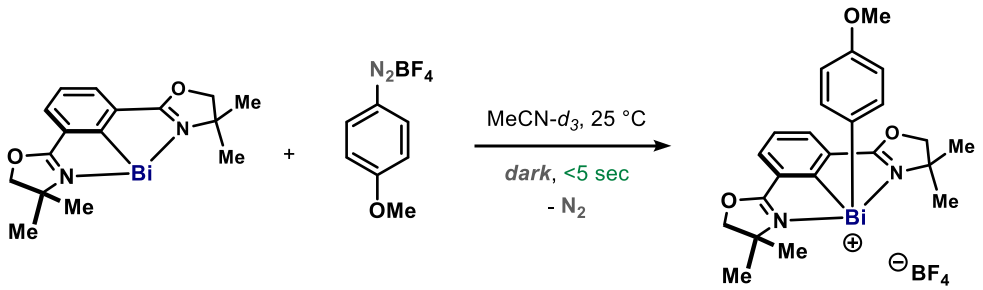

The title compound was obtained following General Procedure **C1** from (Phebox)Bi(I),[3] (14 mg, 0.030 mmol, 1 equiv) and 4-methoxybenzene diazonium tetrafluoroborate (6.8 mg, 0.033 mmol, 1.1 equiv) in 0.6 mL of MeCN-*d*$_3$ (0.05 M) immediately after addition (89% NMR yield).

^{1}H NMR (500 MHz, CD$_3$CN) δ 8.26 (d, *J* = 7.7 Hz, 2H), 8.04 – 8.00 (m, 2H), 7.91 (dd, *J* = 7.9, 7.5 Hz, 1H), 7.07 – 7.02 (m, 2H), 4.50 (d, *J* = 8.8 Hz, 2H), 4.46 (d, *J* = 8.8 Hz, 2H), 3.73 (s, 3H), 1.48 (s, 6H), 1.08 (s, 6H).

^{13}C NMR (151 MHz, CD$_3$CN) δ 187.67, 178.77, 172.00, 161.35, 140.59, 134.98, 133.39, 131.37, 118.38, 83.60, 68.00, 55.84, 28.57, 28.10.

^{19}F NMR (470 MHz, CD$_3$CN) δ -151.6.

HRMS (ESI Positive): calculated for $C_{23}H_{26}BiN_2O_3$ [M-BF$_4$]$^+$: 587.17419; found: 587.17454.

[(2,6-(*t*BuNCH)$_2$C$_6$H$_3$)Bi(4-methoxyphenyl)(tetrafluoroborate)] (**3d**)

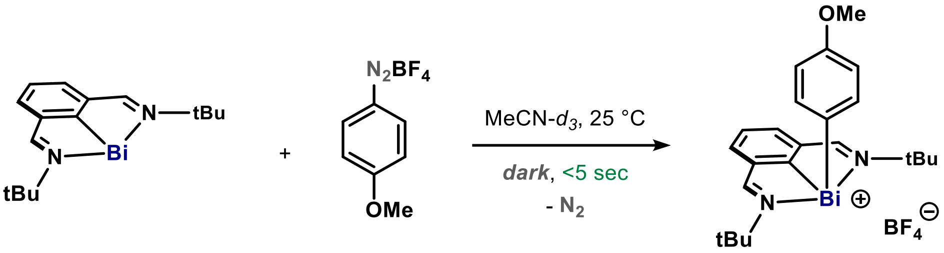

The title compound was obtained following General Procedure **C1** from bismuthinidene **1** (23 mg, 0.050 mmol, 1.0 equiv) and 4-methoxybenzene diazonium tetrafluoroborate (11 mg, 0.055 mmol, 1.1 equiv) in 1.2 mL of MeCN-d_3 (0.05 M) immediately after addition (93% NMR yield). Alternatively, the same reaction was performed at 0.20 mmol-scale, giving the title compound as a pale orange solid after removal of the solvent in vacuum (87% isolated yield).

^{1}H NMR (600 MHz, CD$_3$CN) δ 9.67 (s, 2H), 8.30 (d, *J* = 7.6 Hz, 2H), 8.06 (t, *J* = 7.6 Hz, 1H), 7.98 – 7.87 (m, 2H), 7.04 – 6.96 (m, 2H), 3.71 (s, 3H), 1.29 (s, 18H).

^{13}C NMR (151 MHz, CD$_3$CN) δ 187.10, 169.57, 168.34, 161.50, 149.32, 140.67, 137.46, 131.66, 118.70, 62.25, 55.85, 30.98.

^{19}F NMR (565 MHz, CD$_3$CN) δ -151.6.

HRMS (ESI Positive): calculated for $C_{23}H_{30}BiN_2O$ [M-BF$_4$]$^+$: 559.21566; found: 559.21517.

[(2,6-(*t*BuNCH)$_2$C$_6$H$_3$)Bi(4-benzonitrile)(tetrafluoroborate)] (**3e**)

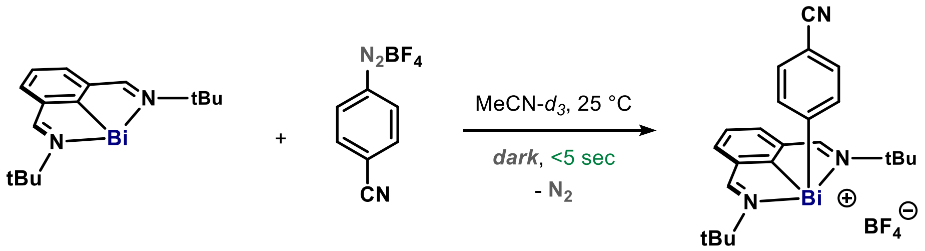

The title compound was obtained following General Procedure **C1** from bismuthinidene **1** (14 mg, 0.030 mmol, 1.0 equiv) and 4-benzonitrile diazonium tetrafluoroborate (6.7 mg, 0.033 mmol, 1.1 equiv) in 0.6 mL of MeCN-d_3 (0.05 M) immediately after addition (86% NMR yield).

^{1}H NMR (600 MHz, CD$_3$CN) δ 9.76 (s, 2H), 8.35 (d, *J* = 7.6 Hz, 2H), 8.24 – 8.20 (m, 2H), 8.10 (dd, *J* = 7.8, 7.4 Hz, 1H), 7.79 (d, *J* = 8.3 Hz, 2H), 1.29 (s, 18H).

^{13}C NMR (151 MHz, CD$_3$CN) δ 188.33, 184.49, 168.98, 149.53, 139.51, 137.77, 135.91, 131.98, 119.48, 113.60, 62.45, 30.97.

^{19}F NMR (565 MHz, CD$_3$CN) δ -151.5.

HRMS (ESI Positive): calculated for $C_{23}H_{27}BiN_3$ [M-BF$_4$]$^+$: 554.20035; found: 554.20074.

[(2,6-(*t*BuNCH)$_2$C$_6$H$_3$)Bi(4-bromophenyl)(tetrafluoroborate)] (**3f**)

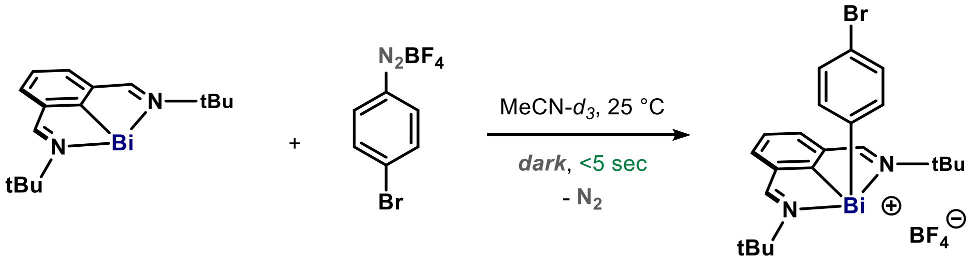

The title compound was obtained following General Procedure **C1** from bismuthinidene **1** (14 mg, 0.030 mmol, 1.0 equiv) and 4-bromobenzene diazonium tetrafluoroborate (8.5 mg, 0.033 mmol, 1.1 equiv) in 0.6 mL of MeCN-d_3 (0.05 M) immediately after addition (94% NMR yield).

^{1}H NMR ^{1}H NMR (600 MHz, CD$_3$CN) δ 9.72 (s, 2H), 8.33 (d, *J* = 7.6 Hz, 2H), 8.08 (dd, *J* = 7.8, 7.4 Hz, 1H), 7.99 – 7.95 (m, 2H), 7.65 – 7.62 (m, 2H), 1.30 (s, 18H).

^{13}C NMR (151 MHz, CD$_3$CN) δ 187.78, 177.53, 168.71, 149.44, 140.79, 137.62, 135.86, 131.85, 124.48, 62.39, 30.98.

^{19}F NMR (565 MHz, CD$_3$CN) δ -151.6.

HRMS (ESI Positive): calculated for C$_{22}$H$_{27}$Bi^{79}BrN$_2$ [M-BF$_4$]$^+$: 607.11561; found: 607.11553.

[(2,6-(*t*BuNCH)$_2$C$_6$H$_3$)Bi(3-quinolinyl)(tetrafluoroborate)] (**3g**)

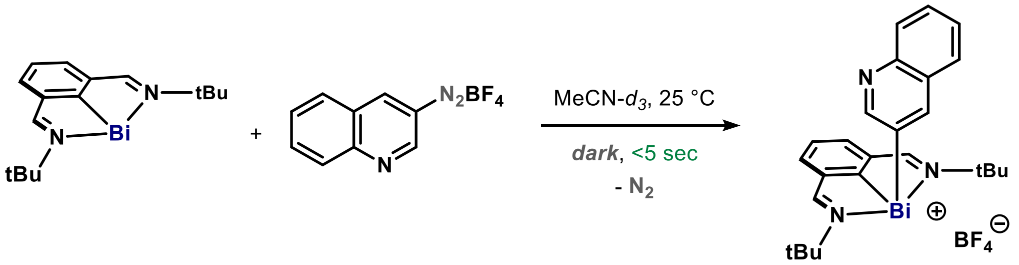

The title compound was obtained following General Procedure **C1** from bismuthinidene **1** (14 mg, 0.030 mmol, 1.0 equiv) and quinolone-3-diazonium tetrafluoroborate (7.7 mg, 0.033 mmol, 1.1 equiv) in 0.6 mL of MeCN-d_3 (0.05 M) immediately after addition (93% NMR yield).

^{1}H NMR (600 MHz, CD$_3$CN) δ 9.75 (s, 2H), 9.14 (dd, *J* = 1.8, 0.8 Hz, 1H), 8.88 (d, *J* = 1.8 Hz, 1H), 8.38 (d, *J* = 7.6 Hz, 2H), 8.12 (dd, *J* = 7.8, 7.4 Hz, 1H), 7.94 – 7.92 (m, 1H), 7.79 – 7.77 (m, 1H), 7.72 (ddd, *J* = 8.4, 6.8, 1.5 Hz, 1H), 7.60 (ddd, *J* = 8.2, 6.9, 1.2 Hz, 1H), 1.29 (s, 18H).

^{13}C NMR (151 MHz, CD$_3$CN) δ 186.50, 170.54, 168.99, 158.06, 149.66, 148.73, 146.83, 137.78, 131.94, 131.76, 131.53, 130.49, 129.12, 128.39, 62.47, 30.99.

^{19}F NMR (565 MHz, CD$_3$CN) δ -151.5.

HRMS (ESI Positive): calculated for C$_{25}$H$_{29}$BiN$_3$ [M-BF$_4$]$^+$: 580.21600; found: 580.21601.

[(2,6-(*t*BuNCH)$_2$C$_6$H$_3$)Bi(8-quinolinyl)(tetrafluoroborate)] (**3h**)

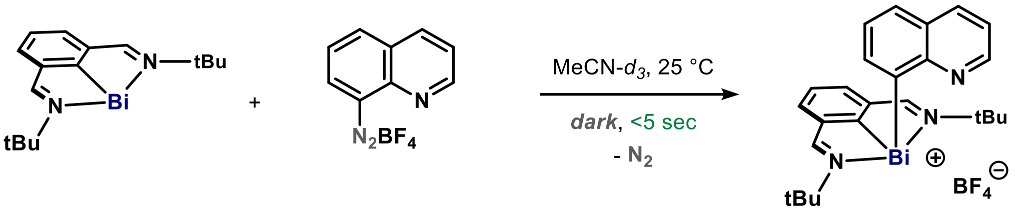

The title compound was obtained following General Procedure **C1** from bismuthinidene **1** (14 mg, 0.030 mmol, 1.0 equiv) and quinolone-8-diazonium tetrafluoroborate (8.1 mg, 0.033 mmol, 1.1 equiv) in 0.6 mL of MeCN-d_3 (0.05 M) immediately after addition (74% NMR yield, with ca. 25% of other minor products, being acetonitrile activation product **A** the major one, see section 7 for details).

^{1}H NMR (600 MHz, CD$_3$CN) δ 9.71 (s, 2H), 9.19 (dd, J = 4.3, 1.7 Hz, 1H), 8.53 (ddd, J = 8.3, 1.7, 0.3 Hz, 1H), 8.32 (d, J = 7.6 Hz, 2H), 8.22 (dd, J = 7.0, 1.3 Hz, 1H), 8.11 – 8.07 (m, 1H), 8.11 – 8.06 (m, 1H), 7.63 (dd, J = 8.3, 4.3 Hz, 1H), 7.57 (dd, J = 8.0, 7.0 Hz, 1H), 1.13 (s, 18H).

^{13}C NMR (151 MHz, CD$_3$CN) δ 189.04, 179.99, 168.60, 151.95, 151.43, 150.10, 139.02, 138.76, 137.27, 132.05, 131.46, 130.84, 130.61, 123.25, 61.91, 30.67.

^{19}F NMR (565 MHz, CD$_3$CN) δ -151.7.

HRMS (ESI Positive): calculated for $C_{25}H_{29}BiN_3$ [M-BF$_4$]$^+$: 580.21600; found: 580.21596.

[(2,6-(*t*BuNCH)$_2$C$_6$H$_3$)Bi(3-(2-methoxycarbonyl)thiophenyl)(tetrafluoroborate)] (**3i**)

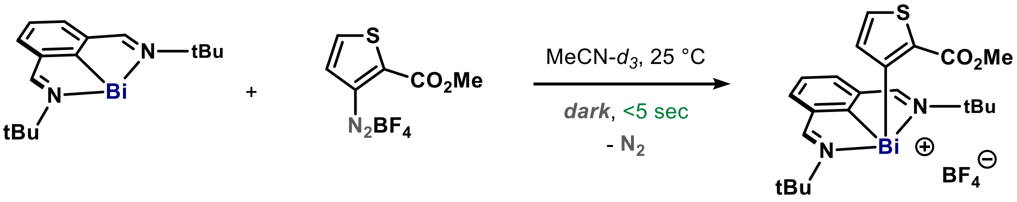

The title compound was obtained following General Procedure **C1** from bismuthinidene **1** (14 mg, 0.030 mmol, 1.0 equiv) and 2-(methoxycarbonyl)thiophene-3-diazonium tetrafluoroborate (8.1 mg, 0.033 mmol, 1.1 equiv) in 0.6 mL of MeCN-d_3 (0.05 M) immediately after addition (85% NMR yield together with 10% of a species that was tentatively assigned as the (5-thiophenyl)-bismuth(III) isomer).

^{1}H NMR (600 MHz, CD$_2$Cl$_2$) δ 9.68 (s, 2H), 8.34 (d, J = 7.6 Hz, 2H), 8.12 – 8.06 (m, 1H), 7.63 (d, J = 4.9 Hz, 1H), 6.48 (d, J = 4.9 Hz, 1H), 4.04 (s, 3H), 1.29 (s, 18H).

^{13}C NMR (151 MHz, CD$_2$Cl$_2$) δ 188.56, 179.18, 168.03, 165.42, 149.55, 141.45, 139.49, 138.73, 136.88, 131.50, 61.97, 53.50, 30.73.

^{19}F NMR (565 MHz, CD$_2$Cl$_2$) δ -151.9.

HRMS (ESI Positive): calculated for $C_{22}H_{28}BiN_2O_2S$ [M-BF$_4$]$^+$: 593.16700; found: 593.16736.

From aryl thianthrenium salts

[(2,6-(*t*BuNCH)$_2$C$_6$H$_3$)Bi(4-methoxyphenyl)(OTf)] (7a)

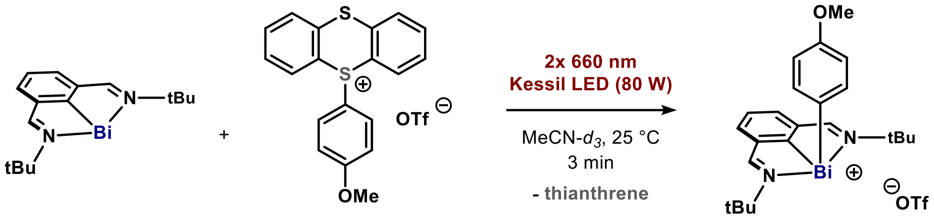

The title compound was obtained following General Procedure **C2** from bismuthinidene **1** (9.1 mg, 0.020 mmol, 1.0 equiv) and the corresponding aryl thianthrenium salt (11 mg, 0.022 mmol, 1.1 equiv) in 0.6 mL of MeCN-*d*$_3$ (0.05 M) after 3 min of red-LED irradiation (93% NMR yield).

^{1}H NMR (600 MHz, CD$_3$CN) δ 9.66 (s, 2H), 8.30 (d, *J* = 7.6 Hz, 2H), 8.10 – 8.02 (m, 1H), 7.95 – 7.91 (m, 2H), 7.01 – 6.99 (m, 2H), 3.71 (s, 3H), 1.29 (s, 18H).

^{13}C NMR (151 MHz, CD$_3$CN) δ 187.1, 169.6, 168.3, 161.5, 149.3, 140.7, 137.4, 131.6, 122.2 (q, *J* = 321.0 Hz), 118.7, 62.2, 55.8, 31.0.

^{19}F NMR (565 MHz, CD$_3$CN) δ -79.3.

HRMS (ESI Positive): calculated for $C_{23}H_{30}BiN_2O$ [M-OTf]$^+$: 559.21566; found: 559.21605.

Comparison between different 4-methoxyphenyl bismuthonium complexes 3c, 7a and 7b

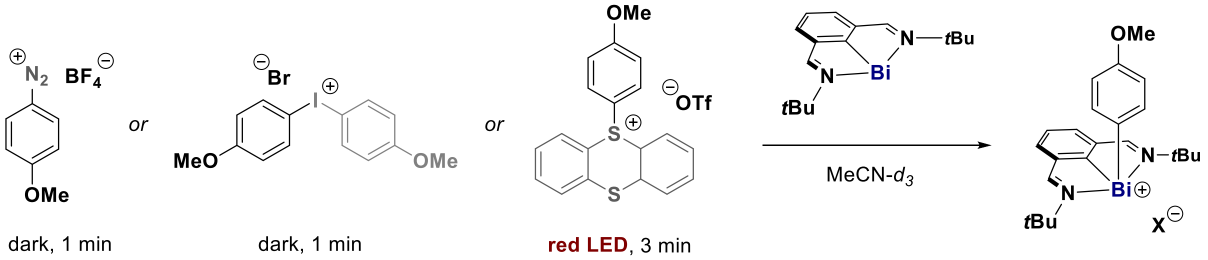

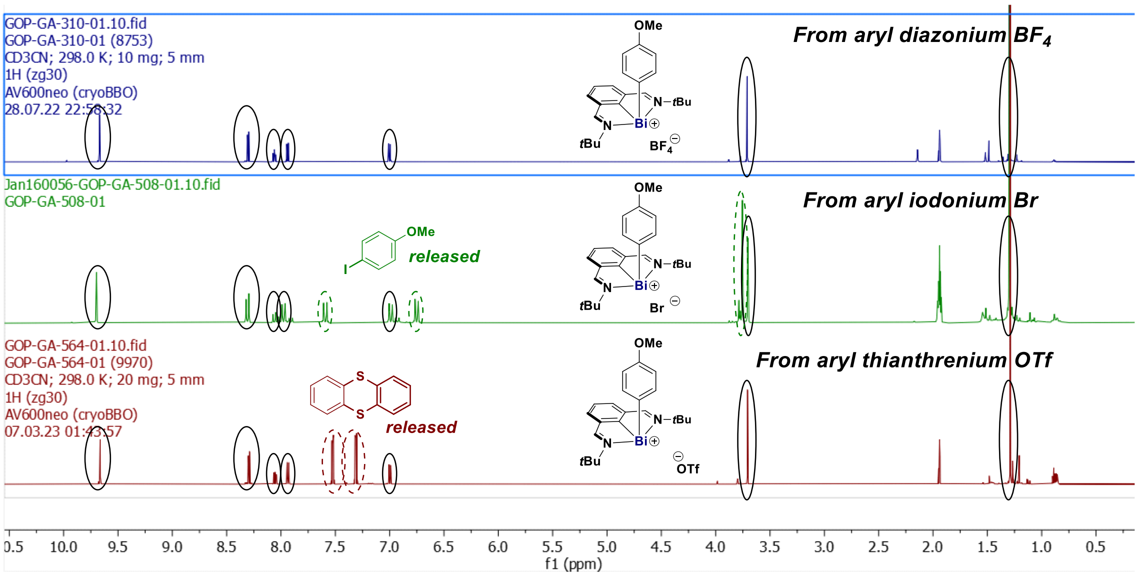

[(2,6-(*t*BuNCH)$_2$C$_6$H$_3$)Bi(4-(4-(2-(pyridin-2-yloxy)propoxy)phenoxy)phenyl)(tetrafluoroborate)] (7b)

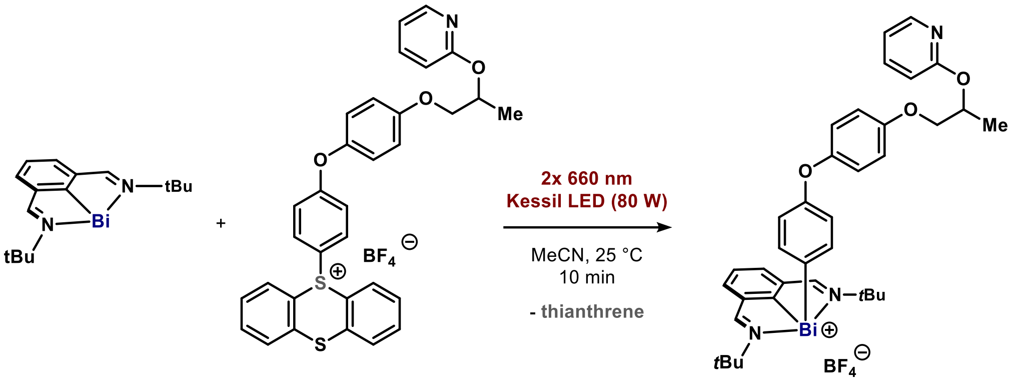

The title compound was obtained following General Procedure **C2** from bismuthinidene **1** (45 mg, 0.10 mmol, 1.0 equiv) and the corresponding aryl thianthrenium salt (62 mg, 0.10 mmol, 1.0 equiv) in 2 mL of MeCN (0.1 M) after 10 min of red-LED irradiation. The product was obtained as 56 mg of an off-white solid (65% isolated yield; 85% NMR yield) after washing with diethyl ether and drying in vacuum.

^{1}H NMR (600 MHz, CD$_3$CN) δ 9.68 (s, 2H), 8.30 (d, *J* = 7.6 Hz, 2H), 8.12 (ddd, *J* = 5.0, 2.0, 0.8 Hz, 1H), 8.08 – 8.01 (m, 1H), 7.99 – 7.92 (m, 2H), 7.63 (ddd, *J* = 8.3, 7.1, 2.0 Hz, 1H), 6.99 – 6.97 (m, 2H), 6.93 – 6.90 (m, 3H), 6.89 – 6.86 (m, 2H), 6.70 (dt, *J* = 8.3, 0.9 Hz, 1H), 5.58 – 5.50 (m, 1H), 4.15 (dd, *J* = 10.3, 6.0 Hz, 1H), 4.09 (dd, *J* = 10.3, 4.1 Hz, 1H), 1.39 (d, *J* = 6.4 Hz, 3H), 1.30 (s, 18H).

^{13}C NMR (151 MHz, CD$_3$CN) δ 187.28, 171.60, 168.42, 164.15, 160.50, 156.61, 150.26, 149.33, 147.85, 140.88, 140.13, 137.49, 131.69, 122.07, 121.76, 117.95, 116.83, 112.20, 71.95, 70.26, 62.29, 30.99, 16.97.

^{19}F NMR (470 MHz, CD$_3$CN) δ -151.7.

^{11}B NMR (160 MHz, CD$_3$CN) δ -1.21.

HRMS (ESI Positive): calculated for C$_{36}$H$_{41}$BiN$_3$O$_3$ [M-BF$_4$]$^+$: 772.29464; found: 772.29431.

[(2,6-(tBuNCH)$_2$C$_6$H$_3$)Bi(4-*N*-tosyl-*N*-methyl-aniline)(tetrafluoroborate)] (**7c**)

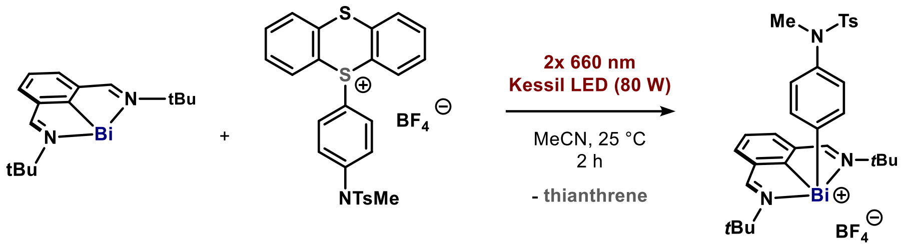

The title compound was obtained following General Procedure **C2** from bismuthinidene **1** (90 mg, 0.20 mmol, 1.0 equiv) and the corresponding aryl thianthrenium salt (112 mg, 0.20 mmol, 1.0 equiv) in 2 mL of MeCN (0.1 M) after 2 h of red-LED irradiation. The product was obtained as 120 mg of a microcrystalline colorless solid (75% isolated yield, 94% NMR yield) after washing with diethyl ether and drying in vacuum. Single crystals suitable for x-ray diffraction analysis were obtained by vapor diffusion of methyl *tert*-butyl ether into a solution of the product in acetonitrile, or diethyl ether into a solution of the product in 1,2-dichloroethane.

^{1}H NMR (600 MHz, CD$_2$Cl$_2$) δ 9.62 (s, 2H), 8.32 (d, J = 7.6 Hz, 2H), 8.13 – 8.05 (m, 1H), 7.95 (m, 2H), 7.25 – 7.21 (m, 4H), 7.20 – 7.17 (m, 2H), 3.08 (s, 3H), 2.39 (s, 3H), 1.34 (s, 18H).

^{13}C NMR (151 MHz, CD$_2$Cl$_2$) δ 185.83, 174.68, 167.37, 148.50, 144.64, 143.34, 138.65, 137.15, 133.23, 131.60, 129.98, 129.79, 127.89, 62.21, 37.82, 31.07, 21.67.

^{19}F NMR (565 MHz, CD$_2$Cl$_2$) δ -151.0.

^{11}B NMR (193 MHz, CD$_2$Cl$_2$) δ -1.14.

HRMS (ESI Positive): calculated for C$_{30}$H$_{37}$BiN$_3$O$_2$S [M-BF$_4$]$^+$: 712.24050; found: 712.24032.

Full assignment for a representative oxidative addition adduct (see NMR section for details)

NMR data supports the following species:

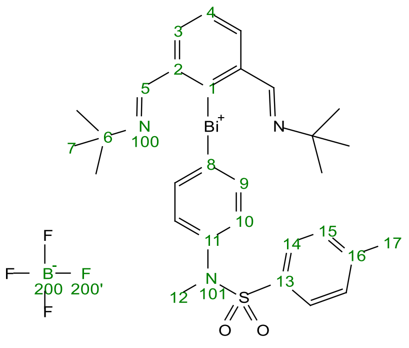

Atom	J	δ (ppm)	HSQC	COSY	HMBC	NOESY
1 C		185.827			3, 5	
2 C		148.497			4, 5	
3 C		137.146	3		3, 5	
H	7.6(4)	8.325	3	4	1, 3, 5	5
4 C		131.600	4			
H	7.6(3)	8.090	4	3	2	
5 C		167.366	5		3	
H		9.622	5		1, 2, 3, 6, 100	3, 7
6 C		62.207			5, 7	
7 C		31.073	7		7	
H3		1.343	7		6, 7, 100	5, 9
8 C		174.680			10	
9 C		138.651	9		9	
H		7.951	9	10	9, 11	7, 10
10 C		129.977	10		10	
H		7.232	10	9	8, 10, 101	9, 12

Atom	J	δ (ppm)	HSQC	COSY	HMBC	NOESY
11 C		143.342			9, 12	
12 C		37.818	12			
H3		3.076	12		11	10
13 C		133.229			15	
14 C		127.891	14		14	
H		7.232	14	15	14, 16	
15 C		129.788	15		15, 17	
H		7.179	15	14	13, 15, 17	17
16 C		144.642			14, 17	
17 C		21.673	17		15	
H3		2.393	17		15, 16	15
100 N		-69.738			5, 7	
101 N		-272.700			10	
200 B		-1.136				
200' F		-151.089, -151.035				

From aryl iodides

[(2,6-(*t*BuNCH)$_2$C$_6$H$_3$)Bi(4-cyanophenyl)(iodide)] (**12a**)

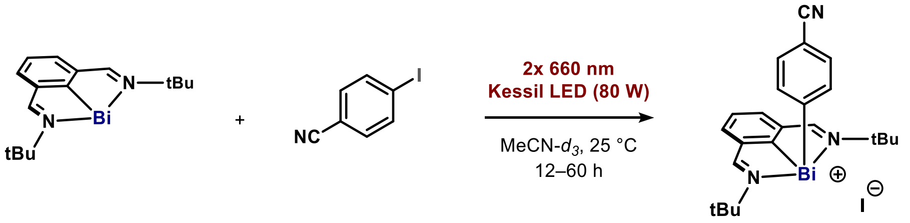

For characterization, the title compound was obtained following General Procedure **C3** from bismuthinidene **1** (9.1 mg, 0.020 mmol, 1.0 equiv) and 4-iodobenzonitrile (5.1 mg, 0.022 mmol, 1.1 equiv) in 0.6 mL of MeCN-d_3 (0.033 M) after 12 h of red-LED irradiation (93% NMR yield). Alternatively, the title compound was also obtained from bismuthinidene **1** (68 mg, 0.15 mmol, 1.0 equiv) and 4-iodobenzonitrile (68 mg, 0.30 mmol, 2.0 equiv) in 2 mL of MeCN (0.08 M) after 60 h of red-LED irradiation and washing with diethyl ether, as a yellow powder (85 mg, 83% yield). Crystals suitable for x-ray diffraction were obtained by vapor diffusion of MTBE into a 1,2- dichloroethane solution.

^{1}H NMR (600 MHz, CD$_3$CN) δ 9.76 (s, 2H), 8.34 (d, *J* = 7.6 Hz, 2H), 8.28 – 8.23 (m, 2H), 8.09 (dd, *J* = 7.8, 7.4 Hz, 1H), 7.81 – 7.75 (m, 2H), 1.30 (s, 18H).

^{13}C NMR (151 MHz, CD$_3$CN) δ 188.98, 185.53, 168.91, 149.51, 139.62, 137.70, 135.81, 131.82, 119.51, 113.44, 62.43, 31.01.

HRMS (ESI Positive): calculated for C$_{23}$H$_{27}$BiN$_3$ [M-I]$^+$: 554.20035; found: 554.20065.

Full assignment for a representative oxidative addition adduct (see NMR section for details)

NMR data supports the following structure:

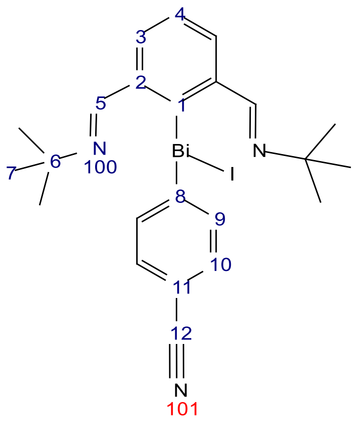

Atom	J	δ (ppm)	HSQC	COSY	HMBC	NOESY
1 C		188.974			3, 5	
2 C		149.511			4, 5	
3 C		137.696	3		3, 5	
H	7.6(4)	8.345	3	4	1, 3, 5	5
4 C		131.824	4			
H	7.6(3)	8.085	4	3	2	
5 C		168.911	5		3	
H		9.762	5		1, 2, 3, 6, 100	3, 7
6 C		62.429			5, 7	
7 C		31.014	7		7	
H3		1.301	7		6, 7, 100	5, 9
8 C		185.530			10	
9 C		139.621	9		9	
H		8.250	9	10	9, 11	7
10 C		135.808	10		10	
H		7.781	10	9	8, 10, 12	
11 C		113.442			9	
12 C		119.506			10	
100 N		-70.800			5, 7	
101 N						

[(2,6-(*t*BuNCH)$_2$C$_6$H$_3$)Bi(1-naphthyl)(iodide)] (**12b**)

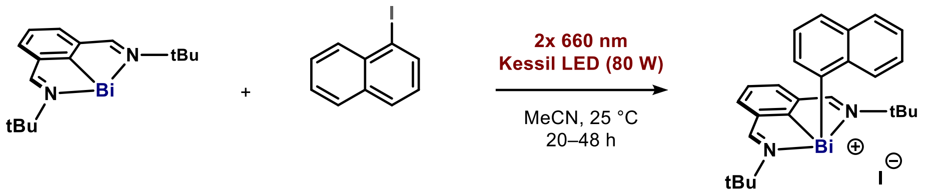

For characterization, the title compound was obtained following General Procedure **C3** from bismuthinidene **1** (9.1 mg, 0.020 mmol, 1.0 equiv) and the 1-iodonaphthalene (9.2 mg, 0.040 mmol, 2.0 equiv) in 0.6 mL of MeCN-*d*$_3$ (0.033 M) after 20 h of red-LED irradiation (93% NMR yield with 80 W 660 nm 2x Kessil lamps, but only 28% with ca. 20 W red LED strip). Alternatively, the title compound was also obtained from bismuthinidene **1** (45 mg, 0.10 mmol, 1.0 equiv) and 1-iodonaphthalene (31 mg, 0.12 mmol, 1.2 equiv) in 2 mL of MeCN (0.05 M) after 48 h of red-LED irradiation and washing with diethyl ether, as a yellow powder (48 mg, 68% yield).

^{1}H NMR (600 MHz, CD$_3$CN) δ 9.71 (s, 2H), 8.57 (d, *J* = 8.3 Hz, 1H), 8.36 (d, *J* = 7.6 Hz, 2H), 8.14 (m, 1H), 8.12 (dd, *J* = 7.8, 7.4 Hz, 1H), 8.10 – 8.07 (m, 1H), 8.05 – 8.00 (m, 1H), 7.76 (ddd, *J* = 8.3, 6.9, 1.3 Hz, 1H), 7.65 – 7.59 (m, 1H), 7.41 (dd, *J* = 8.1, 7.0 Hz, 1H), 1.15 (s, 18H).

^{13}C NMR (151 MHz, CD$_3$CN) δ 186.57, 180.34, 168.12, 149.34, 139.28, 138.21, 137.64, 136.55, 131.94, 131.25, 131.10, 131.05, 130.77, 128.34, 127.51, 61.97, 30.87.

HRMS (ESI Positive): calculated for C$_{26}$H$_{30}$BiN$_2$ [M-I]$^+$: 579.22075; found: 579.22076.

[(2,6-(*t*BuNCH)$_2$C$_6$H$_3$)Bi(4-trifluoromethylphenyl)(iodide)] (**12c**)

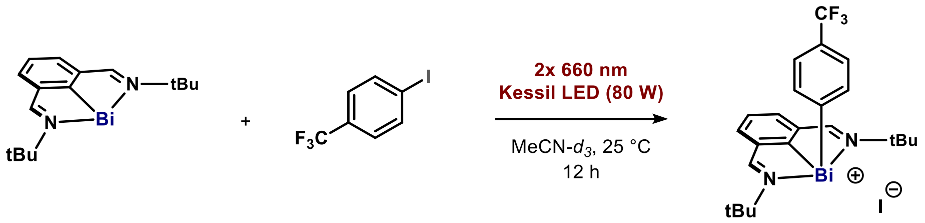

The title compound was obtained following General Procedure **C3** from bismuthinidene **1** (9.1 mg, 0.020 mmol, 1.0 equiv) and 4-iodotrifluorotoluene (6.5 mg, 0.024 mmol, 1.1 equiv) in 0.6 mL of MeCN-*d*$_3$ (0.033 M) after 12 h of red-LED irradiation (97% NMR yield).

^{1}H NMR (600 MHz, CD$_2$Cl$_2$) δ 9.69 (s, 2H), 8.31 (d, *J* = 7.6 Hz, 2H), 8.33 – 8.28 (m, 2H), 8.05 (t, *J* = 7.6 Hz, 1H), 7.69 – 7.64 (m, 2H), 1.38 (s, 18H).

^{13}C NMR (151 MHz, CD$_2$Cl$_2$) δ 190.0, 186.5, 167.3, 148.5, 139.1, 136.8, 131.0 (q, *J* = 32.4 Hz), 130.9, 128.6 (q, *J* = 3.7 Hz), 124.6 (q, *J* = 272.3 Hz), 62.1, 31.3.

^{19}F NMR (565 MHz, CD$_2$Cl$_2$) δ -63.33.

HRMS (ESI Positive): calculated for C$_{23}$H$_{27}$BiF$_3$N$_2$ [M-I]$^+$: 597.19248; found: 597.19303.

[(2,6-(*t*BuNCH)$_2$C$_6$H$_3$)Bi(4-chlorophenyl)(iodide)] (**12d**)

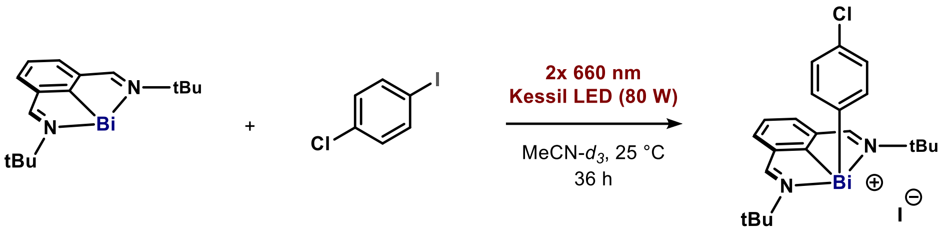

The title compound was obtained following General Procedure **C3** from bismuthinidene **1** (9.1 mg, 0.020 mmol, 1.0 equiv) and 4-chloroiodobenzene (5.3 mg, 0.022 mmol, 1.1 equiv) in 0.6 mL of MeCN-d_3 (0.033 M) after 36 h of red-LED irradiation (96% NMR yield).

^{1}H NMR (600 MHz, CD$_3$CN) δ 9.74 (s, 2H), 8.33 (d, *J* = 7.6 Hz, 2H), 8.10 – 8.05 (m, 2H), 8.07 (dd, *J* = 7.8, 7.4 Hz, 1H), 7.48 – 7.44 (m, 2H), 1.31 (s, 18H).

^{13}C NMR (151 MHz, CD$_3$CN) δ 188.72, 178.26, 168.61, 149.43, 140.83, 137.53, 135.84, 132.84, 131.63, 62.35, 31.03.

HRMS (ESI Positive): calculated for C$_{22}$H$_{27}$BiClN$_2$ [M-I]$^+$: 563.16613; found: 563.16625.

[(2,6-(*t*BuNCH)$_2$C$_6$H$_3$)Bi(4-bromophenyl)(iodide)] (**12e**)

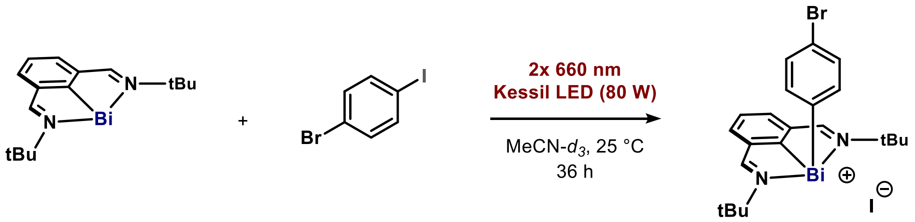

The title compound was obtained following General Procedure **C3** from bismuthinidene **1** (9.1 mg, 0.020 mmol, 1.0 equiv) and 4-bromoiodobenzene (6.2 mg, 0.022 mmol, 1.1 equiv) in 0.6 mL of MeCN-d_3 (0.033 M) after 36 h of red-LED irradiation (95% NMR yield).

^{1}H NMR (600 MHz, CD$_3$CN) δ 9.74 (s, 2H), 8.33 (d, *J* = 7.6 Hz, 2H), 8.07 (dd, *J* = 7.7, 7.4 Hz, 1H), 8.03 – 7.99 (m, 2H), 7.64 – 7.60 (m, 2H), 1.31 (s, 18H).

^{13}C NMR (151 MHz, CD$_3$CN) δ 188.71, 178.71, 168.62, 149.43, 140.97, 137.53, 135.73, 131.64, 124.29, 62.36, 31.03.

HRMS (ESI Positive): calculated for C$_{22}$H$_{27}$Bi^{79}BrN$_2$ [M-I]$^+$: 607.11561; found: 607.11556.

[(2,6-(*t*BuNCH)$_2$C$_6$H$_3$)Bi(4-fluorophenyl)(iodide)] (12f)

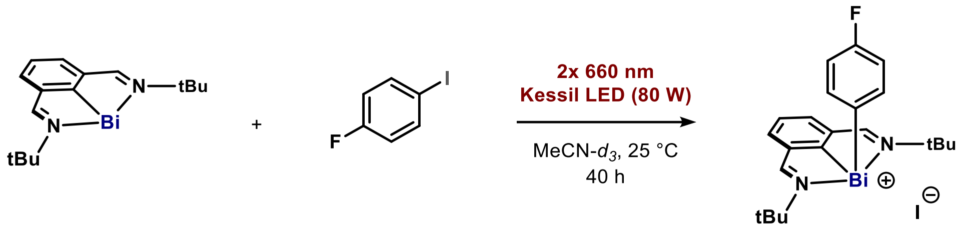

The title compound was obtained following General Procedure **C3** from bismuthinidene **1** (9.1 mg, 0.020 mmol, 1 equiv) and 4-fluoroiodobenzene (4.9 mg, 0.022 mmol, 1.1 equiv) in 0.6 mL of MeCN-*d*$_3$ (0.033 M) after 40 h of red-LED irradiation (95% NMR yield).

^{1}**H NMR** (600 MHz, CD$_3$CN) δ 9.73 (s, 2H), 8.33 (d, *J* = 7.6 Hz, 2H), 8.13 – 8.06 (m, 2H), 8.07 (dd, *J* = 7.7, 7.5 Hz, 1H), 7.20 – 7.15 (m, 2H), 1.31 (s, 18H).

^{13}**C NMR** (151 MHz, CD$_3$CN) δ 188.5, 175.1, 168.5, 164.0 (d, *J* = 247.4 Hz), 149.4, 141.6 (d, *J* = 7.6 Hz), 137.5, 131.6, 120.1 (d, *J* = 20.5 Hz), 62.3, 31.0.

^{19}**F NMR** (470 MHz, CD$_3$CN) δ -111.42 (tt, *J* = 9.4, 6.0 Hz). ^{19}**F{^{1}H} NMR** (470 MHz, CD$_3$CN) δ -111.42.

HRMS (ESI Positive): calculated for C$_{22}$H$_{27}$BiFN$_2$ [M-I]$^{+}$: 547.19575; found: 547.19568.

[(2,6-(*t*BuNCH)$_2$C$_6$H$_3$)Bi(4-methoxyphenyl)(iodide)] (12g)

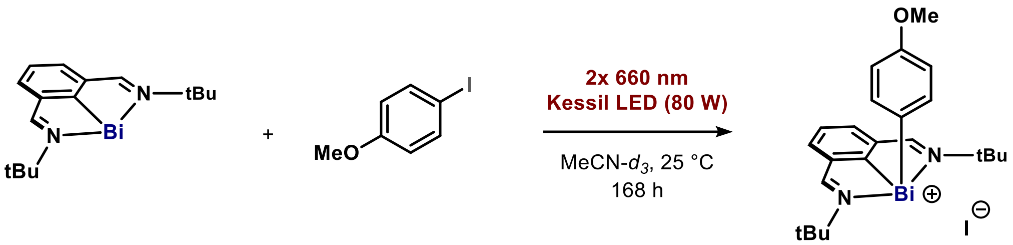

The title compound was obtained following General Procedure **C3** from bismuthinidene **1** (9.1 mg, 0.020 mmol, 1.0 equiv) and 4-methoxyiodobenzene (7.2 mg, 0.030 mmol, 1.5 equiv) in 0.6 mL of MeCN-*d*$_3$ (0.033 M) after 168 h of red-LED irradiation (95% NMR yield).

^{1}**H NMR** (600 MHz, CD$_3$CN) δ 9.70 (s, 2H), 8.31 (d, *J* = 7.6 Hz, 2H), 8.05 (dd, *J* = 7.7, 7.4 Hz, 1H), 7.99 – 7.96 (m, 2H), 7.01 – 6.98 (m, 2H), 3.71 (s, 3H), 1.30 (s, 18H).

^{13}**C NMR** (151 MHz, CD$_3$CN) δ 187.74, 170.27, 168.28, 161.39, 149.31, 140.77, 137.40, 131.51, 118.61, 62.22, 55.82, 31.01.

HRMS (ESI Positive): calculated for C$_{23}$H$_{30}$BiN$_2$O [M-I]$^{+}$: 559.21566; found: 559.21549.

Stability and decomposition of aryl bismuthonium complexes

Electron rich aryl bismuthonium complexes such as **12d** were found to be significantly stable and inert to be handled in solution under air, with wet solvents, either in the dark or under ambient light.

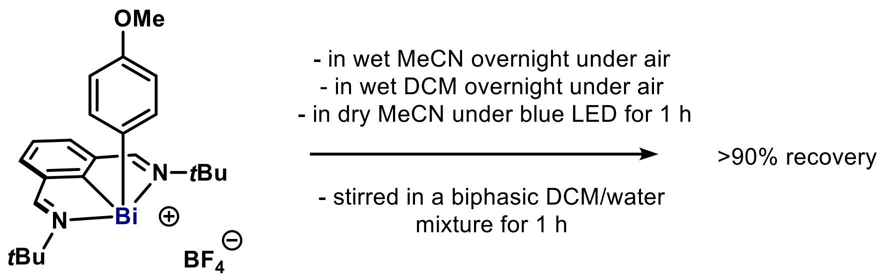

While attempting to crystallize a more electron poor complex under air, complexes **SI6** and **SI7** eventually crystallized instead. Presumably, **SI7** (which appears as a bridge dimer) could arise by a sequence involving the hydrolysis/oxidation of one of the imine arms under air, giving the corresponding carboxylate, whereas the released iodide anion ends up forming bismuth(III) diiodide **SI6**.

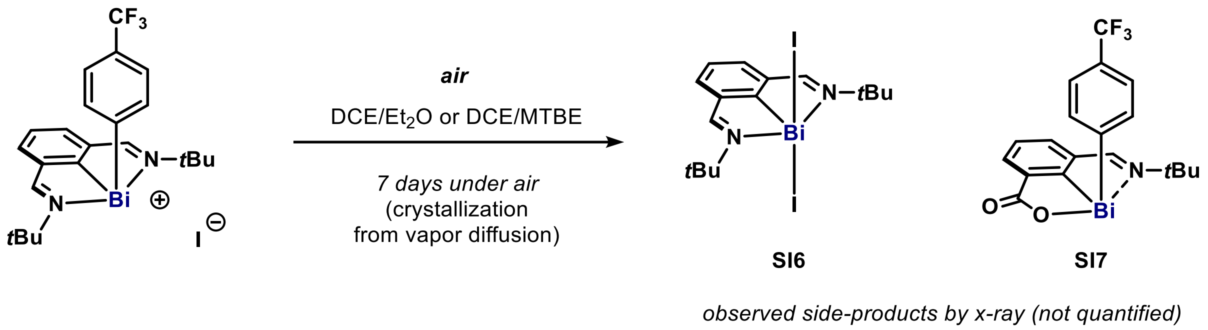

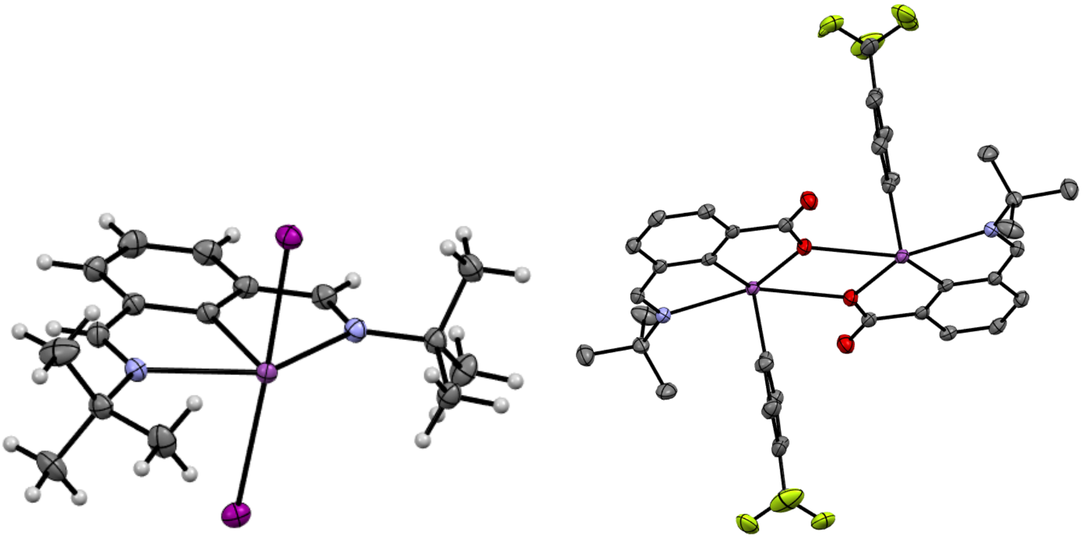

Figure S6. Single-crystal X-ray structures of **S16** (left) and **S17** (right).

5.3. Solid-state structure of aryl bismuth(III) complexes

Both of the oxidative addition bismuth(III) complexes analyzed by single-crystal x-ray diffraction were found to be cationic in the solid state, with both the BF_4^- and I^- staying >3 Å away from the bismuth center.

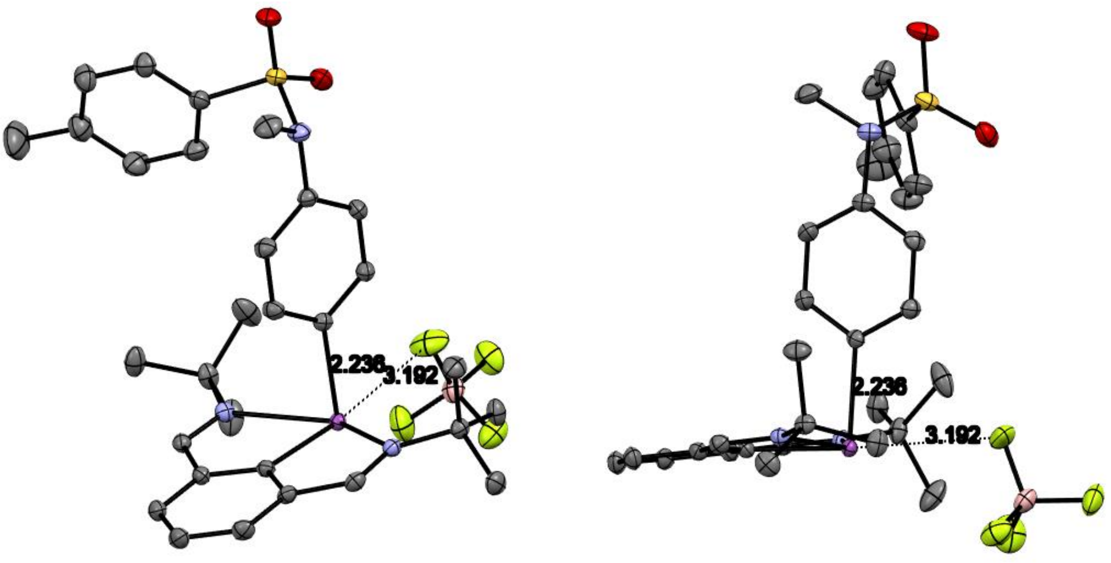

Figure S7. Single-crystal X-ray structure of **9**.

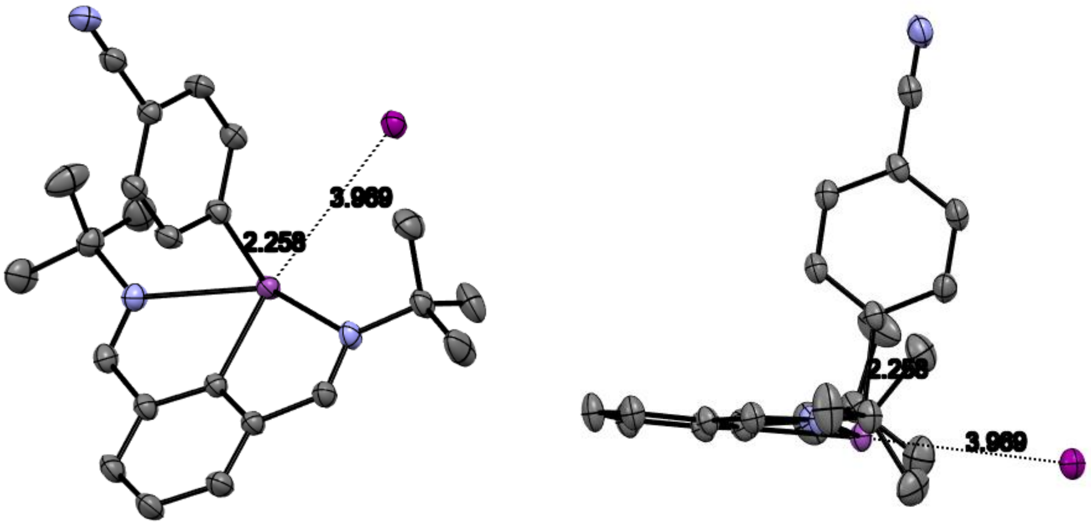

Figure S8. Single-crystal X-ray structure of **12a**.

Assuming that the counteranion does not play a key role, the Bi–Ar distance appears to be longer the more electron poor the aryl group is. This is consistent with the observed trend of lower stability for more electron-poor aryl-bismuth(III) complexes.[3]

6. Electrochemical data

Cyclic voltammograms were collected using a 3-electrode cell consisting of a 3 mm glassy carbon working electrode, platinum wire as the counter electrode, and silver wire as the reference electrode at ambient temperature in an argon-filled glovebox equipped with electrochemical outlets. Sublimed ferrocene was added as the internal reference. For easy comparison with the literature, all potentials were converted to vs. SCE by adding 0.38 V to each value.[8]

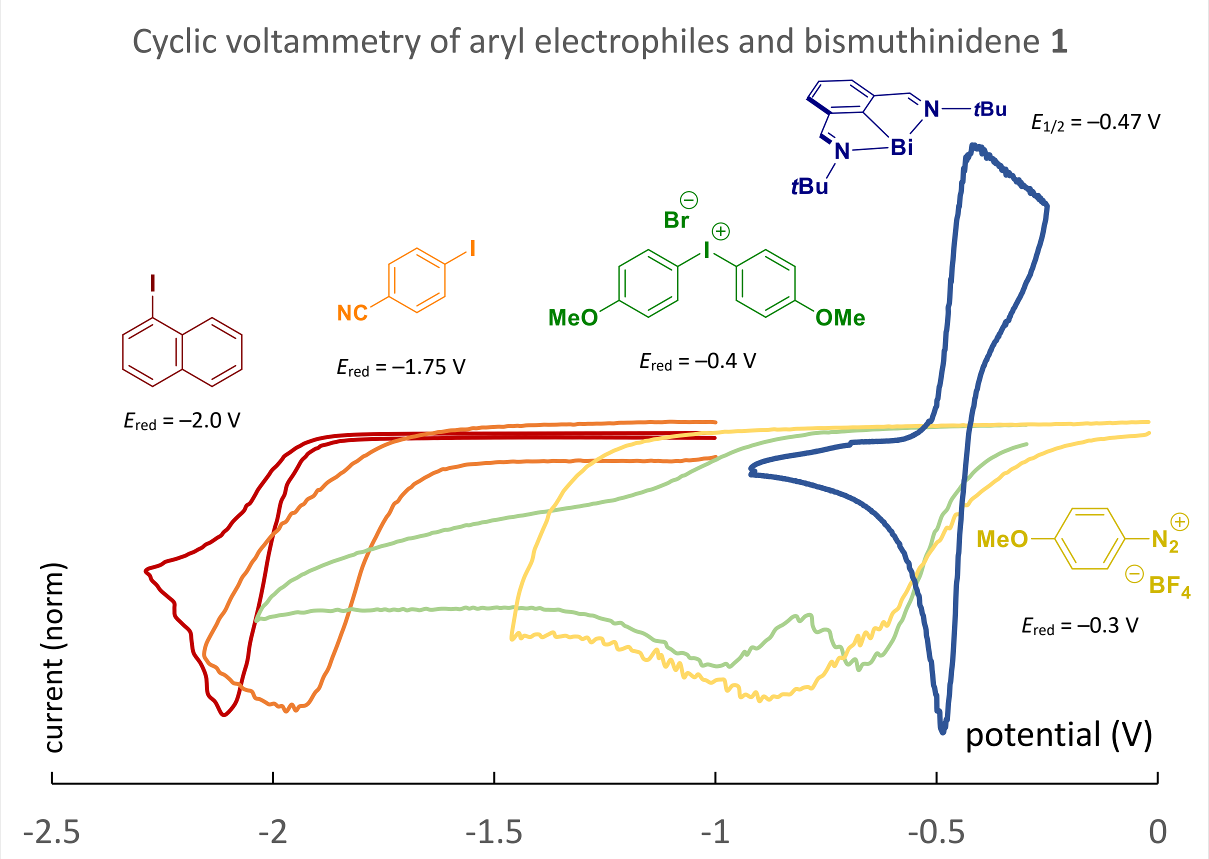

Figure S9. Cyclic voltammogram of bismuth(I) **1** and different types of aryl electrophiles in CH_3CN using 0.1 M [$^{n}Bu_4N$][PF_6] as supporting electrolyte at ambient temperature; scan rate: 100 mV/s. Potential in V vs SCE. Current is scaled for comparability.

A reversible oxidation peak corresponding to the Bi(I)/(II) redox step was observed at –0.47 V for bismuthinidene **1** vs SCE, with an onset potential at ca. –0.6 V vs SCE. Thus, bismuth(I) complex **1** on the ground state (without light irradiation) should be able to easily activate electrophiles showing a reduction event above this number.

Aryl diazonium tetrafluoroborate **2a** and bisaryliodonium bromide **6** undergo single-electron reduction at an on-set potential of around –0.3 V and –0.4 V vs SCE, respectively. This is consistent with the immediate reaction of **1** with these substrates in the absence of light, giving the corresponding SET aryl-oxidative addition adducts.

On the other hand, aryl iodides **10** and **11** undergo single-electron reduction at an on-set potential of around –1.7 V and –2.0 V vs SCE, respectively. This falls way out of the reducing capability of bismuth(I) complex **1** on the ground state. This is consistent with these electrophiles being unreactive with **1** in the absence of light.

However, under visible-light irradiation, **1** becomes significantly more reducing, and it undergoes oxidative addition with these aryl iodides. Aryl halides that are more difficult to reduce (e.g. E_{red} < –2.5 V vs SCE) showed either very slow reactivity or no reactivity at all under light irradiation.

Thus, we can estimate empirically the excited state Bi(I)*/Bi(II) potential to be around -2 V vs SCE.

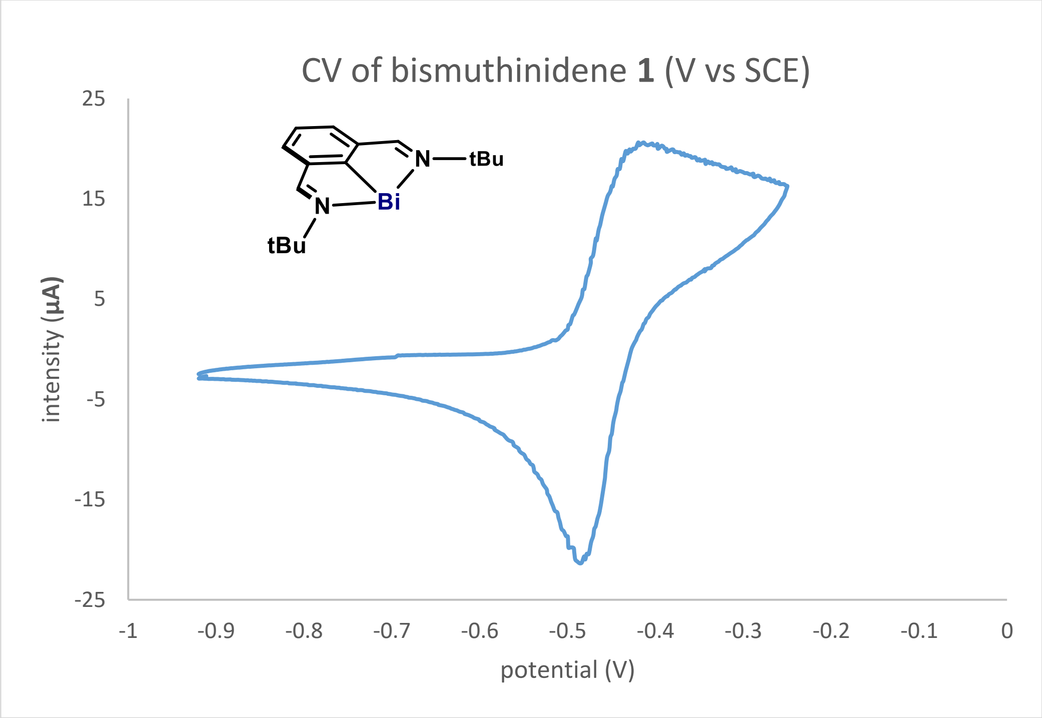

Figure S10. Cyclic voltammogram of bismuthinidene **1** in CH_3CN using 0.1 M [$^{n}Bu_4N$][PF_6] as supporting electrolyte at ambient temperature; scan rate: 100 mV/s. Potential in V vs SCE.

The significantly slower oxidative addition of aryl iodides vs aryl thianthrenium salts can be attributed to the extremely fast fragmentation rate of the latter compared to the former. The relative rate of fragmentation (upon accepting one electron) of aryl thianthrenium salts vs aryl iodides are in the order of 10^{10} favoring aryl thianthrenium salts, as estimated by Ritter and co-workers.[9]

The group of Ritter reported the reduction potential of 4-methoxyphenyl thianthrenium to be –1.5 V vs SCE.[9] This, together with the fast fragmentation rate, explains why these electrophiles are unreactive with bismuthinidene **1** in the dark, but react very rapidly under light irradiation (<5 min).

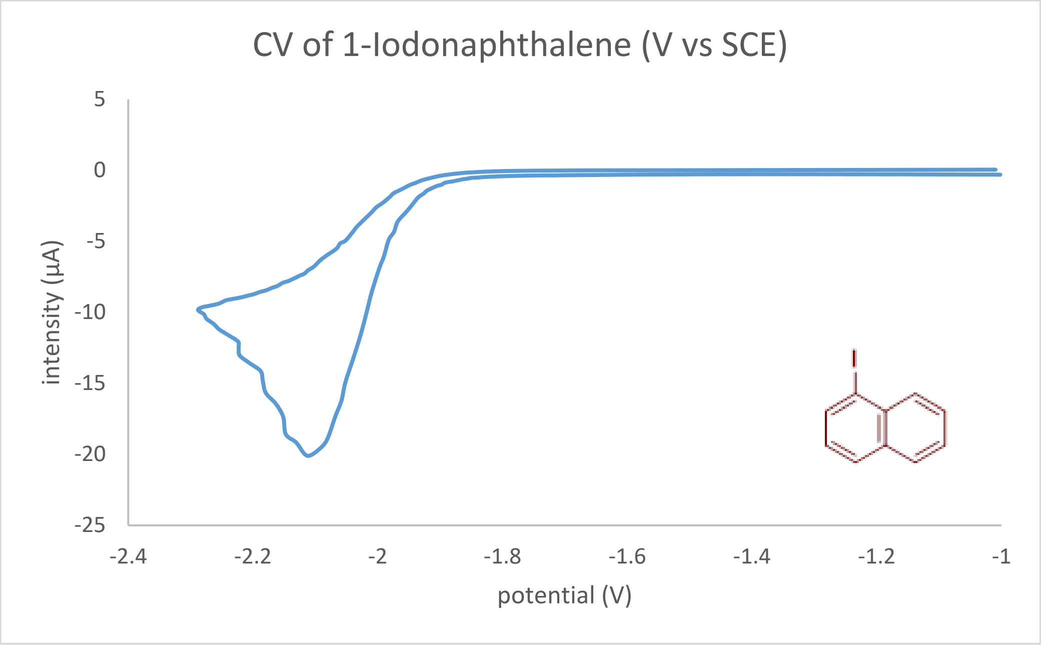

Figure S11. Cyclic voltammogram of **11** in CH_3CN using 0.1 M [$^n Bu_4N$][PF_6] as supporting electrolyte at ambient temperature; scan rate: 100 mV/s, referenced to SCE.

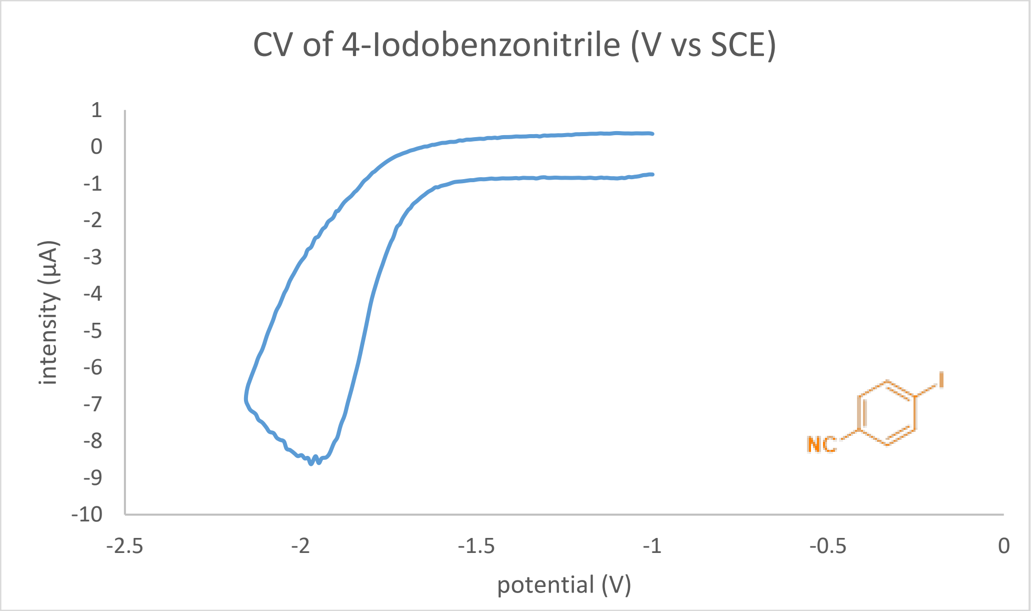

Figure S12. Cyclic voltammogram of **10** in CH_3CN using 0.1 M [$^n Bu_4N$][PF_6] as supporting electrolyte at ambient temperature; scan rate: 100 mV/s, referenced to SCE.

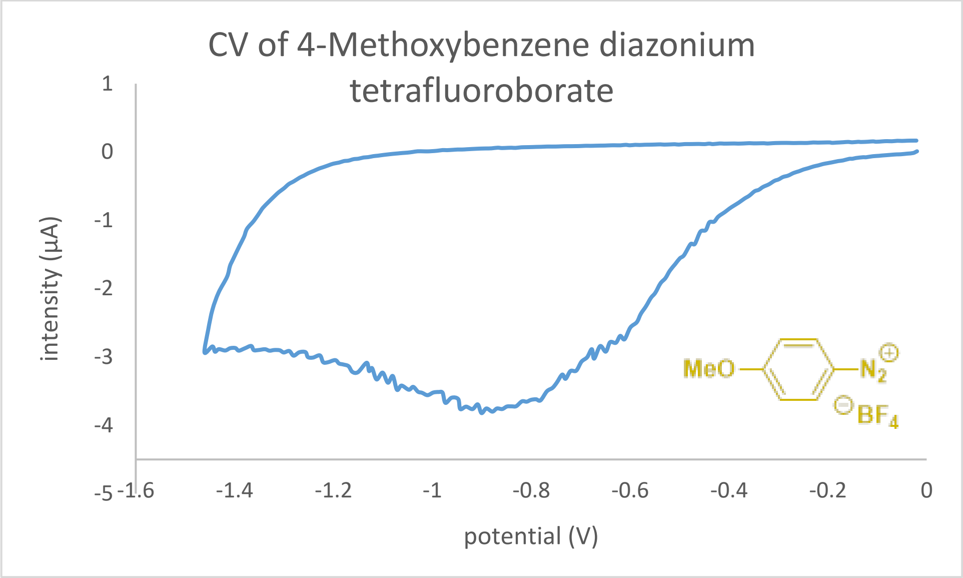

Figure S13. Cyclic voltammogram of **2a** in CH_3CN using 0.1 M [^{n}Bu$_4$N][PF$_6$] as supporting electrolyte at ambient temperature; scan rate: 100 mV/s, referenced to SCE.

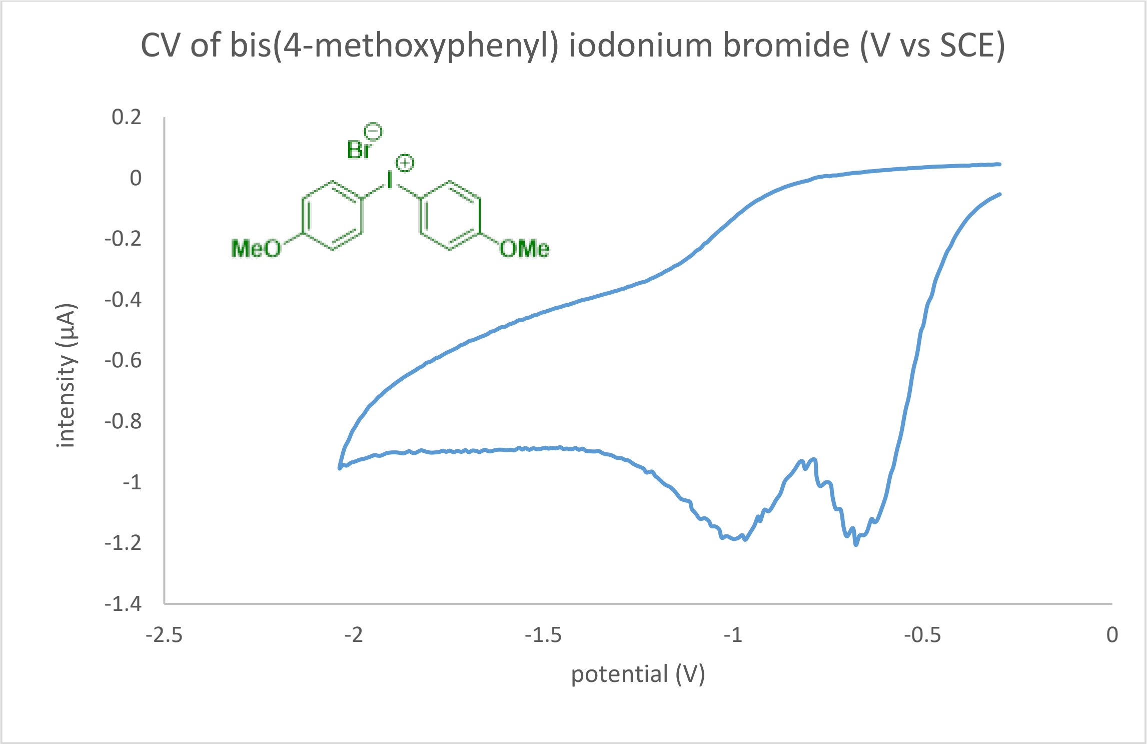

Figure S14. Cyclic voltammogram of **6** in CH_3CN using 0.1 M [^{n}Bu$_4$N][PF$_6$] as supporting electrolyte at ambient temperature; scan rate: 100 mV/s, referenced to SCE.

7. Interception of aryl radical intermediates

In most oxidative additions tested, the corresponding aryl bismuthonium was the major product of the reaction. However, minor products can be formed as a consequence of alternative aryl radical reactivity which competes with the radical recombination.

One of these reactions is the HAT from the solvent. This is more dramatic in THF, but can also occur to some extent in acetonitrile. A significant amount (ca. 15%) of acetonitrile activation product **A** could be obtained when a bulky aryl diazonium salt was employed. The increase of steric bulk hampers radical recombination, favoring HAT. Adduct **A** could be characterized by NMR and HRMS. Preliminary single-crystal x-ray diffraction analysis also suggested the formation of this side-product, but the structure was not of quality high enough for publication.

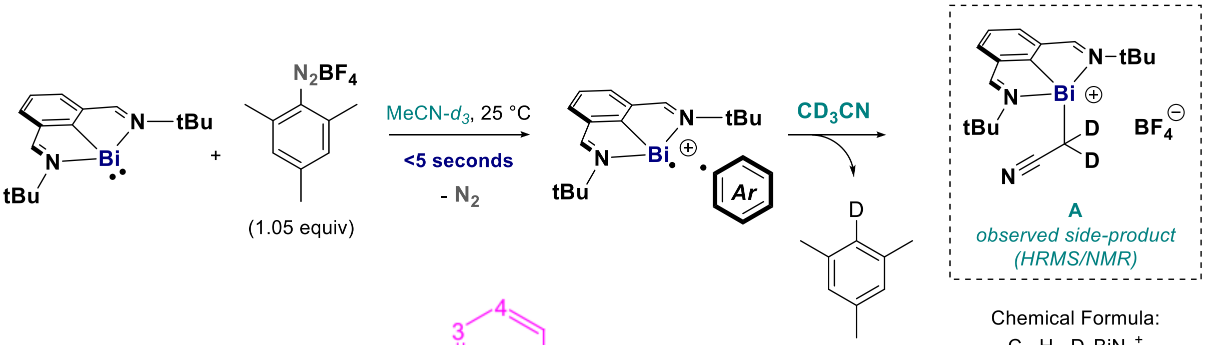

Chemical Formula: $C_{18}H_{23}D_2BiN_3^+$
Exact Mass: 494.19724
found: 494.1965

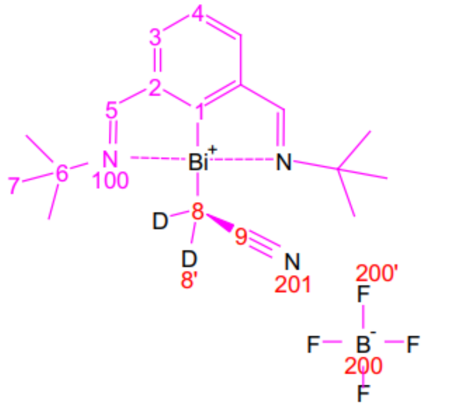

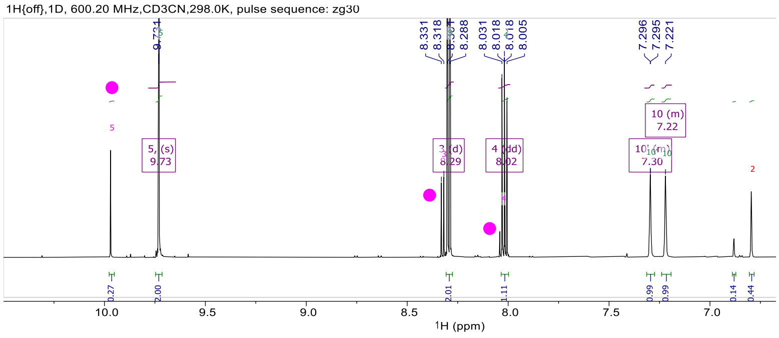

Atom	J	δ (ppm)	HSQC	HMBC
1 C		190.510		3, 5
2 C		149.806		4, 5
3 C		137.633	3	3, 5
H	7.60(4)	8.325	3	1, 3, 5
4 C		131.747	4	
H	7.60(3)	8.029	4	2
5 C		169.650	5	3
H		9.970	5	1, 2, 3, 6, 100
6 C		62.380		5, 7
7 C		30.796	7	7
H3		1.484	7	6, 7, 100
8 C				
8' D				
9 C				
100 N		-72.786		5, 7
200 B				
200' F				
201 N				

More evidence of the intermediacy of aryl radicals was provided when using 4-iodobenzene diazonium tetrafluoroborate as electrophile with bismuthinidene **1**. Besides 76% of the expected oxidative addition adduct and 11% of acetonitrile activation product **A**, 13% of bis-arylbismuthonium dication **C** was observed by NMR, and confirmed by HRMS, together with the same amount of 1,4-diiodobenzene.

This product would arise from reaction of **B** with another equivalent of aryl radical, through halogen-atom-transfer ("XAT"), which would release 1,4-diiodobenzene upon formation of a 4-bismuthonium phenyl radical, which could then recombine with bismuth(II) to give product **C**.

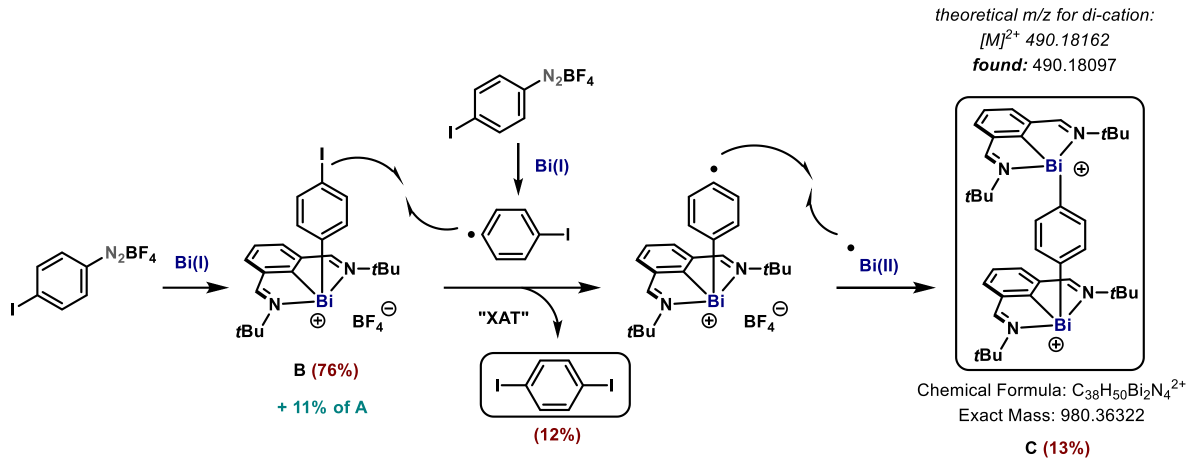

The corresponding aryl radical could also be intercepted by B_2pin_2 (a common radical trap) in 51% yield.

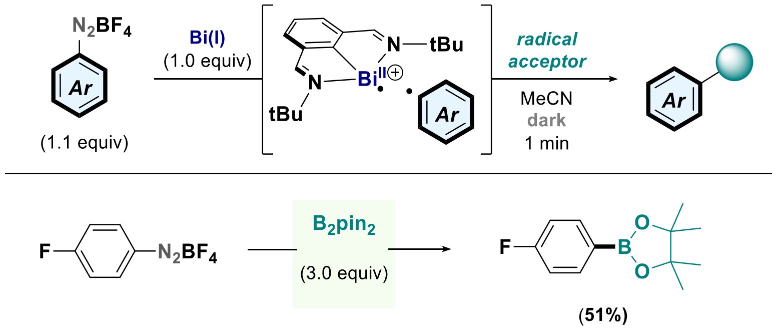

For the trapping experiments, the corresponding aryl diazonium salt (0.11 mmol, 1.1 equiv) and trapping agent (B_2pin_2, 0.30 mmol, 3.0 equiv) were dissolved under argon in 2 mL of dry/degassed MeCN-d_3 (0.05 M). To this solution was added bismuthinidene **1** (0.10 mmol, 1.1 equiv). The mixture bubbled out nitrogen gas immediately. After stirring for 1 min, the vial was taken out of the glovebox, and 1 equiv of 1,4-nitrofluorobenzene was added as internal standard in MeCN-d_3. The yield of product was determined to be 51% by ^{1}H and ^{19}F NMR. Furthermore, flash column in silica gel using 99:1 to 97:3 hexanes/EtOAc as eluent, allowed to isolate the product as a colorless oil in 47% yield.[10] **^{1}H NMR** (300 MHz, $CDCl_3$) δ 7.72 (dd, *J* = 8.2, 6.2 Hz, 2H), 6.97 (dd, *J* = 9.9, 7.7 Hz, 2H), 1.27 (s, 12H). **^{19}F NMR** (282 MHz, CDC13) δ -108.48.

EPR Spectroscopy: DMPO aryl-radical trapping

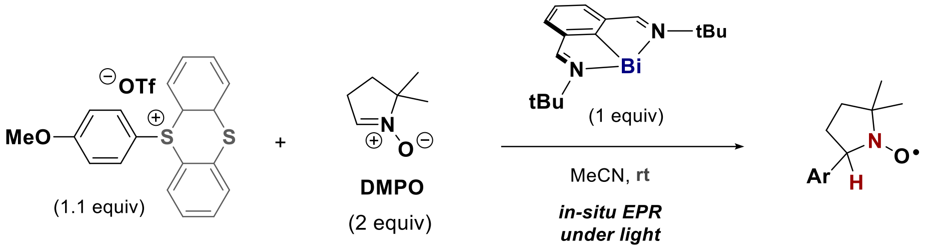

In an argon-filled glovebox, a vial was charged with bismuthinidene **1** (1.0 equiv, 0.020 mmol) 4-methoxyphenyl thianthrenium triflate (1.1 equiv, 0.022 mmol) and DMPO (2.0 equiv, 0.040 mmol). Everything was dissolved in dry degassed acetonitrile (0.5 mL, 0.04 M) and the dark green solution was filtered (through a HPLC filter) into an EPR tube (2.8 or 5 mm). The tube was closed, taken out of the glovebox, and introduced in the EPR instrument. (Unless stated otherwise, the tube was not protected from ambient light. Therefore, some reactivity can be expected even before illumination).

The X-band (microwave frequency of 9.42 GHz) continuous-wave (CW)-EPR spectra were recorded on a Bruker Elexsys E500 spectrometer equipped with a ER4116DM resonator and an Oxford ESR900 cryostat at the corresponding temperature.

A laser-pumped white light source LS-WL1 (lightsource.tech, Göttingen) was connected to the optical port of the resonator using a 1 mm optical fiber and a 5 mm collimator and turned on for the experiments under illumination. The light intensity reaching the sample tube is estimated to be of the order of 50 mW.

EPR spectra were simulated by using the Easyspin package (version 6.0.0 dev 50).[11]

Additional details of the EPR spin-trapping study

The spin-trapping experiments performed in this study are reported in Figure S15. The spin-trapping methodology is based on the capturing of short-lived radicals by reacting with a diamagnetic spin-trap molecule that forms a paramagnetic adduct with sufficient lifetime to be detected by CW-EPR. In this work, 5,5-dimethyl-1-pyrroline-*N*-oxide (DMPO) was employed as spin trap. Due to the different hyperfine couplings between the unpaired electron and the ^{14}N nucleus as well as the ^{1}H nucleus in the beta position to the radical adduct, it is possible to indirectly identify the nature of the short-lived radicals generated during the reaction.[12]

An EPR signal characterized by ^{14}N isotropic hyperfine coupling a_N = 14.45 G and ^{1}H isotropic hyperfine coupling a_H = 21.41 G was observed (Figure S1). The a_N and a_H coupling constants retrieved from the spectrum agree with literature values reported for DMPO-anisole adducts in solution,[13] allowing us to assign the trapped species to the anisole radical. Although a weak signal is already visible after keeping the EPR tube for about 1 h under ambient light, upon illumination, the intensity of such species increased significantly, dominating the EPR spectrum. Upon direct illumination, a triplet signal (marked with asterisks in Figure S1) also appeared in the spectra and gained more intensity after leaving the light on (green, blue and brown traces). The three-line spectrum is consistent with a nitroxide radical showing splitting only on a nitrogen atom (a_N = 14.27 G). This signal has been previously identified as di-*tert*-butyl-nitroxide generated by the ring-opening of DMPO itself.[14] Computer simulation of this spectrum

showed that the amount of DMPO degradation product corresponds to 15% of the total EPR signal under continuous illumination.

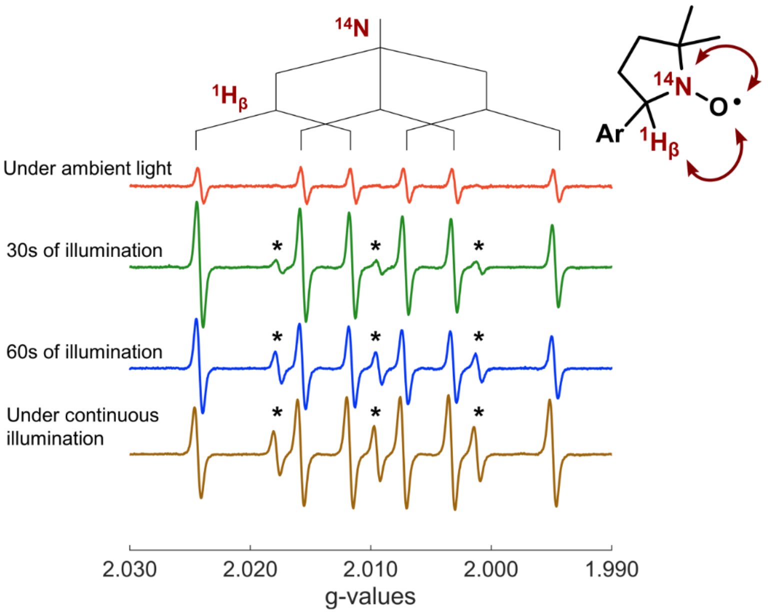

Figure S15. X-band spectra recorded at room temperature of the reaction between bismuthinidene **1** and 4-methoxyphenyl thianthrenium tetrafluoroborate in presence of DMPO in acetonitrile under different irradiation times. The asterisks indicate the signal of an unidentified nitroxide radical.

Control experiments without bismuth(I), or without aryl thianthrenium salt gave no detectable EPR signal. Furthermore, the oxidative addition adducts by themselves (e.g.: **3c**) were also EPR-silent.

8. Photophysical properties

UV-Vis absorption spectroscopy

A 0.1 mg/mL solution of bismuthinidene **1** in dry and degassed acetonitrile (prepared in an argon-filled glovebox in a sealable cuvette as the one in the picture below) was analyzed by UV-Vis absorption spectroscopy, using the same, degassed solvent as blank.

UV-Vis spectra have been recorded on a Cary6000i UVVIS/NiR spectrometer in 2 mm Suprasil Quartz cuvettes.

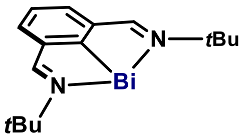

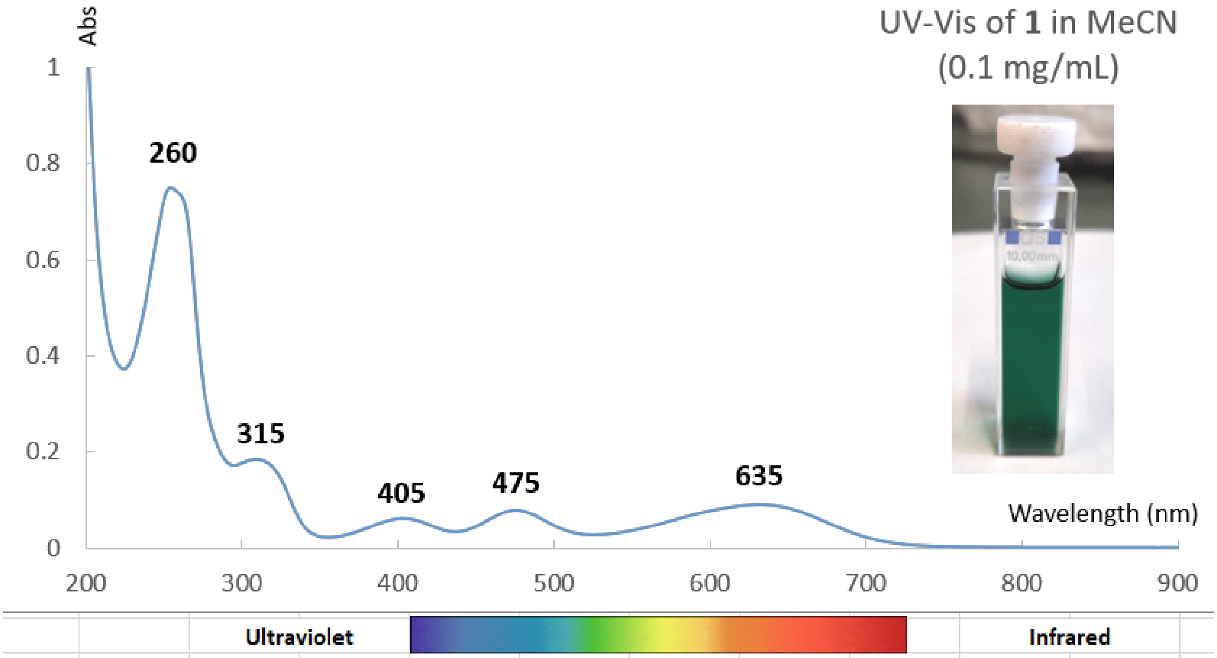

Figure S16. UV-Vis spectrum of **1** in MeCN

Emission spectroscopy

A 0.1 mg/mL solution of bismuthinidene **1** in dry and degassed acetonitrile (prepared in an argon-filled glovebox in a sealable 10x10 mm quartz-glass cuvette as the one depicted in the previous section) was analyzed on a Cary Eclipse spectrometer in a 90 degree setup from 300 to 1100 nm with the corresponding excitation wavelength.

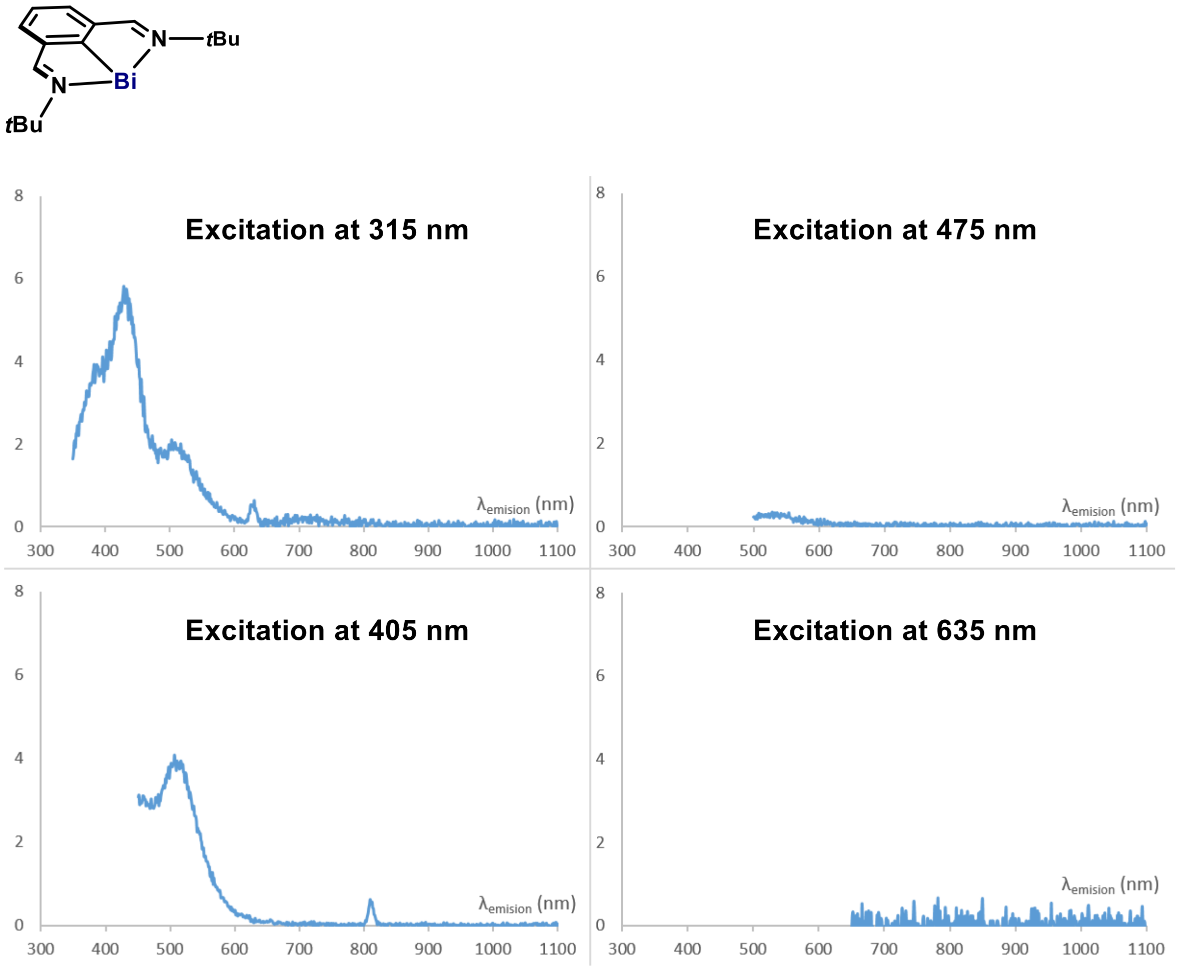

Figure S17. Emission spectra of **1** at different excitation wavelengths.

Whereas some emission was observed upon excitation at higher energy (315 or 405 nm), no emission could be detected up to 1100 nm when exiting at the 475 nm or 635 nm maxima.

Presumably, due to the high spin-orbit coupling in heavy-element containing molecules, a non-radiant self-quench of the excited states can take place.[15] This makes radian emission non-observable in the time scale and/or wavelength range analyzed. This fluorescence self-quenching could also be responsible of the low photon efficiency observed in the oxidative addition of aryl iodides (requiring high intensities over long periods of time to afford full conversion).

For the higher excited states reached with purple or blue light excitation, some weak emission with a significant Stokes shifts was observed.

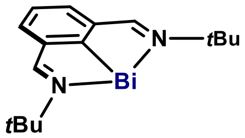

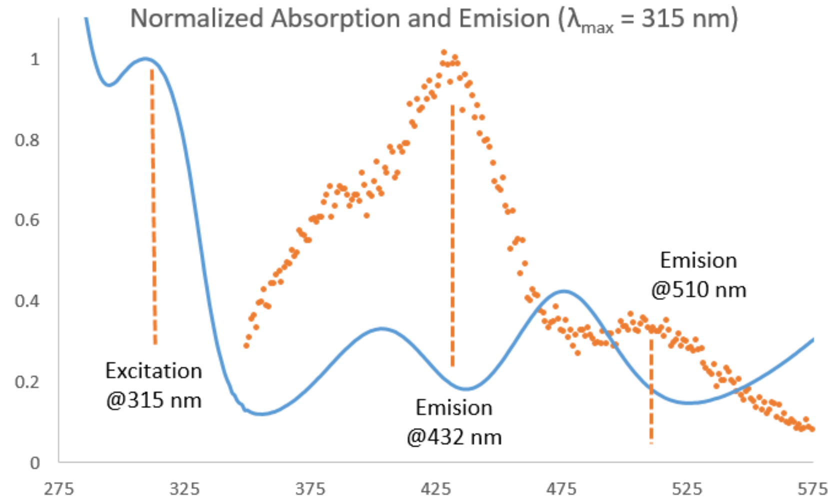

Figure S18. Normalized absorption and emission after excitation at 315 nm.

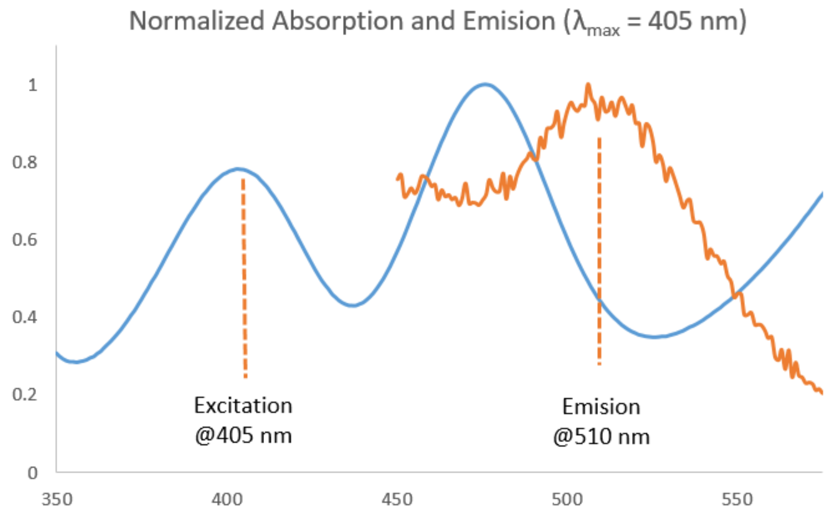

Figure S19. Normalized absorption and emission after excitation at 405 nm.

UV-Vis absorption of a mixture of bismuthinidene and electrophile

In order to evaluate potential charge-transfer interaction between the two reagents in solution, we conducted UV-Vis absorption analysis of a solution of bismuthinidene **1** (0.1 mg/mL in MeCN), a solution of 4-iodobenzonitrile (0.2 mg/mL in MeCN) and a 1:1 mixture of the two solutions (0.05 mg/mL of **1**, 1 equiv, and 0.1 mg/mL of 4-iodobenzonitrile, 4 equiv).

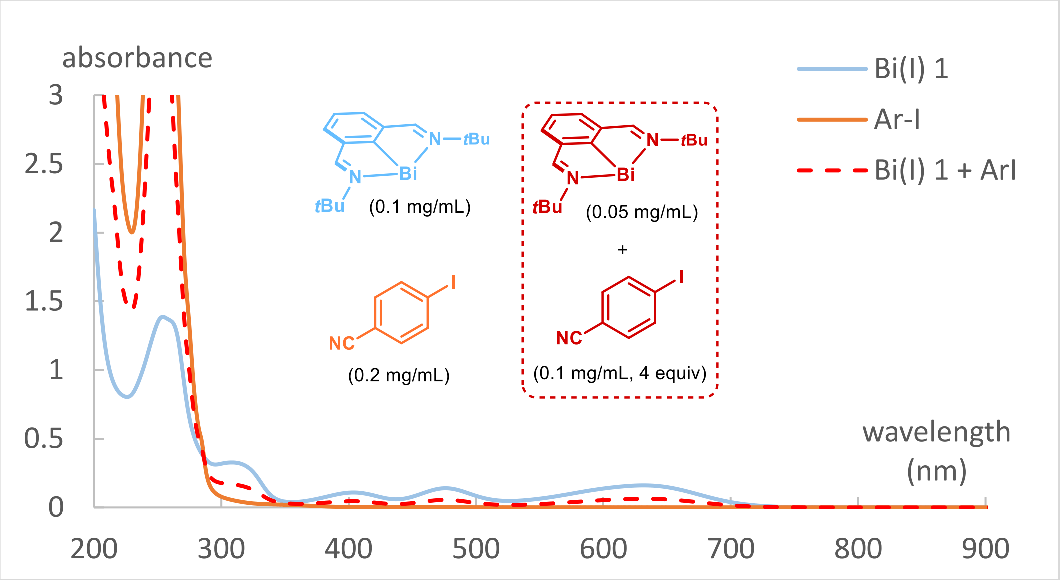

Figure S20. UV Vis absorption spectra of **1**, an electrophile, and a mixture of both.

We found no significant shift in the absorption bands. For easy visualization, a normalized zoomed in version of the spectrum is attached below.

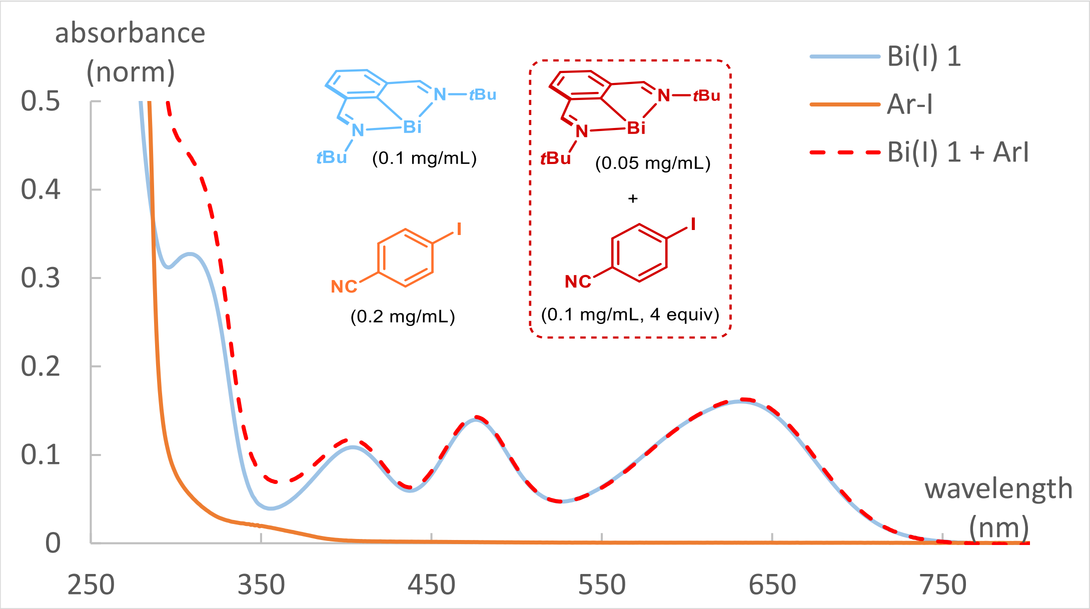

Figure S21. Zoomed in UV Vis absorption spectra of **1**, an electrophile, and a mixture of both.

Thus, no charge transfer of EDA complex formation can be observed, further supporting the hypothesis of direct excitation of **1** followed by SET.

A similar analysis was performed by ^{1}H NMR in MeCN-d_3.

A 0.01 M solution of bismuthinidene **1** in MeCN-d_3 (4.5 mg/mL, 0.01 mmol) of bismuthinidene **1** in MeCN-d_3 was first prepared and measured by NMR. A second 0.01 M solution of 4-iodobenzonitrile in MeCN-d_3 (2.3 mg/mL, 0.01 mmol) was prepared and measured by NMR. Then, a 1:1 mixture of the two solutions was prepared and also measured by NMR.

Again, no significant shift or broadening of the signals that might suggest a bonding interaction was observed.

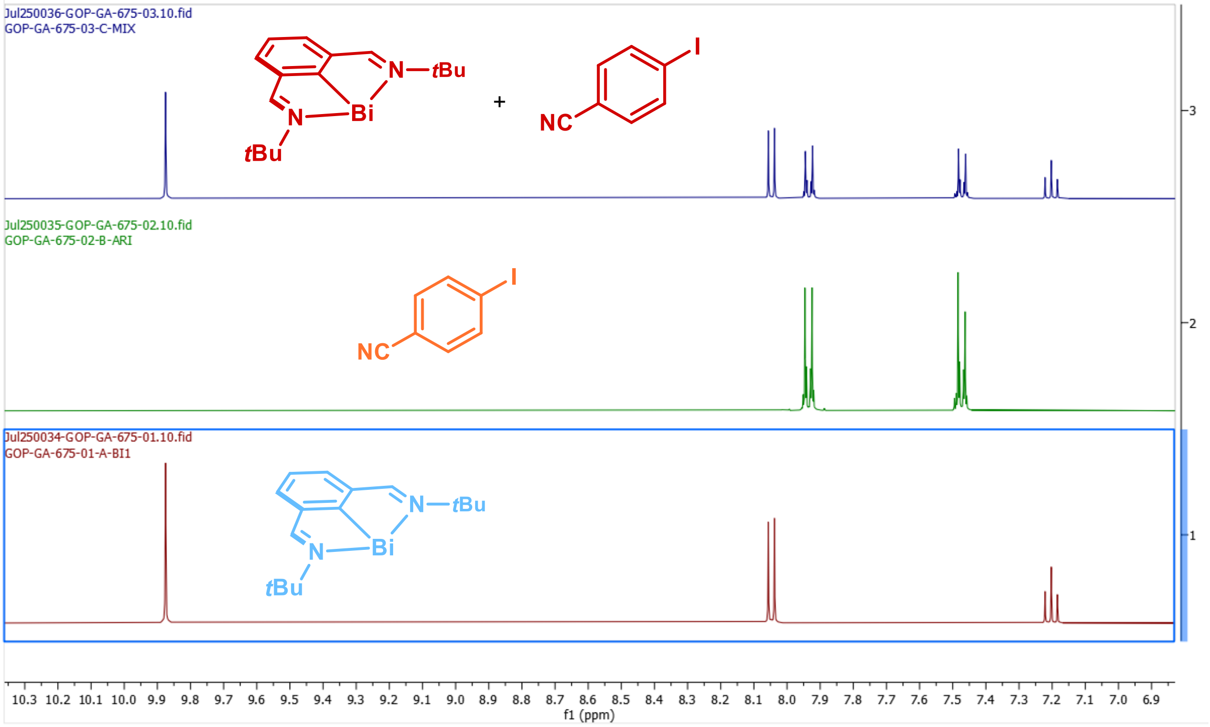

Figure S22. ^{1}H NMR spectra of **1** (bottom), an electrophile (middle), and a mixture of both (top).

9. Computational study

Geometry optimization and vibrational frequency calculations

All computations were performed using ORCA 5.0.4 and a development version of ORCA 5.0.[16] Geometry optimizations and vibrational frequencies calculations of Bi(I) **1** in a singlet spin state (ground state) and as a triplet (excited state) as well as its singly oxidized Bi(II) form were performed in the frame of scalar relativistic density functional theory (DFT) in combination with the ZORA Hamiltonian.[17] The B3LYP functional[18] was employed, in conjunction with the D3 method (D3BJ) developed by Grimme *et al.*[19] to take into account dispersive interactions. All the elements (apart from the Bi atom) were treated with the def2-TZVP basis set with exponents from def2-TZVP[20] re-contracted for ZORA by D. A. Pantazis. For the Bi center, the SARC basis set (Tl-Rn) was adopted.[21] The RIJCOSX[22] approximation was used in conjunction with the SARC/J fitting basis,[21,23] which is the decontracted version of the def2/J auxiliary basis sets for elements up to Kr. The geometries in acetonitrile solution were determined self-consistently using the SMD model. Tight convergence and optimization criteria (TightSCF, TightOpt) and a fine grid (DefGrid3) were used. Vibrational frequencies were calculated analytically[24] for the optimized geometries to obtain the zero point vibrational energy (ZPVE) and thermochemical contributions.

Inspection of the optimized structure of **1** in a singlet spin state and **1*** in a triplet spin state (Figure S2) shows a slight decrease of the Bi-N bond distance in **1*** and an increase of the Bi-aryl bond compared to **1**. Loewdin population analysis of **1*** indicates a spin population on the Bi (0.79) while the remaining spin population is distributed over the *N*,*C*,*N* ligand. These results suggest the presence of one electron on bismuth with another electron of the same spin on the ligands, which is consistent with the occurrence of MLCT transitions.

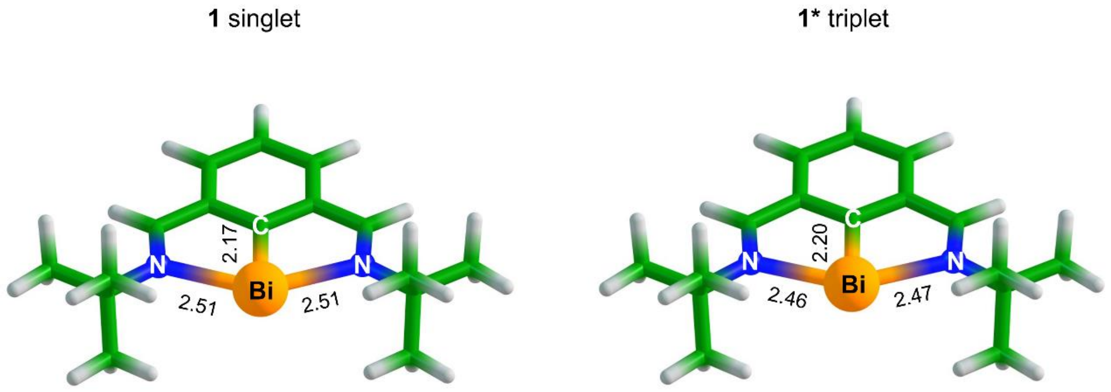

Figure S23. Optimized structures obtained for **1** (singlet) and **1*** (triplet) within the SMD solvation model in acetonitrile. Bi-N and Bi-C bond distances are also given in Ångstrom. C, N, H and Bi atoms are depicted in green, blue, white and orange, respectively

Investigation of excited states for 1 through CASSCF/NEVPT2 calculations

The electronic structure of **1** was further investigated by means of single-point state-averaged complete active space self-consistent field (SA-CASSCF) calculations performed at the DFT optimized geometry of **1** and dynamic electron correlation was incorporated using the N-electron valence second-order perturbation theory (NEVPT2). An active space (CAS) comprising of 6 electrons in 7 orbitals was employed (CAS(6e,7o)). This is composed by the two σ orbitals involving the bonding between Bi $6p_x$ with the two sp^2 orbitals of the N atoms ($6p_x+\sigma_x$) and Bi $6p_y$ with the sp^2 orbital of the C atom ($6p_y+\sigma_y$), the Bi $6p_z$ orbital containing the lone pair ($6p_z+\pi_z$), the corresponding antibonding orbitals and the MLCT orbital (see Figure S21). X2C scalar relativistic Hamiltonian,[25] as implemented into a development version of ORCA, was employed. The X2C-QZVPall-2c basis set[26] was adopted for Bi, X2C-TZVPPall-2c for C and N atoms and X2C-SVPall-2c for the H nuclei. Spin-orbit coupling (SOC) was treated using the mean-field SOC Hamiltonian,[27] including picture change effects. The scalar relativistic states composed by 6 singlets and 5 triplets were allowed to interact via the SOC interaction.

SA-CASSCF calculations predicted a singlet ground state for **1**, as expected. Occupational numbers of the optimized state-averaged active space indicate that 5.03 electrons are contained in the occupied orbitals while the remaining 0.97 is spread over the unoccupied orbitals, with a major contribution from MLCT orbital (LUMO) and the $6p_z+\pi_z$ orbital (LUMO+1).

The first excited state is predicted to possess a triplet spin multiplicity where the 97% of the wavefunction is represented by the following configuration: $(6p_x+\sigma_x)^2(6p_y+\sigma_y)^2(6p_z+\pi_z)^{\uparrow}(\mathrm{MLCT})^{\uparrow}$. The corresponding transition (HOMO→LUMO) represents therefore a spin-forbidden transition $S_0 \rightarrow T_1$ that is calculated to appear at 13229 cm^{-1}≡756 nm. The SOC interaction splits such transition into three transitions falling in the red-light range, namely 14189 cm^{-1}≡704 nm, 14528 cm^{-1}≡688 nm and 14529 cm^{-1}≡688 nm. Although the oscillator strength of such transitions is relatively low compared to the analogous spin-allowed $S_0 \rightarrow S_1$ transition (with configuration $(6p_x+\sigma_x)^2(6p_y+\sigma_y)^2(6p_z+\pi_z)^{\uparrow}(\mathrm{MLCT})^{\downarrow}$), these calculations pinpoint that direct $S_0 \rightarrow T_1$ may be enabled through the assistance of SOC. Moreover, it has been shown that bismuth is capable of achieving long-lived excited states due the high SOC constant.[28] This allows for easy singlet−triplet crossover which contributes, together with direct $S_0 \rightarrow T_1$ transitions, to the population of the reactive ^{3}MLCT state.

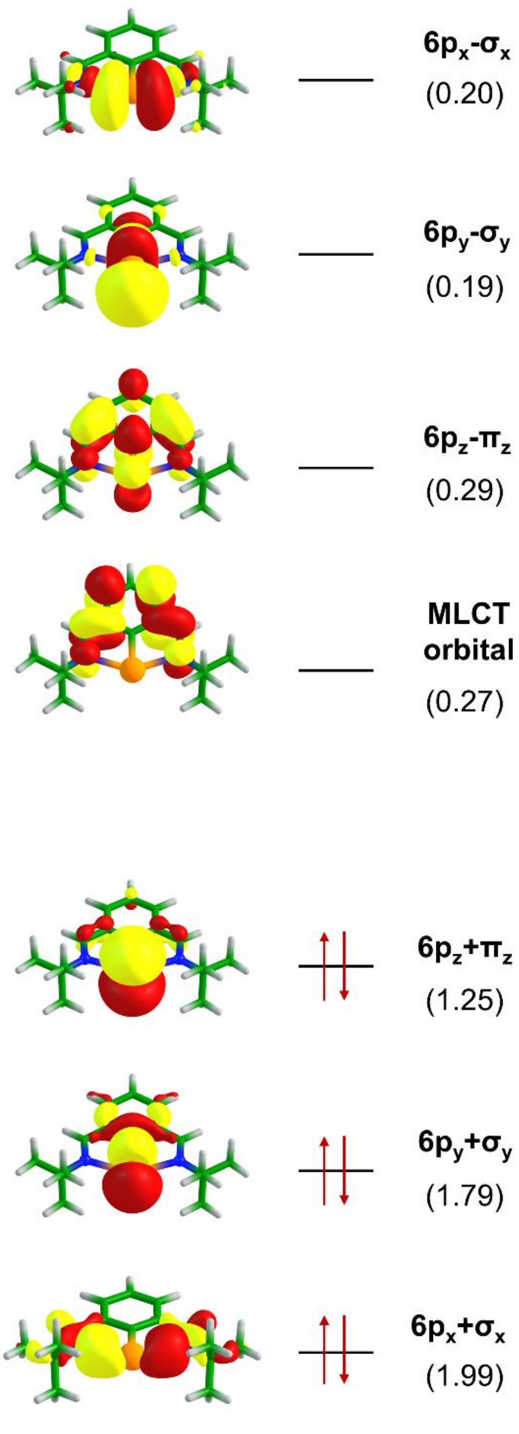

Figure S24. Contour plots of the natural orbitals optimized with CASSCF/NEVPT2(6e,7o). Qualitative nature and fractional occupation number (n) of the orbitals are reported in parenthesis. C, N, H and Bi atoms are in green, blue, white and orange, respectively.

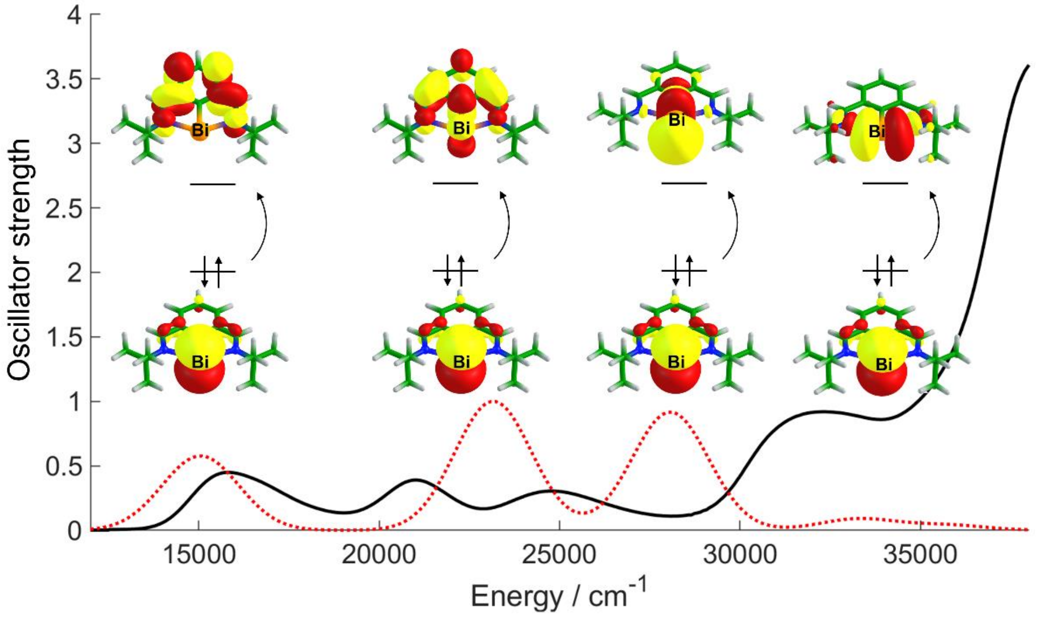

Figure S25. Experimental (black line) and CASSCF/NEVPT2 (dotted red line) UV-Vis spectra.

The contour plot of the molecular orbitals involved in the transitions corresponding to the first four bands of the spectrum is reported.

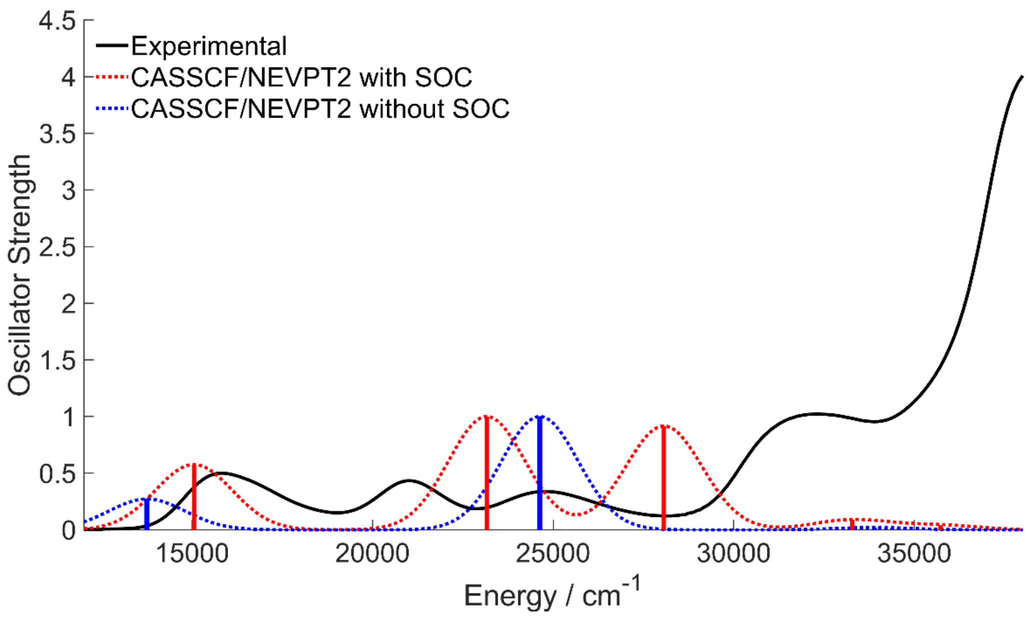

Figure S26. Comparison between experimental (black line) and CASSCF/NEVPT2 (dotted red line) UV-Vis spectra computed with and without SOC interaction.

The calculated SOC-corrected spectrum agrees better with the experimental one by predicting correctly three transitions at ≈23000 cm^{-1}, 28000 cm^{-1} and 33000 cm^{-1} instead of only two transitions at ≈16000 cm^{-1} and ≈25000 cm^{-1} obtained without SOC (although a blue shift is present, probably due to insufficient capturing of differential dynamic correlation effects[18]). Moreover, the MLCT transition calculated with SOC correction appears closer in energy with the experimental spectrum.

Calculation of the redox potential for Bi(II)/Bi(I) of 1

Single point energies were carried out on the optimized structures at various levels of theory to calculate the redox potential of *N*,*C*,*N*-Bi(II)/*N*,*C*,*N*-Bi(I) couple of bismuthinidene **1**, considering the *N*,*C*,*N*-Bi(I) as a singlet (ground state) or as triplet (excited state). B3LYP and PBE0[29] functionals with D3BJ were used. Effective core potentials (ECPs) were employed in conjunction with the CPCM solvation model.[30] def2-TZVP together with def2-SD (for Bi)[31] basis sets were employed. The auxiliary basis sets def2/J[32] and def2/JK[33] were also adopted. The redox potential of the Bi(I)/Bi(II) couple in acetonitrile was calculated according to:

$$E^0_{Bi(I)/Bi(II)} = \frac{\Delta G_{ox}}{nF} - SCE$$

where ΔG_{ox} is the Gibbs free energy difference between the reductant and the oxidant in aqueous solution, *n* is the number of electrons which attribute to the reaction, *F* is Faraday constant and *SCE* is the absolute reduction potentials of saturated calomel electrode in acetonitrile at 298 K. In this work, the value of 4.60 V was consistently used in all calculations.[34] The superscript circle indicates the standard state of 1 atom ideal gas for gases and a standard state of 1 molar ideal solution for solute. The difference of the Gibbs free energy ΔG_{ox} is defined as:

$$\Delta G_{ox} = IE(0K) + \Delta G^0_{trans,vib,rot-TS} + \Delta\Delta G_{solv}$$

where $IE(0K)$ is the adiabatic ionization energy at 0 K with the consideration of ZPVE.

$$IE(0K) = \Delta U_{elec} + ZPVE$$

whereby ΔU_{elec} being the difference of total electronic energy between Bi(II) and Bi(I). $\Delta G^0_{trans,vib,rot-TS}$ involves the thermal contribution of translational, vibrational, and rotational motion at 298 K and the stabilization by entropic contribution. $\Delta\Delta G_{solv}$ is given as a difference of the solvation energy of oxidized and reduced species.

$$\Delta\Delta G_{solv} = \Delta G^0_{solv}(Ox) - \Delta G^0_{solv}(Red)$$

with ΔG^0_{solv} indicating the Gibbs free energy associated with moving a single ion from the gas phase into solution.

		Level of theory	E^0 at 298 K (V)
**1**(II)/**1**(I)	Calculated	B3LYP	–0.71
		PBE0	–0.59
	Experimental		–0.47
**1**(II)/**1***(I)	Calculated	B3LYP	–1.92
		PBE0	–1.79

Table S1. Comparison between computed and experimental redox potentials for the **1**(II)/**1**(I) and **1**(II)/**1***(I) couples in acetonitrile at 298 K. Potential (V) vs SCE.

The calculated redox potential for the **1**(II)/**1***(I) couple is consistent with the range of aryl electrophiles that can be activated with **1** under light irradiation (up to ca. E_{red} = –2 V vs SCE).

10. Kinetic analysis and other mechanistic experiments

For all kinetic experiments of the light-promoted oxidative addition of aryl iodides, the reactions were conducted at 0.01–0.02-mmol scale (concentration kept identical when comparing kinetic profiles), in an NMR tube using anhydrous/degassed deuterated acetonitrile as solvent and 1,3,5-trimethoxybenzene as internal standard. The reaction tube was exposed to the corresponding light for a given time, and then, ^{1}H NMR was used to determine conversion and yield. After measuring, the same reaction tube was exposed again to the light source to continue building the kinetic profile. As shown in control and on/off experiments, these reaction do not proceed in the absence of direct, high intensity light irradiation. The good linear fittings to the expected zero-order profiles obtained with this approach validate the accuracy of this method. For simplicity, and for reactions with the same concentration of substrate, kinetic data is shown as a function of yield of product (%) vs time. Thus, unless stated otherwise, *k* (which for zero order reactions corresponds to the slope of the straight line) are in "yield/h" units, which can be translated into "M/h" considering the concentration of the reaction.

10.1. Influence of the wavelength in the oxidative addition of aryl iodides

Influence of light in the oxidative addition of aryl iodides

We have found that the aryl bismuthonium cations are sensitive to high energy visible light, and eventually decompose (giving complex mixtures) when exposed to blue or white LED irradiation for several hours.

Whereas the oxidative additions of aryl thianthrenium salts are very fast, and they can be promoted cleanly with any light of any color (there is no time for the product to decompose significantly, see previous section), we found that not to be the case for the oxidative addition of aryl iodides.

Since they are much slower, under blue light, the product decomposes too fast, and a clean oxidative addition could not be carried out.

However, since the oxidative addition adducts do not absorb low energy light (e.g. green or red) significantly, but the starting bismuth(I) does, this reaction can be promoted very cleanly using low-energy-light LEDs.

Influence of light in the oxidative addition of aryl iodides

The reaction was followed using irradiation from different light colors, with LEDs with an estimated maximum output power of 20 W. The number of photons reaching each reaction may vary due to fabrication of the different light strips, and slight differences in reaction set-up. Hence, this only aims to be a qualitative comparison on selectivity. Reaction performed with 0.010 mmol of Bi(I) and 0.020 mmol of 4-iodobenzonitrile in 0.6 mL of MeCN-d_3 (0.0166 M), in an NMR tube. Yields determined by ^{1}H NMR using 1,3,5-trimethoxybenzene as internal standard.

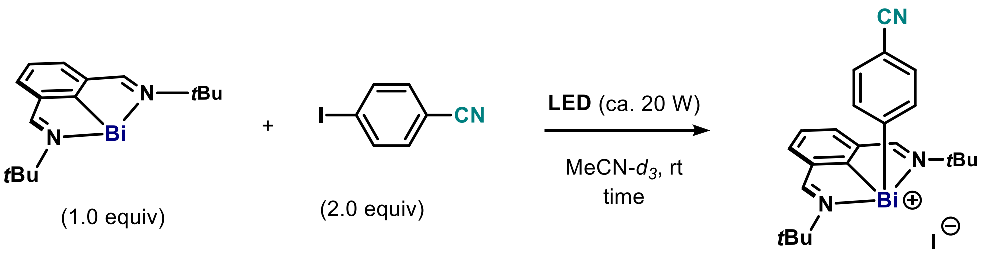

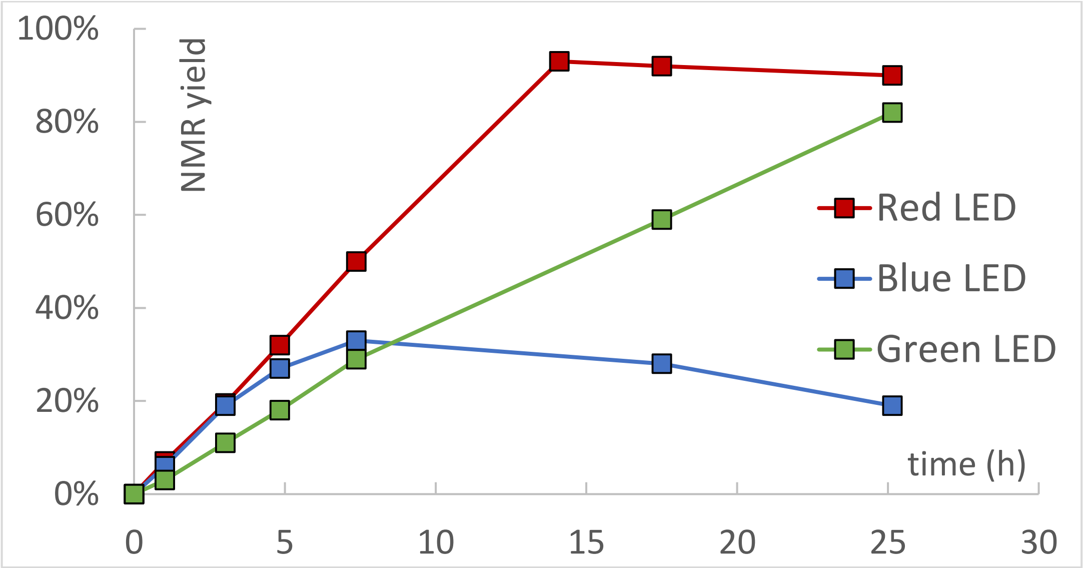

Figure S27. Reaction profiles using different light wavelengths.

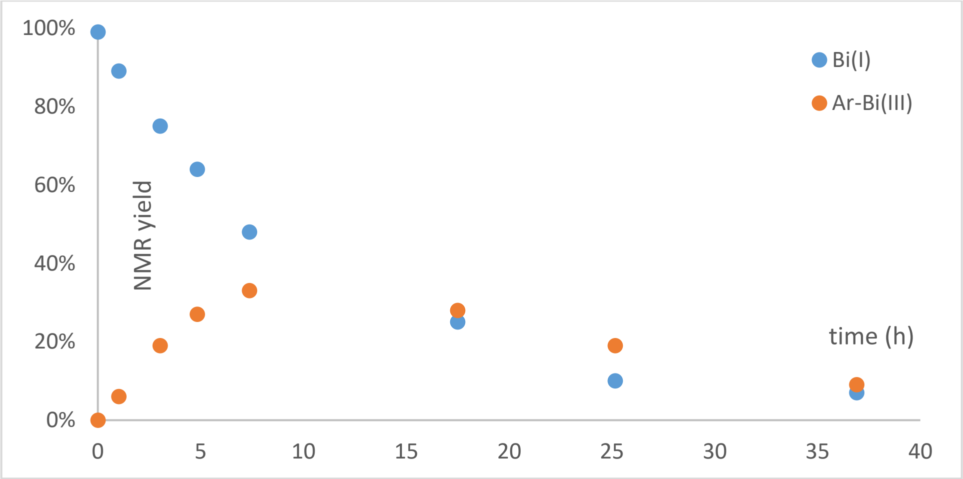

Figure S28. Blue LED (465 nm) full profile. *After 7 days (168 h) under blue-light irradiation, no Ar-Bi(III) was detected (complex mixture).

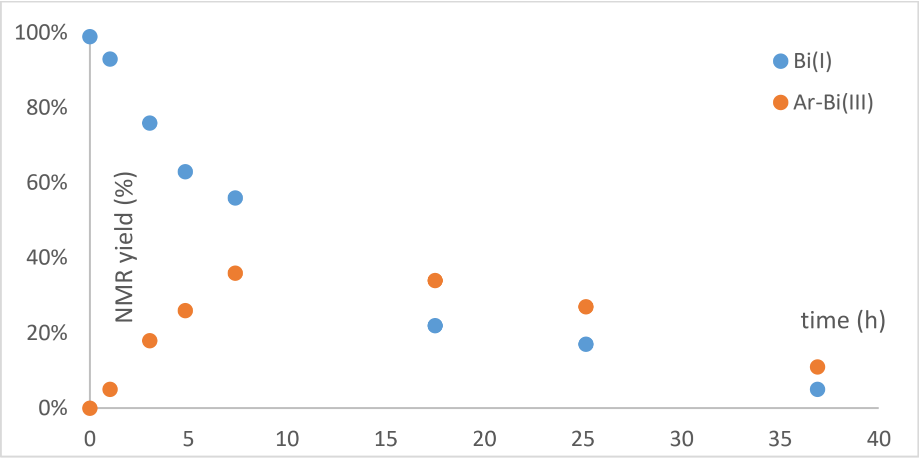

Figure S29. White LED full profile. *After 7 days (168 h) under white-light irradiation, no Ar-Bi(III) was detected (complex mixture).

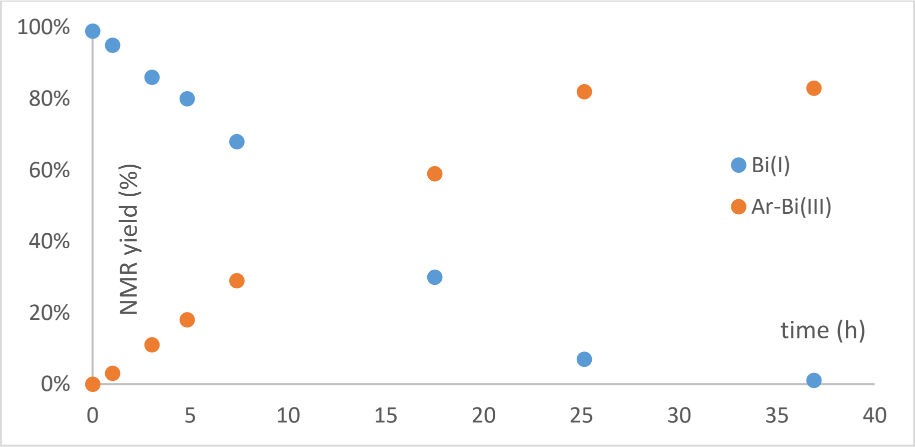

Figure S30. Green LED (550 nm) full profile. *After 7 days (168 h) under green-light LED irradiation, 26% of Ar-Bi(III) complex remained.

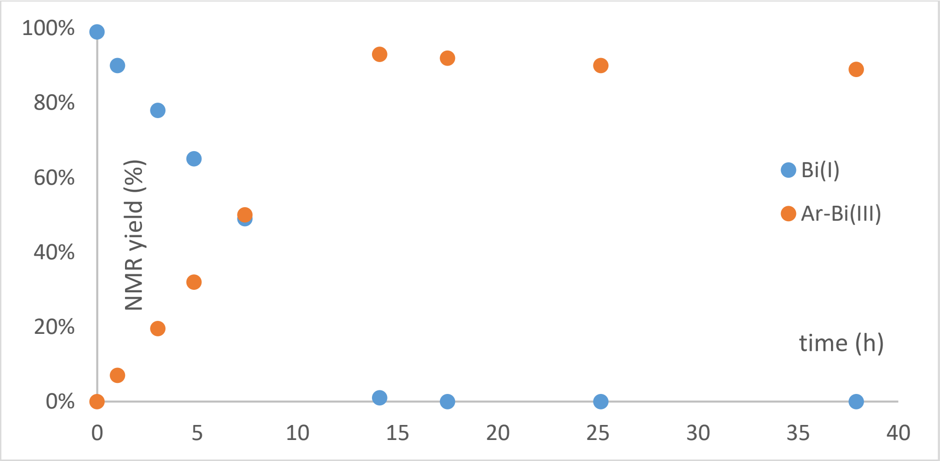

Figure S31. Red LED (660 nm) full profile. *After 7 days (168 h) under red-light LED irradiation, 72% of Ar-Bi(III) complex remained.

Influence of the red-light source in the oxidative addition of aryl iodides

As expected, the intensity of the light dictates the rate of conversion of the oxidative addition reaction. Different light sources/set-ups were compared for the reaction of bismuthinidene **1** with 1-iodonaphthalene. Except for the last entry, the reactions performed with 0.010 mmol of Bi(I) and 0.020 mmol of 1-iodonaphthalene in 0.6 mL of MeCN-d_3 (0.033 M), in an NMR tube. Yields determined by ^{1}H NMR using 1,3,5-trimethoxybenzene as internal standard.

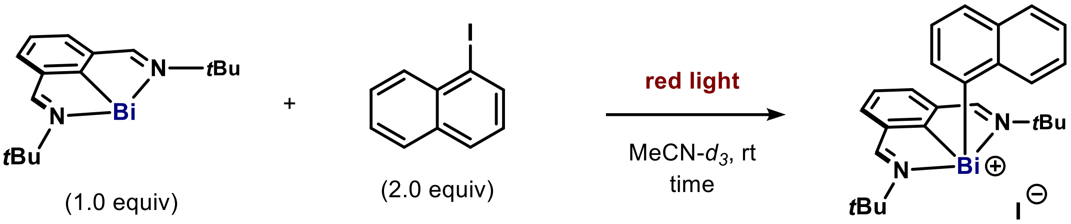

red LED strip (650 nm, ca. 20 W) *0.02 mmol Bi(I)* — full conversion Bi(I), 93% yield **(85 h)**

focused 660 nm laser, (300 mW) *0.02 mmol Bi(I)* — full conversion Bi(I), 93% yield **(92 h)**

2x Kessil LED (660 nm, 100% int, ca. 80 W) *0.02 mmol Bi(I)* — full conversion Bi(I), 95% yield **(24 h)**

2x Kessil LED (660 nm, 100% int, ca. 80 W) ***0.1 mmol Bi(I)*** — full conversion Bi(I), 95% yield (67% isolated) **(48 h)**

Under 300 mW of monochromatic focused 660 nm laser light, slower reaction was observed, following a clear zero-order rate.

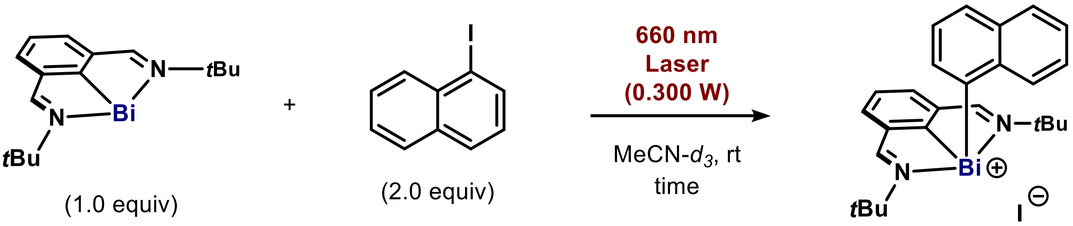

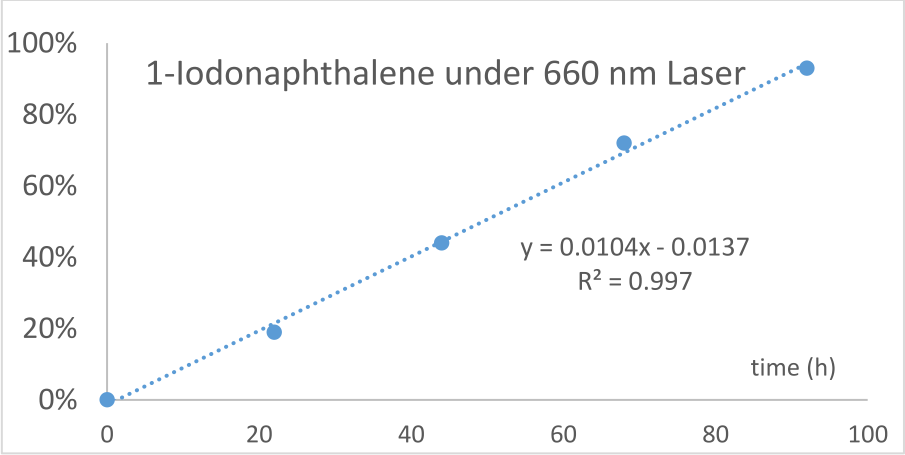

Figure S32. Reaction performed with 0.020 mmol of Bi(I) and 0.040 mmol of 1-iodonaphthalene in 0.6 mL of MeCN-d_3, in an NMR tube (0.033 M). Yields determined by ^{1}H NMR using 1,3,5-trimethoxybenzene as internal standard. Rate constant in yield/h.

The power of the light can be easily regulated between 0% (0 W), 25% (20 W), 50% (40 W), 75% (60 W) or 100% (80 W) using Kessil LED lamps. A positive relationship was found between light intensity and the rate of the reaction between bismuth(I) and 4-iodobenzonitrile. Rate constants in yield/h.

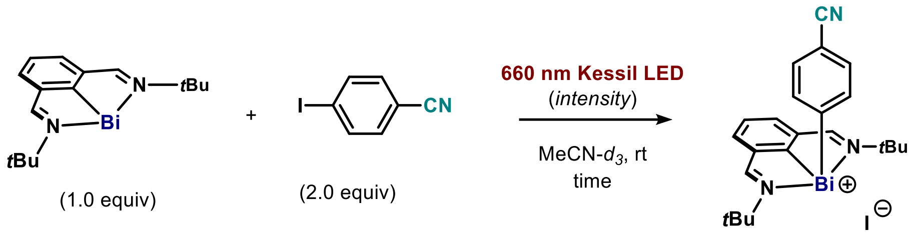

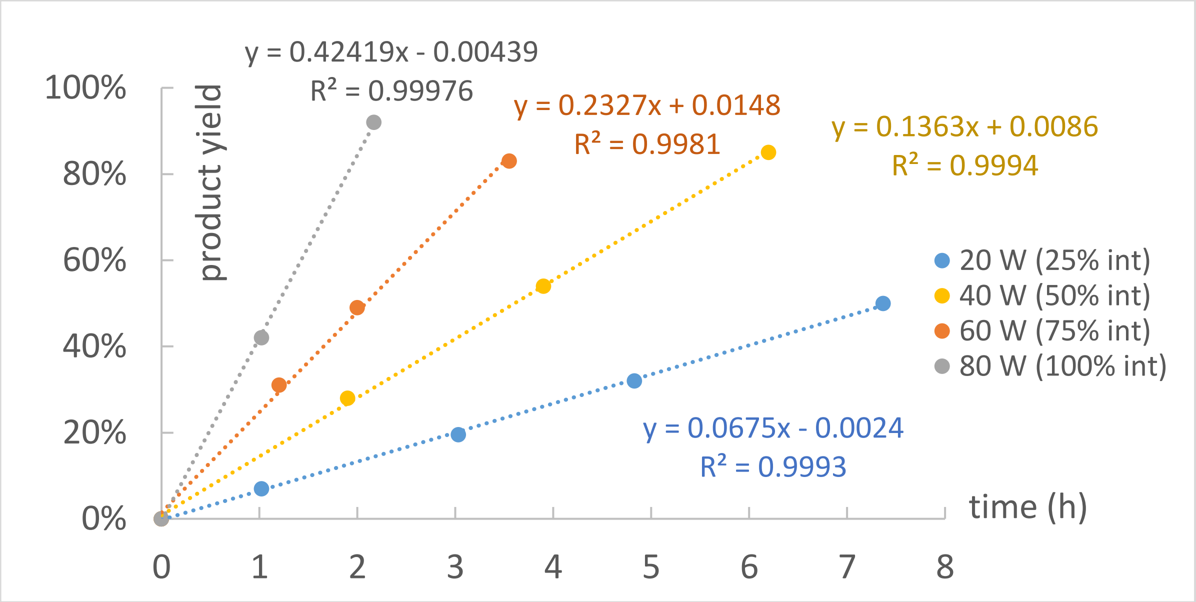

Figure S33. Reactions performed with 0.010 mmol of Bi(I) and 0.020 mmol of 4-iodobenzonitrile in 0.6 mL of MeCN-d_3 (0.0166 M), in an NMR tube. Yields determined by ^{1}H NMR using 1,3,5-trimethoxybenzene as internal standard. Rate constants in M/h.

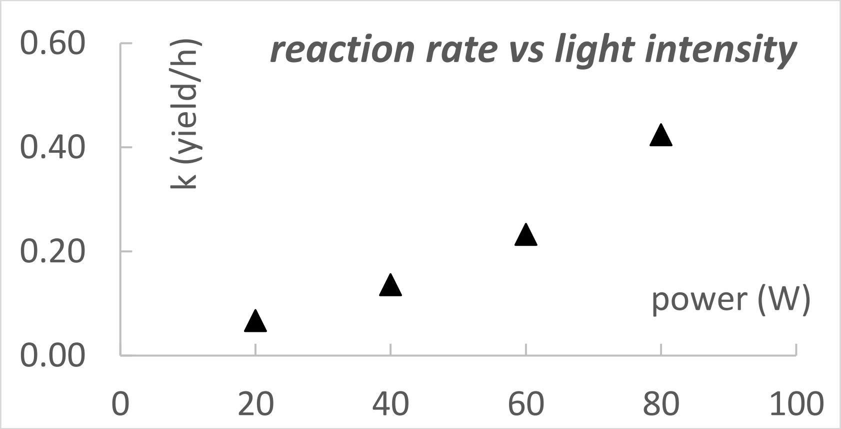

Figure S34. Relationship obtained when plotting the rate constants (yield/h) vs light power (W)

It should be noted that when scaling up any of these reactions, the total reaction time to reach full conversion scales up proportionally, under the same irradiation conditions. This is to be expected for zero-order reactions for which rate is dependent only on the number of photons absorbed.

10.2. On/off experiments with monochromatic 660 nm light

An on/off experiment with monochromatic 660 nm light was performed. The reaction did not proceed at all in the periods without light irradiation, and it readily proceeded upon laser-light irradiation, following the same zero-order profile.

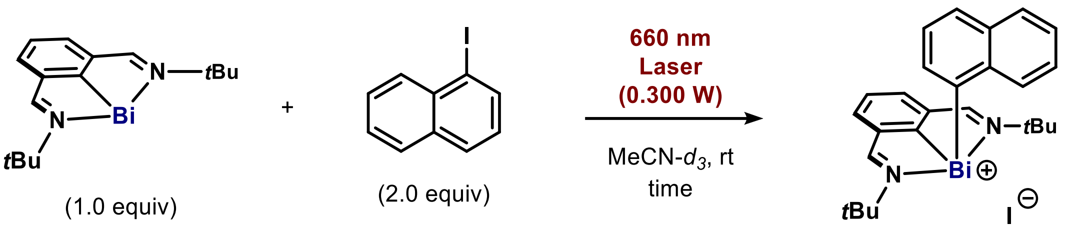

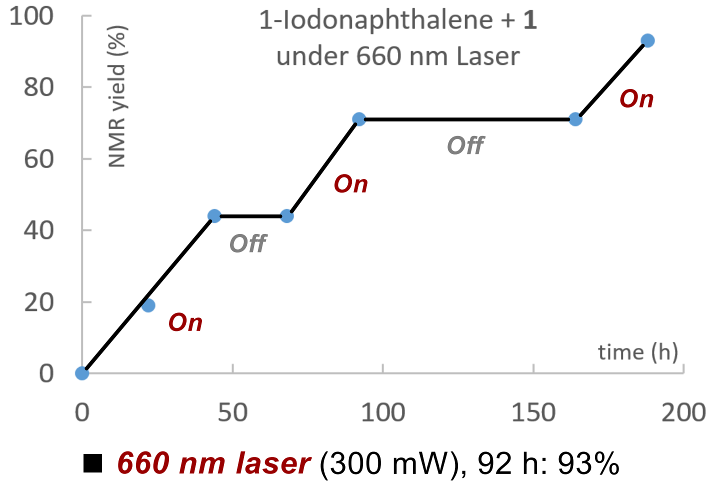

Figure S35. Reaction performed with 0.020 mmol of Bi(I) and 0.040 mmol of 1-iodonaphthalene in 0.6 mL of MeCN-d_3 (0.033 M), in an NMR tube. Yields determined by ^{1}H NMR using 1,3,5-trimethoxybenzene as internal standard.

10.3. Hammett correlation in the oxidative addition of aryl iodides

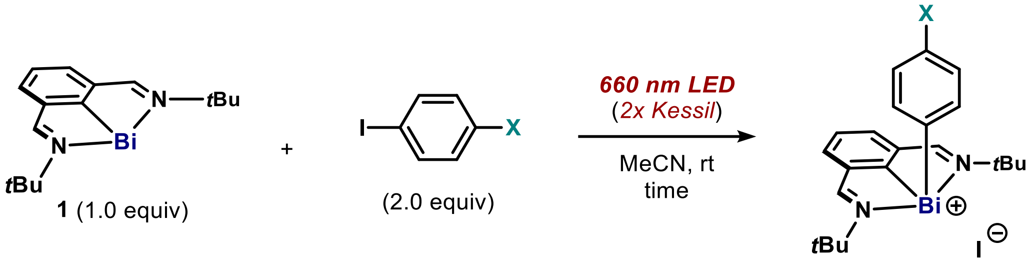

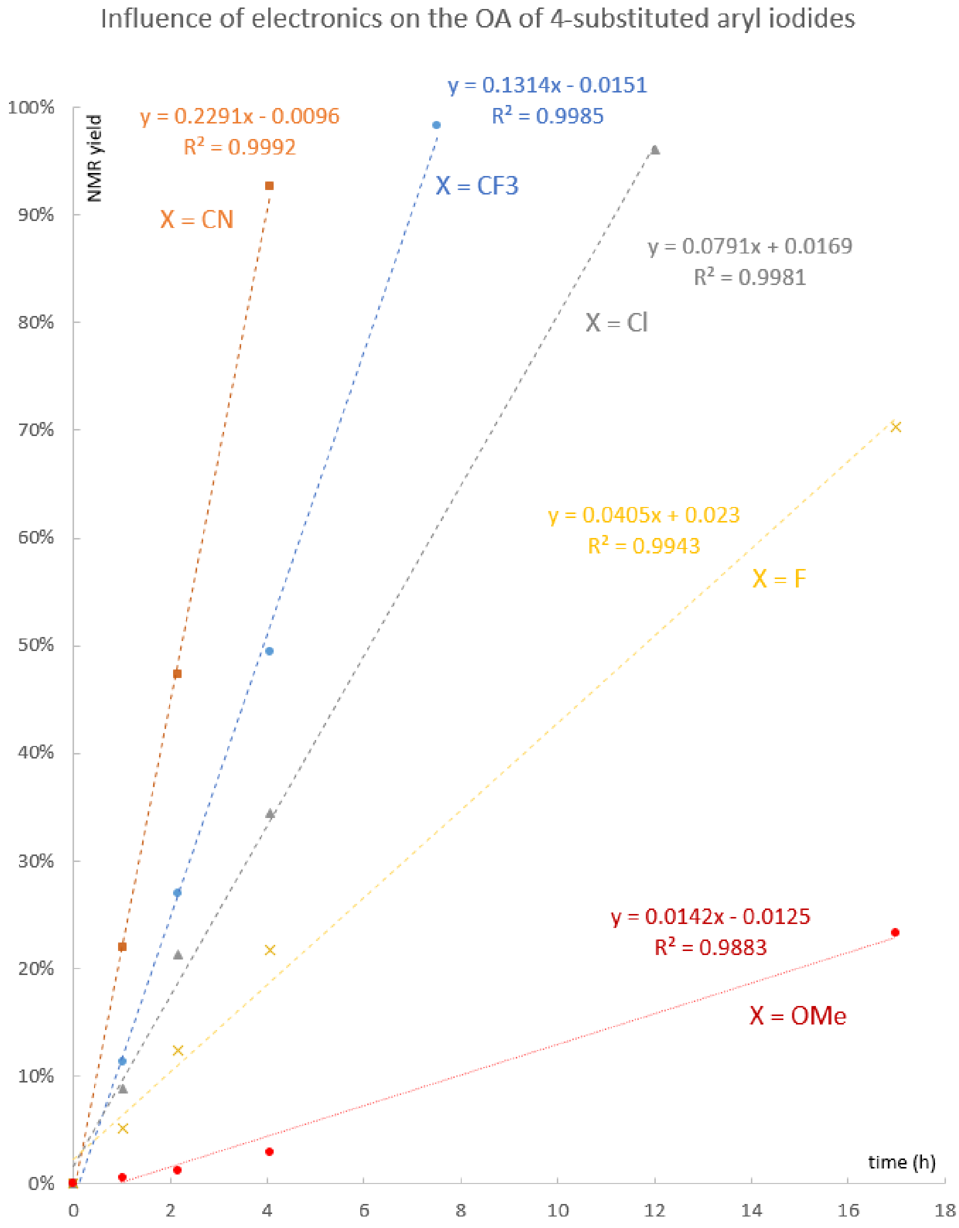

Figure S36. Reactions performed with 0.020 mmol of Bi(I) and 0.040 mmol of electrophile in 0.6 mL of MeCN-d_3 (0.033 M) in an NMR tube. Yields determined by ^{1}H NMR using 1,3,5-trimethoxybenzene as internal standard. Rates in yield/h.

A clear inverse fitting against Hammett σ_p is obtained. Reaction rate decreases as electron density on the aryl ring increases.[35]

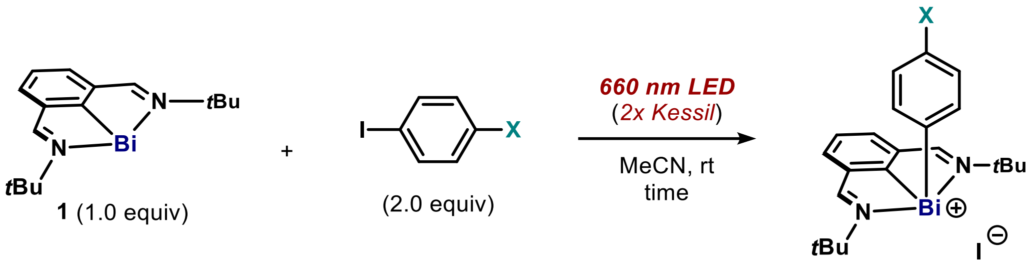

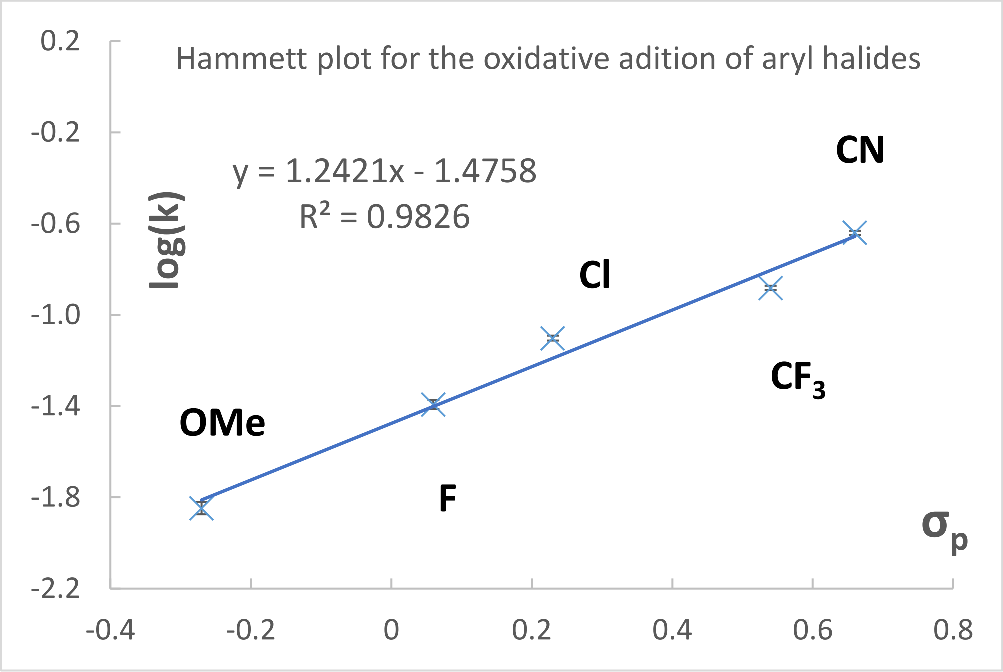

Figure S37. Hammett plot. The x-error was extracted from the fittings shown in Figure S33 using MS Excel linest function. The uncertainty after the log transformation was calculated as dF = 1/ln(10) * dx/x, and it was found to be very small (barely visible error bars).

10.4. Using all visible-light spectrum for the OA of aryl thianthrenium

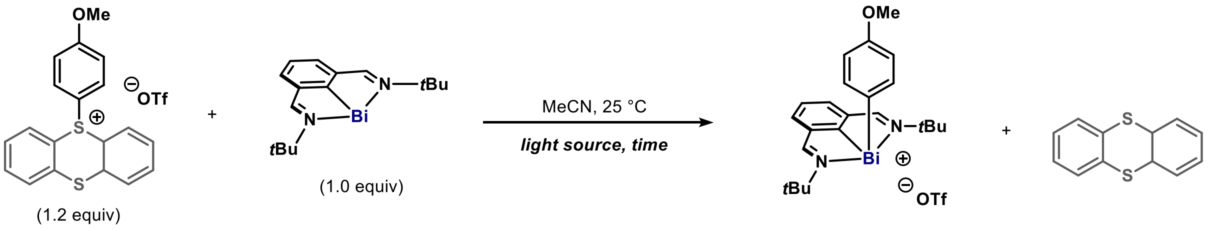

Following General Procedure C2, at 0.030 mmol scale in 0.6 mL of MeCN-d_3 and in the presence of 1.0 equiv of 1,3,5-trimethoxybenzene as internal standard, several light sources were employed for the fast oxidative addition of an aryl-thianthrenium salt. Since the mixtures only need to be exposed to light for a short period of time, no significant decomposition was observed with more energetic light sources.

light source	*time for full conv. of Bi(I) (judged by color change)*	*NMR yield of OA*	*NMR yield of TT*
blue LED strip (465 nm)	*<5 min*	*90%*	
white LED strip	*<5 min*	*79%*	
green LED strip (550 nm)	*<5 min*	*82%*	
red LED strip (650 nm)	*< 5 min*	*90%*	*91%*
red Kessil lamps (660 nm)	*3 min*	*93%*	*>95%*
dark (>90% Bi(I) recovery)	*4 days*	*<5%*	*<5%*
then, green LED	*<5 min*	*88%*	*92%*
with constant irradiance (Penn PhD Photoreactor M2, 50% irradiance)			
395 nm LED	*60 sec*	*85%*	
420 nm LED	*60 sec*	*89%*	
450 nm LED	*30 sec*	*92%*	

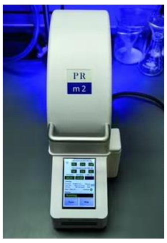

Control experiments under thermal conditions at high temperatures unequivocally highlight the influence of the light in the oxidative addition process.

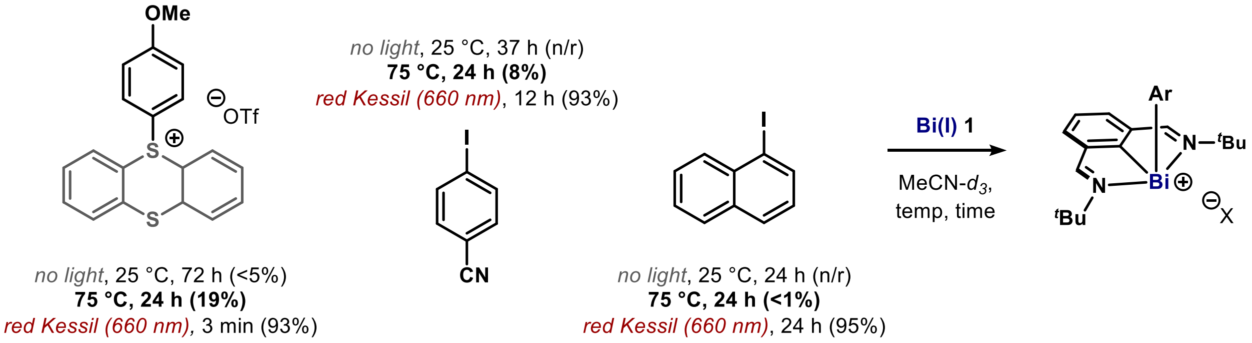

11. Crystal data and structure refinement

[(2,6-(tBuNCH)$_2$C$_6$H$_3$)Bi(4-methyltosylphenyl)-(tetrafluoroborate)] (**9**)

Single crystals of **9** were obtained by vapor diffusion of methyl tert-butyl ether (anti-solvent) into a solution of **9** in 1,2-dichloroethane at room temperature over 3 days, protected from light. Alternatively, vapor diffusion of diethyl ether into a solution of **9** in acetonitrile under the same conditions also afforded single-crystal x-ray quality material. CCDC deposit number: **2258383**.

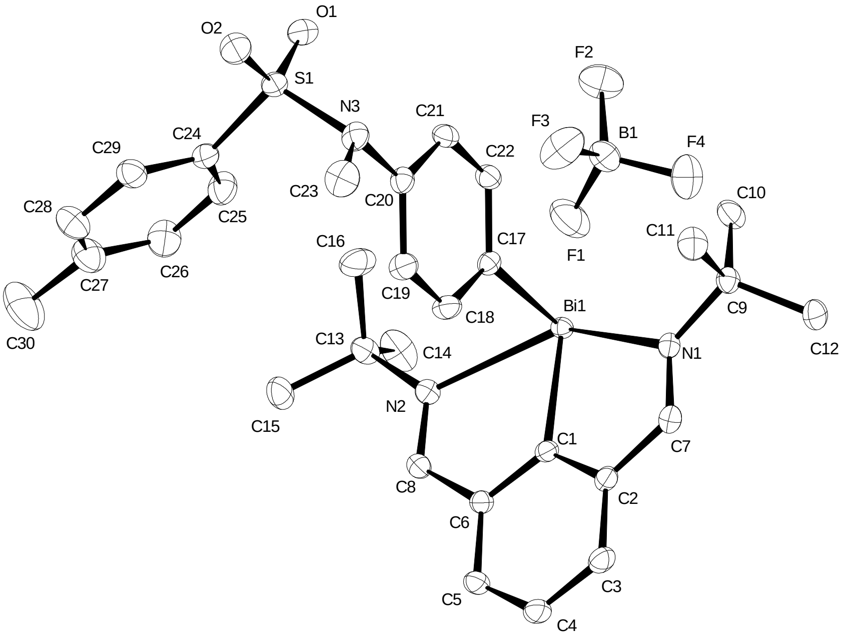

Figure S38. The molecular structure of **9**. H atoms have been removed for clarity.

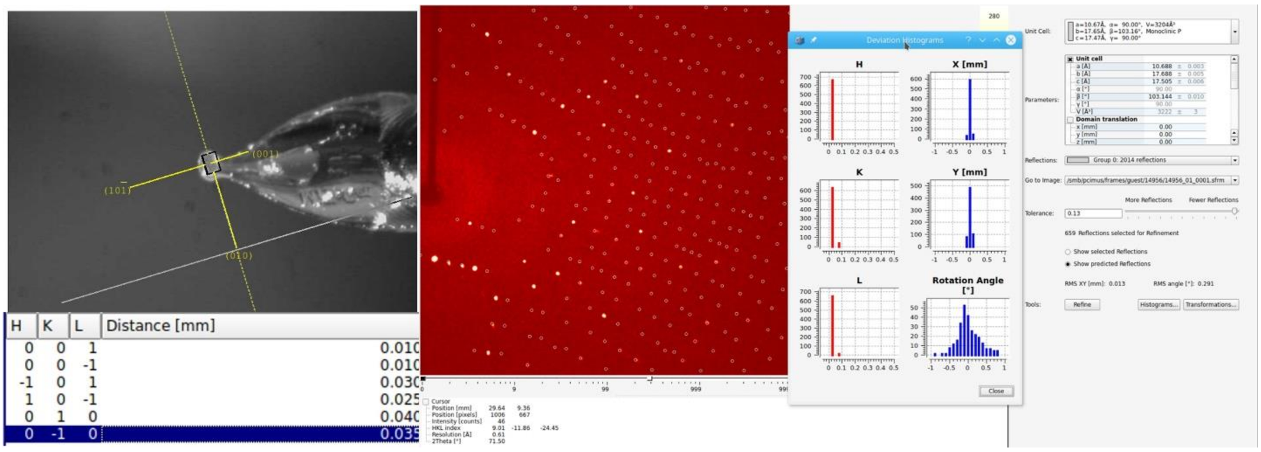

Figure S39. Crystal faces and unit cell determination/refinement of **9**

INTENSITY STATISTICS FOR DATASET # 1 14956sadabs.raw

Resolution	#Data	#Theory	%Complete	Redundancy	Mean I	Mean I/s	Rmerge	Rsigma
Inf - 2.71	184	184	100.0	16.71	157.03	114.97	0.0199	0.0066
2.71 - 1.79	432	432	100.0	18.03	98.71	111.07	0.0192	0.0068
1.79 - 1.42	606	606	100.0	18.22	71.29	93.99	0.0215	0.0075
1.42 - 1.23	634	634	100.0	18.01	56.83	84.37	0.0242	0.0083
1.23 - 1.12	591	591	100.0	17.33	45.16	74.65	0.0282	0.0094
1.12 - 1.03	679	679	100.0	13.52	38.77	57.29	0.0325	0.0122
1.03 - 0.97	598	598	100.0	10.81	33.75	46.44	0.0366	0.0153
0.97 - 0.92	644	644	100.0	9.18	30.09	39.49	0.0407	0.0184
0.92 - 0.88	599	599	100.0	8.35	24.46	32.38	0.0483	0.0223
0.88 - 0.85	558	558	100.0	7.64	24.66	30.94	0.0492	0.0242
0.85 - 0.82	608	608	100.0	7.59	19.87	26.12	0.0554	0.0282
0.82 - 0.79	721	721	100.0	7.13	19.97	24.84	0.0577	0.0307
0.79 - 0.77	539	539	100.0	6.98	17.73	22.21	0.0647	0.0347
0.77 - 0.75	604	604	100.0	6.78	15.71	19.75	0.0708	0.0390
0.75 - 0.73	673	673	100.0	6.48	15.88	18.74	0.0715	0.0413
0.73 - 0.71	747	747	100.0	6.39	14.26	17.18	0.0803	0.0464
0.71 - 0.70	389	389	100.0	5.99	13.37	15.36	0.0853	0.0516
0.70 - 0.68	892	892	100.0	5.91	11.55	13.62	0.0976	0.0597
0.68 - 0.67	468	468	100.0	5.84	10.94	13.10	0.1019	0.0632
0.67 - 0.66	507	507	100.0	5.59	10.72	12.23	0.1085	0.0681
0.66 - 0.65	521	553	94.2	4.54	9.53	10.37	0.1171	0.0849
0.75 - 0.65	4197	4229	99.2	5.87	12.48	14.60	0.0899	0.0560
Inf - 0.65	12194	12226	99.7	9.55	30.31	38.66	0.0326	0.0201

Merged [A], lowest resolution = 17.48 Angstroms

Complete .cif-data of the compound are available under the CCDC number CCDC-**2258383**.

Table S2. Crystal data and structure refinement of compound **[(2,6-(tBuNCH)$_2$C$_6$H$_3$)Bi(4-methyltosylphenyl)(tetrafluoroborate)]**.

Identification code	14956	
Empirical formula	$C_{30} H_{37} B Bi F_4 N_3 O_2 S$	
Color	colourless	
Formula weight	799.47 g·mol^{-1}	
Temperature	100(2) K	
Wavelength	0.71073 Å	
Crystal system	MONOCLINIC	
Space group	**P2$_1$/n, (no. 14)**	
Unit cell dimensions	a = 10.5738(9) Å	□= 90°.
	b = 17.4819(15) Å	□= 103.155(3)°.
	c = 17.3149(14) Å	□ = 90°.
Volume	3116.7(5) Å^3	
Z	4	
Density (calculated)	1.704 Mg · m^{-3}	
Absorption coefficient	5.780 mm^{-1}	
F(000)	1576 e	
Crystal size	0.081 x 0.073 x 0.024 mm^3	
□ range for data collection	1.678 to 33.140°.	
Index ranges	-16 □ h □ 16, -26□ k □ 26, -26 □ l □ 26	
Reflections collected	114829	
Independent reflections	11848 [R_{int} = 0.0329]	
Reflections with I>2□(I)	10213	
Completeness to □ = 25.242°	100.0 %	
Absorption correction	Gaussian	
Max. and min. transmission	0.90 and 0.67	
Refinement method	Full-matrix least-squares on F^2	
Data / restraints / parameters	11848 / 0 / 387	
Goodness-of-fit on F^2	1.026	
Final R indices [I>2□(I)]	R_1 = 0.0204	wR^2 = 0.0454
R indices (all data)	R_1 = 0.0282	wR^2 = 0.0478
Largest diff. peak and hole	2.4 and -0.8 e · Å^{-3}	

Table S3. Bond lengths [Å] and angles [°] of compound **[(2,6-(tBuNCH)$_2$C$_6$H$_3$)Bi(4-methyltosylphenyl)(tetrafluoroborate)]**.

	Bi(1)-N(1)	2.4681(14)	Bi(1)-N(2)
2.5326(15)	Bi(1)-C(1)	2.1988(17)	Bi(1)-C(17)
2.2359(17)	S(1)-O(1)	1.4299(17)	S(1)-O(2)
1.4330(15)	S(1)-N(3)	1.6564(17)	S(1)-C(24)
1.7599(19)	N(1)-C(7)	1.279(2)	N(1)-C(9)
1.496(2)	N(2)-C(8)	1.274(2)	N(2)-C(13)
1.485(2)	N(3)-C(20)	1.444(2)	N(3)-C(23)
1.479(3)	C(1)-C(2)	1.386(2)	C(1)-C(6)
1.392(2)	C(2)-C(3)	1.400(3)	C(2)-C(7)
1.470(3)	C(3)-C(4)	1.393(3)	C(4)-C(5)
1.390(3)	C(5)-C(6)	1.393(3)	C(6)-C(8)
1.468(2)	C(9)-C(10)	1.522(3)	C(9)-C(11)
1.532(3)	C(9)-C(12)	1.529(3)	C(13)-
C(14)	1.511(3)	C(13)-C(15)	1.532(3)
C(13)-C(16)	1.524(3)	C(17)-C(18)	1.392(3)
C(17)-C(22)	1.392(2)	C(18)-C(19)	1.393(3)
C(19)-C(20)	1.390(3)	C(20)-C(21)	1.387(3)
C(21)-C(22)	1.390(3)	C(24)-C(25)	1.397(3)
C(24)-C(29)	1.378(3)	C(25)-C(26)	1.382(3)
C(26)-C(27)	1.397(3)	C(27)-C(28)	1.380(3)
C(27)-C(30)	1.503(3)	C(28)-C(29)	1.390(3)
F(1)-B(1)	1.388(3)	F(2)-B(1)	1.388(3)
F(3)-B(1)	1.388(3)	F(4)-B(1)	1.390(3)
N(1)-Bi(1)-N(2)	140.62(5)	C(1)-Bi(1)-N(1)	71.53(6)
C(1)-Bi(1)-N(2)	70.32(6)	C(1)-Bi(1)-C(17)	94.22(6)
C(17)-Bi(1)-N(1)	85.57(5)	C(17)-Bi(1)-N(2)	87.80(5)
O(1)-S(1)-O(2)	119.93(10)	O(1)-S(1)-N(3)	107.75(9)
O(1)-S(1)-C(24)	106.54(10)	O(2)-S(1)-N(3)	105.70(9)
O(2)-S(1)-C(24)	108.56(9)	N(3)-S(1)-C(24)	107.88(9)
C(7)-N(1)-Bi(1)	112.33(11)	C(7)-N(1)-C(9)	122.22(15)
C(9)-N(1)-Bi(1)	125.44(11)	C(8)-N(2)-Bi(1)	112.14(11)
C(8)-N(2)-C(13)	122.41(15)	C(13)-N(2)-Bi(1)	125.45(11)
C(20)-N(3)-S(1)	114.95(13)	C(20)-N(3)-C(23)	117.32(16)
C(23)-N(3)-S(1)	115.17(14)	C(2)-C(1)-Bi(1)	118.62(13)
C(2)-C(1)-C(6)	121.10(16)	C(6)-C(1)-Bi(1)	120.14(12)
C(1)-C(2)-C(3)	119.28(17)	C(1)-C(2)-C(7)	117.30(16)
C(3)-C(2)-C(7)	123.39(16)	C(4)-C(3)-C(2)	119.47(17)
C(5)-C(4)-C(3)	121.08(17)	C(4)-C(5)-C(6)	119.24(17)
C(1)-C(6)-C(5)	119.79(16)	C(1)-C(6)-C(8)	117.44(16)
C(5)-C(6)-C(8)	122.74(16)	N(1)-C(7)-C(2)	119.45(16)
N(2)-C(8)-C(6)	119.41(16)	N(1)-C(9)-C(10)	107.22(14)
N(1)-C(9)-C(11)	107.09(15)	N(1)-C(9)-C(12)	111.98(15)
C(10)-C(9)-C(11)	110.18(16)	C(10)-C(9)-C(12)	109.81(16)
C(12)-C(9)-C(11)	110.48(15)	N(2)-C(13)-C(14)	107.31(17)
N(2)-C(13)-C(15)	112.92(16)	N(2)-C(13)-C(16)	105.97(14)
C(14)-C(13)-C(15)	110.18(17)	C(14)-C(13)-C(16)	110.64(19)
C(16)-C(13)-C(15)	109.72(18)	C(18)-C(17)-Bi(1)	120.98(13)

C(22)-C(17)-Bi(1)	119.72(13)	C(22)-C(17)-C(18)	119.26(16)
C(17)-C(18)-C(19)	120.55(17)	C(20)-C(19)-C(18)	119.78(18)
C(19)-C(20)-N(3)	120.73(17)	C(21)-C(20)-N(3)	119.40(17)
C(21)-C(20)-C(19)	119.86(17)	C(20)-C(21)-C(22)	120.27(17)
C(21)-C(22)-C(17)	120.27(17)	C(25)-C(24)-S(1)	119.53(15)
C(29)-C(24)-S(1)	119.47(15)	C(29)-C(24)-C(25)	120.72(18)
C(26)-C(25)-C(24)	119.0(2)	C(25)-C(26)-C(27)	120.7(2)
C(26)-C(27)-C(30)	120.4(2)	C(28)-C(27)-C(26)	119.3(2)
C(28)-C(27)-C(30)	120.3(2)	C(27)-C(28)-C(29)	120.6(2)
C(24)-C(29)-C(28)	119.6(2)	F(1)-B(1)-F(2)	109.36(18)
F(1)-B(1)-F(3)	110.30(19)	F(1)-B(1)-F(4)	108.7(2)
F(2)-B(1)-F(4)	110.19(18)	F(3)-B(1)-F(2)	109.21(19)
F(3)-B(1)-F(4)	109.09(18)		

[(2,6-(tBuNCH)$_2$C$_6$H$_3$)Bi(4-cyanophenyl)(iodide)] (12a)

Single crystals of **12a** were obtained by vapor diffusion of methyl tert-butyl ether (anti-solvent) into a solution of **12a** in 1,2-dichloroethane at room temperature over 3 days, protected from light. CCDC deposit number: **2258382**.

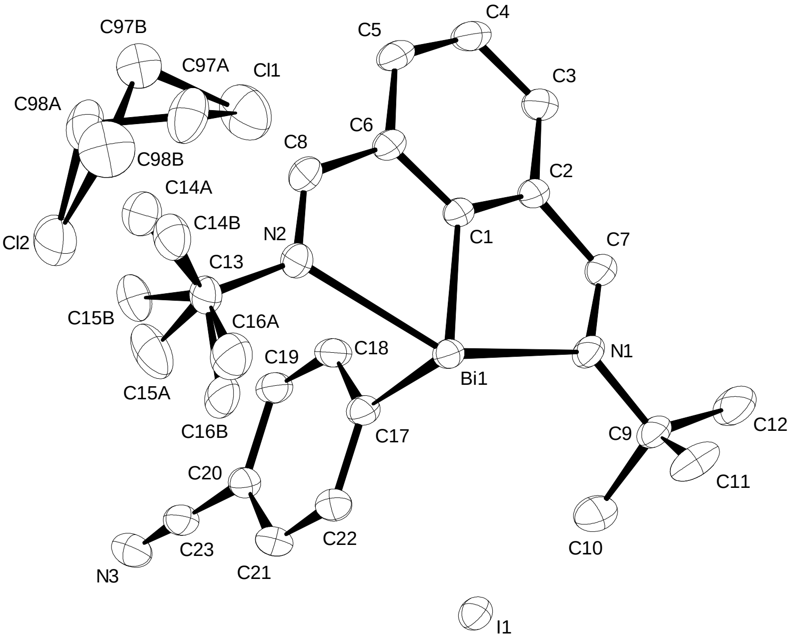

Figure S40. The molecular structure of 12a. H atoms have been removed for clarity.

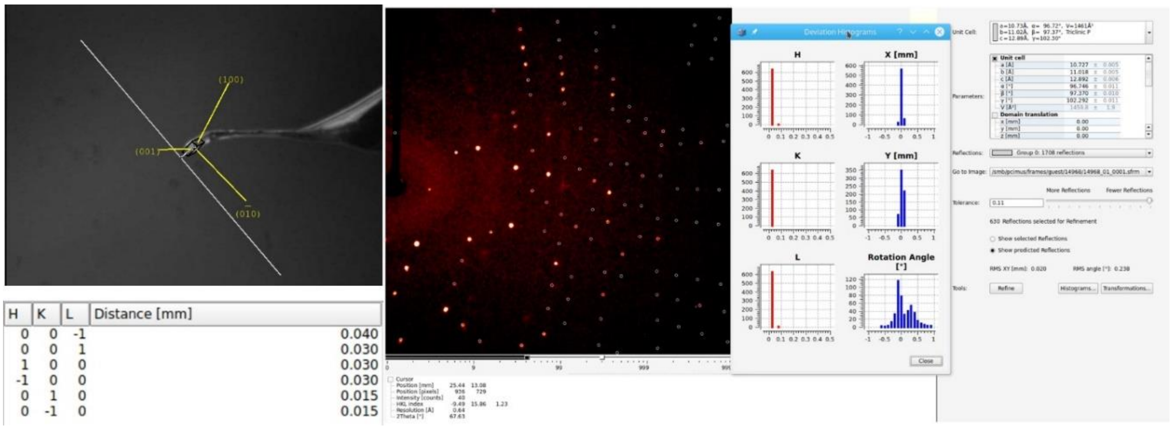

Figure S41. Crystal faces and unit cell determination/refinement of compound **12**

INTENSITY STATISTICS FOR DATASET # 1 14968sadabs.raw

Resolution	#Data	#Theory	%Complete	Redundancy	Mean I	Mean I/s	Rmerge	Rsigma
Inf - 2.58	165	165	100.0	12.12	124.49	73.95	0.0301	0.0123
2.58 - 1.74	385	385	100.0	14.18	80.21	74.07	0.0267	0.0116
1.74 - 1.39	533	533	100.0	14.35	51.43	68.91	0.0275	0.0121
1.39 - 1.21	562	562	100.0	14.08	38.62	62.05	0.0289	0.0128
1.21 - 1.10	538	538	100.0	13.33	29.23	55.80	0.0343	0.0140
1.10 - 1.02	572	572	100.0	10.80	22.04	44.45	0.0410	0.0174
1.02 - 0.96	538	538	100.0	9.42	19.49	38.77	0.0449	0.0201
0.96 - 0.91	574	574	100.0	8.60	16.77	34.12	0.0490	0.0227
0.91 - 0.87	561	561	100.0	8.09	14.07	30.08	0.0549	0.0260
0.87 - 0.84	478	478	100.0	7.70	11.77	26.74	0.0611	0.0294
0.84 - 0.81	583	583	100.0	7.42	10.02	22.93	0.0689	0.0342
0.81 - 0.78	650	650	100.0	7.18	8.87	21.31	0.0761	0.0386
0.78 - 0.76	506	506	100.0	6.91	8.55	19.58	0.0835	0.0423
0.76 - 0.74	554	554	100.0	6.82	7.76	18.14	0.0917	0.0465
0.74 - 0.72	612	612	100.0	6.38	6.46	15.19	0.1038	0.0567
0.72 - 0.71	333	333	100.0	6.47	5.86	14.51	0.1134	0.0609
0.71 - 0.69	759	760	99.9	6.18	5.38	12.91	0.1247	0.0685
0.69 - 0.68	384	384	100.0	6.02	3.96	9.92	0.1487	0.0910
0.68 - 0.66	847	852	99.4	5.85	3.96	9.88	0.1515	0.0949
0.66 - 0.65	478	481	99.4	5.46	3.44	8.27	0.1656	0.1126
0.65 - 0.64	177	316	56.0	1.31	3.60	5.67	0.1462	0.1736
0.74 - 0.64	3590	3738	96.0	5.64	4.78	11.44	0.1294	0.0802
Inf - 0.64	10789	10937	98.6	8.41	18.53	30.23	0.0403	0.0232

Complete .cif-data of the compound are available under the CCDC number CCDC-**2258382**.

Table S4. Crystal data and structure refinement of compound **[(2,6-(tBuNCH)$_2$C$_6$H$_3$)Bi(4-cyanophenyl)(iodide)]**.

Identification code	14968	
Empirical formula	$C_{25} H_{31} Bi Cl_2 I N_3$	
Color	yellow	
Formula weight	780.31 g · mol^{-1}	
Temperature	100(2) K	
Wavelength	0.71073 Å	
Crystal system	TRICLINIC	
Space group	**P$\bar{1}$, (no. 2)**	
Unit cell dimensions	a = 10.5537(5) Å	□= 96.767(2)°.
	b = 10.8425(5) Å	□= 97.394(2)°.
	c = 12.6967(7) Å	□ = 102.234(2)°.
Volume	1392.19(12) Å^3	
Z	2	
Density (calculated)	1.861 Mg · m^{-3}	
Absorption coefficient	7.650 mm^{-1}	
F(000)	744 e	
Crystal size	0.091 x 0.071 x 0.032 mm^3	
□ range for data collection	1.636 to 33.505°.	
Index ranges	-16 □ h □ 16, -16□ k □ 16, -19 □ l □ 19	
Reflections collected	91928	
Independent reflections	10787 [R_{int} = 0.0406]	
Reflections with I>2□(I)	9676	
Completeness to □ = 25.242°	100.0 %	
Absorption correction	Gaussian	
Max. and min. transmission	0.67 and 0.37	
Refinement method	Full-matrix least-squares on F^2	
Data / restraints / parameters	10787 / 0 / 333	
Goodness-of-fit on F^2	1.029	
Final R indices [I>2□(I)]	R_1 = 0.0255	wR^2 = 0.0590
R indices (all data)	R_1 = 0.0317	wR^2 = 0.0611
Largest diff. peak and hole	1.8 and -0.9 e · Å^{-3}	

Table S5. Bond lengths [Å] and angles [°] of compound **[(2,6-(tBuNCH)$_2$C$_6$H$_3$)Bi(4-cyanophenyl)(iodide)]**.

Bi(1)-N(1)	2.497(2)	Bi(1)-N(2)	2.558(2)
Bi(1)-C(1)	2.195(2)	Bi(1)-C(17)	2.258(3)
N(1)-C(7)	1.275(3)	N(1)-C(9)	1.483(3)
N(2)-C(8)	1.274(3)	N(2)-C(13)	1.494(3)
N(3)-C(23)	1.137(4)	C(1)-C(2)	1.385(3)
C(1)-C(6)	1.385(3)	C(2)-C(3)	1.392(3)
C(2)-C(7)	1.462(3)	C(3)-C(4)	1.391(4)
C(4)-C(5)	1.395(4)	C(5)-C(6)	1.395(4)
C(6)-C(8)	1.467(4)	C(9)-C(10)	1.525(4)
C(9)-C(11)	1.526(5)	C(9)-C(12)	1.513(4)
C(13)-C(14A)	1.547(6)	C(13)-C(14B)	1.465(9)
C(13)-C(15A)	1.520(7)	C(13)-C(15B)	1.539(8)
C(13)-C(16A)	1.497(7)	C(13)-C(16B)	1.526(9)
C(17)-C(18)	1.396(4)	C(17)-C(22)	1.396(3)
C(18)-C(19)	1.376(4)	C(19)-C(20)	1.398(3)
C(20)-C(21)	1.392(4)	C(20)-C(23)	1.441(4)
C(21)-C(22)	1.378(4)	Cl(1)-C(97A)	1.756(6)
Cl(1)-C(97B)	1.879(9)	Cl(2)-C(98A)	1.799(6)
Cl(2)-C(98B)	1.828(15)	C(97A)-C(98A)	1.462(8)
C(97B)-C(98B)	1.566(17)		
N(1)-Bi(1)-N(2)	140.25(7)	C(1)-Bi(1)-N(1)	71.56(8)
C(1)-Bi(1)-N(2)	70.42(8)	C(1)-Bi(1)-C(17)	92.02(9)
C(17)-Bi(1)-N(1)	87.02(8)	C(17)-Bi(1)-N(2)	83.16(8)
C(7)-N(1)-Bi(1)	111.44(15)	C(7)-N(1)-C(9)	122.0(2)
C(9)-N(1)-Bi(1)	126.44(16)	C(8)-N(2)-Bi(1)	110.64(16)
C(8)-N(2)-C(13)	120.7(2)	C(13)-N(2)-Bi(1)	128.65(16)
C(2)-C(1)-Bi(1)	118.78(16)	C(2)-C(1)-C(6)	121.1(2)
C(6)-C(1)-Bi(1)	120.10(18)	C(1)-C(2)-C(3)	119.6(2)
C(1)-C(2)-C(7)	118.0(2)	C(3)-C(2)-C(7)	122.3(2)
C(4)-C(3)-C(2)	119.6(2)	C(3)-C(4)-C(5)	120.5(2)
C(4)-C(5)-C(6)	119.5(2)	C(1)-C(6)-C(5)	119.6(2)
C(1)-C(6)-C(8)	117.9(2)	C(5)-C(6)-C(8)	122.1(2)
N(1)-C(7)-C(2)	120.1(2)	N(2)-C(8)-C(6)	120.0(2)
N(1)-C(9)-C(10)	107.6(2)	N(1)-C(9)-C(11)	106.3(2)
N(1)-C(9)-C(12)	113.1(2)	C(10)-C(9)-C(11)	109.4(3)
C(12)-C(9)-C(10)	110.7(3)	C(12)-C(9)-C(11)	109.7(3)
N(2)-C(13)-C(14A)	113.1(3)	N(2)-C(13)-C(15A)	108.8(3)
N(2)-C(13)-C(15B)	105.5(4)	N(2)-C(13)-C(16A)	106.9(3)
N(2)-C(13)-C(16B)	107.3(3)	C(14B)-C(13)-N(2)	113.5(4)
C(14B)-C(13)-C(15B)	112.7(5)	C(14B)-C(13)-C(16B)	109.8(6)
C(15A)-C(13)-C(14A)	109.6(4)	C(16A)-C(13)-C(14A)	107.4(4)
C(16A)-C(13)-C(15A)	111.0(5)	C(16B)-C(13)-C(15B)	107.8(6)
C(18)-C(17)-Bi(1)	122.62(18)	C(22)-C(17)-Bi(1)	118.29(19)
C(22)-C(17)-C(18)	118.9(2)	C(19)-C(18)-C(17)	120.9(2)
C(18)-C(19)-C(20)	119.2(2)	C(19)-C(20)-C(23)	119.9(2)
C(21)-C(20)-C(19)	120.8(3)	C(21)-C(20)-C(23)	119.3(2)
C(22)-C(21)-C(20)	119.1(2)	C(21)-C(22)-C(17)	121.1(3)
N(3)-C(23)-C(20)	179.4(3)	C(98A)-C(97A)-Cl(1)	112.4(5)
C(98B)-C(97B)-Cl(1)	105.5(7)	C(97A)-C(98A)-Cl(2)	110.2(4)
C(97B)-C(98B)-Cl(2)	106.5(9)		

[(2,6-(tBuNCH)$_2$C$_6$H$_3$)BiI$_2$] (S6)

Single crystals of **S6** (a decomposition product of **12c**) were obtained by vapor diffusion of methyl tert-butyl ether (anti-solvent) into a solution of **12c** in 1,2-dichloroethane at room temperature over 7 days under air. CCDC deposit number: **2259026**.

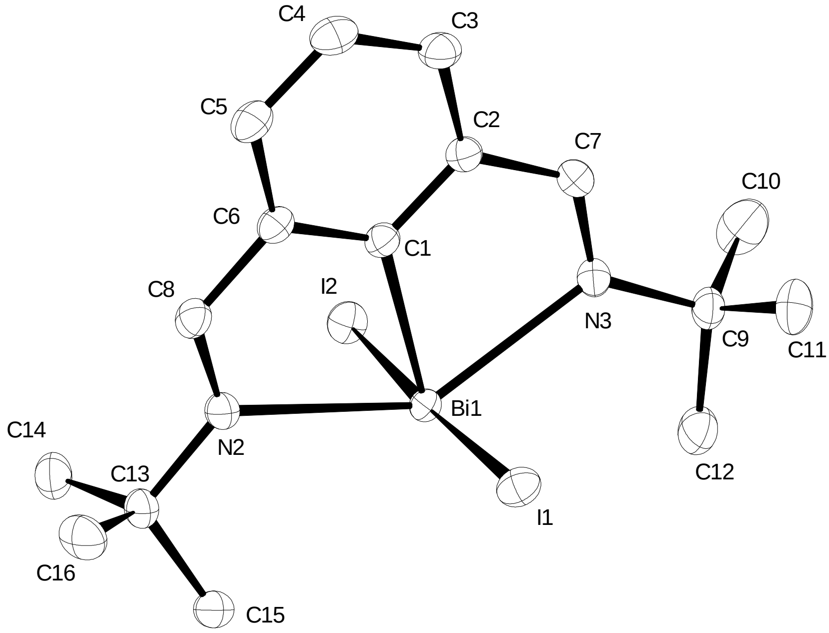

Figure S42. The molecular structure of **[(2,6-(tBuNCH)$_2$C$_6$H$_3$)BiI$_2$]**. H atoms have been removed for clarity.

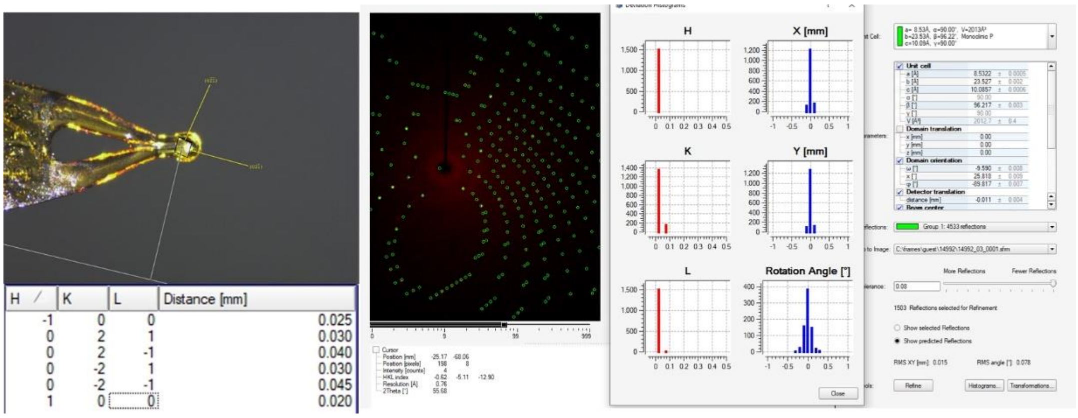

Figure S43. Crystal faces and unit cell determination/refinement of compound **[(2,6-(tBuNCH)$_2$C$_6$H$_3$)BiI$_2$]**.

INTENSITY STATISTICS FOR DATASET # 1 14992sadabs.raw

Resolution	#Data	#Theory	%Comp	Redundancy	Rrim	Rpim
Inf - 2.57	136	138	98.55	30.72	0.0339	0.0099
2.57 - 1.69	323	323	100.00	40.33	0.0307	0.0066
1.69 - 1.34	461	461	100.00	49.82	0.0316	0.0049
1.34 - 1.17	450	450	100.00	41.59	0.0347	0.0056
1.17 - 1.06	450	450	100.00	37.80	0.0410	0.0067
1.06 - 0.98	481	481	100.00	35.81	0.0464	0.0078
0.98 - 0.92	470	470	100.00	34.55	0.0492	0.0084
0.92 - 0.88	398	398	100.00	33.04	0.0563	0.0097
0.88 - 0.84	466	466	100.00	32.09	0.0611	0.0107
0.84 - 0.81	428	428	100.00	29.63	0.0712	0.0129
0.81 - 0.78	454	454	100.00	28.72	0.0749	0.0139
0.78 - 0.75	586	586	100.00	25.77	0.0728	0.0141
0.75 - 0.73	416	416	100.00	24.50	0.0835	0.0168
0.73 - 0.71	469	469	100.00	23.73	0.0847	0.0174
0.71 - 0.69	550	550	100.00	23.62	0.0878	0.0179
0.69 - 0.68	294	294	100.00	23.24	0.1013	0.0209
0.68 - 0.66	612	612	100.00	22.78	0.1077	0.0224
0.66 - 0.65	357	357	100.00	21.91	0.1208	0.0255
0.65 - 0.64	374	374	100.00	21.86	0.1190	0.0252
0.64 - 0.62	846	867	97.58	19.47	0.1463	0.0319
0.72 - 0.62	3268	3289	99.36	21.94	0.1084	0.0228
Inf - 0.62	9021	9044	99.75	29.44	0.0502	0.0092

Complete .cif-data of the compound are available under the CCDC number CCDC-**2259026**.

Table S6. Crystal data and structure refinement of compound [(2,6-(tBuNCH)$_2$C$_6$H$_3$)BiI$_2$]

Identification code	14992	
Empirical formula	$C_{16} H_{23} Bi I_2 N_2$	
Color	clear yellow	
Formula weight	706.14 g · mol^{-1}	
Temperature	100(2) K	
Wavelength	0.71073 Å	
Crystal system	MONOCLINIC	
Space group	**P2$_1$/c, (no. 14)**	
Unit cell dimensions	a = 8.5112(2) Å	□= 90°.
	b = 23.4727(6) Å	□= 96.1440(10)°.
	c = 10.0672(2) Å	□ = 90°.
Volume	1999.68(8) Å^3	
Z	4	
Density (calculated)	2.346 Mg · m^{-3}	
Absorption coefficient	11.901 mm^{-1}	
F(000)	1288 e	
Crystal size	0.101 x 0.085 x 0.052 mm^3	
□ range for data collection	2.212 to 35.059°.	
Index ranges	-13 □ h □ 13, -37□ k □ 37, -16 □ l □ 16	
Reflections collected	262965	
Independent reflections	8828 [R_{int} = 0.0496]	
Reflections with I>2□(I)	8274	
Completeness to □ = 25.242°	100.0 %	
Absorption correction	Semi-empirical from equivalents	
Max. and min. transmission	0.66 and 0.45	
Refinement method	Full-matrix least-squares on F^2	
Data / restraints / parameters	8828 / 0 / 204	
Goodness-of-fit on F^2	1.083	
Final R indices [I>2□(I)]	R_1 = 0.0216	wR^2 = 0.0548
R indices (all data)	R_1 = 0.0239	wR^2 = 0.0558
Largest diff. peak and hole	2.1 and -1.2 e · Å^{-3}	

Table S7. Bond lengths [Å] and angles [°] of compound **[(2,6-(tBuNCH)$_2$C$_6$H$_3$)BiI$_2$]**

Bi(1)-I(1)	3.11214(19)	Bi(1)-I(2)	
2.98812(19)	Bi(1)-C(1)	2.212(2)	Bi(1)-N(3)
2.475(2)	Bi(1)-N(2)	2.520(2)	C(1)-C(2)
1.387(3)	C(1)-C(6)	1.391(3)	N(3)-C(7)
1.284(3)	N(3)-C(9)	1.487(3)	C(7)-C(2)
1.465(4)	C(7)-H(7)	0.83(4)	C(2)-C(3)
1.400(3)	C(6)-C(5)	1.401(3)	C(6)-C(8)
1.460(4)	C(5)-C(4)	1.389(4)	C(4)-C(3)
1.396(4)	C(8)-N(2)	1.280(3)	C(8)-H(8)
0.94(4)	N(2)-C(13)	1.490(3)	C(13)-
C(16)	1.532(4)	C(13)-C(14)	1.530(4)
C(13)-C(15)	1.527(4)	C(9)-C(11)	1.524(4)
C(9)-C(10)	1.517(4)	C(9)-C(12)	1.526(4)

I(2)-Bi(1)-I(1)	177.212(6)	C(1)-Bi(1)-I(1)	88.11(6)
C(1)-Bi(1)-I(2)	91.90(6)	C(1)-Bi(1)-N(3)	71.26(8)
C(1)-Bi(1)-N(2)	70.39(8)	N(3)-Bi(1)-I(1)	87.52(5)
N(3)-Bi(1)-I(2)	89.84(5)	N(3)-Bi(1)-N(2)	141.65(7)
N(2)-Bi(1)-I(1)	91.52(5)	N(2)-Bi(1)-I(2)	91.10(5)
C(2)-C(1)-Bi(1)	118.89(17)	C(2)-C(1)-C(6)	121.1(2)
C(6)-C(1)-Bi(1)	119.94(17)	C(7)-N(3)-Bi(1)	112.88(16)
C(7)-N(3)-C(9)	121.0(2)	C(9)-N(3)-Bi(1)	126.08(16)
N(3)-C(7)-C(2)	119.3(2)	N(3)-C(7)-H(7)	123(3)
C(2)-C(7)-H(7)	118(3)	C(1)-C(2)-C(7)	117.7(2)
C(1)-C(2)-C(3)	119.6(2)	C(3)-C(2)-C(7)	122.7(2)
C(1)-C(6)-C(5)	119.2(2)	C(1)-C(6)-C(8)	117.6(2)
C(5)-C(6)-C(8)	123.2(2)	C(4)-C(5)-C(6)	120.0(2)
C(5)-C(4)-C(3)	120.5(2)	C(4)-C(3)-C(2)	119.5(2)
C(6)-C(8)-H(8)	122(3)	N(2)-C(8)-C(6)	119.6(2)
N(2)-C(8)-H(8)	118(3)	C(8)-N(2)-Bi(1)	112.42(16)
C(8)-N(2)-C(13)	122.1(2)	C(13)-N(2)-Bi(1)	125.44(15)
N(2)-C(13)-C(16)	110.7(2)	N(2)-C(13)-C(14)	108.3(2)
N(2)-C(13)-C(15)	105.94(19)	C(14)-C(13)-C(16)	111.2(2)
C(15)-C(13)-C(16)	110.4(2)	C(15)-C(13)-C(14)	110.3(2)
N(3)-C(9)-C(11)	108.3(2)	N(3)-C(9)-C(10)	110.6(2)
N(3)-C(9)-C(12)	106.3(2)	C(11)-C(9)-C(12)	108.8(2)
C(10)-C(9)-C(11)	111.7(3)	C(10)-C(9)-C(12)	111.0(3)

[(2-(tBuNCH)-6-(CO_2)C_6H_3)Bi(4-trifluoromethylphenyl)] (S7)

Single crystals of **S7** (a decomposition product of **12c**) were obtained by vapor diffusion of diethyl ether (anti-solvent) into a solution of **12c** in 1,2-dichloroethane at room temperature over 7 days under air. CCDC deposit number: **2259025**.

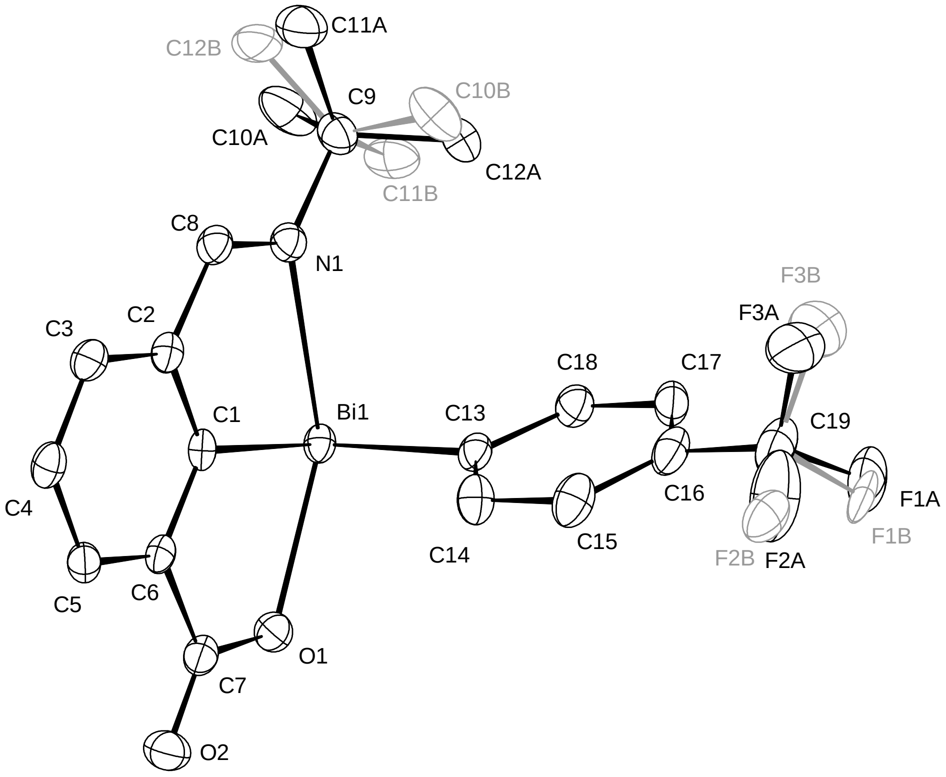

Figure S44. The molecular structure of **S7**. H atoms have been removed for clarity. Main structure shown in black and disordered parts shown in grey.

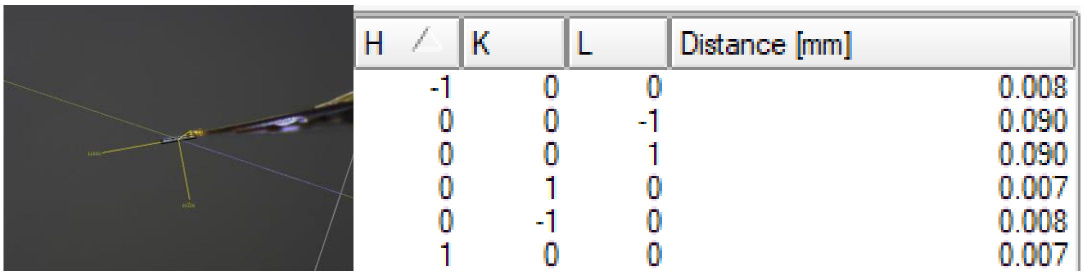

H	K	L	Distance [mm]
-1	0	0	0.008
0	0	-1	0.090
0	0	1	0.090
0	1	0	0.007
0	-1	0	0.008
1	0	0	0.007

Figure S45. Crystal faces of **S7**.

INTENSITY STATISTICS FOR DATASET # 1 14996sadabs.raw

Resolution	#Data	#Theory	%Complete	Redundancy	Mean I	Mean I/s	Rmerge	Rsigma
Inf - 2.80	101	103	98.1	33.86	155.99	76.49	0.0349	0.0159
2.80 - 1.85	238	238	100.0	38.93	119.45	74.99	0.0387	0.0095
1.85 - 1.45	338	338	100.0	38.80	77.50	58.73	0.0521	0.0112
1.45 - 1.26	345	345	100.0	39.31	64.00	53.42	0.0634	0.0130
1.26 - 1.15	326	326	100.0	39.08	47.85	46.03	0.0779	0.0150
1.15 - 1.06	362	362	100.0	38.49	41.94	40.48	0.0871	0.0170
1.06 - 1.00	316	316	100.0	38.04	37.85	35.89	0.0948	0.0185
1.00 - 0.95	325	325	100.0	35.83	32.44	31.33	0.1113	0.0213
0.95 - 0.90	412	412	100.0	30.39	23.81	24.50	0.1285	0.0279
0.90 - 0.87	290	290	100.0	28.25	24.88	23.66	0.1330	0.0293
0.87 - 0.84	337	337	100.0	26.36	22.56	20.80	0.1392	0.0329
0.84 - 0.81	389	389	100.0	25.28	18.64	18.09	0.1549	0.0387
0.81 - 0.79	289	289	100.0	24.52	16.40	16.32	0.1704	0.0438
0.79 - 0.77	307	307	100.0	23.59	15.01	14.87	0.1772	0.0477
0.77 - 0.75	375	375	100.0	22.83	14.02	13.82	0.1879	0.0519
0.75 - 0.73	379	379	100.0	21.07	13.24	12.40	0.2001	0.0586
0.73 - 0.71	442	442	100.0	18.67	11.14	9.60	0.2267	0.0733
0.71 - 0.70	239	239	100.0	17.24	10.20	8.44	0.2429	0.0823
0.70 - 0.69	282	282	100.0	14.00	9.70	7.63	0.2439	0.0991
0.69 - 0.67	632	644	98.1	8.94	8.93	5.52	0.2534	0.1446
0.77 - 0.67	2349	2361	99.5	16.36	11.08	9.27	0.2162	0.0852
Inf - 0.67	6724	6738	99.8	27.03	31.68	26.34	0.0870	0.0268

Complete .cif-data of the compound are available under the CCDC number CCDC-**2259025**.

The occupancy of the fluorine was fixed to be 60:40%. Anisotropic atomic displacement parameters were used for the main part. For all other disordered atoms isotropic atomic displacement parameters where used.

Table S8. Crystal data and structure refinement of compound **[(2-(tBuNCH)-6-(CO_2)C_6H_3)Bi(4-trifluoromethylphenyl)]**.

Identification code	14996	
Empirical formula	C_{38} H_{34} Bi_2 F_6 N_2 O_4	
Color	yellow	
Formula weight	1114.63 g·mol^{-1}	
Temperature	100(2) K	
Wavelength	0.71073 Å	
Crystal system	Monoclinic	
Space group	$P2_1/c$, (no. 14)	
Unit cell dimensions	a = 14.0632(8) Å	□= 90°.
	b = 15.3836(9) Å	□= 99.127(2)°.
	c = 8.6105(4) Å	□ = 90°.
Volume	1839.23(17) Å^3	
Z	2	
Density (calculated)	2.013 Mg·m^{-3}	
Absorption coefficient	9.628 mm^{-1}	
F(000)	1056 e	
Crystal size	0.185 x 0.021 x 0.02 mm^3	
□ range for data collection	1.976 to 32.247°.	
Index ranges	-21 □ h □ 21, -23□ k □ 23, -12 □ l □ 12	
Reflections collected	178872	
Independent reflections	6506 [R_{int} = 0.0883]	
Reflections with I>2□(I)	5372	
Completeness to □ = 25.242°	100.0 %	
Absorption correction	Gaussian	
Max. and min. transmission	0.8305 and 0.4088	
Refinement method	Full-matrix least-squares on F^2	
Data / restraints / parameters	6506 / 0 / 295	
Goodness-of-fit on F^2	1.036	
Final R indices [I>2□(I)]	R_1 = 0.0210	wR^2 = 0.0393
R indices (all data)	R_1 = 0.0318	wR^2 = 0.0428
Extinction coefficient	n/a	
Largest diff. peak and hole	0.987 and -0.640 e·Å^{-3}	

Table S9. Bond lengths [Å] and angles [°] of compound **[(2-(tBuNCH)-6-(CO_2)C_6H_3)Bi(4-trifluoromethylphenyl)]**.

Bi(1)-O(1)	2.3322(18)	Bi(1)-N(1)	2.618(2)
Bi(1)-C(1)	2.219(2)	Bi(1)-C(13)	2.248(2)
F(1A)-C(19)	1.341(18)	F(1B)-C(19)	1.36(2)
F(2A)-C(19)	1.216(15)	F(2B)-C(19)	1.49(2)
F(3A)-C(19)	1.289(14)	F(3B)-C(19)	1.475(19)
O(1)-C(7)	1.302(3)	O(2)-C(7)	1.223(3)
N(1)-C(8)	1.276(3)	N(1)-C(9)	1.483(3)
C(1)-C(2)	1.391(3)	C(1)-C(6)	1.384(3)
C(2)-C(3)	1.399(3)	C(2)-C(8)	1.465(3)
C(3)-C(4)	1.391(4)	C(4)-C(5)	1.393(3)
C(5)-C(6)	1.399(3)	C(6)-C(7)	1.508(3)
C(9)-C(10A)	1.591(7)	C(9)-C(10B)	1.578(7)
C(9)-C(11A)	1.579(6)	C(9)-C(11B)	1.523(6)
C(9)-C(12A)	1.474(6)	C(9)-C(12B)	1.433(7)
C(13)-C(14)	1.389(4)	C(13)-C(18)	1.396(4)
C(14)-C(15)	1.388(4)	C(15)-C(16)	1.384(4)
C(16)-C(17)	1.388(4)	C(16)-C(19)	1.493(4)
C(17)-C(18)	1.386(4)		
O(1)-Bi(1)-N(1)	141.18(6)	C(1)-Bi(1)-O(1)	72.20(7)
C(1)-Bi(1)-N(1)	69.10(8)	C(1)-Bi(1)-C(13)	94.03(8)
C(13)-Bi(1)-O(1)	93.31(8)	C(13)-Bi(1)-N(1)	86.49(7)
C(7)-O(1)-Bi(1)	118.41(15)	C(8)-N(1)-Bi(1)	110.78(16)
C(8)-N(1)-C(9)	120.5(2)	C(9)-N(1)-Bi(1)	128.55(15)
C(2)-C(1)-Bi(1)	121.66(17)	C(6)-C(1)-Bi(1)	117.51(16)
C(6)-C(1)-C(2)	120.8(2)	C(1)-C(2)-C(3)	119.7(2)
C(1)-C(2)-C(8)	117.9(2)	C(3)-C(2)-C(8)	122.5(2)
C(4)-C(3)-C(2)	119.4(2)	C(3)-C(4)-C(5)	120.8(2)
C(4)-C(5)-C(6)	119.4(2)	C(1)-C(6)-C(5)	119.8(2)
C(1)-C(6)-C(7)	117.4(2)	C(5)-C(6)-C(7)	122.8(2)
O(1)-C(7)-C(6)	114.4(2)	O(2)-C(7)-O(1)	123.9(2)
O(2)-C(7)-C(6)	121.7(2)	N(1)-C(8)-C(2)	120.3(2)
N(1)-C(9)-C(10A)	106.0(3)	N(1)-C(9)-C(10B)	102.6(3)
N(1)-C(9)-C(11A)	115.8(3)	N(1)-C(9)-C(11B)	105.5(3)
C(11A)-C(9)-C(10A)	104.4(4)	C(11B)-C(9)-C(10B)	107.4(4)
C(12A)-C(9)-N(1)	111.2(3)	C(12A)-C(9)-C(10A)	109.4(4)
C(12A)-C(9)-C(11A)	109.7(4)	C(12B)-C(9)-N(1)	113.0(3)
C(12B)-C(9)-C(10B)	112.5(5)	C(12B)-C(9)-C(11B)	114.8(4)
C(14)-C(13)-Bi(1)	122.94(18)	C(14)-C(13)-C(18)	119.1(2)
C(18)-C(13)-Bi(1)	117.92(18)	C(15)-C(14)-C(13)	120.7(3)
C(16)-C(15)-C(14)	119.6(3)	C(15)-C(16)-C(17)	120.4(3)
C(15)-C(16)-C(19)	121.4(3)	C(17)-C(16)-C(19)	118.2(3)
C(18)-C(17)-C(16)	119.7(3)	C(17)-C(18)-C(13)	120.3(3)
F(1A)-C(19)-C(16)	112.5(11)	F(1B)-C(19)-F(2B)	102.7(16)
F(1B)-C(19)-F(3B)	101.0(18)	F(1B)-C(19)-C(16)	110.7(16)
F(2A)-C(19)-F(1A)	108.9(12)	F(2A)-C(19)-F(3A)	96.4(8)
F(2A)-C(19)-C(16)	116.3(8)	F(2B)-C(19)-C(16)	110.8(9)
F(3A)-C(19)-F(1A)	109.0(13)	F(3A)-C(19)-C(16)	112.5(7)
F(3B)-C(19)-F(2B)	121.1(10)	F(3B)-C(19)-C(16)	109.5(9)

12. DFT coordinates

Closed-shell singlet structure of *N*,*C*,*N*-Bi(I) **1** in gas phase

C	0.89980798315739	-4.14555028259518	1.22294506717789
C	-0.34479697266432	-4.69266423568400	1.52616709891637
C	1.00203444051613	-2.83385019320700	0.75481668470842
H	-0.41475443377508	-5.70970771907220	1.88839719633148
C	-1.49935549718646	-3.93053358477589	1.36345781851436
C	-0.16366094578634	-2.05883120393148	0.58875264123832
H	-2.46708883129558	-4.35986685359875	1.60080479254193
C	-1.42102311456017	-2.61659271755158	0.89703248602793
H	1.79700140429260	-4.74202169375277	1.35112158537807
C	2.26154443335893	-2.21624246513627	0.42587142842935
C	-2.58055465886354	-1.78181206213852	0.71122619841962
N	2.28324520551073	-0.99929443049154	-0.00199415758424
N	-2.43573412390106	-0.57551748612620	0.27728318214746
H	3.17067457813183	-2.80082496723480	0.54904933612360
H	-3.55831372290034	-2.19708156722925	0.94569326490009
Bi	-0.02439508374287	-0.03408696907354	-0.13228951211682
C	-3.56945529211676	0.34149335241931	0.06763326370330
C	3.52839969158071	-0.29774505640801	-0.35906109330095
C	3.61469493886017	0.95153657853702	0.52862447451203
H	4.50145737889727	1.53650329108648	0.27666350270624
H	2.73471696217860	1.58124988630479	0.39581032172603
H	3.66967109167597	0.66960155696766	1.58109174189366
C	4.79392675617793	-1.13944149332221	-0.17283798187613
H	5.66368763684345	-0.54681194967666	-0.45818439872933
H	4.92667407121198	-1.44496768771798	0.86666988833401
H	4.78076044395403	-2.03289166304021	-0.79979941437936
C	3.40776853456840	0.11784847398744	-1.83178859407751
H	3.31549358535989	-0.76162354174167	-2.47073365168667
H	2.52530209661576	0.73894135607716	-1.98685192290340
H	4.28913941833659	0.68335740522878	-2.14062016907306
C	-4.93652299000524	-0.27646601005226	0.37430169192598

H	-5.71471958716521	0.46392122673504	0.18628112896793
H	-5.13976772309918	-1.14118933959269	-0.26016552209368
H	-5.01680329688876	-0.58377412646579	1.41860421037932
C	-3.34457110760981	1.55521279480538	0.98034677070400
H	-4.13654343398476	2.29253949414549	0.83473515732252
H	-3.34010806697312	1.24986687769927	2.02772065044548
H	-2.38618729108319	2.02792560038266	0.76466154817060
C	-3.53025399814810	0.78134850455053	-1.40226821679730
H	-2.57471993300790	1.24919209458315	-1.64039667071805
H	-3.65648961112923	-0.07821428905137	-2.06209238727925
H	-4.32777793534133	1.49861009515765	-1.60584943903025

Closed-shell singlet structure of *N*,*C*,*N*-Bi(I) **1** in MeCN

C	0.90111196092859	-4.15184060182741	1.22001698817595
C	-0.34529016710298	-4.69841635890695	1.52288761960381
C	1.00120665792925	-2.83730507756382	0.75516596815614
H	-0.41536944954242	-5.71693023357217	1.88262451093952
C	-1.50163719570664	-3.93598045509929	1.36306818677019
C	-0.16381748450795	-2.06097526595641	0.59167759749277
H	-2.47030859931196	-4.36371443198683	1.59869229547488
C	-1.42077210690352	-2.61984525290412	0.89922942423602
H	1.79930927381775	-4.74702167926828	1.34544113417310
C	2.26609735217266	-2.21881127308783	0.42575473739048
C	-2.58584184220973	-1.78366799418855	0.71363079671572
N	2.28330395692606	-1.00258243752785	0.00280535454463
N	-2.43700531499926	-0.57987554437421	0.28132801369513
H	3.17392130359738	-2.80469750058857	0.54656544147527
H	-3.56227629242034	-2.20087520068485	0.94737772979224
Bi	-0.02341509890192	-0.01759166329884	-0.13256885075510
C	-3.57059805477660	0.34107249477241	0.06606693160868
C	3.52930412451926	-0.29591400905153	-0.35513984018077
C	3.61344538024795	0.94736265528628	0.53903220822266
H	4.50166430706299	1.53088070861982	0.28887207298791

H	2.73418590497045	1.57927309953480	0.40488144032374
H	3.67224935226998	0.66027529339073	1.59087235834727
C	4.79281270058038	-1.13905857506498	-0.17857156309620
H	5.65997866903865	-0.54209621273874	-0.46415560867909
H	4.92867276389306	-1.45191640261611	0.85864720590054
H	4.77616635040508	-2.02915178870347	-0.81059891067346
C	3.39853254750752	0.13049634195848	-1.82259782525270
H	3.30276439848448	-0.74384580196578	-2.46952277979559
H	2.51879451223897	0.76000263201018	-1.96469930281541
H	4.28156056759068	0.69413311187635	-2.13015038591982
C	-4.93579704668422	-0.26893508697041	0.38669247475123
H	-5.70967605515624	0.47631654848955	0.19813386834300
H	-5.14760368050284	-1.13786864262080	-0.23960429724565
H	-5.00900716663052	-0.56941452881704	1.43378054478635
C	-3.33139212581805	1.56301460222601	0.96161936845230
H	-4.12452376285658	2.29864580794033	0.81414124979302
H	-3.32062608393074	1.27139849720903	2.01378071593221
H	-2.37492409475316	2.03229243089043	0.72606366496871
C	-3.53177297245639	0.76437467722462	-1.40753203117701
H	-2.57624744236451	1.23237697521095	-1.64948694465446
H	-3.66441217098204	-0.10158009397533	-2.05919686401922
H	-4.33036487566252	1.47954123672024	-1.61419469878899

Open-shell doublet structure of *N*,*C*,*N*-Bi(II) **1*** in gas phase

C	0.90003191058926	-4.17848519030054	1.22677442923013
C	-0.34726572954654	-4.71968229969058	1.52653712789871
C	0.99405791218640	-2.86369814224027	0.76195081387618
H	-0.41744447552715	-5.73711223756307	1.88604918056460
C	-1.50501545539188	-3.96273633802637	1.36792421707946
C	-0.16742769249343	-2.11241087396950	0.60550810535480
H	-2.47010885245545	-4.39447545933812	1.60468326957857
C	-1.41762634603474	-2.64729480414276	0.90370597628063
H	1.79406084675292	-4.77703206875367	1.35448205195500

C	2.26472590865621	-2.22320798428043	0.42619021499904
C	-2.58480099899210	-1.78792773939301	0.71210806842514
N	2.28320231556286	-1.01725438257665	0.00620670360065
N	-2.43790319553539	-0.59314839994664	0.28560407432829
H	3.17797097813817	-2.80178066099706	0.54563205732722
H	-3.56600265285979	-2.19667783497466	0.94272051096562
Bi	-0.02505283883163	-0.05217215007736	-0.12172873515358
C	-3.55631195764489	0.35150683960357	0.06306231787664
C	3.51720350910692	-0.28415525984630	-0.35781415922726
C	3.57187279439012	0.95694817208577	0.54139836771362
H	4.44432986720440	1.56040300400003	0.29077664227644
H	2.68663185294964	1.58228005930611	0.40821755656102
H	3.63888900912904	0.67421823616384	1.59260029715893
C	4.78751140266818	-1.11455282977829	-0.17934567633039
H	5.64836176795086	-0.51135892576086	-0.46525037324424
H	4.93145895317404	-1.41984541632929	0.85853211988718
H	4.78501700448318	-2.00294947890516	-0.81324373912042
C	3.36464023190549	0.13066190337246	-1.82636198135999
H	3.28406601195719	-0.74441047737327	-2.47228043466136
H	2.47776476048344	0.75057515595996	-1.97330875815476
H	4.23118599164508	0.71229410014367	-2.14090567166210
C	-4.92522579651614	-0.24701066688502	0.38398421730716
H	-5.69278038934903	0.50237751062284	0.19443053316688
H	-5.14854365313728	-1.11111298233582	-0.24416962971828
H	-5.00577369901525	-0.54020532545912	1.43214008655314
C	-3.29115065774826	1.56508432102646	0.96257077705581
H	-4.06610719348182	2.31670122458903	0.81199020086518
H	-3.28970129644032	1.27754646768885	2.01459903186643
H	-2.32989795761619	2.02821433487154	0.73037624784440
C	-3.49740072360859	0.76194947737248	-1.41338005482209
H	-2.53765892564321	1.22117160155082	-1.65931967789033
H	-3.64312642532080	-0.10104231945781	-2.06413023331072
H	-4.27825311574353	1.49135284004427	-1.62868607294132

Open-shell doublet structure of *N*,*C*,*N*-Bi(II) **1*** in MeCN

C	0.90130374344714	-4.16870971393021	1.22214575213326
C	-0.34632660459846	-4.71236874922095	1.51973463469496
C	0.99325224292498	-2.85305048853933	0.75854126234902
H	-0.41603274564305	-5.73085547659660	1.87879966907471
C	-1.50481856776015	-3.95540542147741	1.35975041534981
C	-0.16730508911565	-2.09745069036102	0.59824950296282
H	-2.47271470695996	-4.38332352905118	1.59358088640419
C	-1.41663993037024	-2.63939833221440	0.89651520099644
H	1.79924585681003	-4.76197251814132	1.34968063124301
C	2.26718261323002	-2.21791150472880	0.42765560667715
C	-2.58871598486762	-1.78677924480104	0.70779093273979
N	2.28424042951325	-1.01106793470718	0.00953377965543
N	-2.44098543121012	-0.59172675895789	0.28178269746126
H	3.17526193757332	-2.80326815619810	0.54946689548407
H	-3.56506856185130	-2.20420962541464	0.94178973146865
Bi	-0.02409908658888	-0.02610153304487	-0.13216617266754
C	-3.56060121803334	0.34684287157165	0.06427374932679
C	3.51956580044713	-0.28674882018154	-0.35407947191952
C	3.58117643064049	0.95740204255081	0.53906871634493
H	4.46033321777354	1.55198539952306	0.28553082372535
H	2.69550479250000	1.58073016901399	0.40292029338663
H	3.64566993563072	0.67314591280665	1.59105163720460
C	4.78790196460963	-1.11881574112539	-0.17408593387035
H	5.64873540754194	-0.51470298150229	-0.46231461739309
H	4.92562823157078	-1.42543957655684	0.86463895570470
H	4.77799740381093	-2.01066717060435	-0.80351018719348
C	3.37199806531750	0.12678758022703	-1.82285205350192
H	3.28952216822156	-0.75279833098479	-2.46402437764456
H	2.48388866395630	0.74465806463695	-1.96810735506657
H	4.24449862312938	0.70321612595807	-2.13461701094162
C	-4.92873085063525	-0.24937456478930	0.39025191895745
H	-5.69411793411342	0.50453241253824	0.20364014296439
H	-5.15138912197449	-1.11584412173970	-0.23545560884009

H	-5.00010498142488	-0.54736027537703	1.43808524967155
C	-3.29392709086030	1.56243061071114	0.95932571092836
H	-4.07523787342034	2.30981988998225	0.81241252671501
H	-3.28517396967070	1.27112833807047	2.01131769009065
H	-2.33248209838535	2.02039622667728	0.71919072309960
C	-3.50888917026659	0.75945572097590	-1.41109818931873
H	-2.54573910388791	1.21170194749352	-1.65549796069184
H	-3.65860967236604	-0.10658755889801	-2.05848504576565
H	-4.29279473464457	1.48925050640725	-1.61960175199960

Open-shell triplet structure of *N*,*C*,*N*-Bi(II) **1*** in gas phase

C	0.90518185806415	-4.24632618183390	1.17179624928043
C	-0.35132541005064	-4.77528233945777	1.44856838895841
C	1.00330854512121	-2.88919271328943	0.73369633536191
H	-0.42218782955010	-5.80466944321489	1.78098880322660
C	-1.52056574747110	-4.03445791094920	1.31606255333638
C	-0.17320011774082	-2.15478433988284	0.60337838062904
H	-2.48205923135239	-4.47918746485710	1.54073245386957
C	-1.44221984289788	-2.67505114645007	0.87879428202841
H	1.79701789913335	-4.84960361053325	1.28579214011738
C	2.23544594828978	-2.22874760628765	0.41569586711064
C	-2.56108513045736	-1.80755098744303	0.70142251236016
N	2.26201045478942	-0.98185729686585	0.02478498833506
N	-2.40626870090120	-0.55722636049889	0.31059714404873
H	3.15431482422606	-2.80253116562828	0.51087374752153
H	-3.55213938753801	-2.20463635226324	0.90082240691011
Bi	-0.05217556585725	-0.07733169176784	-0.06281005360864
C	-3.52445578110480	0.36250185072969	0.07145253671272
C	3.49504477541182	-0.26476023018909	-0.32185027240216
C	3.56987142257319	0.98281950796054	0.57092003804147
H	4.44949320790851	1.58232874983922	0.32750828078805
H	2.68579046450444	1.60861488253775	0.43524334015219
H	3.62351770766601	0.69635283615240	1.62230052376364
C	4.76959691247597	-1.09325451127900	-0.13299237650690

H	5.63627979782111	-0.48743091512740	-0.40014731639418
H	4.89139907261504	-1.41215421538841	0.90362136451062
H	4.77339842476454	-1.97854796352009	-0.77105692563653
C	3.38597950391388	0.16244181821130	-1.79358523940580
H	3.30741146859337	-0.71342463758730	-2.43939634728198
H	2.50012043100536	0.78026189847594	-1.95194963549700
H	4.26157815803090	0.74097140973185	-2.09562566407603
C	-4.88135101720019	-0.18894783581591	0.52206830535911
H	-5.65113669921282	0.56502194358004	0.35282811322252
H	-5.16333861039108	-1.08095068876962	-0.03928728742634
H	-4.87835158842984	-0.43453906323218	1.58544342362113
C	-3.23526021812242	1.65875535115055	0.84234850470153
H	-4.02007441639368	2.39791676554406	0.66916147715654
H	-3.17416388399645	1.45935740925691	1.91325605418247
H	-2.28664400611720	2.09647338744245	0.52458355884093
C	-3.57862708682001	0.66221047446572	-1.43526846823127
H	-2.63350634457690	1.08791148211011	-1.77821164416299
H	-3.75994094660734	-0.25480153397467	-1.99795770958834
H	-4.37428031411860	1.37485343891842	-1.66377283392916

Open-shell triplet structure of *N*,*C*,*N*-Bi(II) **1*** in MeCN

C	0.90817262836205	-4.23436676312011	1.23911215163745
C	-0.34956173081399	-4.75620891085121	1.53331362131022
C	1.00865657058225	-2.88381762125615	0.76318061169639
H	-0.41960896348295	-5.77769280505500	1.89310997603272
C	-1.52080207212690	-4.01724926080145	1.38315060778626
C	-0.17076543969911	-2.14626245627838	0.61385636318708
H	-2.48415291750715	-4.45359398117757	1.61982936944389
C	-1.43647435981345	-2.66539373472618	0.90771745823927
H	1.80062142204195	-4.83532047018135	1.36853429279866
C	2.24057638951659	-2.23301710505314	0.42979118845098
C	-2.56414233039955	-1.80394840825153	0.71279154743840
N	2.25658746453268	-0.99464312312949	0.00585340656146
N	-2.41022829791455	-0.57911618378604	0.27683516374568

H	3.16290733305078	-2.79814760110095	0.54053104860462
H	-3.55240964611182	-2.19723117374984	0.93850480925680
Bi	-0.02928109679846	-0.07204803372676	-0.11442037300892
C	-3.52415155699980	0.35643855308160	0.06251743209343
C	3.48560823875122	-0.27401680047673	-0.35855014369632
C	3.55929708500933	0.98349838190586	0.51855595625439
H	4.43597448579338	1.58157849178111	0.26008904302205
H	2.67028634162224	1.60238459119010	0.38069830158831
H	3.62539194194926	0.71021761963701	1.57383784861322
C	4.76288446466875	-1.09573872195324	-0.17085032158582
H	5.62322690394927	-0.48880206732252	-0.45716223737252
H	4.89817809933747	-1.39936819765342	0.86921317698850
H	4.76260338775406	-1.99118353698015	-0.79544361145095
C	3.35940787034591	0.13706496422117	-1.83228070609860
H	3.28732632669594	-0.74590440004760	-2.47087956116694
H	2.46554008057562	0.74481330300686	-1.98752390256919
H	4.22868280018432	0.72017632325343	-2.14455621883846
C	-4.89966481326594	-0.23621701071498	0.37643515166555
H	-5.66462530595520	0.51889790694555	0.18902018856553
H	-5.12021186611210	-1.10039331298274	-0.25340613027434
H	-4.97955213543822	-0.54082658822994	1.42184712965657
C	-3.27948891950200	1.57548719755710	0.96285345089565
H	-4.05660882140656	2.32805790755246	0.81185449844773
H	-3.28280271405406	1.28068535907920	2.01438849934956
H	-2.31287055534184	2.03151414690836	0.73864196041439
C	-3.48371602043878	0.79136808889770	-1.40900814346624
H	-2.51825239064537	1.24063029305704	-1.65118419810740
H	-3.63550301048937	-0.06771388496060	-2.06592827466907
H	-4.26465187040591	1.52695402549256	-1.61404043144001

13. NMR spectra

Bismuthinidene **1**

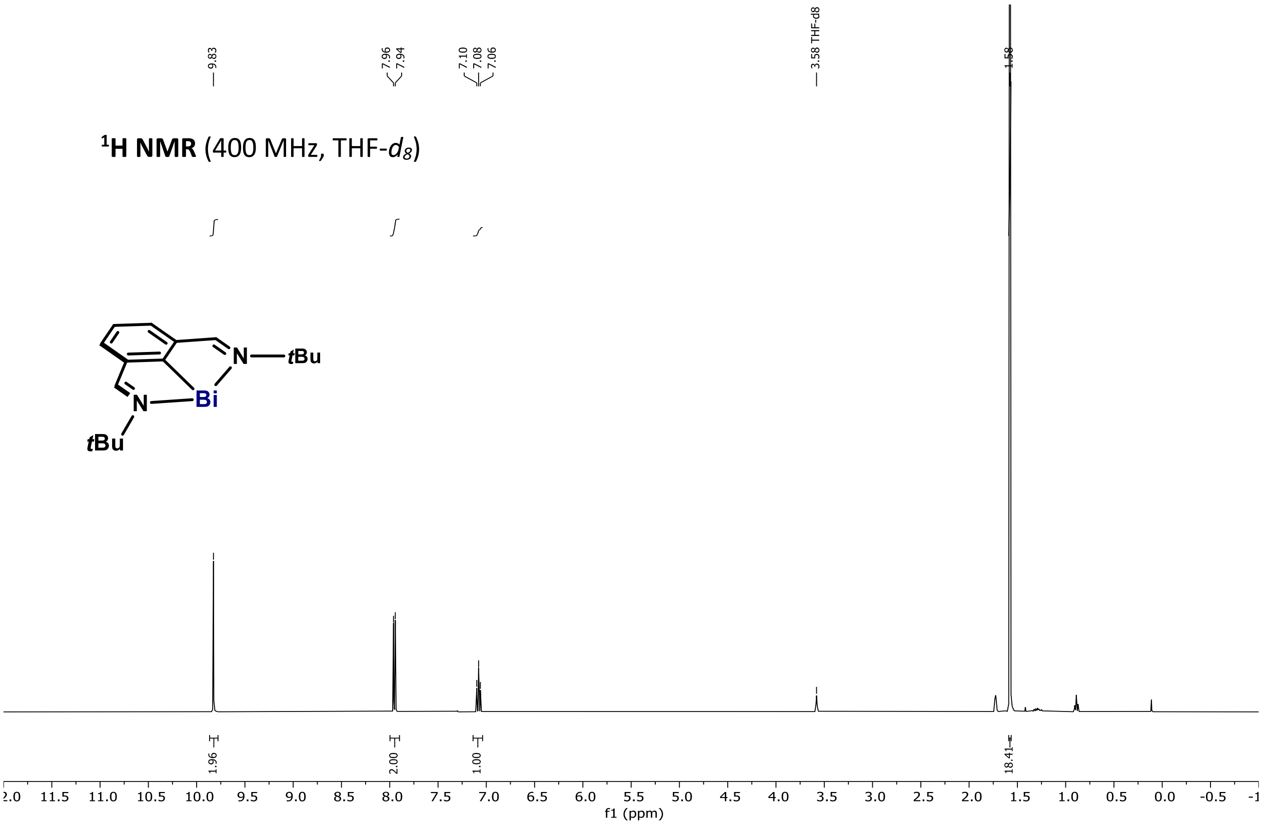

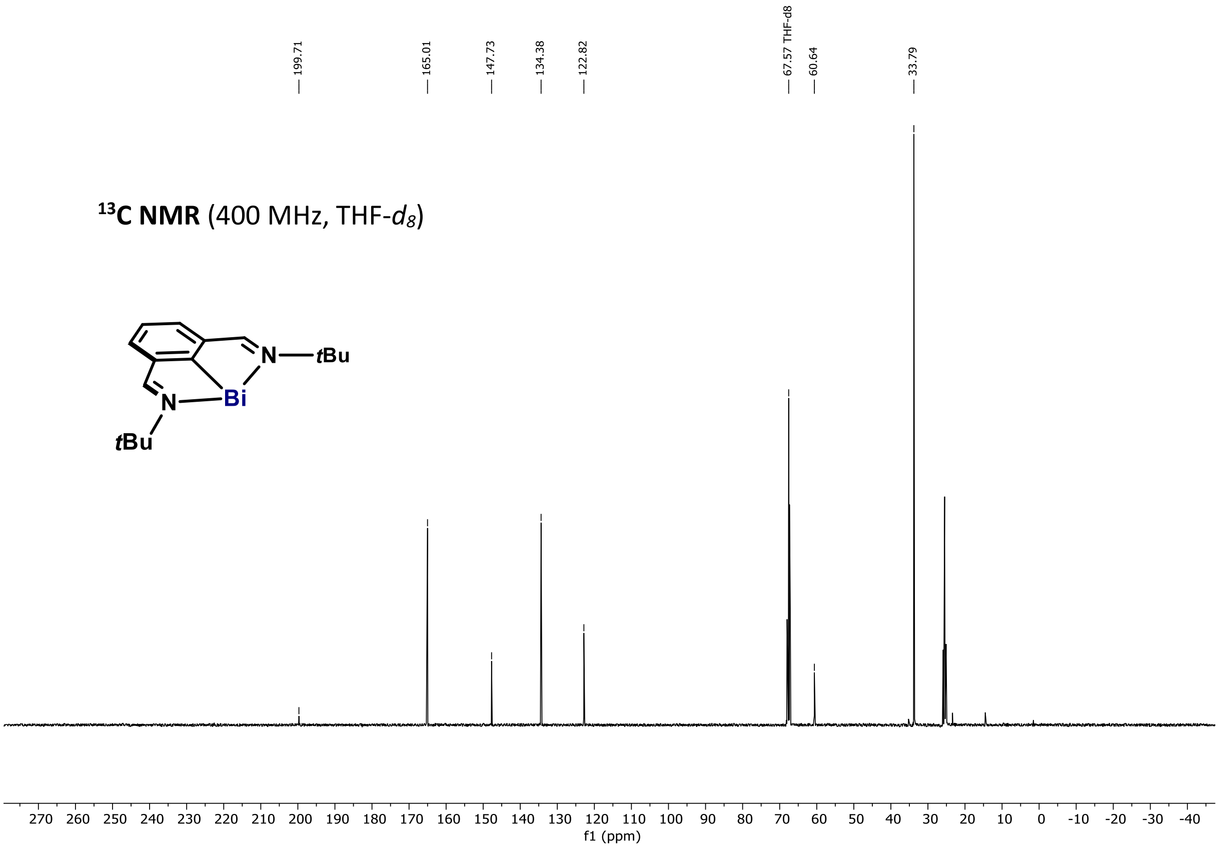

(*E*)-1-(2-Bromo-3-((*E*)-(cyclohexylimino)methyl)phenyl)-N-cyclohexylmethanimine (S3)

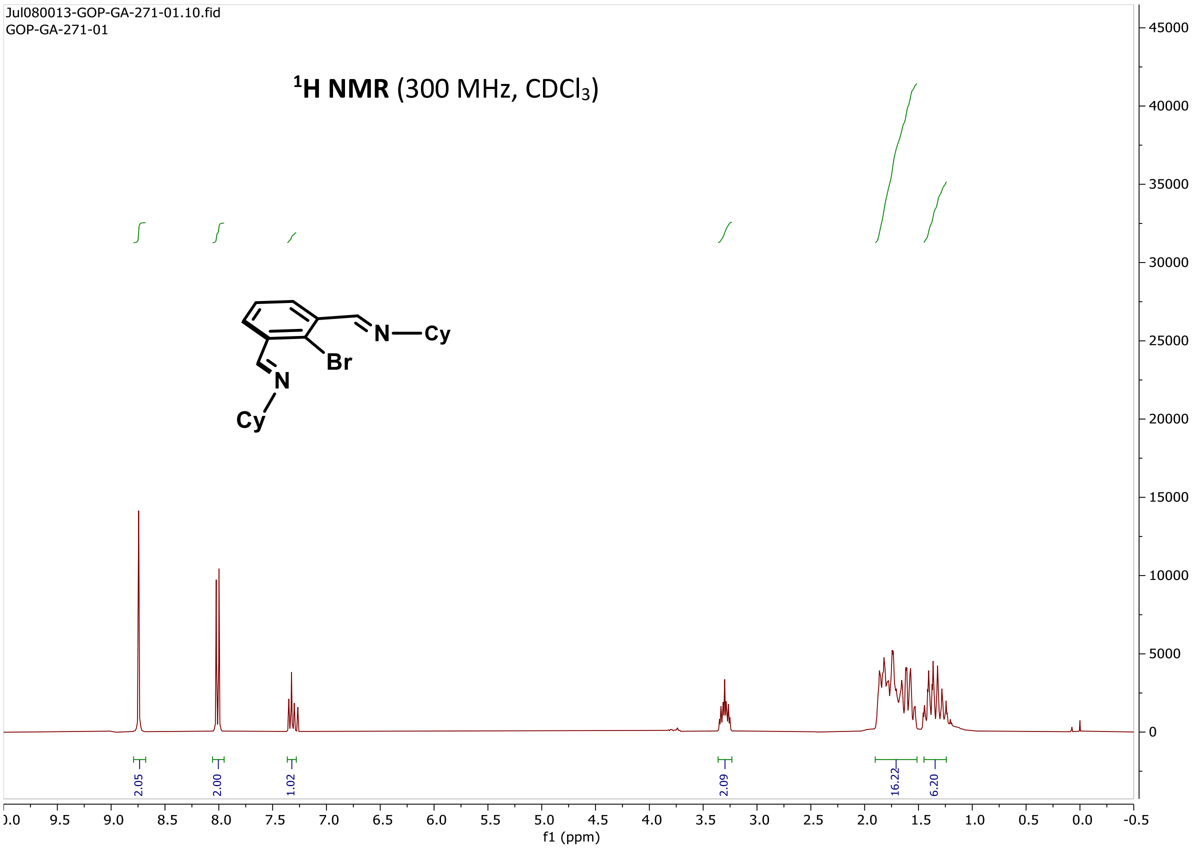

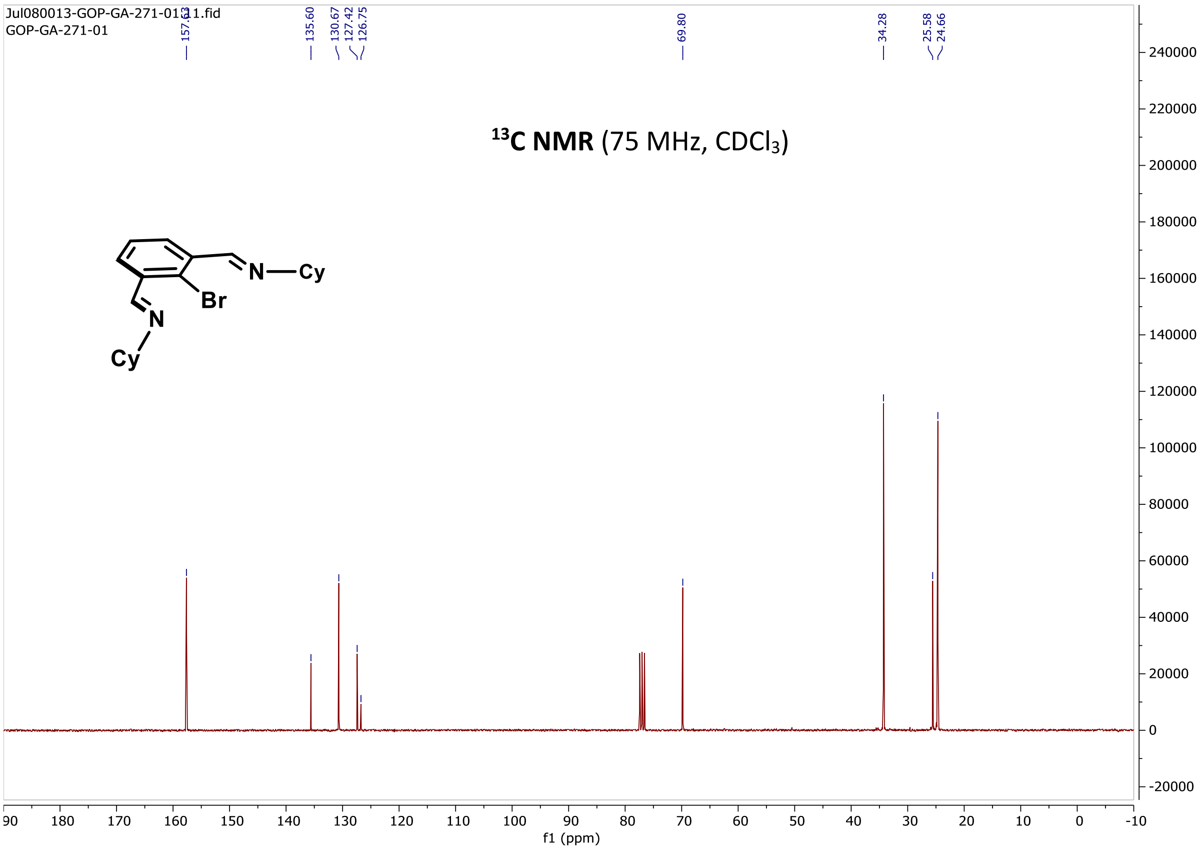

Bis-cyclohexyl dichlorobismuthine (S4)

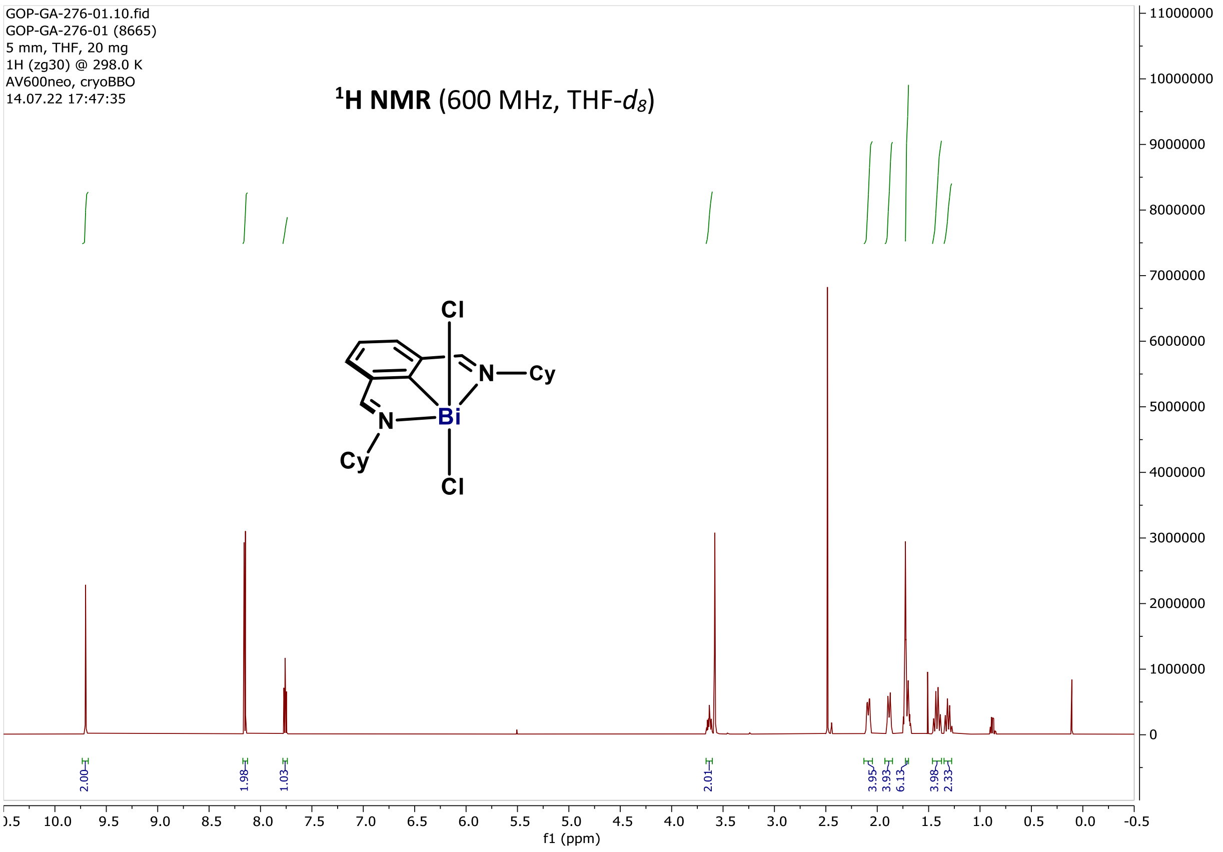

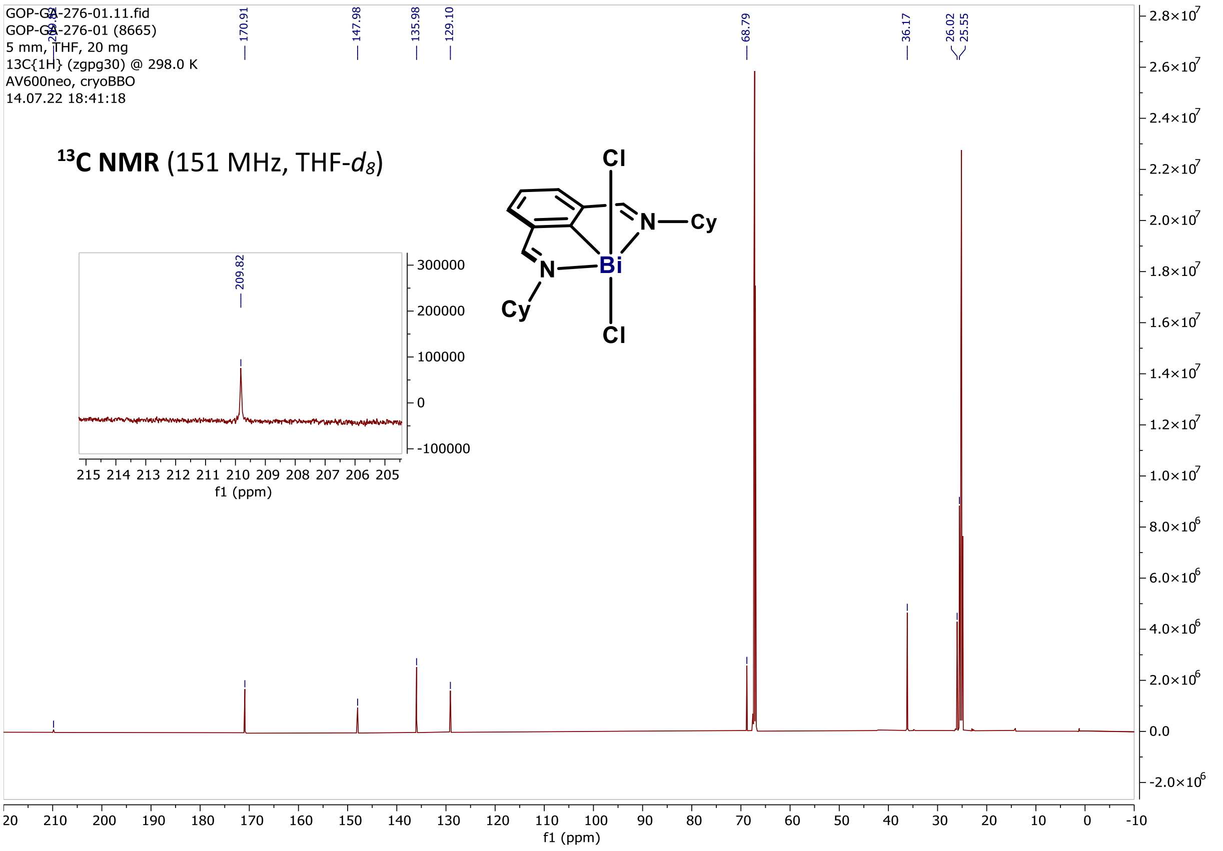

Bis-cyclohexyl bismuthinidine (S5)

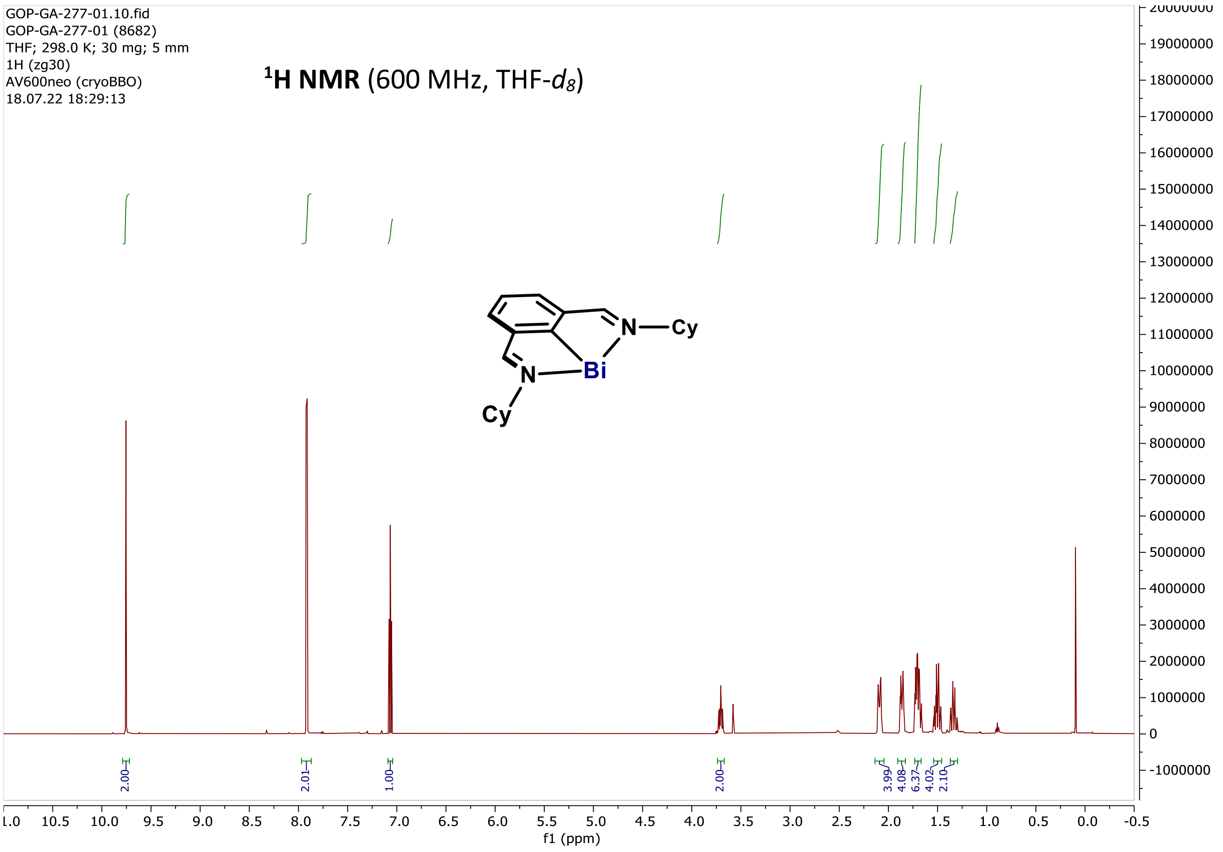

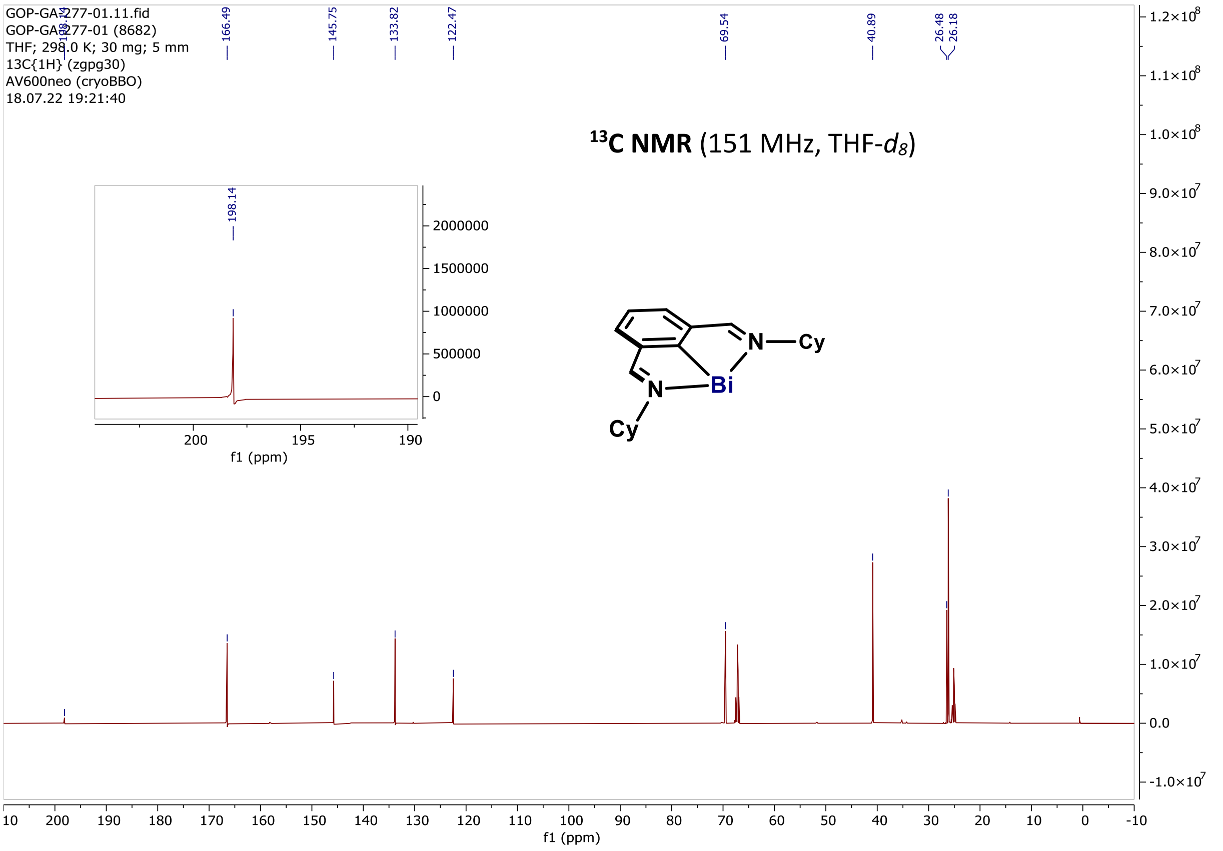

5-(4-Methoxyphenyl)-5H-thianthren-5-ium trifluoromethanesulfonate (5a)

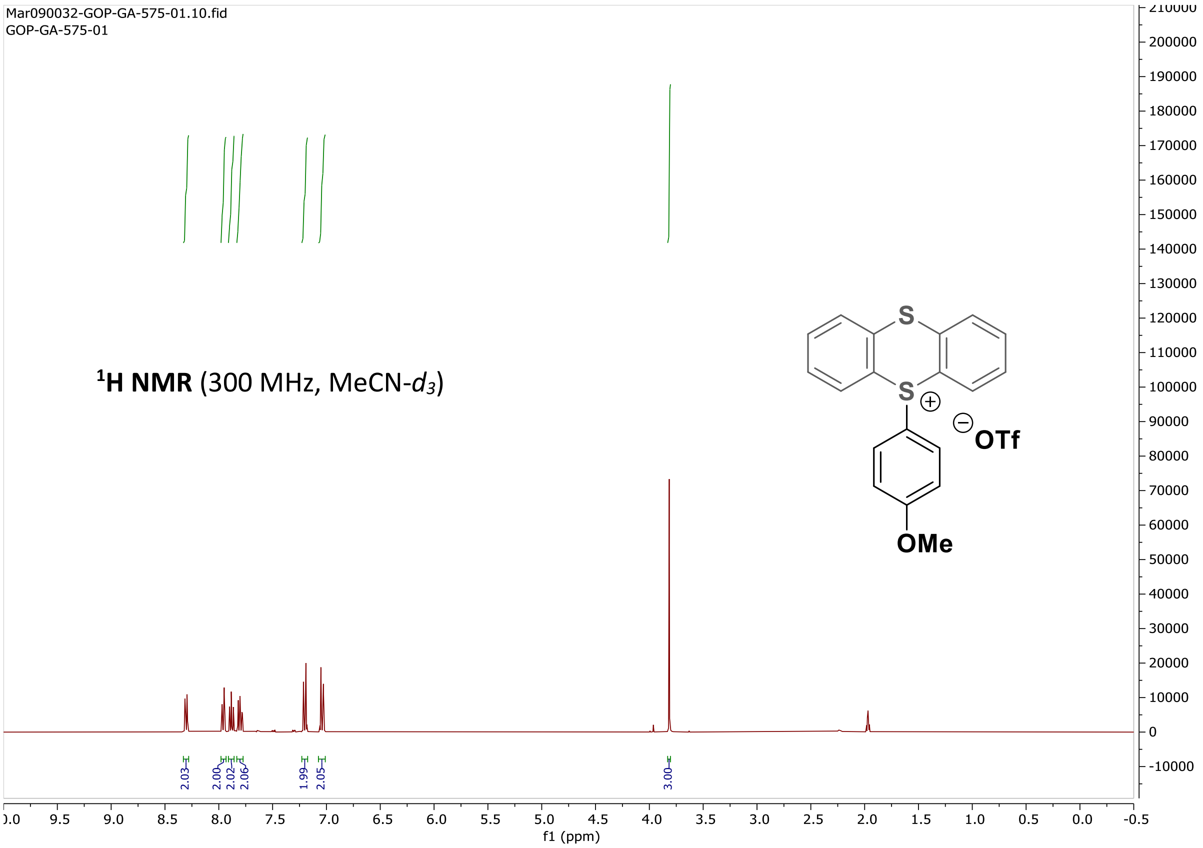

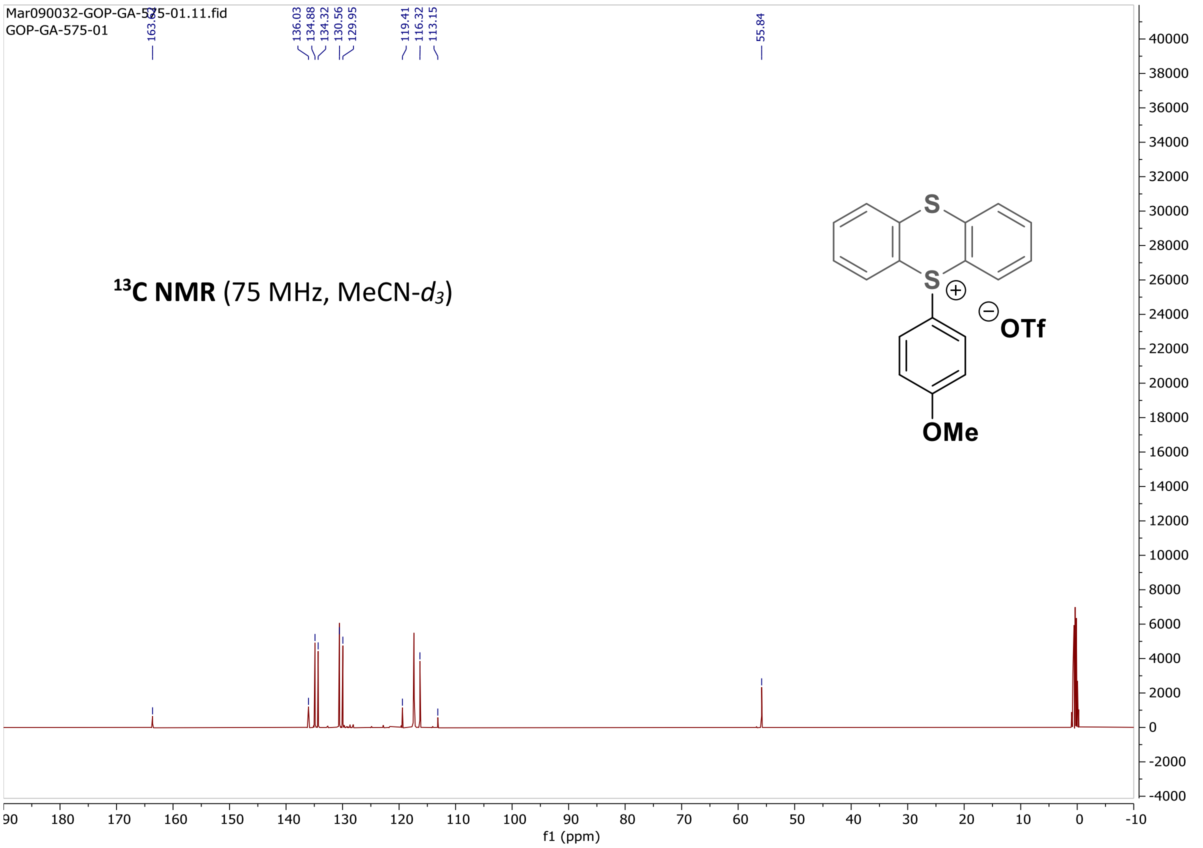

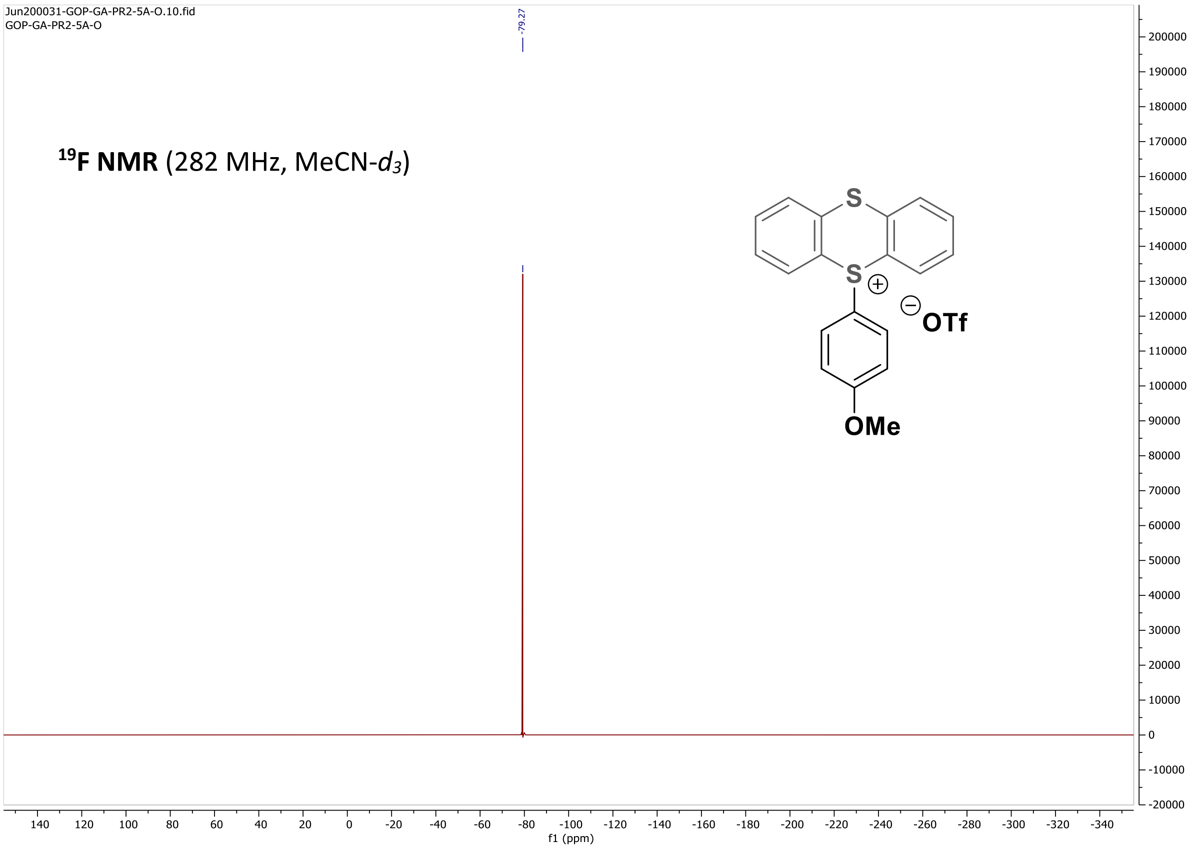
Jun200031-GOP-GA-PR2-5A-O.10.fid
GOP-GA-PR2-5A-O
-79.27
19F NMR (282 MHz, MeCN-d3)
S
S
OTf
OMe
f1 (ppm)

5-(4-(4-(2-(Pyridin-2-yloxy)propoxy)phenoxy)phenyl)-5*H*-thianthren-5-ium tetrafluoroborate (5b)

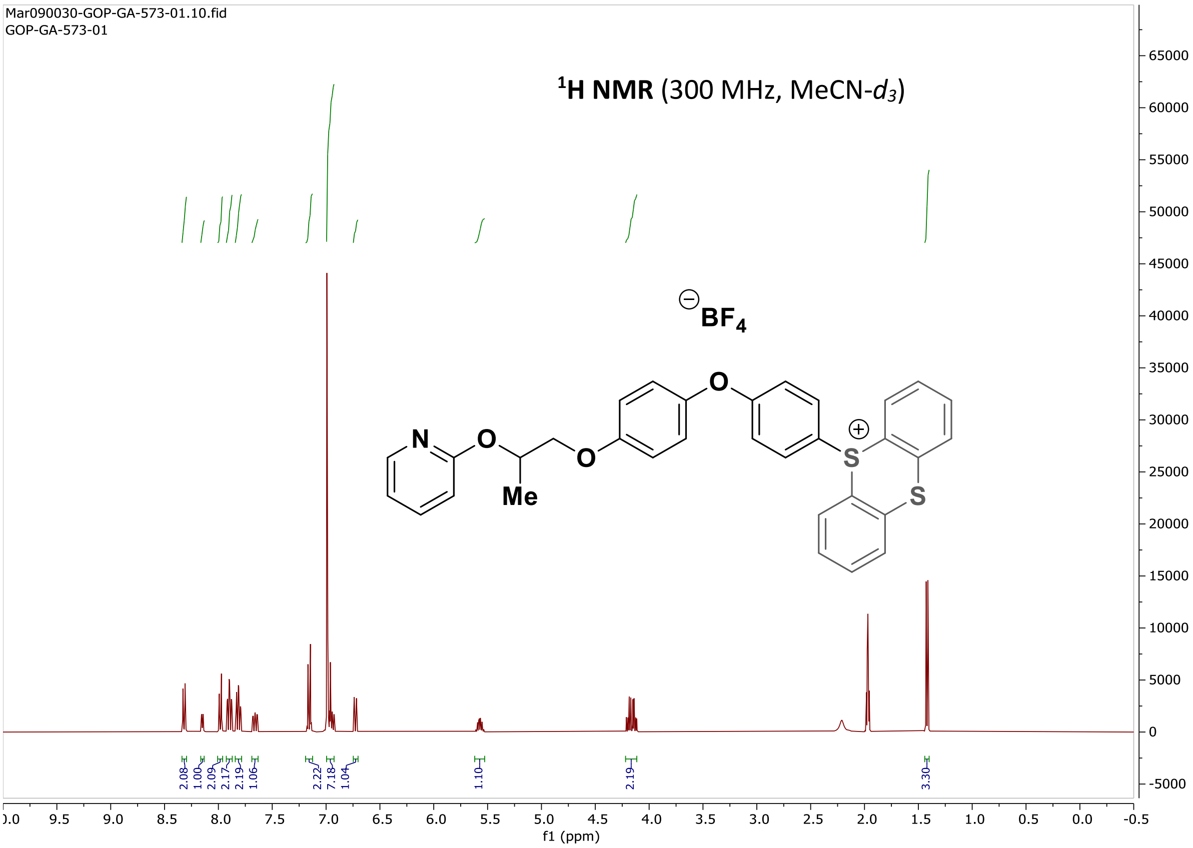

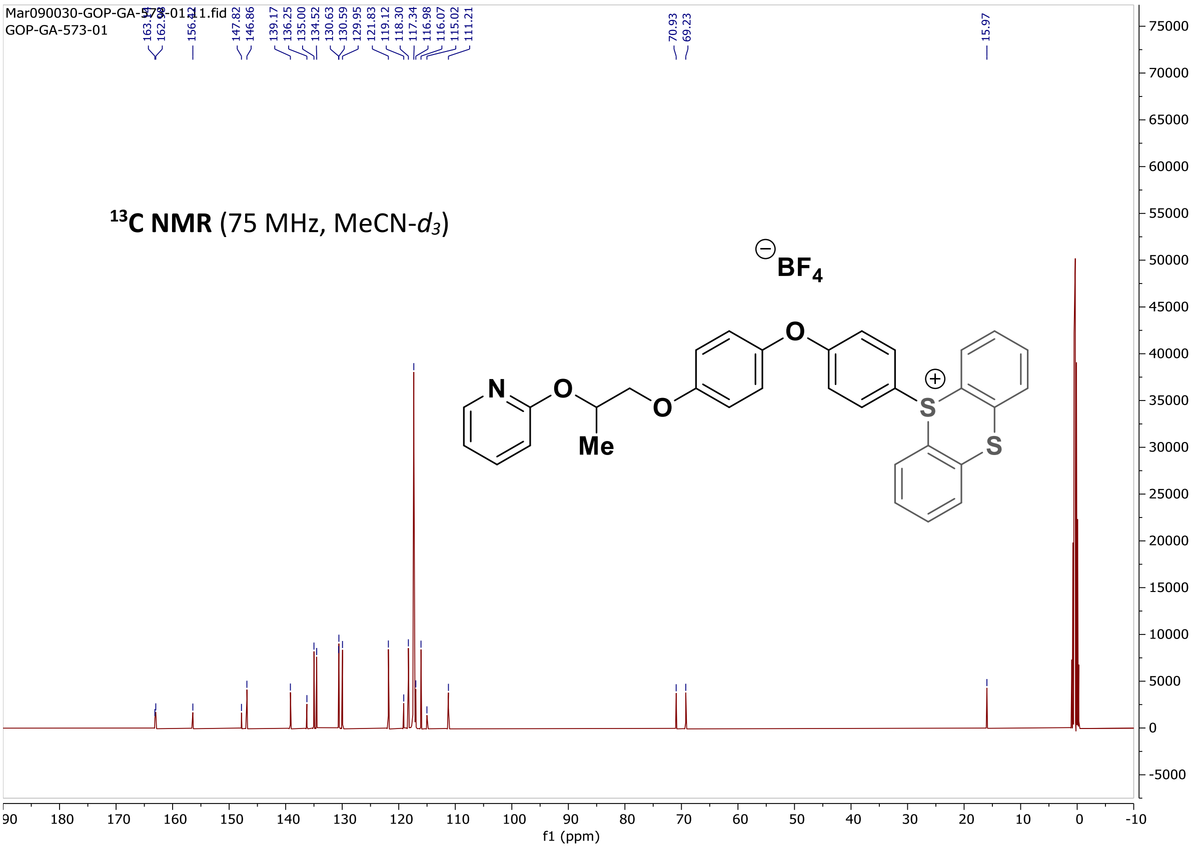

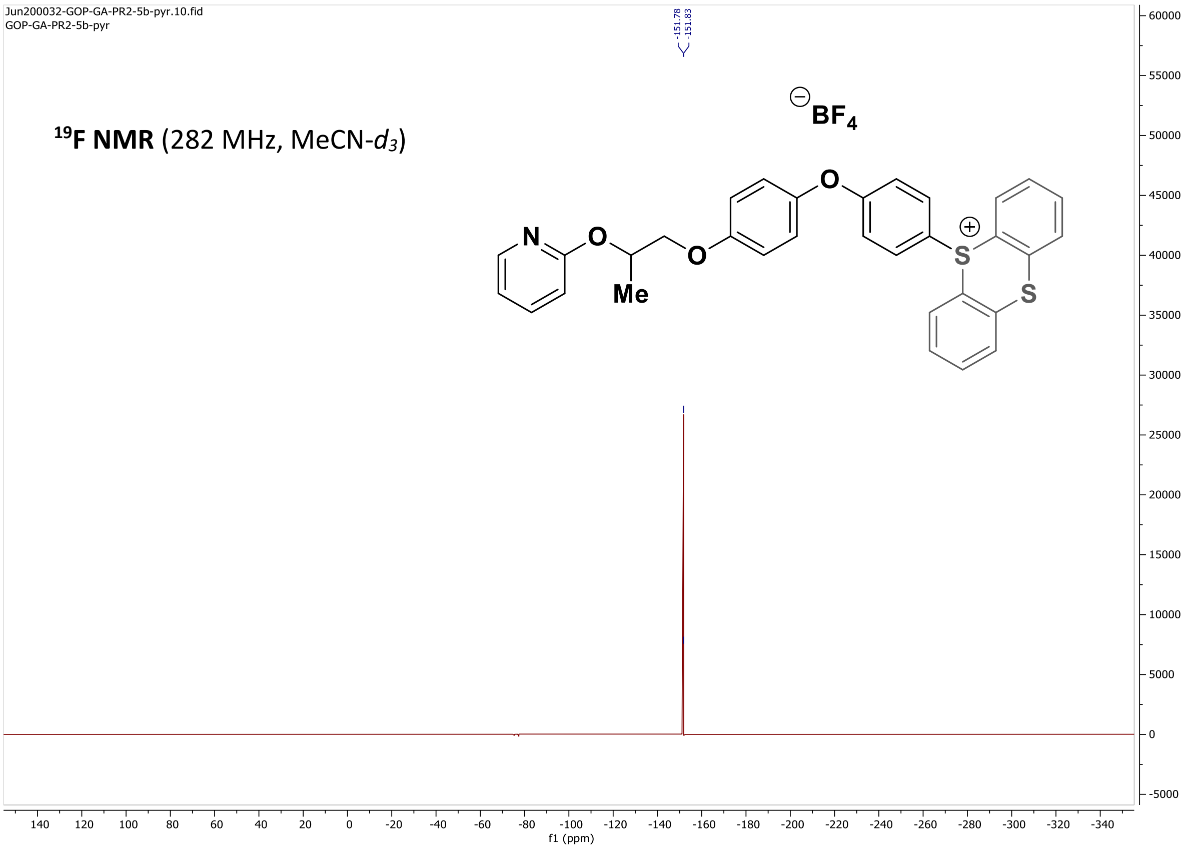

Jun200032-GOP-GA-PR2-5b-pyr.10.fid
GOP-GA-PR2-5b-pyr
-151.78
-151.83
19F NMR (282 MHz, MeCN-d3)
BF4
N
O
O
Me
O
S
S
f1 (ppm)

5-(4-((*N*,4-Dimethylphenyl)sulfonamido)phenyl)-5*H*-thianthren-5-ium tetrafluoroborate (5c)

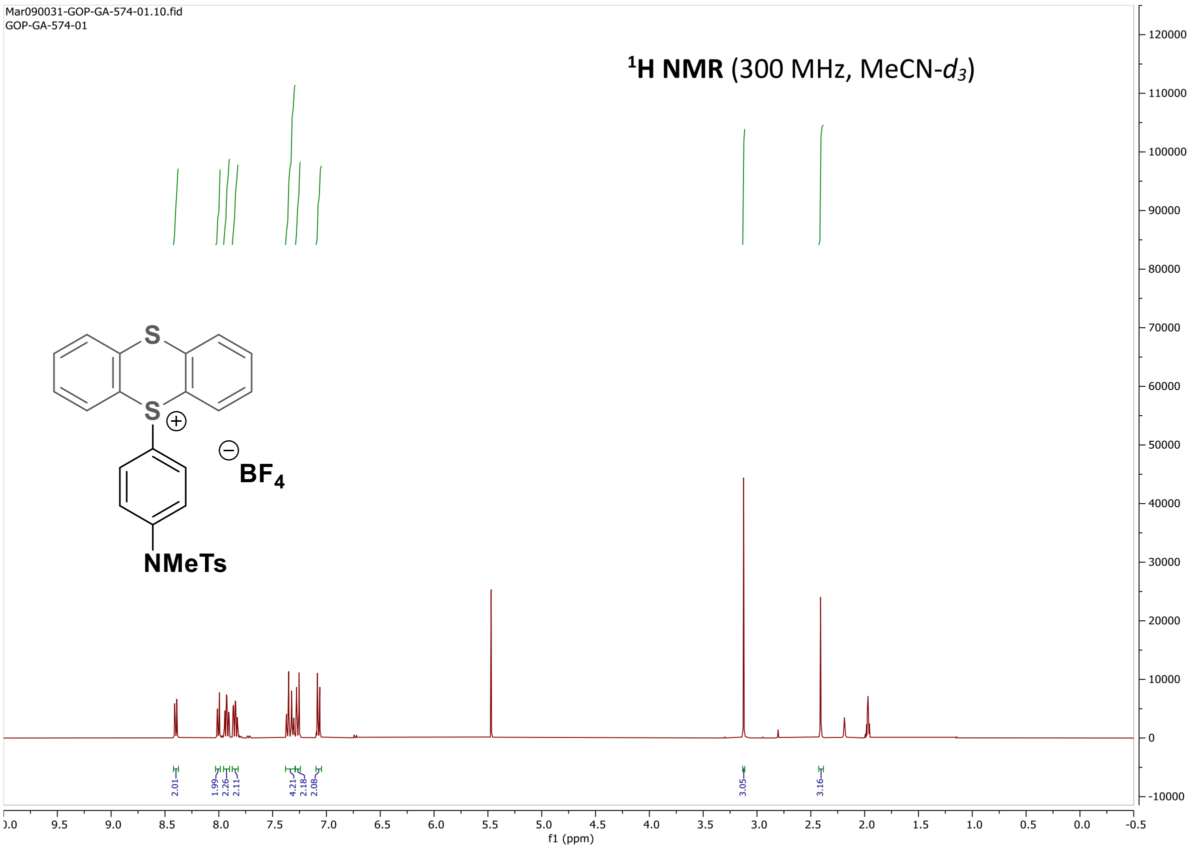

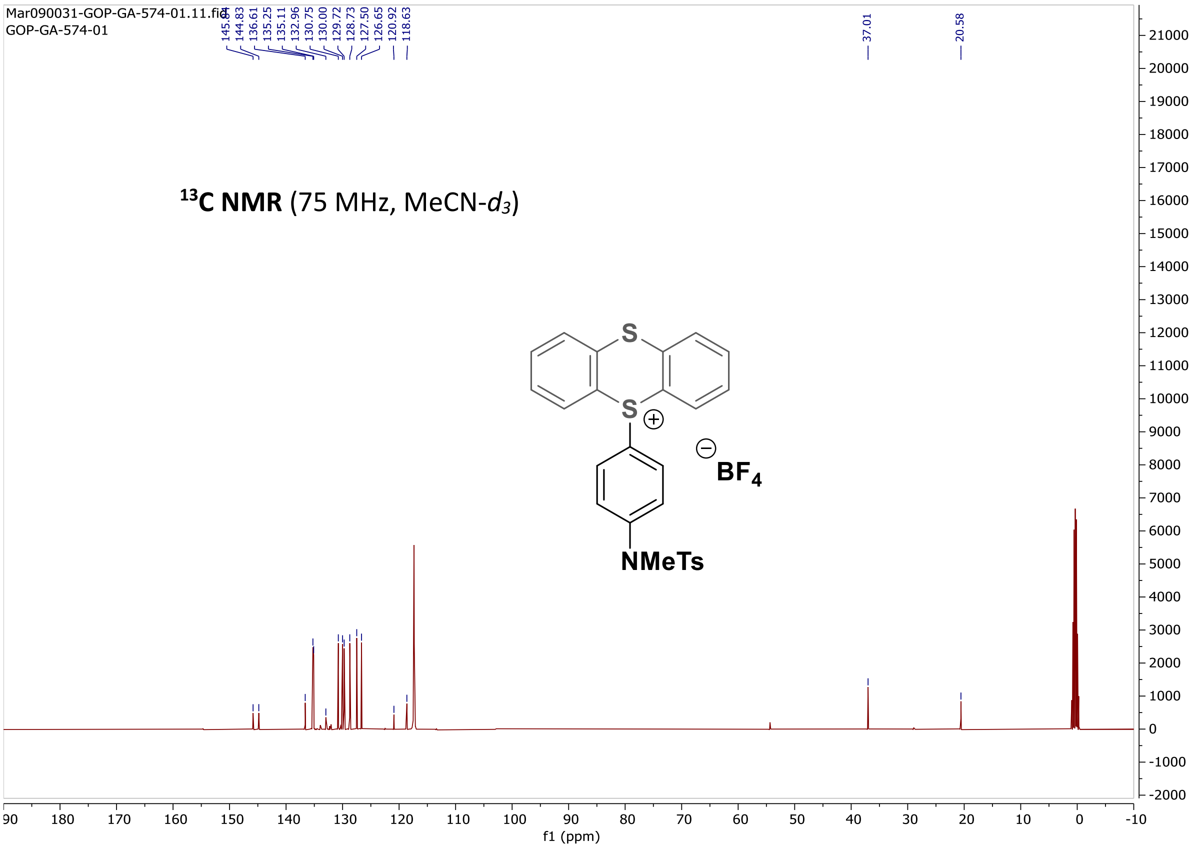

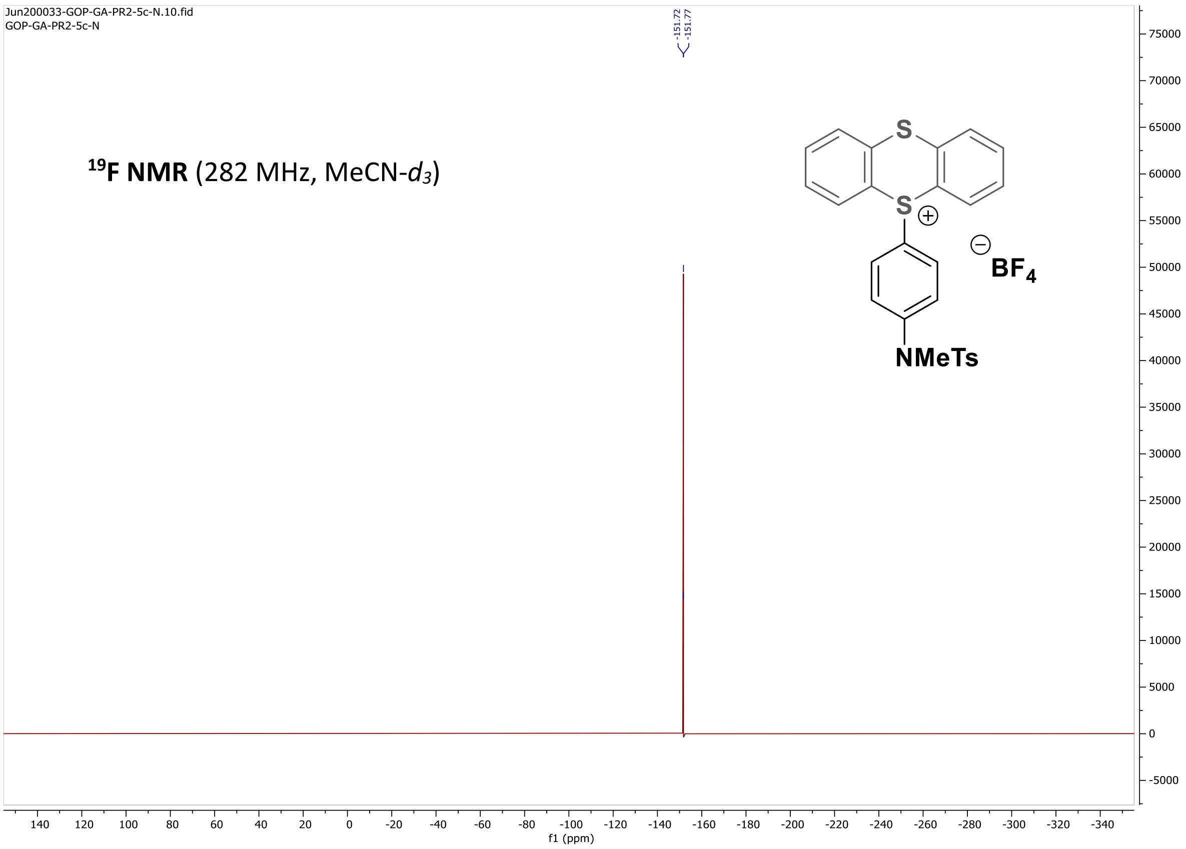
Jun200033-GOP-GA-PR2-5c-N.10.fid
GOP-GA-PR2-5c-N
-151.72
-151.77
19F NMR (282 MHz, MeCN-d3)
S
S
BF4
NMeTs
f1 (ppm)

[(2,6-(*t*BuNCH)$_2$C$_6$H$_3$)Bi(4-fluorophenyl)(tetrafluoroborate)] (3a)

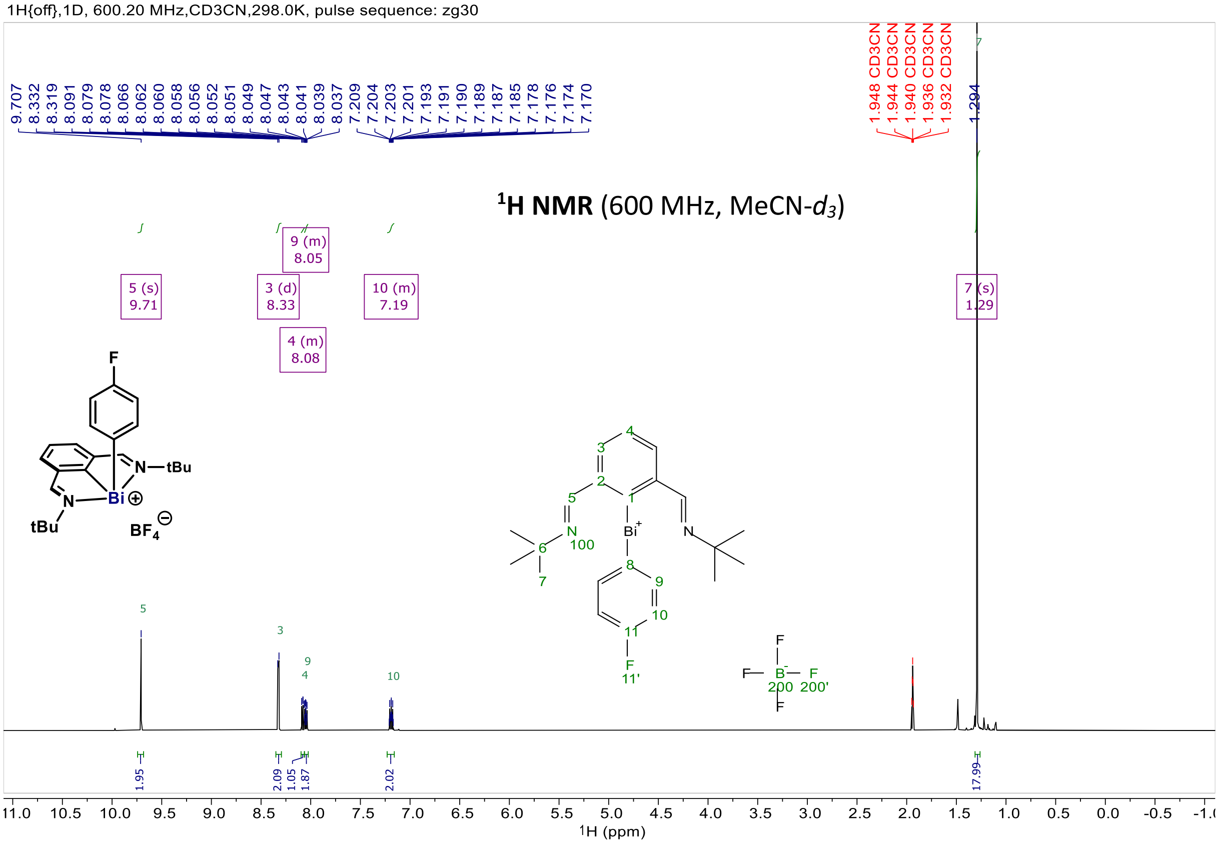

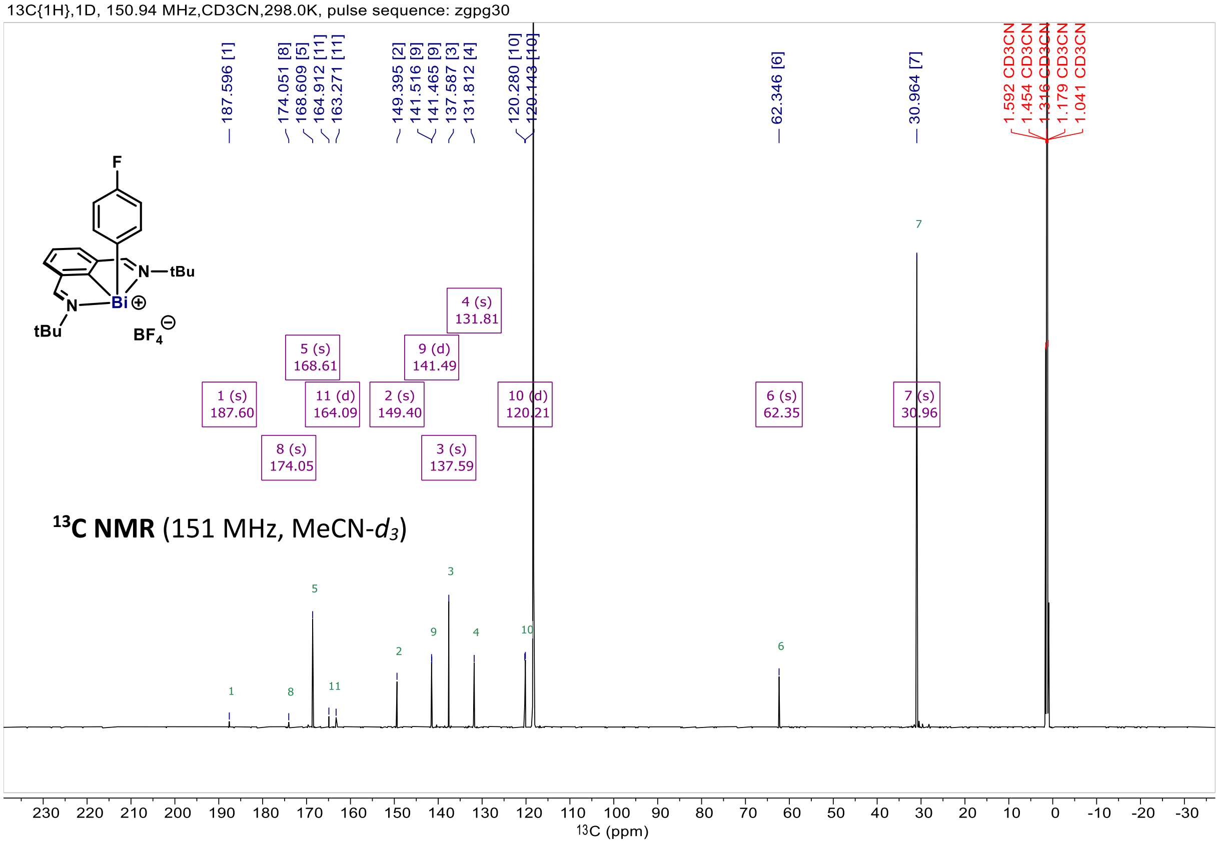

$^{1}H\{^{13}C\}$ HSQC

1H{13C},HSQC-EDITED, 600.20 MHz,CD3CN,298.0K, pulse sequence: hsqcedetgpsisp2.3

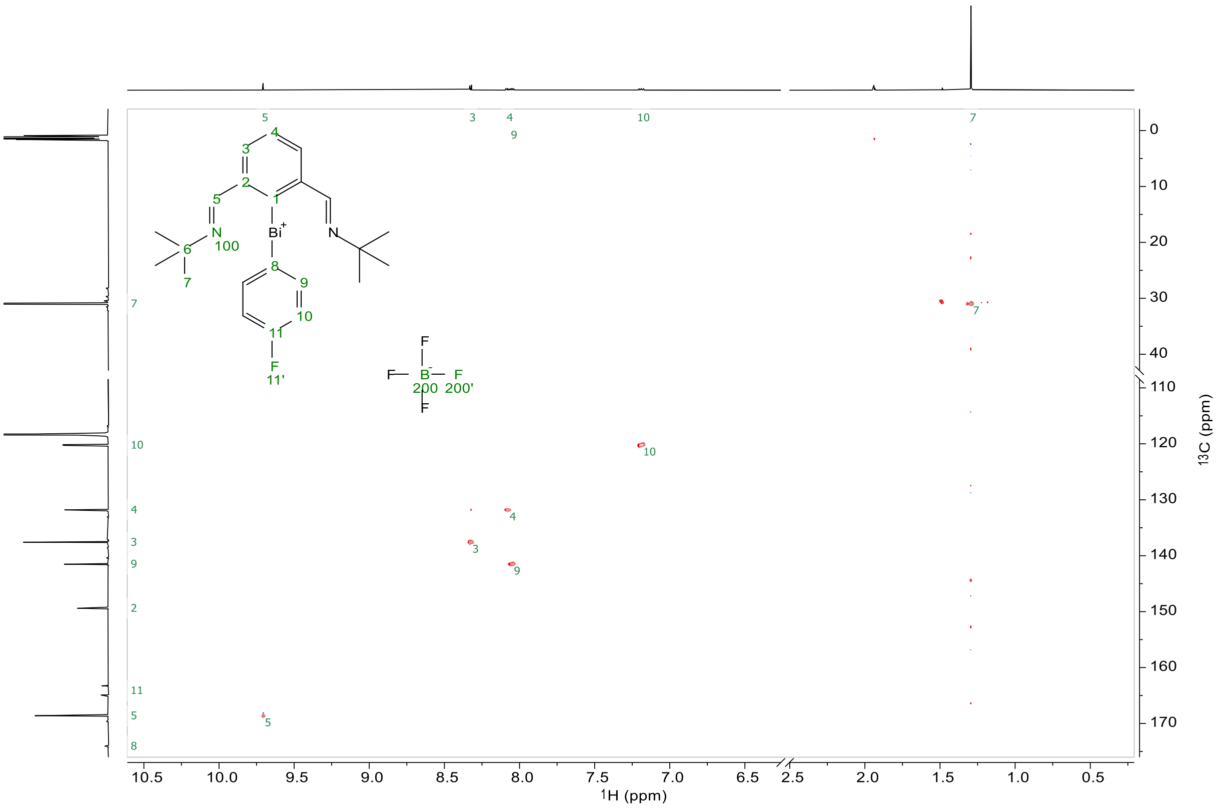

$^{1}H\{^{13}C\}$ HMBC

1H{13C},HMBC, 600.20 MHz,CD3CN,298.0K, pulse sequence: hmbcetgpl3nd

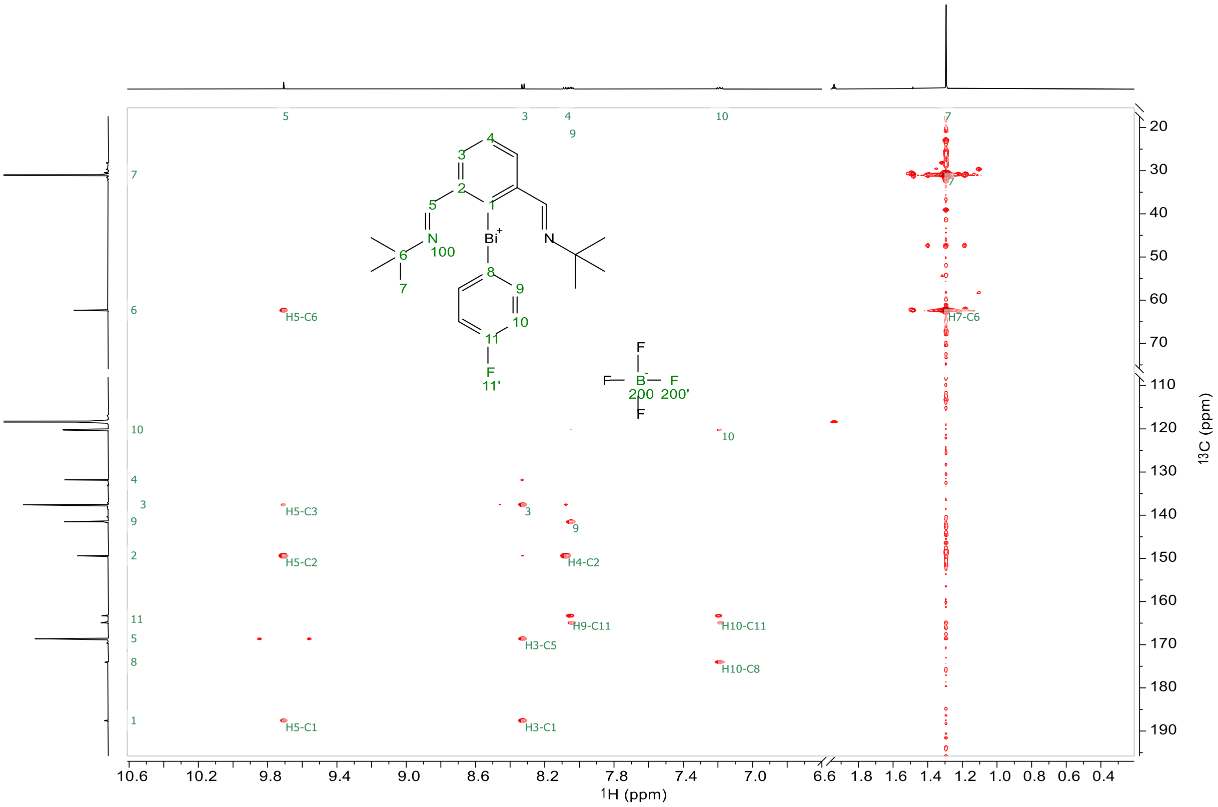

$^{1}H\{^{13}C\}$ HMBC

1H{13C},HMBC, 600.20 MHz,CD3CN,298.0K, pulse sequence: hmbcetgpl3nd

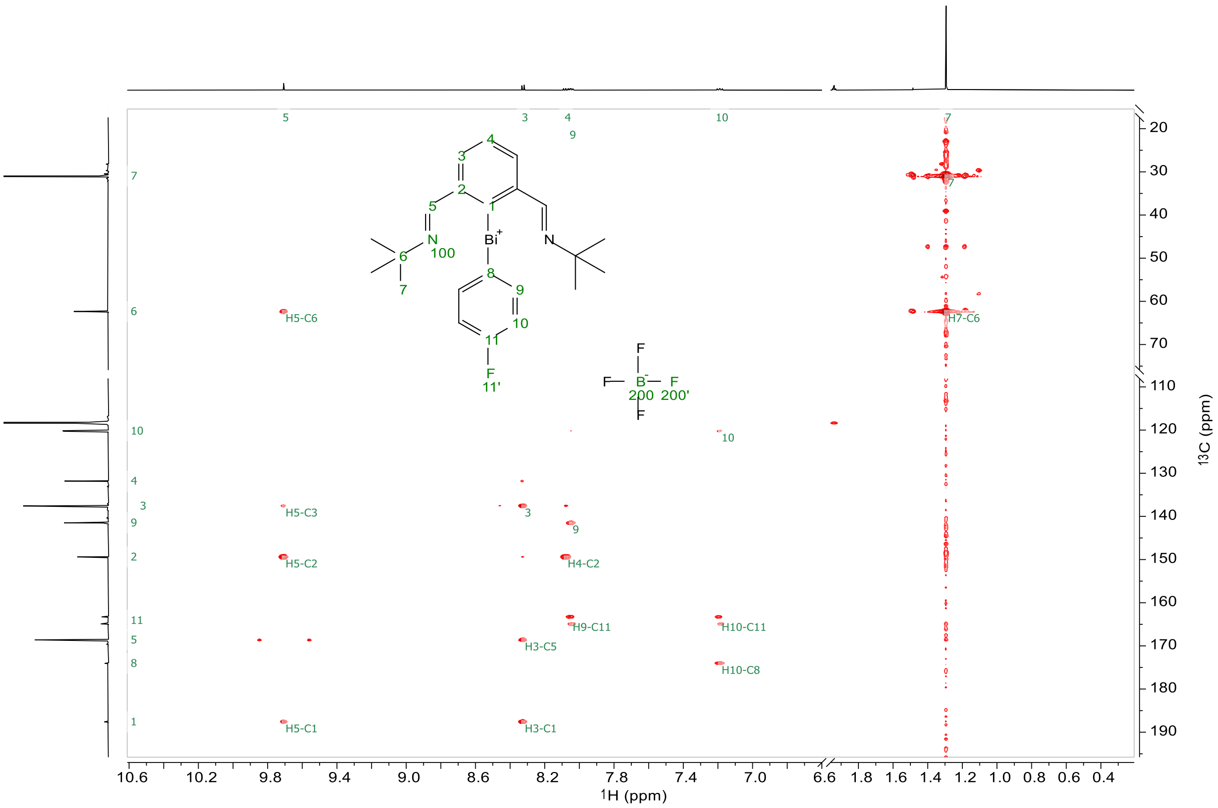

^{1}H COSY

1H{off},COSY, 600.20 MHz,CD3CN,298.0K, pulse sequence: cosygpppqf

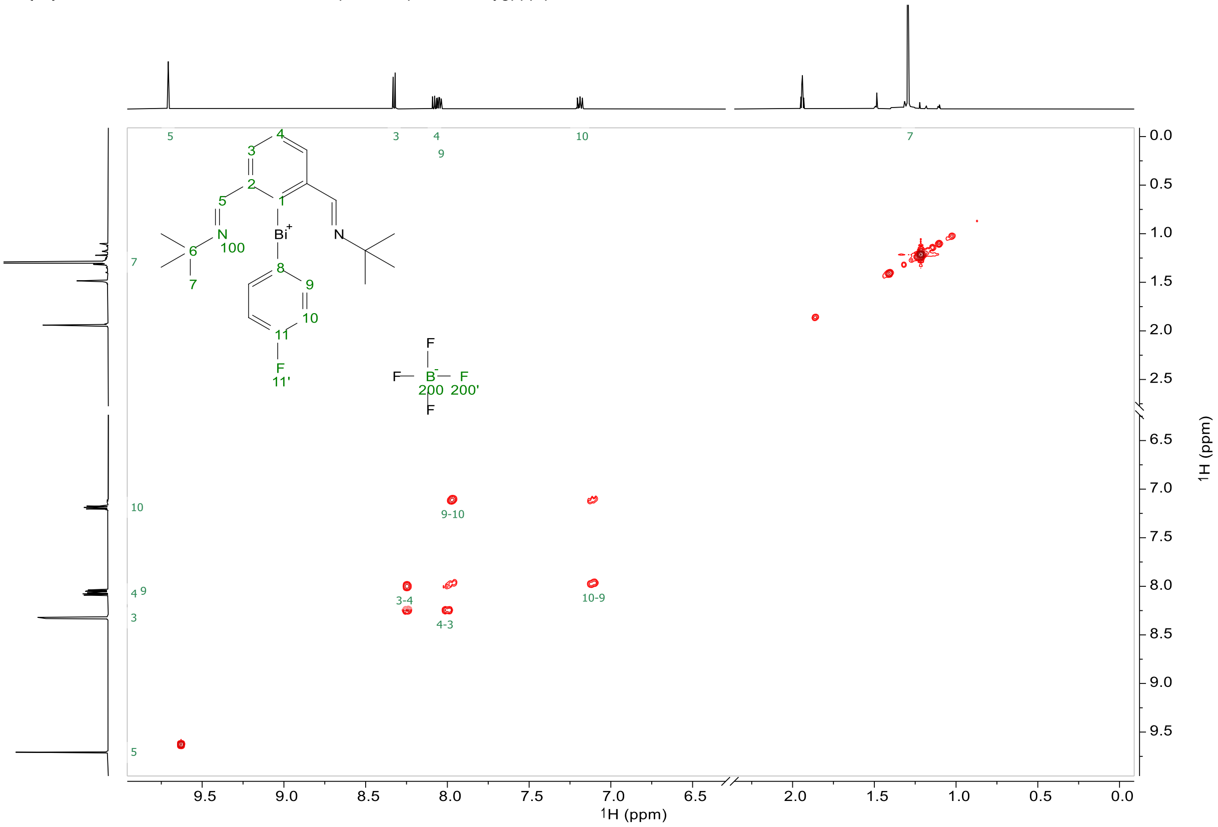

^{1}H NOESY

1H{off},NOESY, 600.20 MHz,CD3CN,298.0K, pulse sequence: noesygpphpp

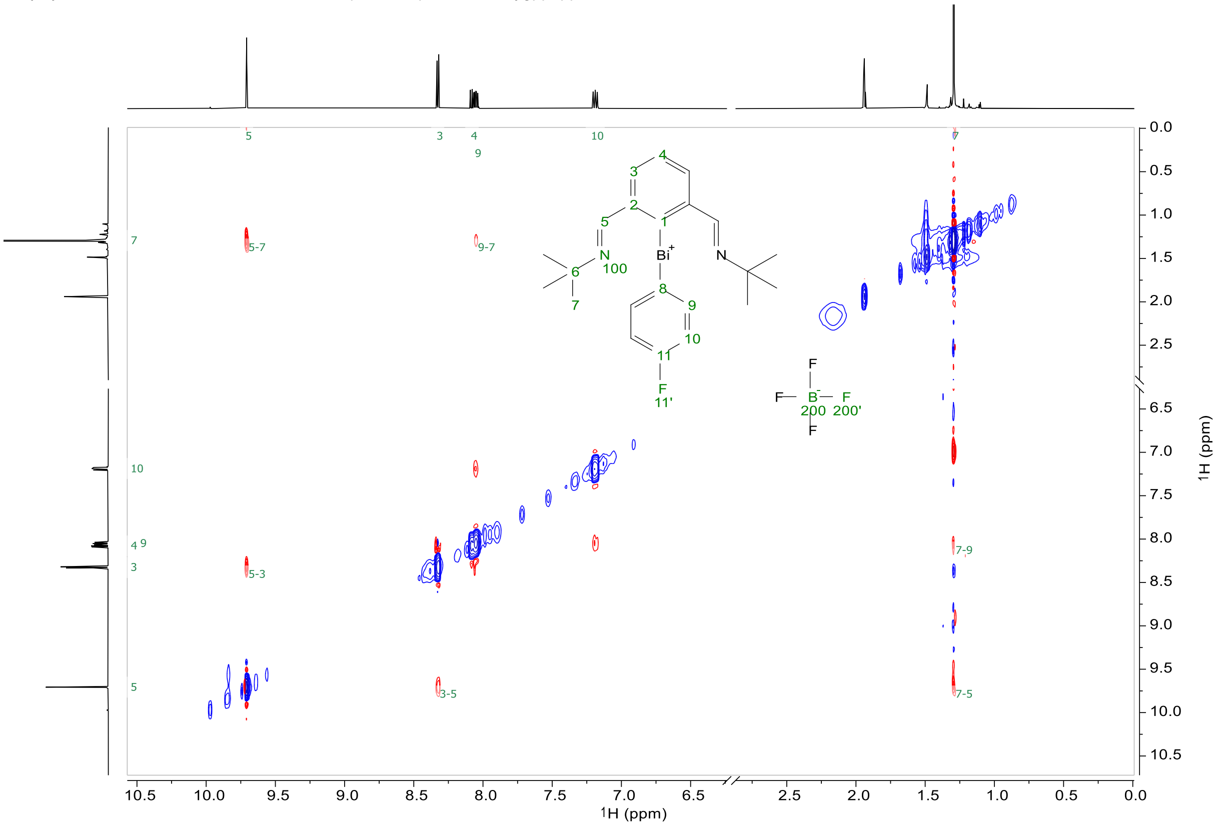

^{19}F NMR

19F{off},1D, 564.72 MHz,CD3CN,298.0K, pulse sequence: zg30

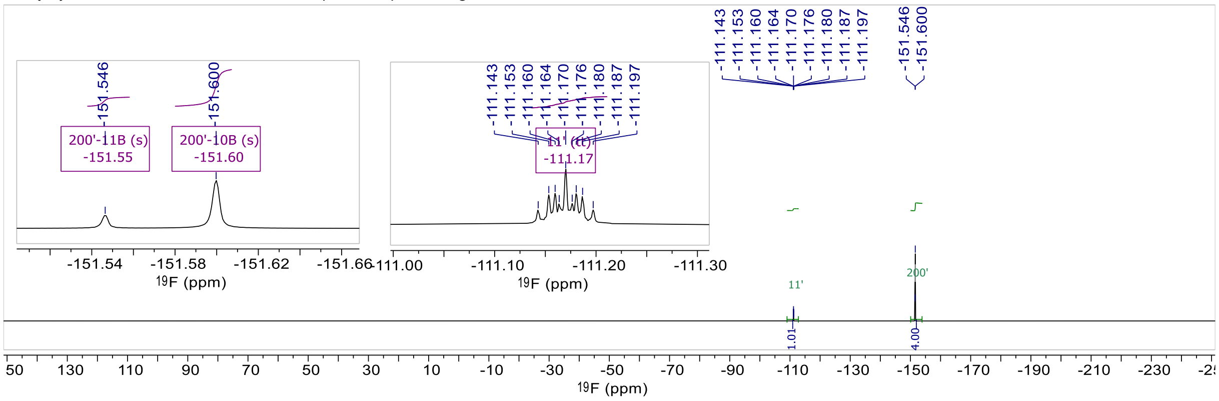

^{1}H{^{15}N} HMBC

1H{15N},HMBC, 600.20 MHz,CD3CN,298.0K, pulse sequence: hmbcgpndqf

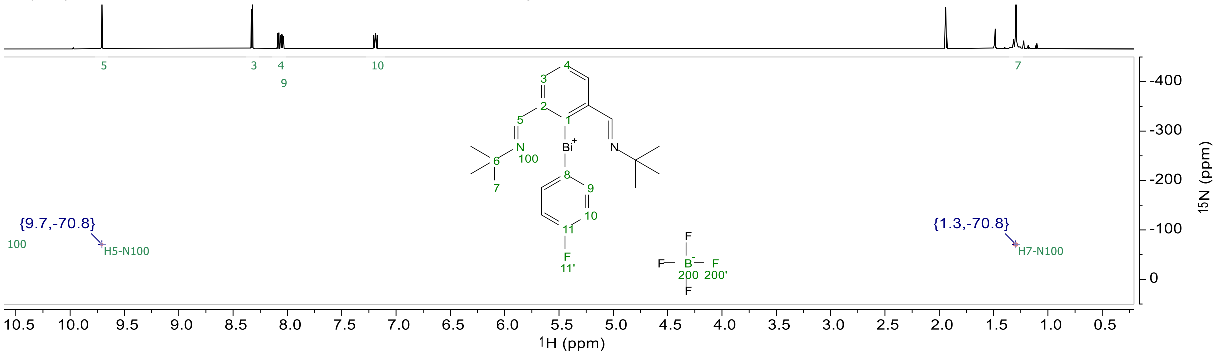

[(2,6-(CyNCH)$_2$C$_6$H$_3$)Bi(4-fluorophenyl)(tetrafluoroborate)] (3b)

1H{off},1D, 600.20 MHz,CD3CN,298.0K, pulse sequence: zg30

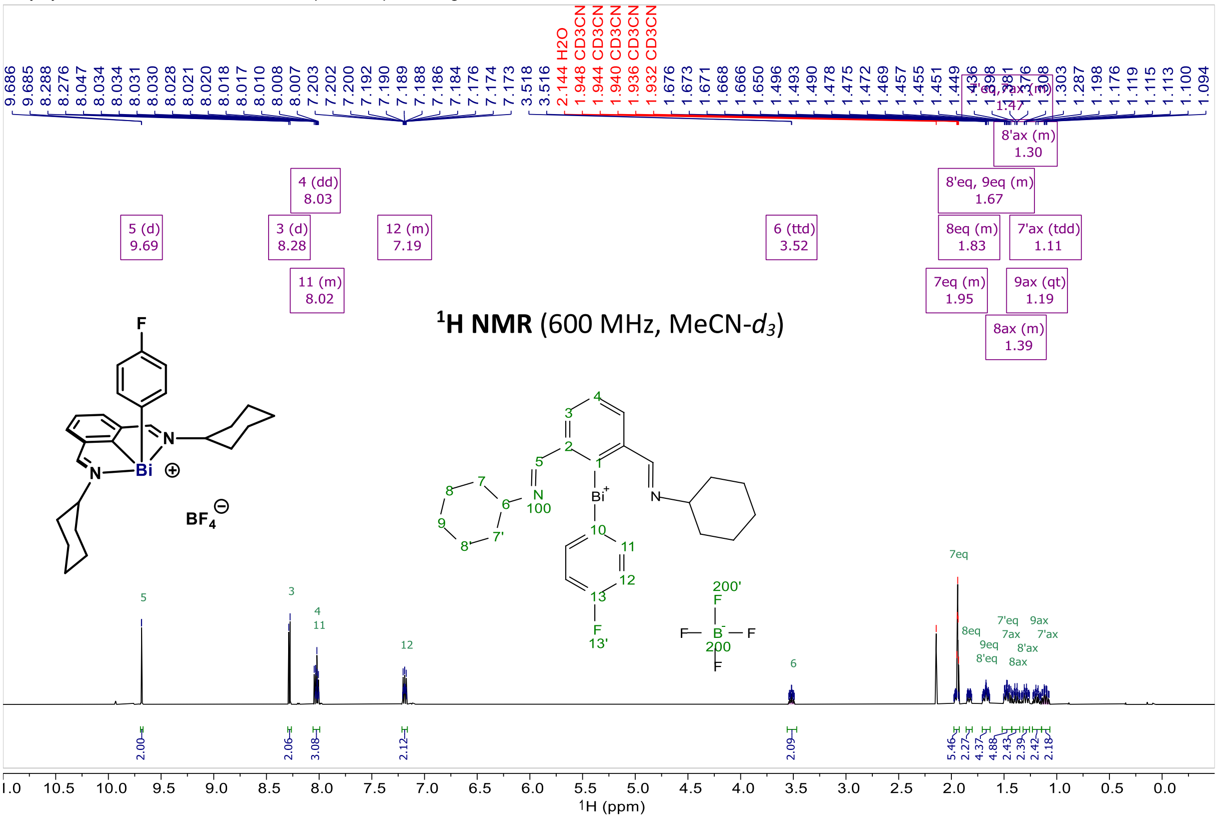

13C{1H},1D, 150.94 MHz,CD3CN,298.0K, pulse sequence: zgpg30

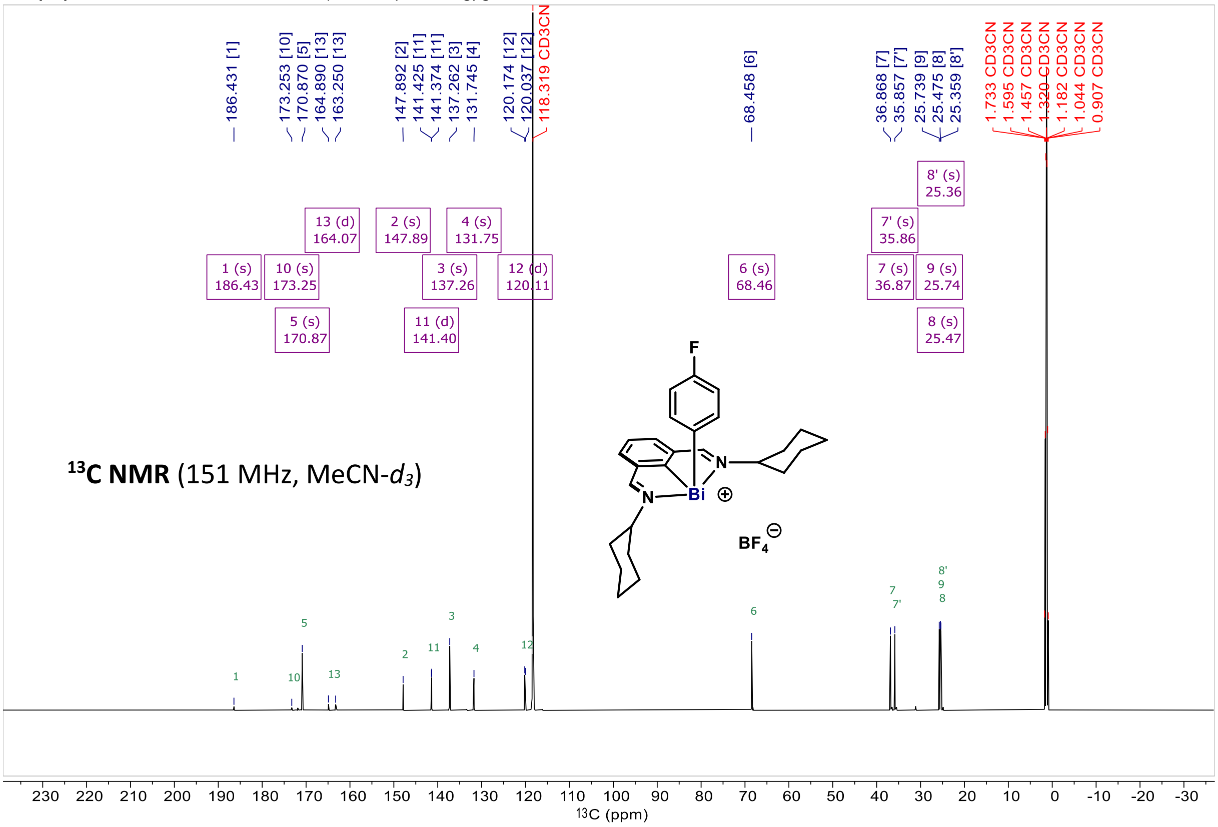

19F{off},1D, 564.72 MHz,CD3CN,298.0K, pulse sequence: zg30

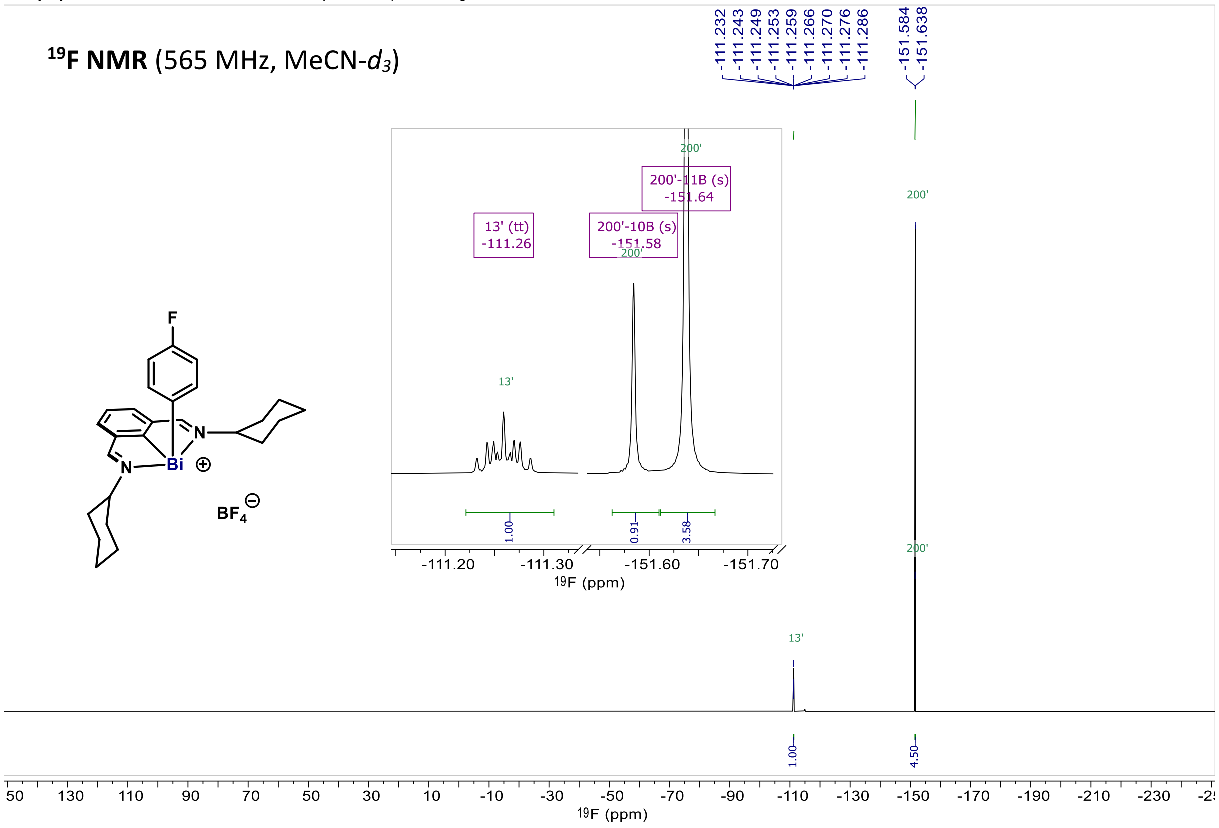

11B{off},1D, 192.57 MHz,CD3CN,298.0K, pulse sequence: zg30

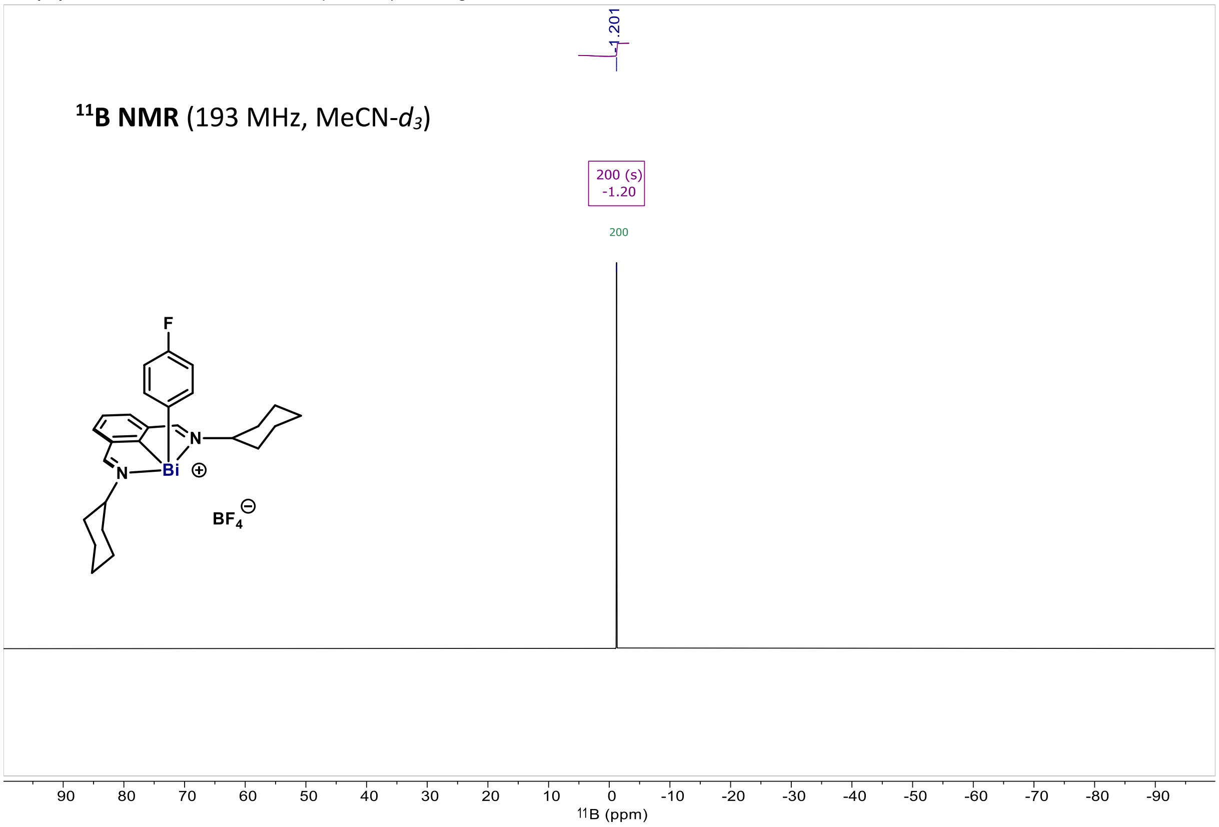

[(Phebox)Bi(4-methoxyphenyl)(tetrafluoroborate)] (3c)

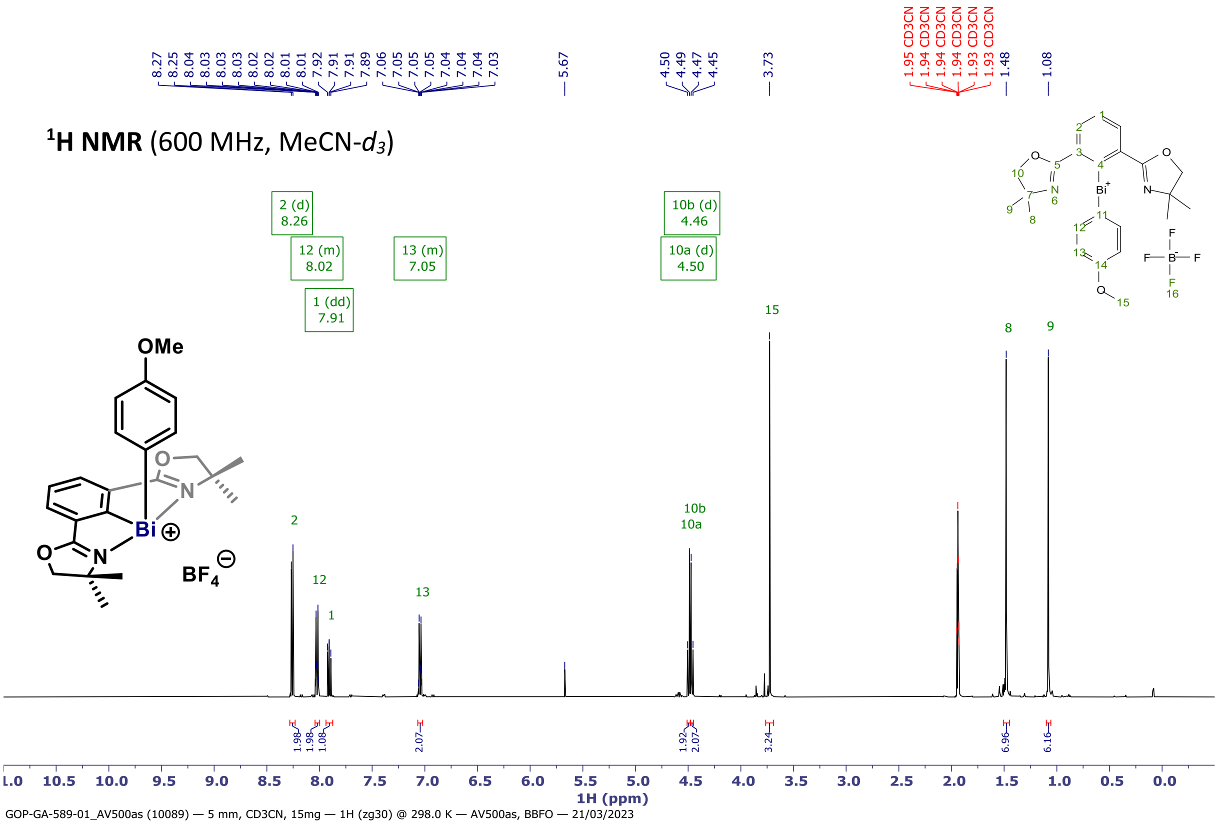

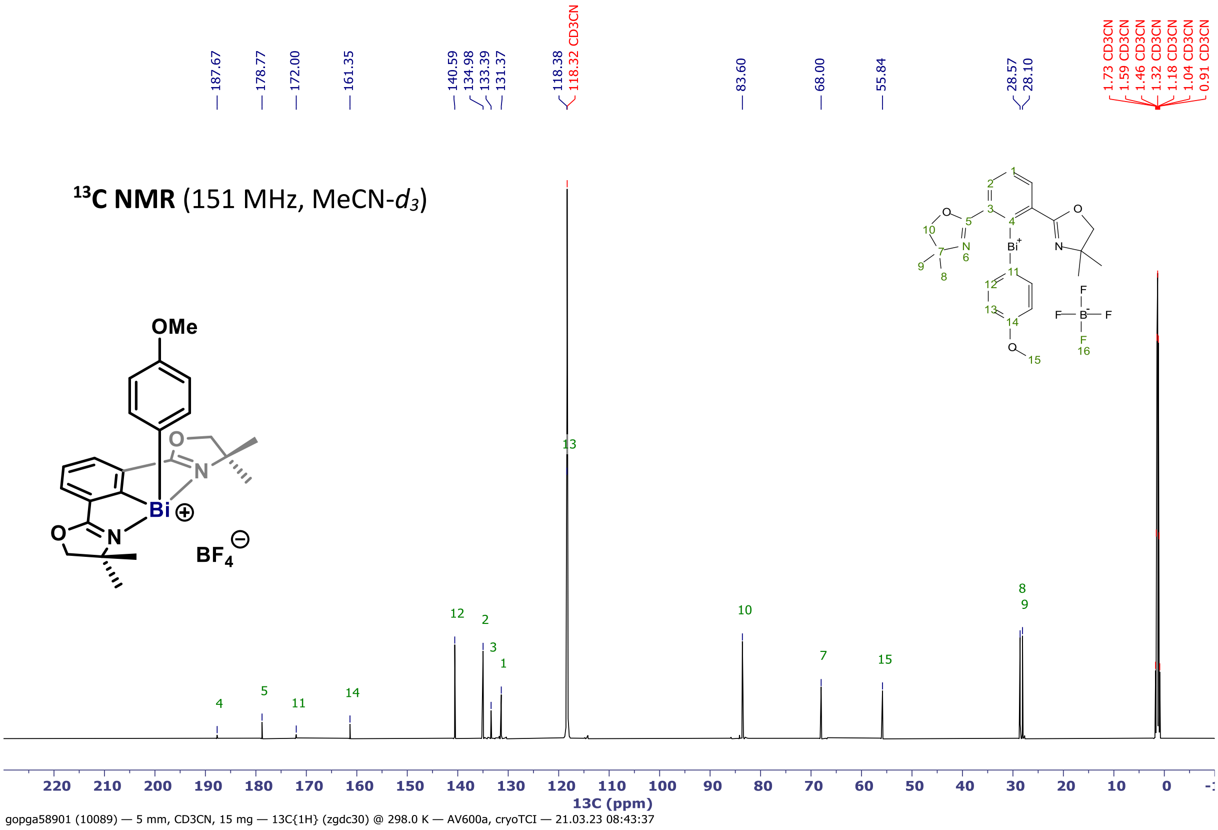

^{19}F NMR (470 MHz, MeCN-d_3)

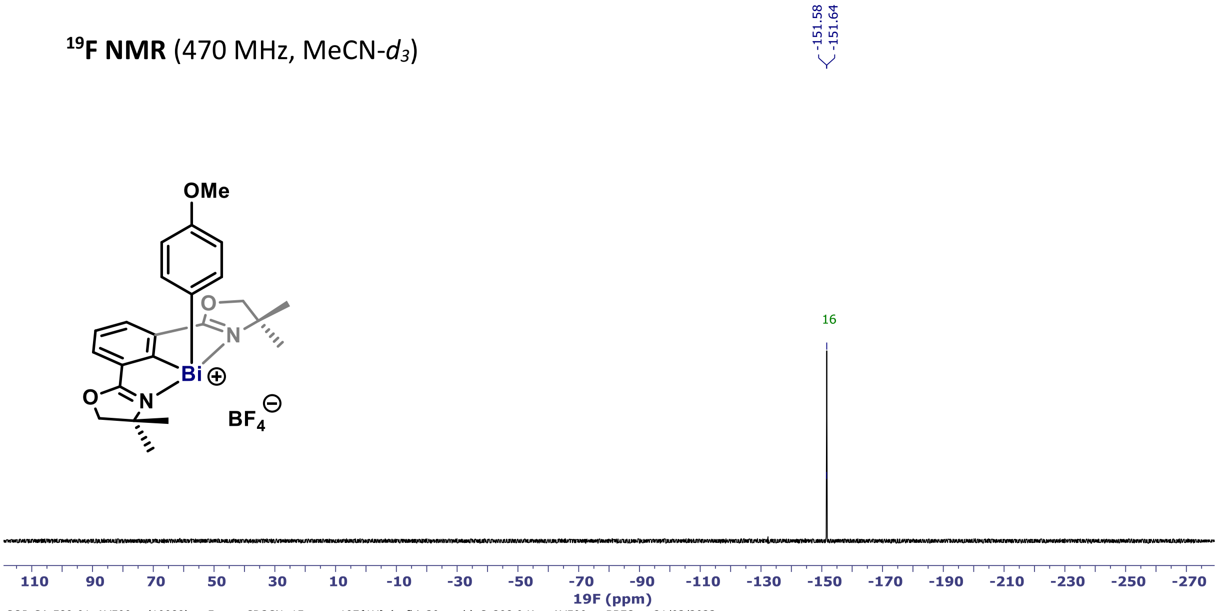

GOP-GA-589-01_AV500as (10089) — 5 mm, CD3CN, 15mg — 19F{1H} (zgfhig30qn.ph) @ 298.0 K — AV500as, BBFO — 21/03/2023

[(2,6-(*t*BuNCH)$_2$C$_6$H$_3$)Bi(4-methoxyphenyl)(tetrafluoroborate)] (3d)

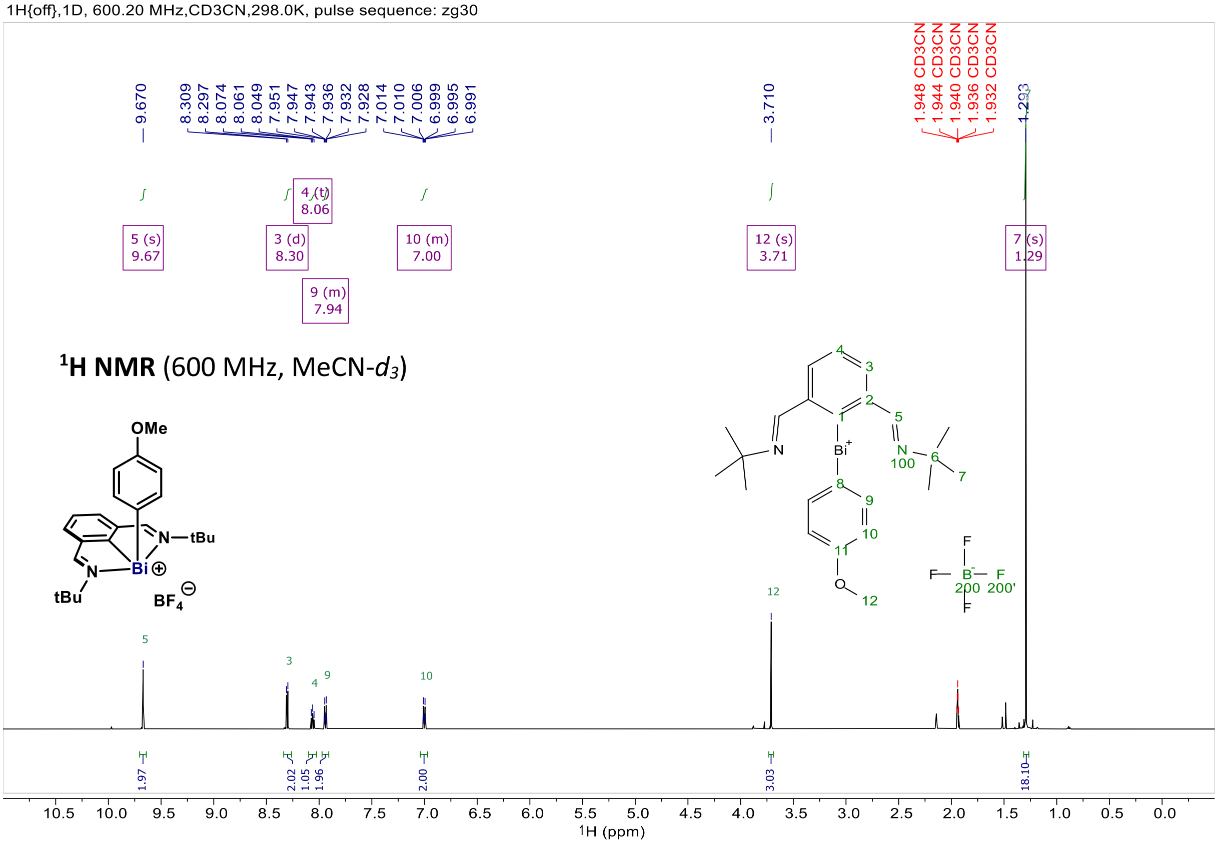

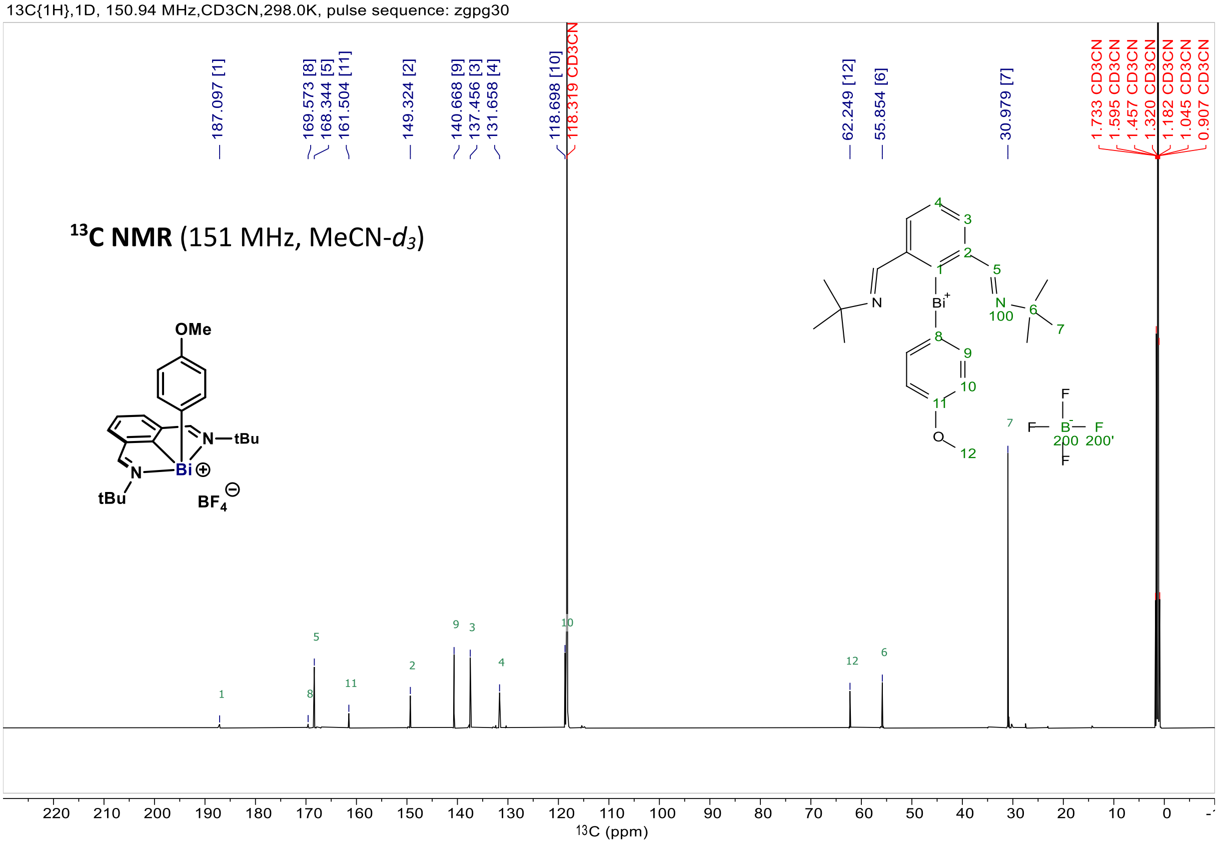

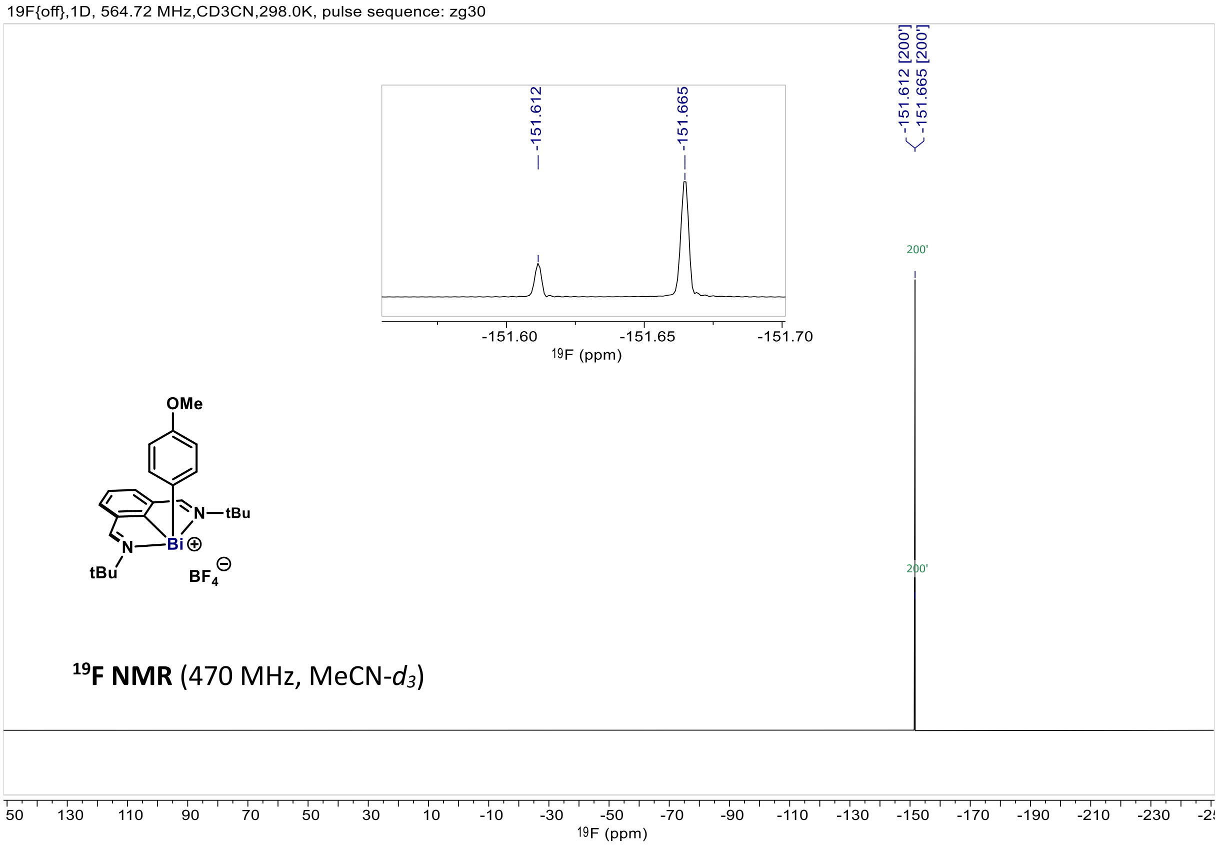
19F{off},1D, 564.72 MHz,CD3CN,298.0K, pulse sequence: zg30
-151.612 [200']
-151.665 [200']
-151.612
-151.665
-151.60
-151.65
-151.70
19F (ppm)
OMe
N—tBu
N
Bi
tBu
BF4
19F NMR (470 MHz, MeCN-d3)
19F (ppm)

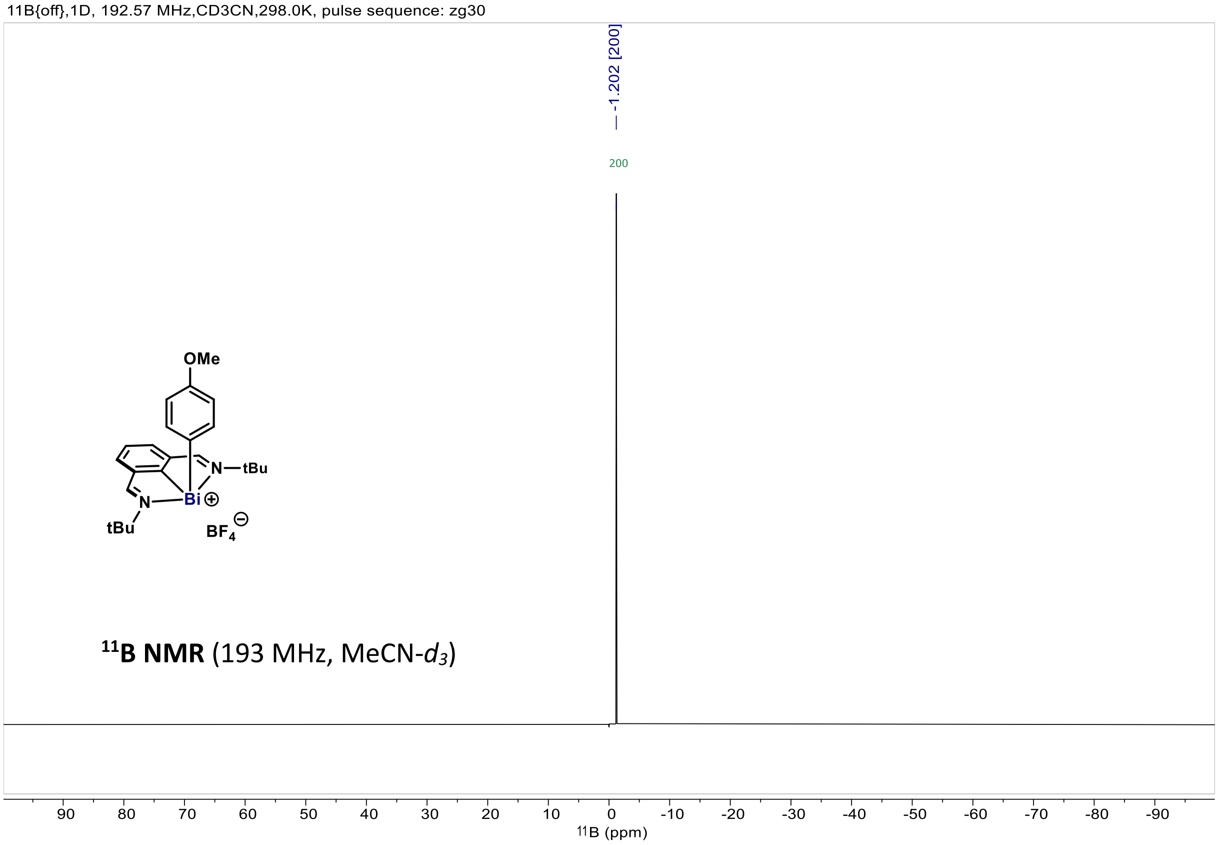
11B{off},1D, 192.57 MHz,CD3CN,298.0K, pulse sequence: zg30
-1.202 [200]
OMe
N—tBu
N
Bi
tBu
BF4
11B NMR (193 MHz, MeCN-d3)
11B (ppm)

[(2,6-(*t*BuNCH)$_2$C$_6$H$_3$)Bi(4-benzonitrile)(tetrafluoroborate)] (3e)

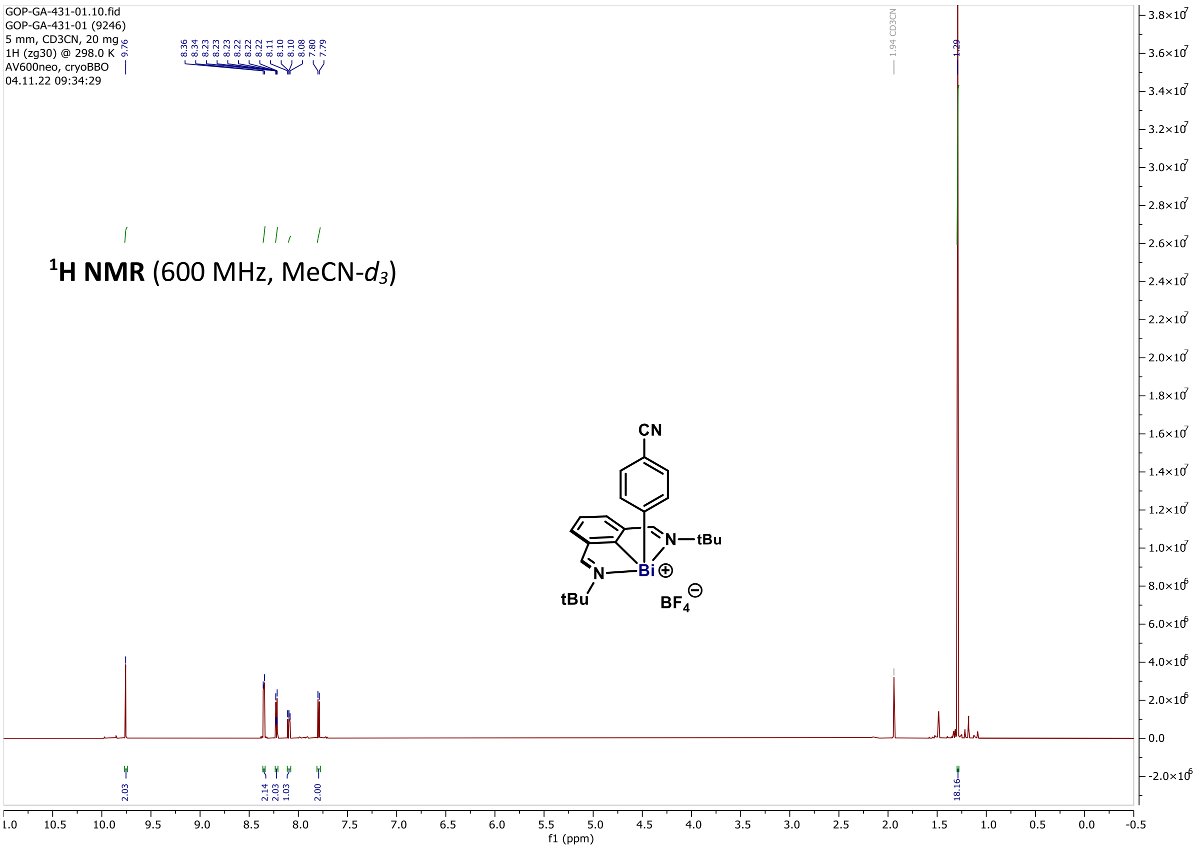

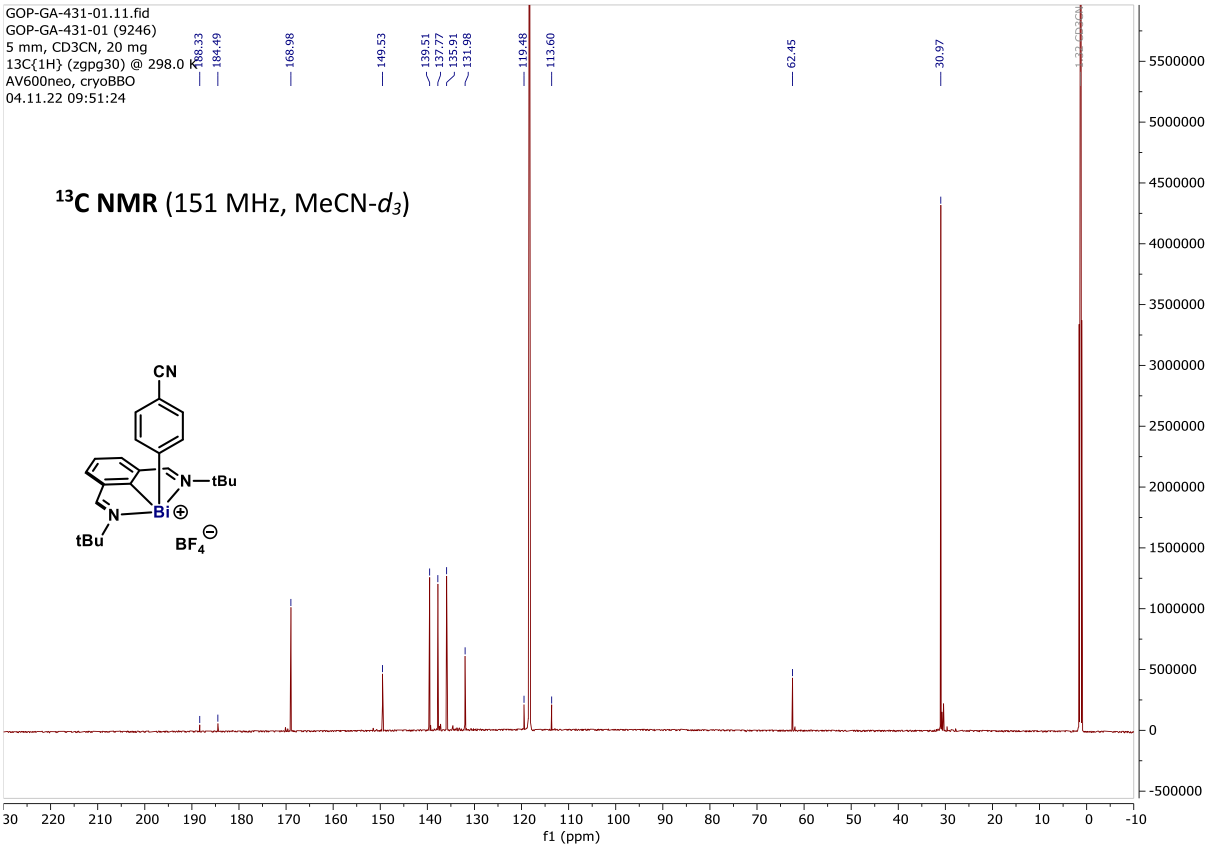

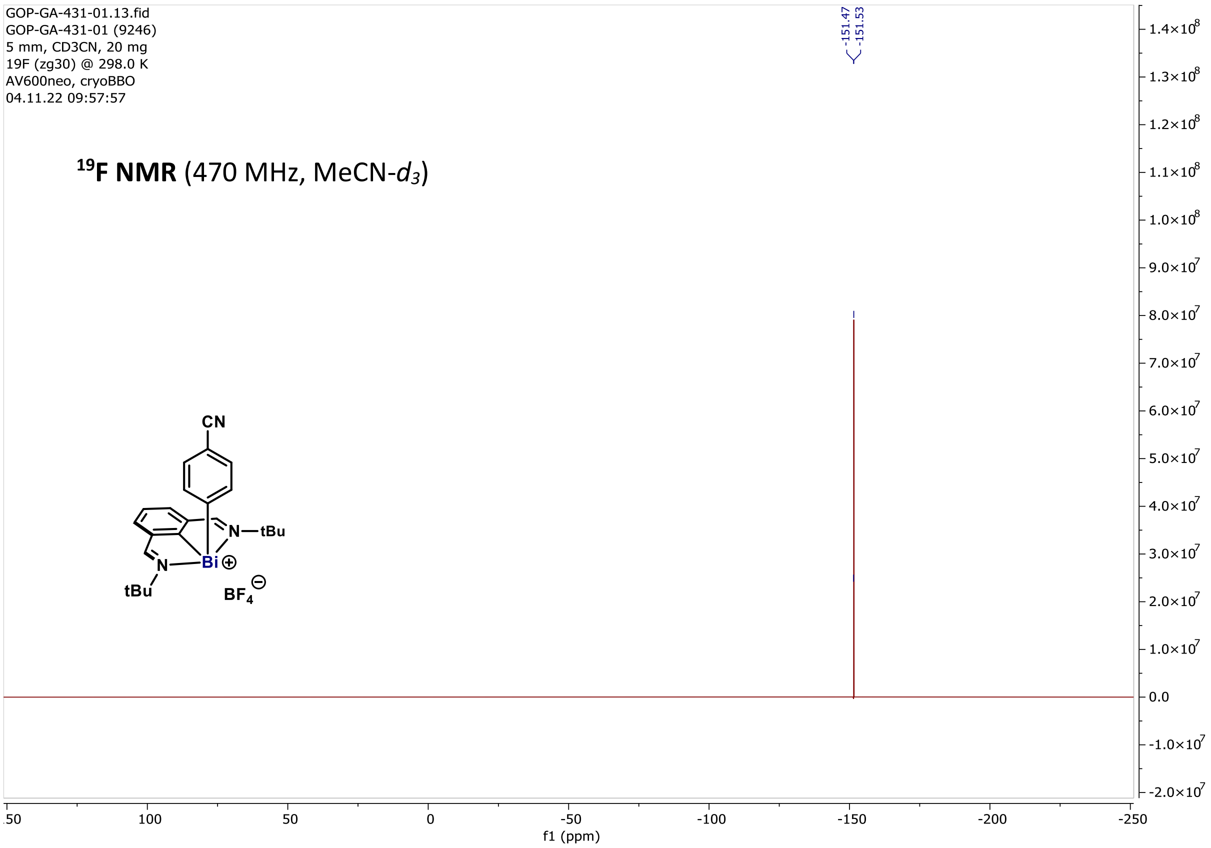
GOP-GA-431-01.13.fid
GOP-GA-431-01 (9246)
5 mm, CD3CN, 20 mg
19F (zg30) @ 298.0 K
AV600neo, cryoBBO
04.11.22 09:57:57
^{19}F NMR (470 MHz, MeCN-d_3)
-151.47
-151.53
CN
N—tBu
N
Bi
tBu
BF_4
f1 (ppm)

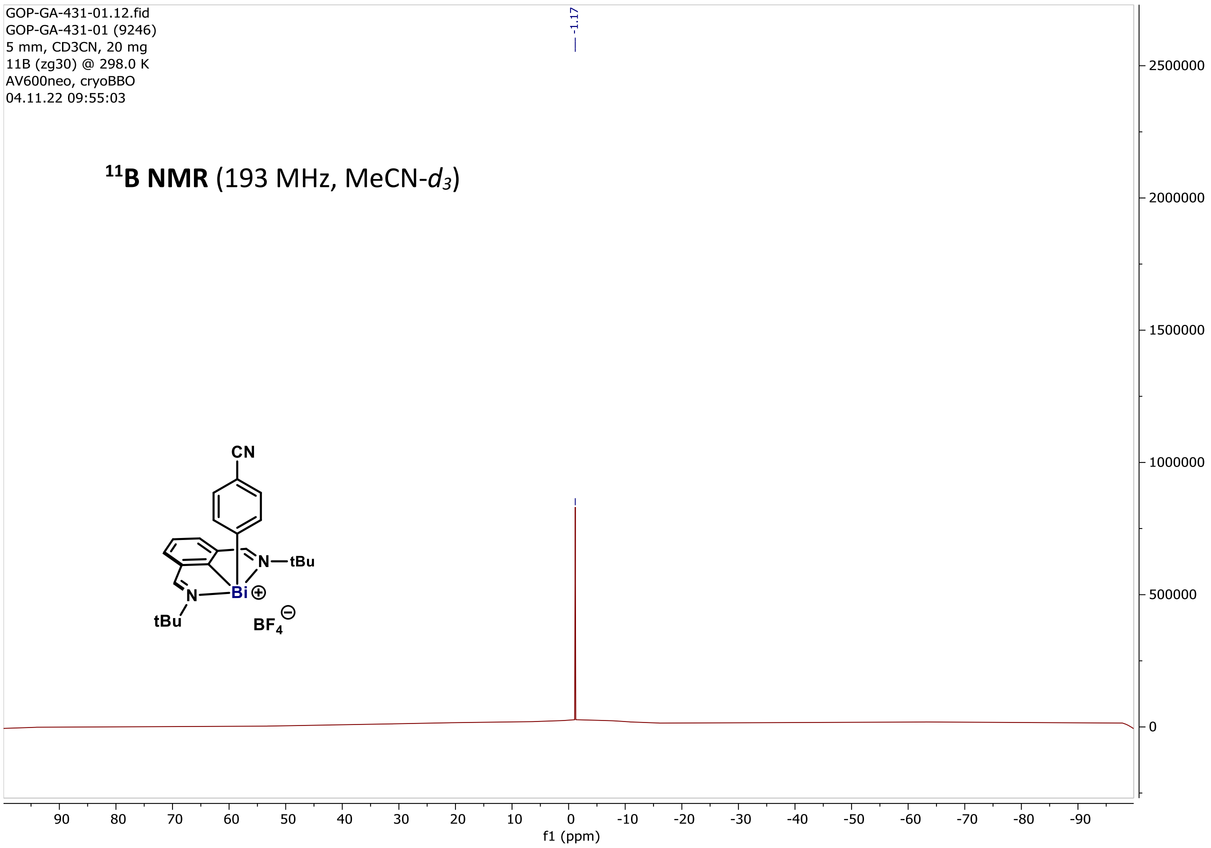
GOP-GA-431-01.12.fid
GOP-GA-431-01 (9246)
5 mm, CD3CN, 20 mg
11B (zg30) @ 298.0 K
AV600neo, cryoBBO
04.11.22 09:55:03
^{11}B NMR (193 MHz, MeCN-d_3)
-1.17
CN
N—tBu
N
Bi
tBu
BF_4
f1 (ppm)

[(2,6-(*t*BuNCH)$_2$C$_6$H$_3$)Bi(4-bromophenyl)(tetrafluoroborate)] (3f)

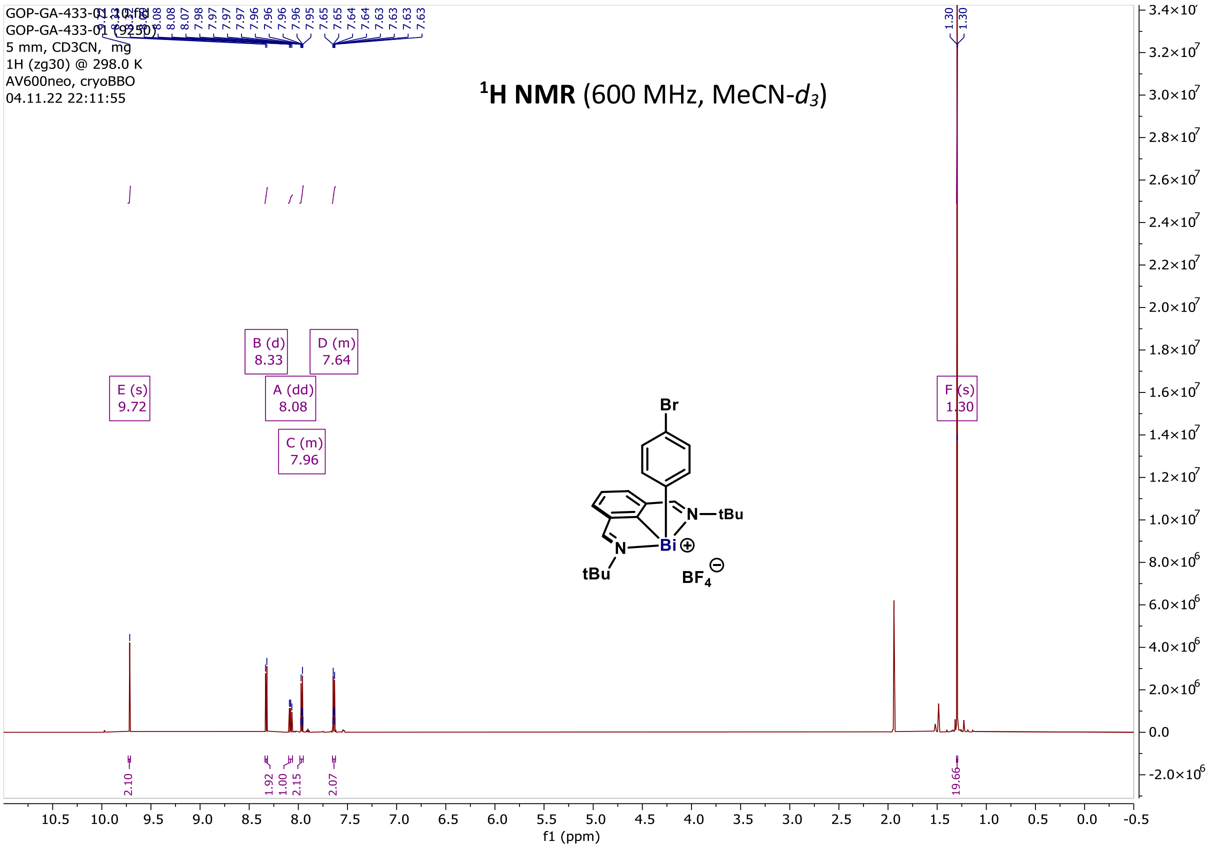

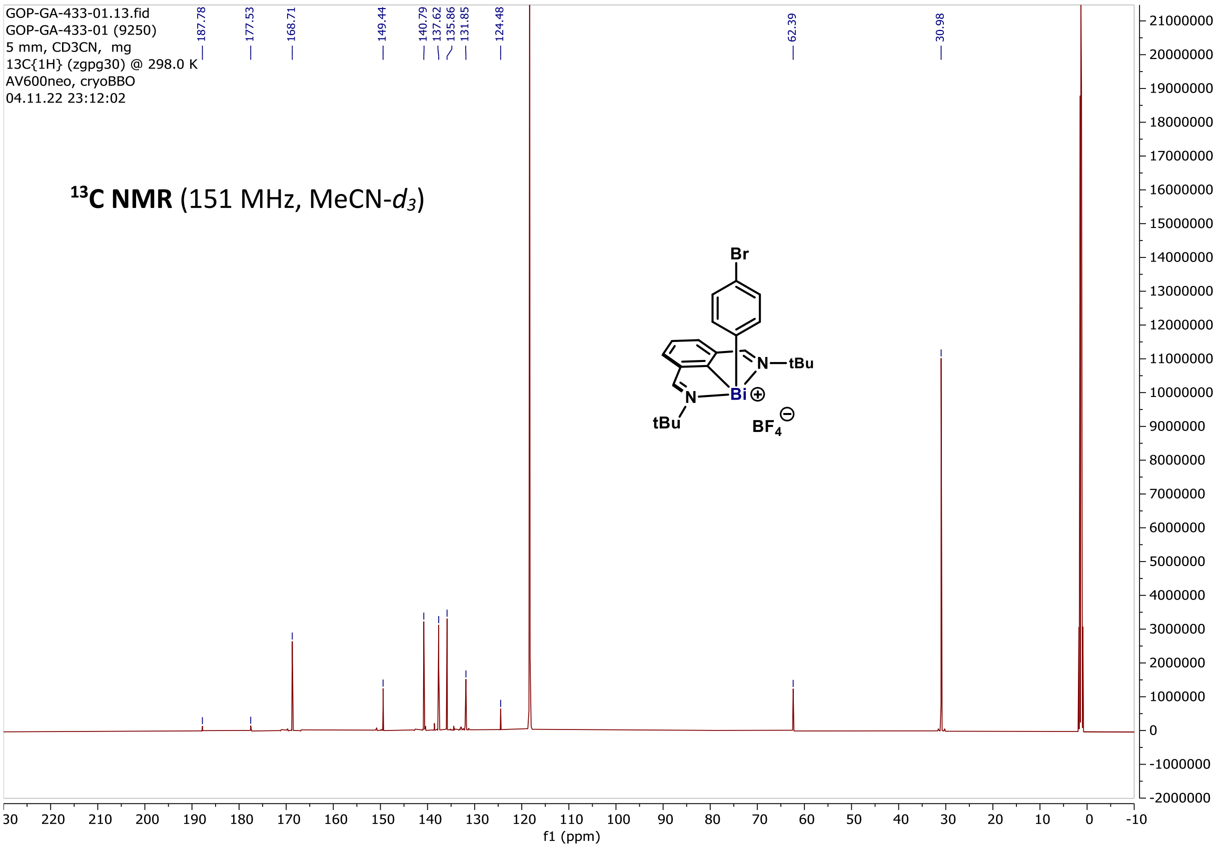

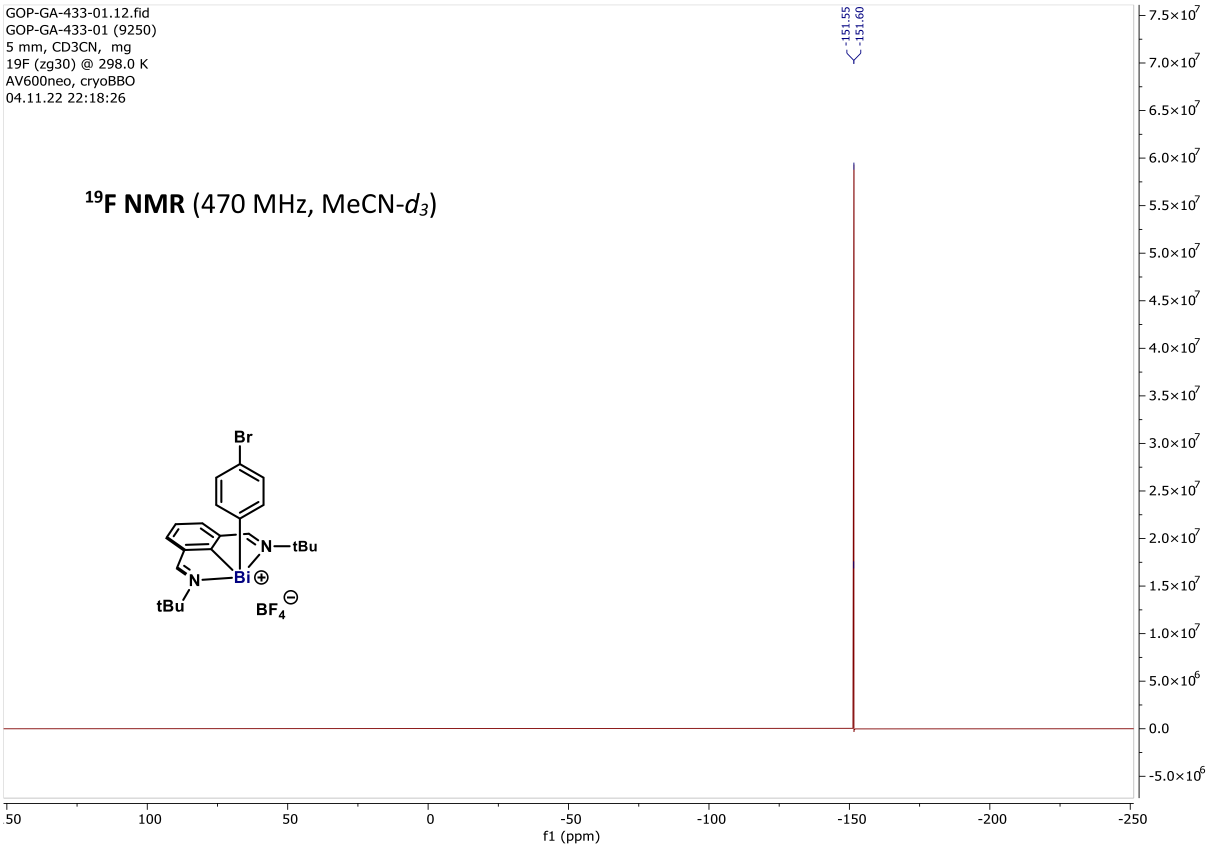
GOP-GA-433-01.12.fid
GOP-GA-433-01 (9250)
5 mm, CD3CN, mg
19F (zg30) @ 298.0 K
AV600neo, cryoBBO
04.11.22 22:18:26
-151.55
-151.60
19F NMR (470 MHz, MeCN-d3)
Br
N—tBu
N
Bi ⊕
tBu
BF4 ⊖
f1 (ppm)

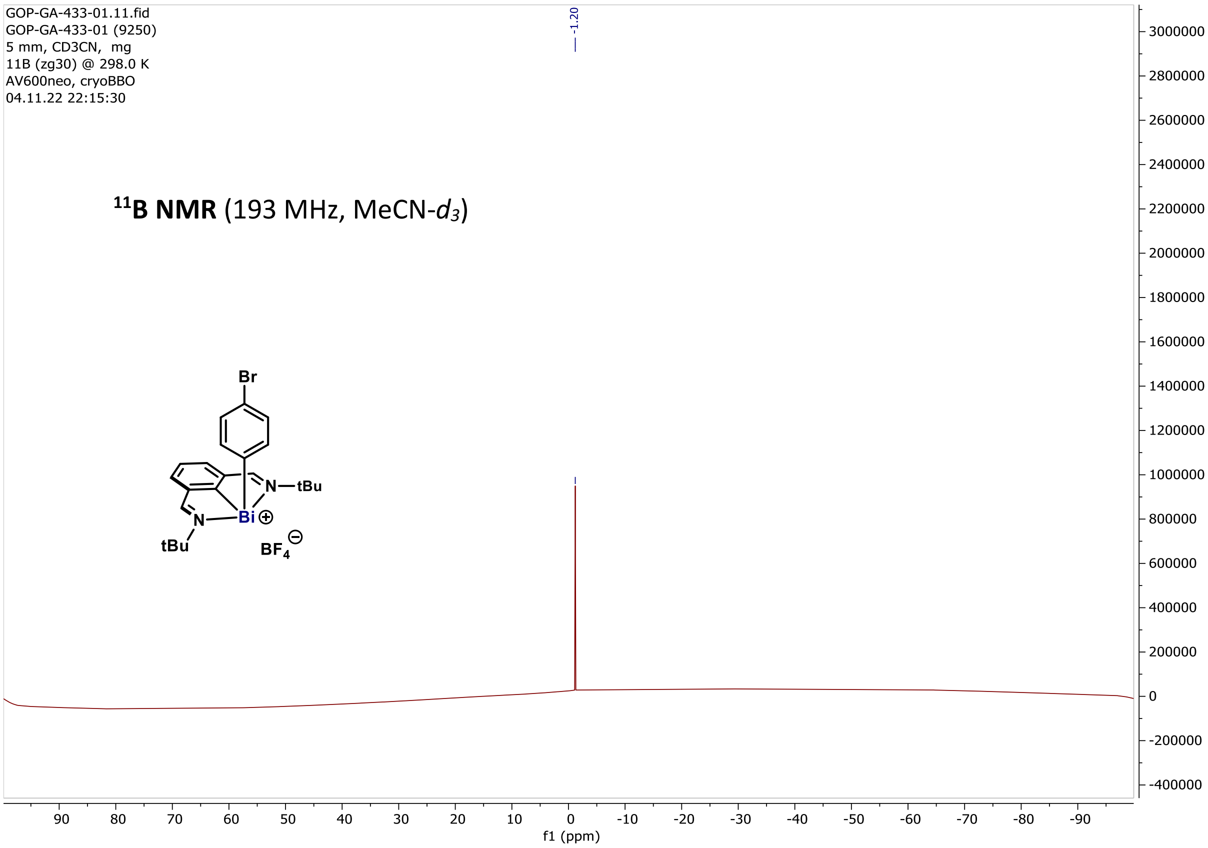
GOP-GA-433-01.11.fid
GOP-GA-433-01 (9250)
5 mm, CD3CN, mg
11B (zg30) @ 298.0 K
AV600neo, cryoBBO
04.11.22 22:15:30
-1.20
11B NMR (193 MHz, MeCN-d3)
Br
N—tBu
N
Bi ⊕
tBu
BF4 ⊖
f1 (ppm)

[(2,6-(*t*BuNCH)$_2$C$_6$H$_3$)Bi(3-quinolinyl)(tetrafluoroborate)] (3g)

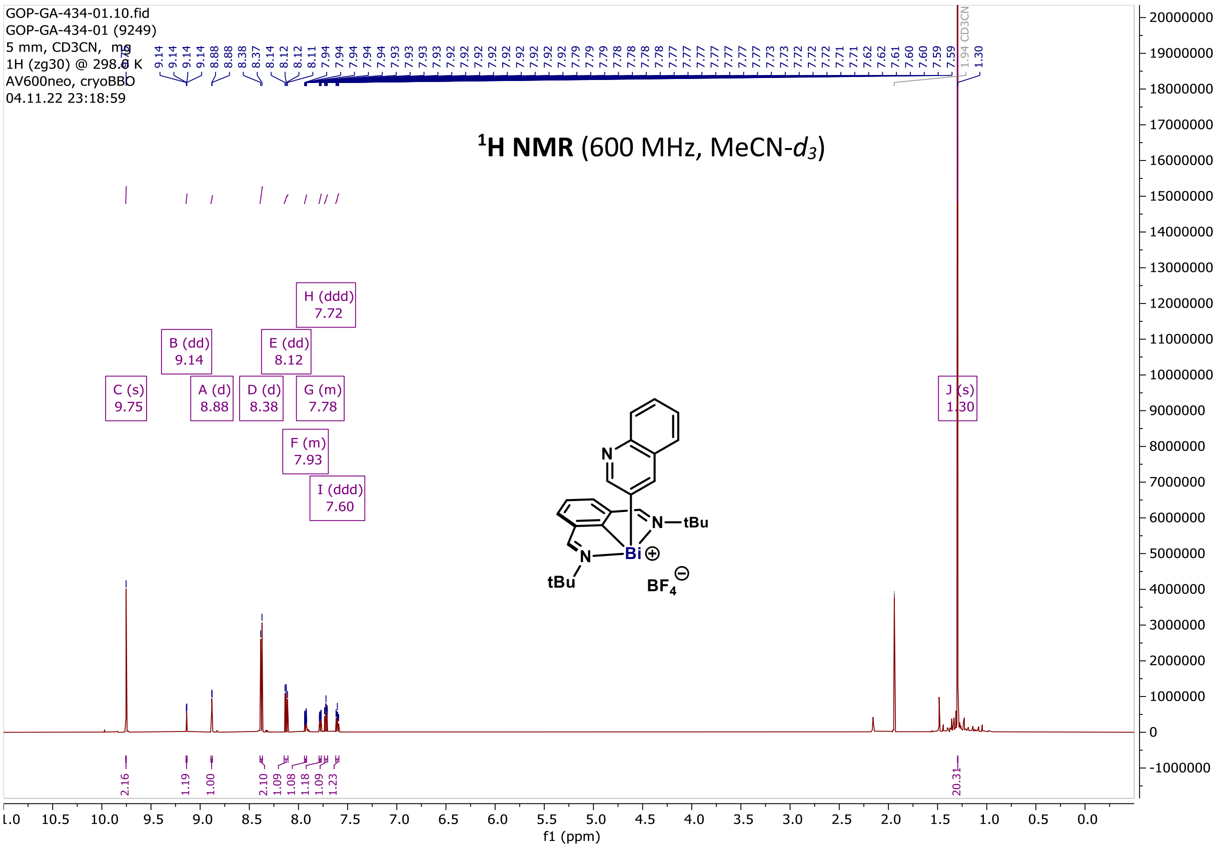

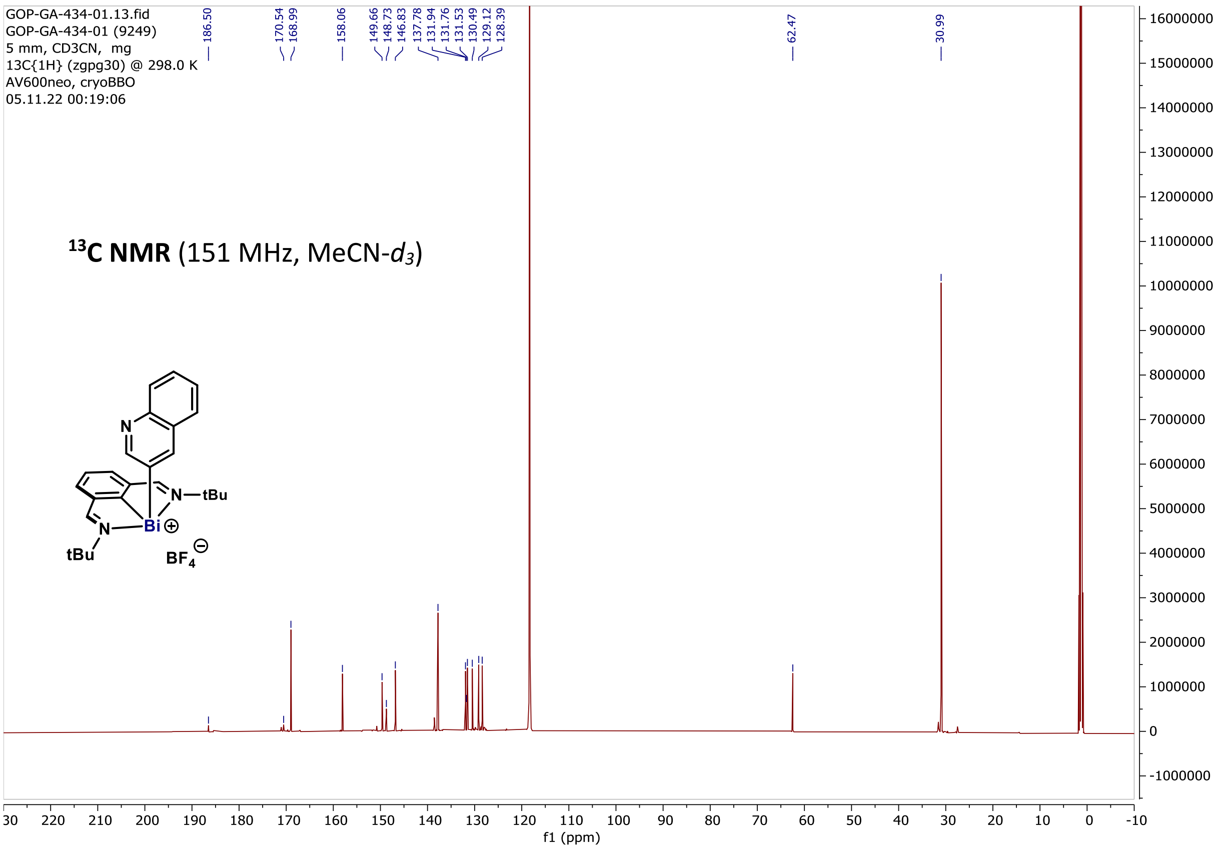

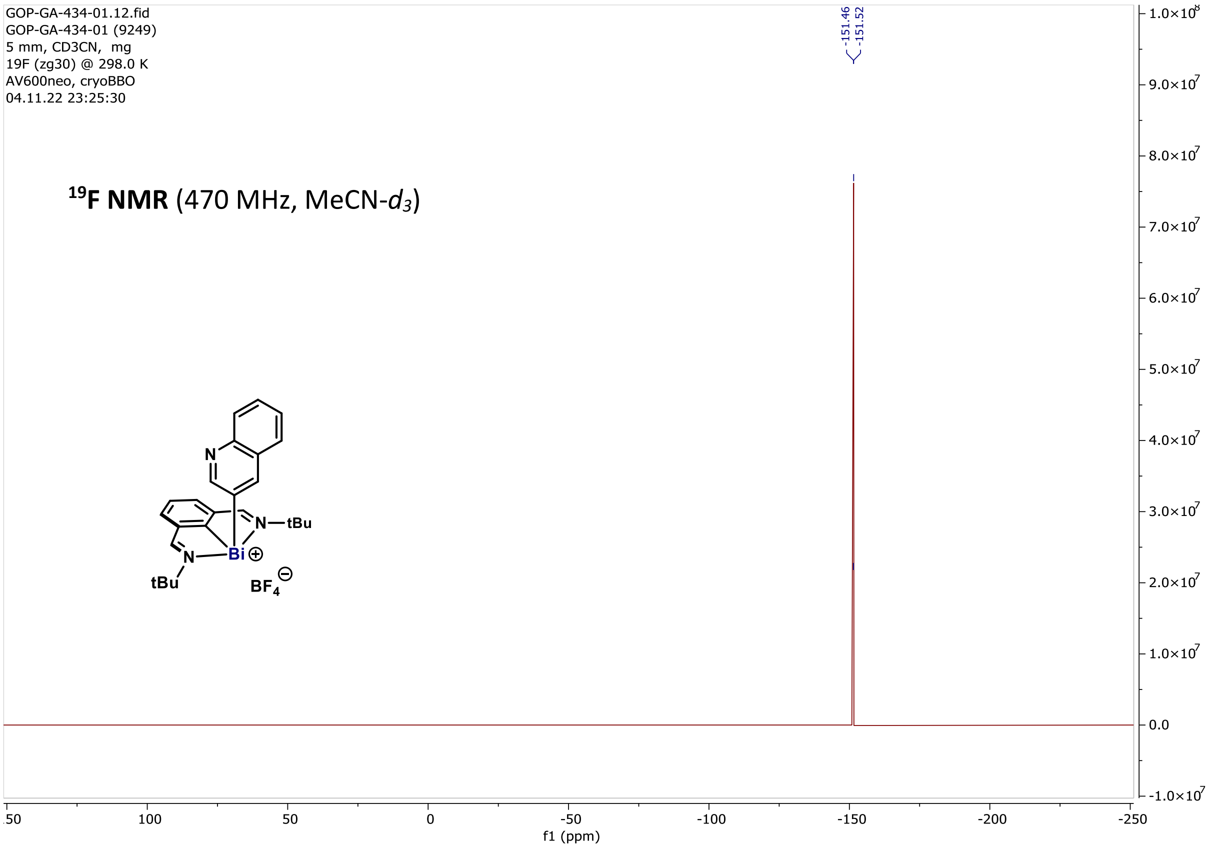
GOP-GA-434-01.12.fid
GOP-GA-434-01 (9249)
5 mm, CD3CN, mg
19F (zg30) @ 298.0 K
AV600neo, cryoBBO
04.11.22 23:25:30
-151.46
-151.52
^{19}F NMR (470 MHz, MeCN-d_3)
N
N—tBu
N
Bi ⊕
tBu
$BF_4^{\ominus}$
f1 (ppm)

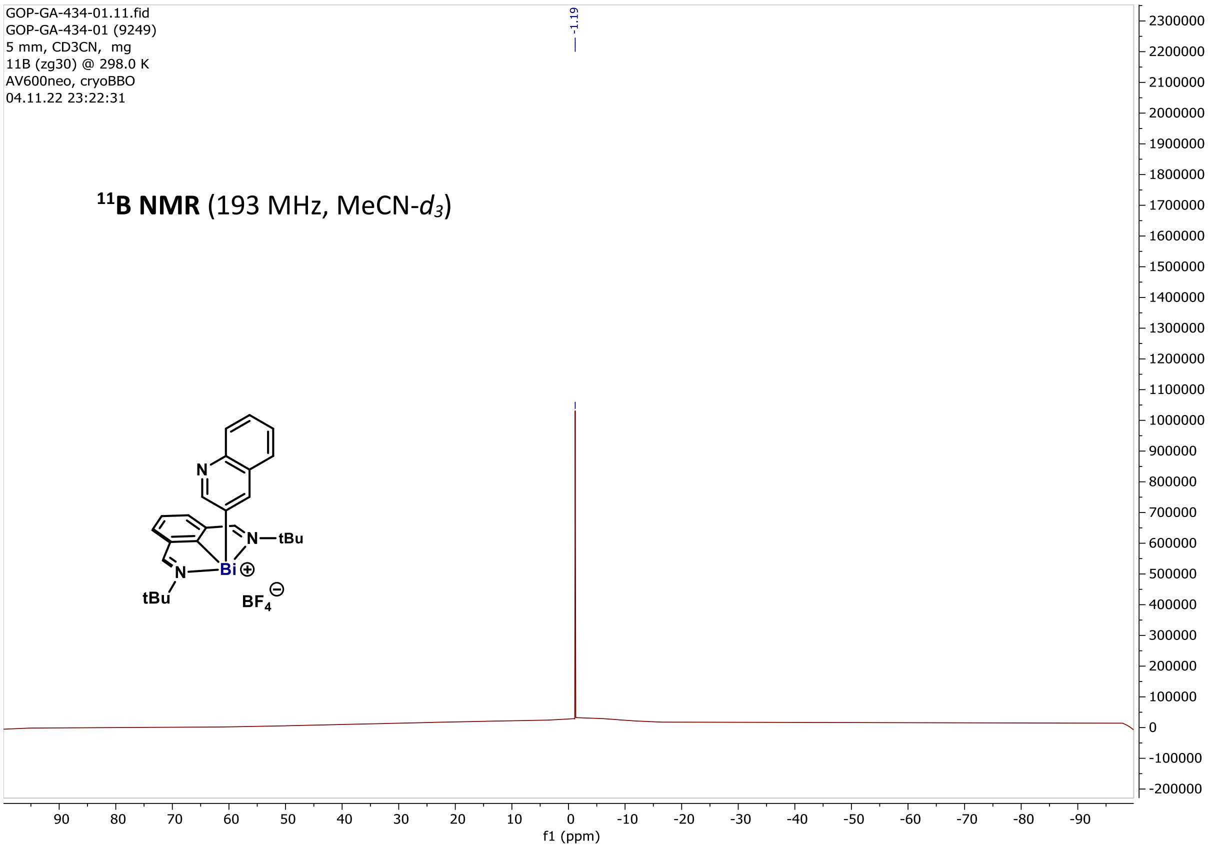
GOP-GA-434-01.11.fid
GOP-GA-434-01 (9249)
5 mm, CD3CN, mg
11B (zg30) @ 298.0 K
AV600neo, cryoBBO
04.11.22 23:22:31
-1.19
^{11}B NMR (193 MHz, MeCN-d_3)
N
N—tBu
N
Bi ⊕
tBu
$BF_4^{\ominus}$
f1 (ppm)

[(2,6-(*t*BuNCH)$_2$C$_6$H$_3$)Bi(8-quinolinyl)(tetrafluoroborate)] (**3h**) (together with unknown side-products)

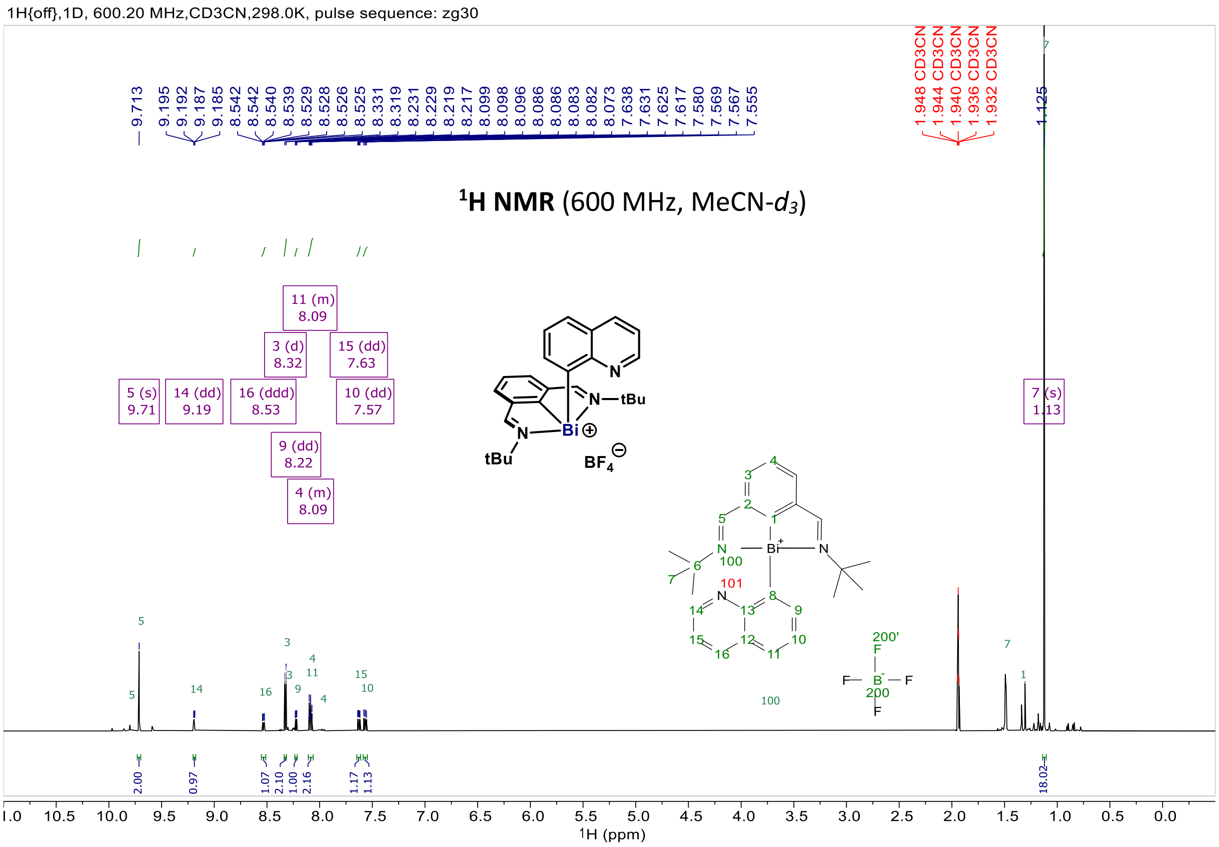

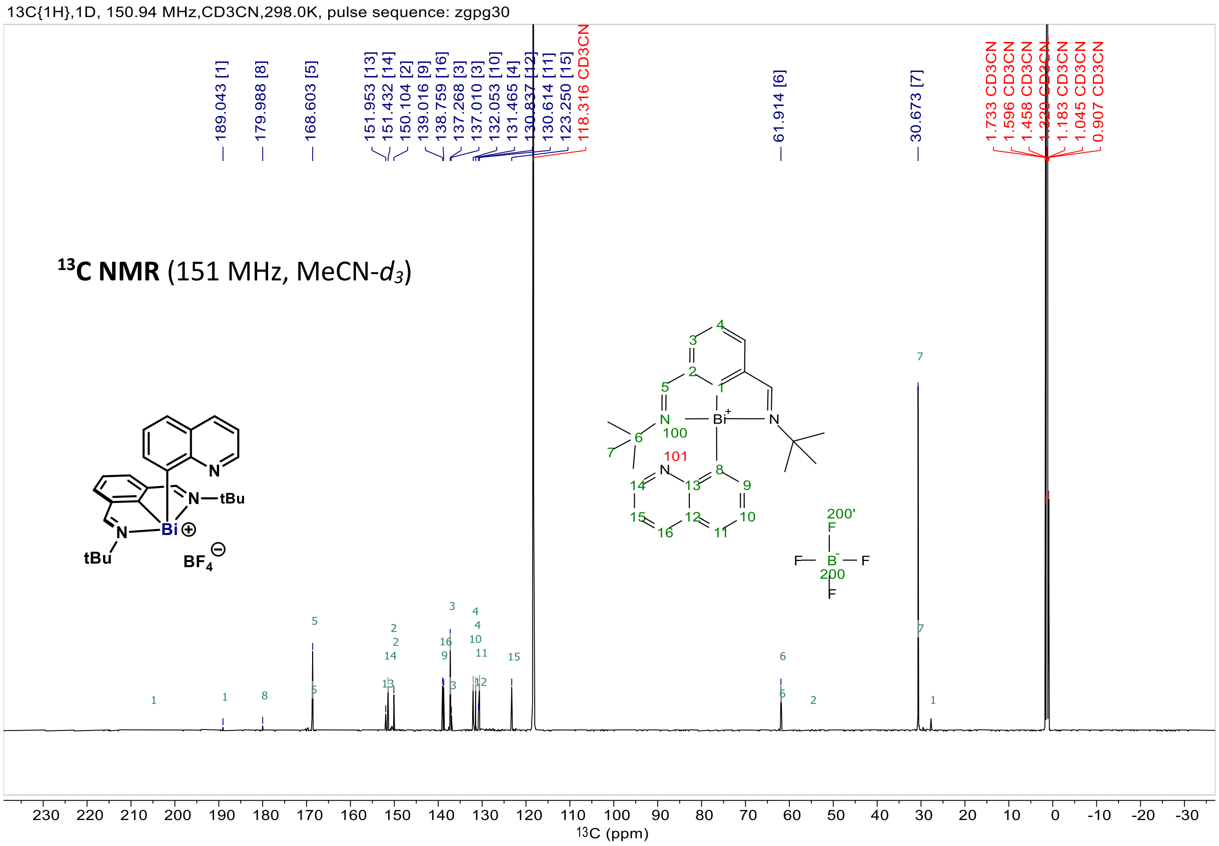

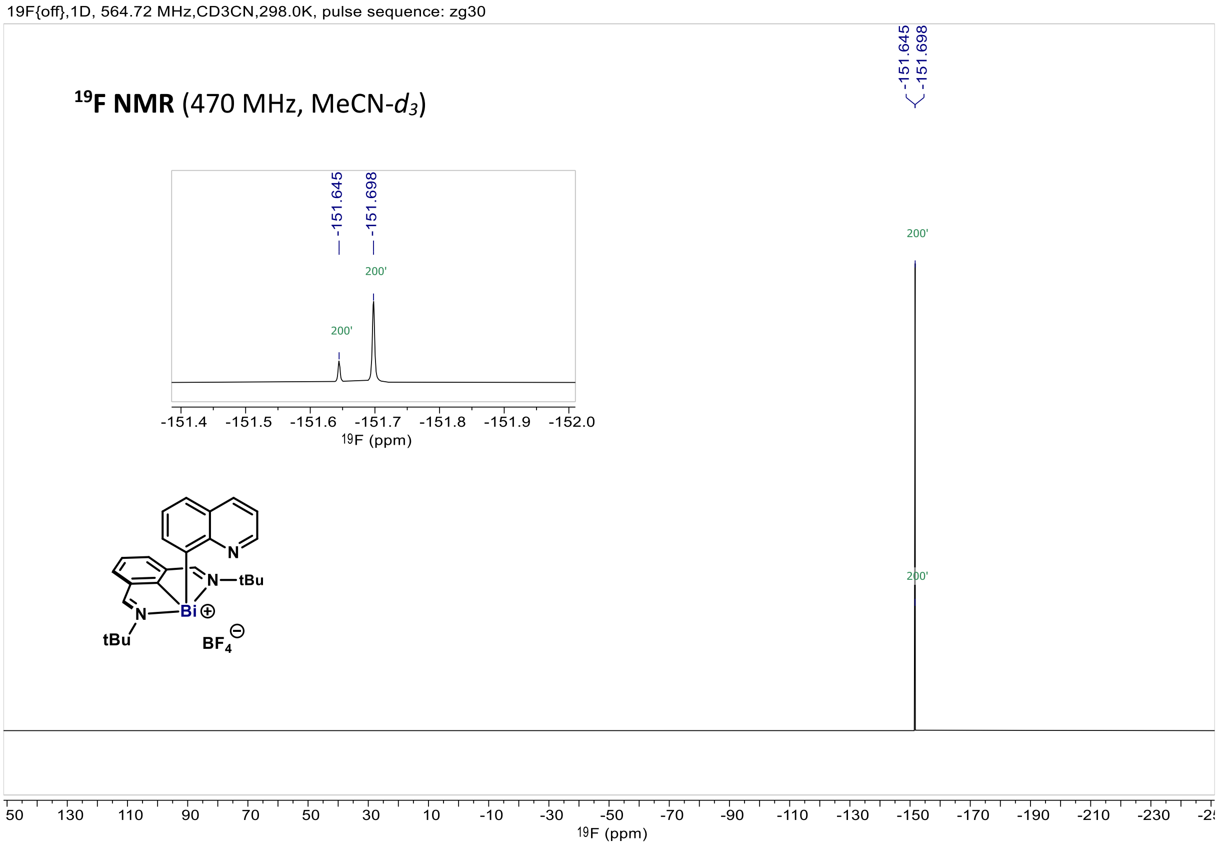
19F{off},1D, 564.72 MHz,CD3CN,298.0K, pulse sequence: zg30
^{19}F NMR (470 MHz, MeCN-d_3)
-151.645
-151.698
200'
-151.4 -151.5 -151.6 -151.7 -151.8 -151.9 -152.0
19F (ppm)
N
N—tBu
Bi
tBu
BF_4
50 130 110 90 70 50 30 10 -10 -30 -50 -70 -90 -110 -130 -150 -170 -190 -210 -230
19F (ppm)

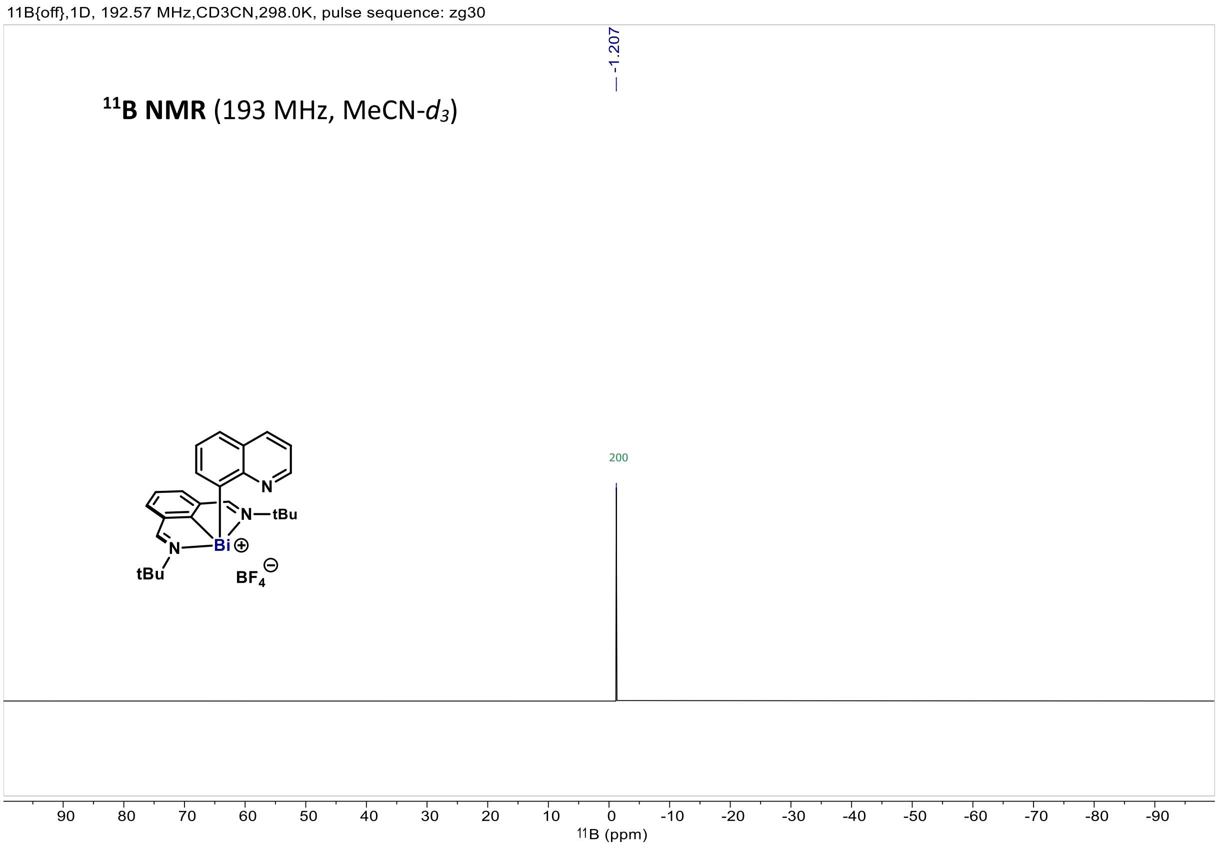
11B{off},1D, 192.57 MHz,CD3CN,298.0K, pulse sequence: zg30
^{11}B NMR (193 MHz, MeCN-d_3)
-1.207
200
N
N—tBu
Bi
tBu
BF_4
90 80 70 60 50 40 30 20 10 0 -10 -20 -30 -40 -50 -60 -70 -80 -90
11B (ppm)

[(2,6-(*t*BuNCH)$_2$C$_6$H$_3$)Bi(3-(2-methoxycarbonyl)thiophenyl)(tetrafluoroborate)] (3i) (together with 10% of what looks like the (5-thiophenyl)-bismuth(III) isomer)

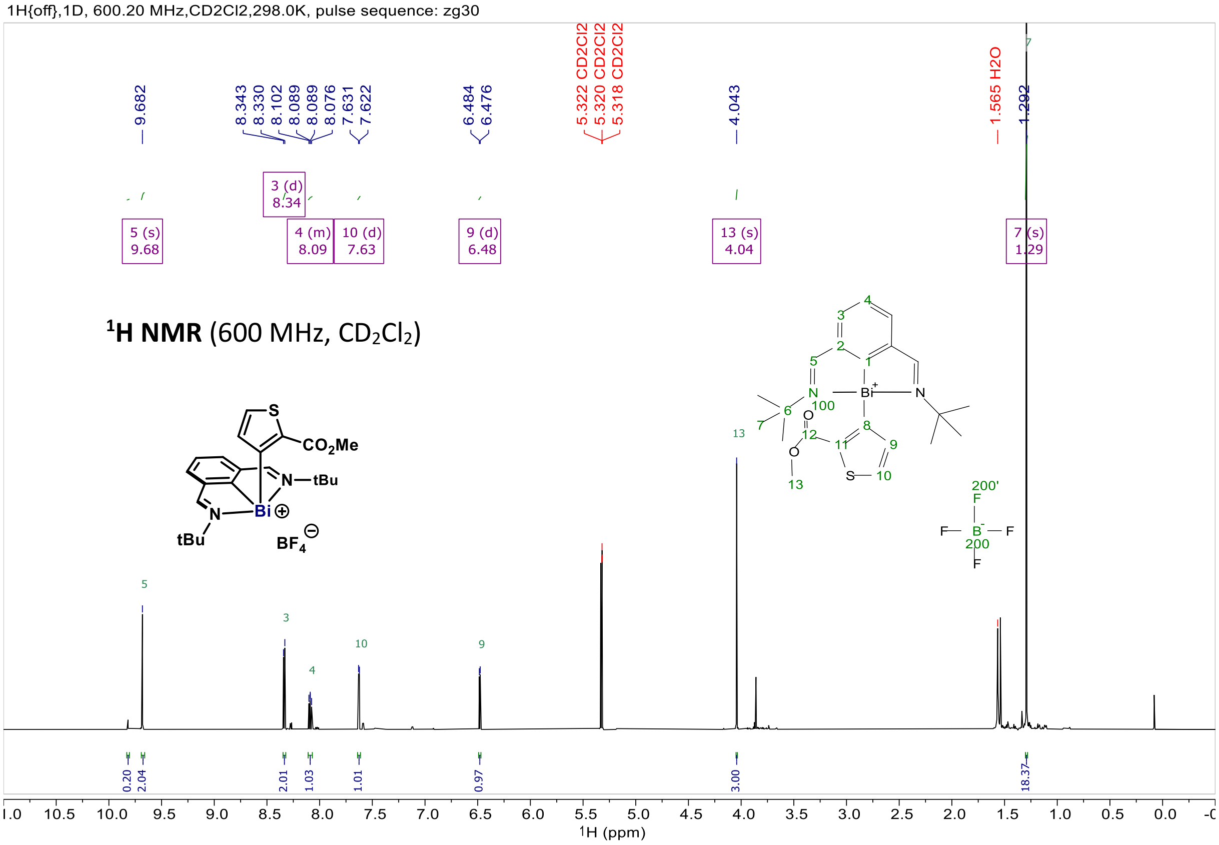

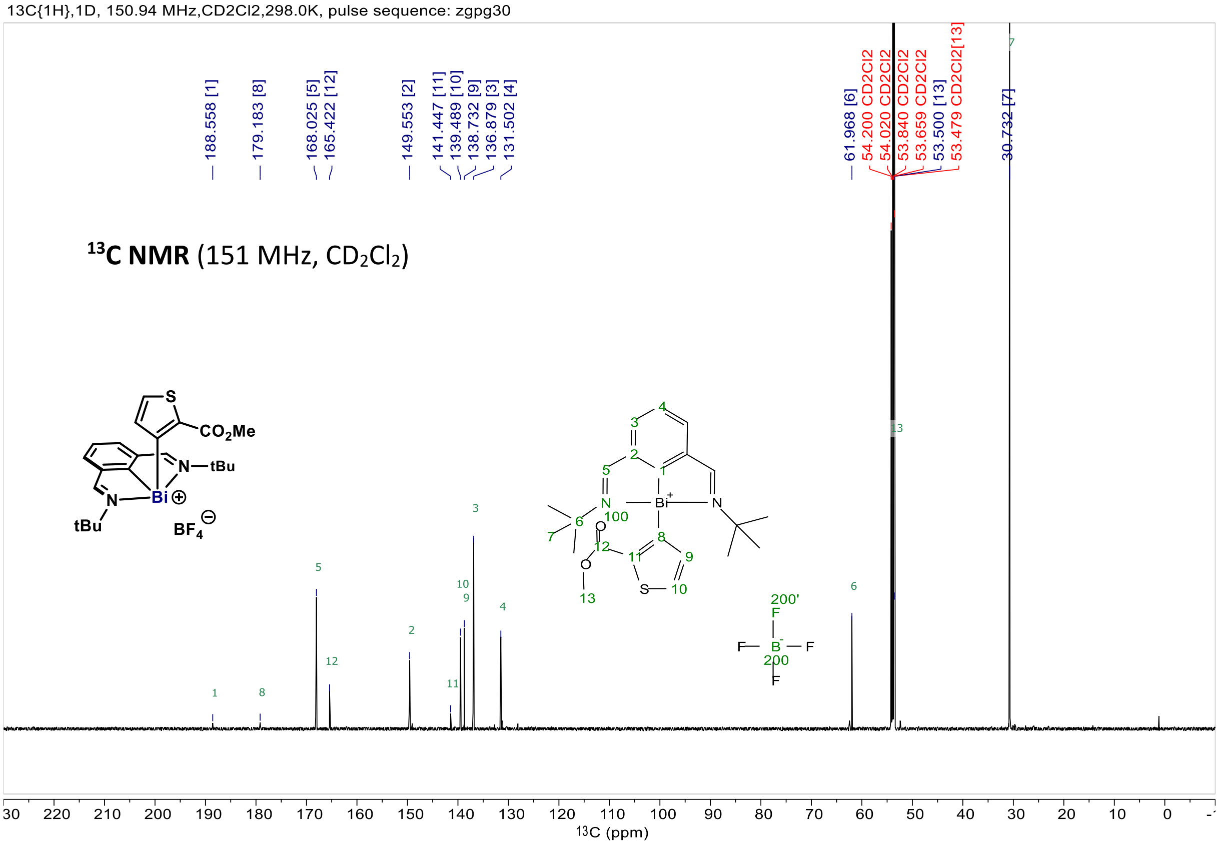

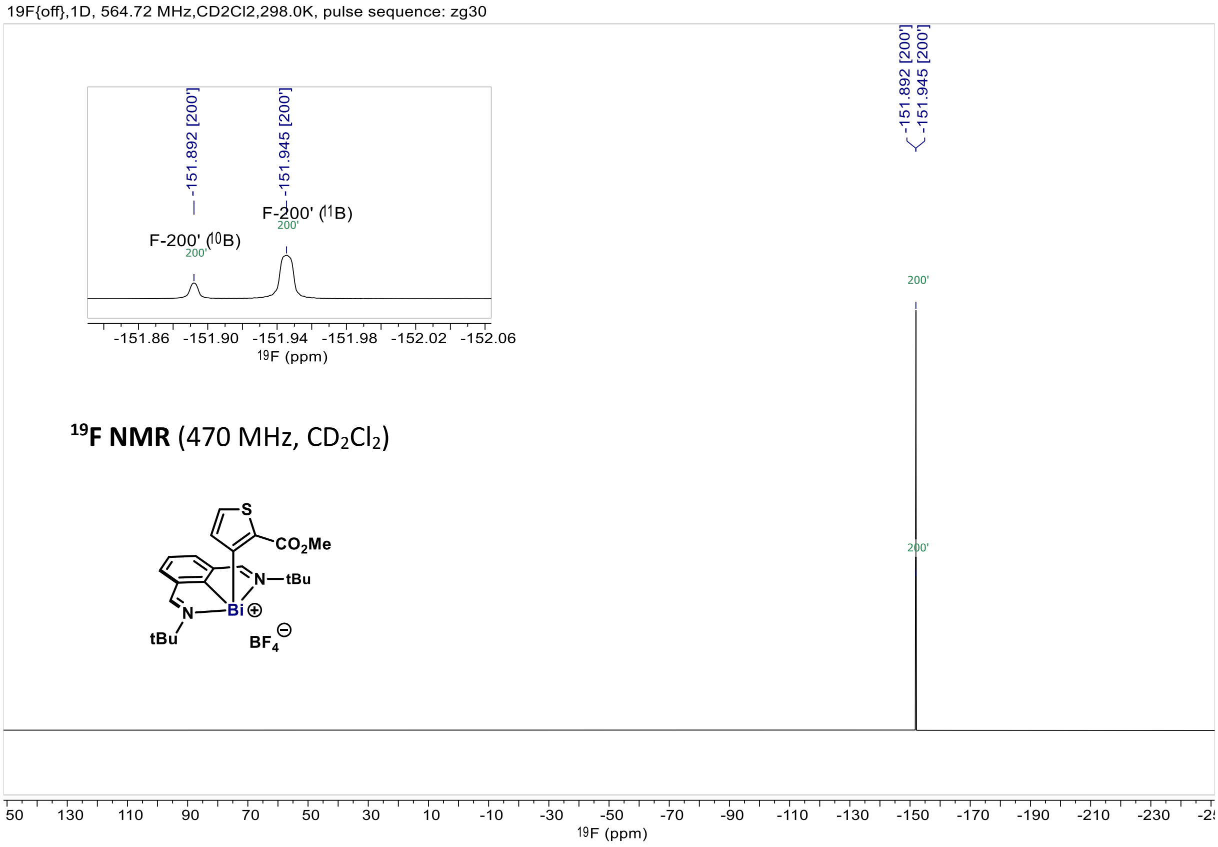
19F{off},1D, 564.72 MHz,CD2Cl2,298.0K, pulse sequence: zg30
-151.892 [200']
-151.945 [200']
F-200' (10B)
F-200' (11B)
-151.86 -151.90 -151.94 -151.98 -152.02 -152.06
19F (ppm)
19F NMR (470 MHz, CD2Cl2)
S
CO2Me
N—tBu
N—Bi ⊕
tBu
BF4 ⊖
200'
19F (ppm)

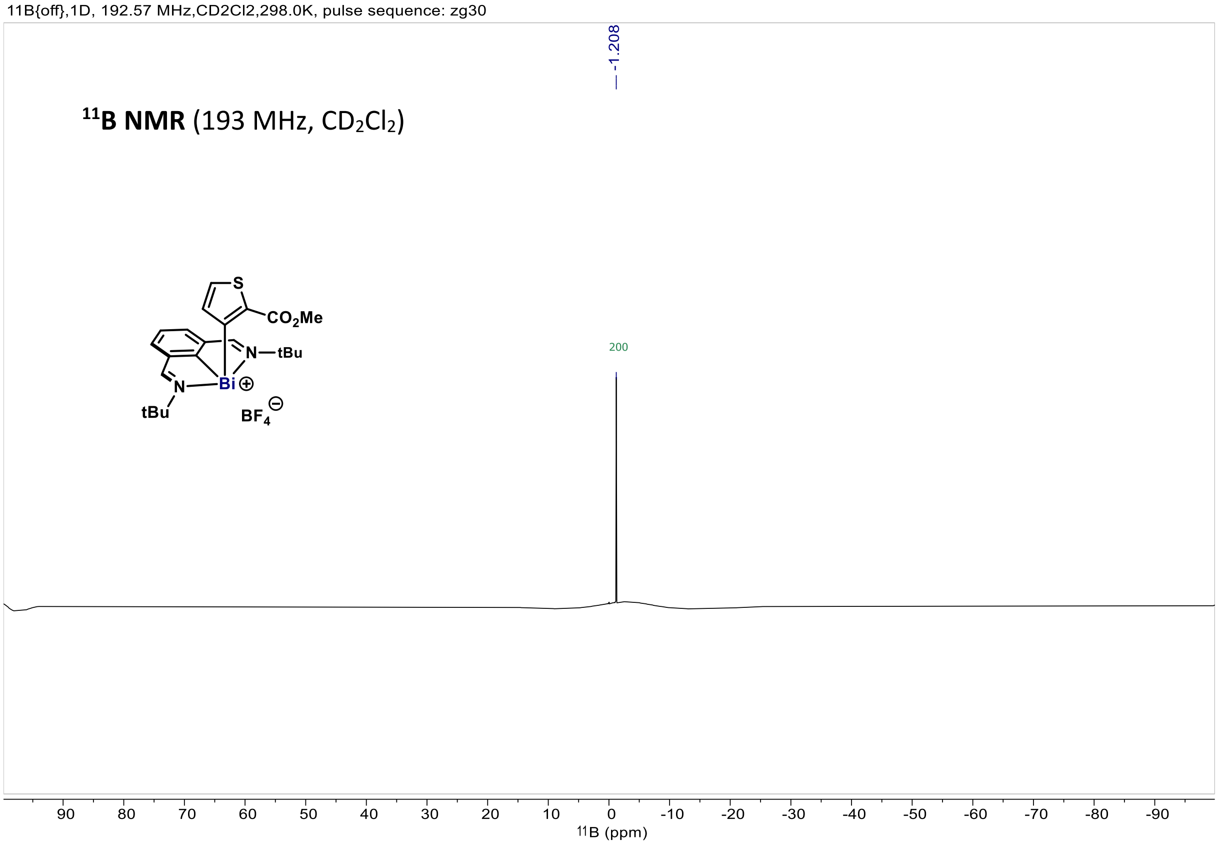
11B{off},1D, 192.57 MHz,CD2Cl2,298.0K, pulse sequence: zg30
-1.208
11B NMR (193 MHz, CD2Cl2)
S
CO2Me
N—tBu
N—Bi ⊕
tBu
BF4 ⊖
200
90 80 70 60 50 40 30 20 10 0 -10 -20 -30 -40 -50 -60 -70 -80 -90
11B (ppm)

[(2,6-(*t*BuNCH)$_2$C$_6$H$_3$)Bi(4-methoxyphenyl)(tetrafluoroborate)] (7a)

1H{off},1D, 600.20 MHz,CD3CN,298.0K, pulse sequence: zg30

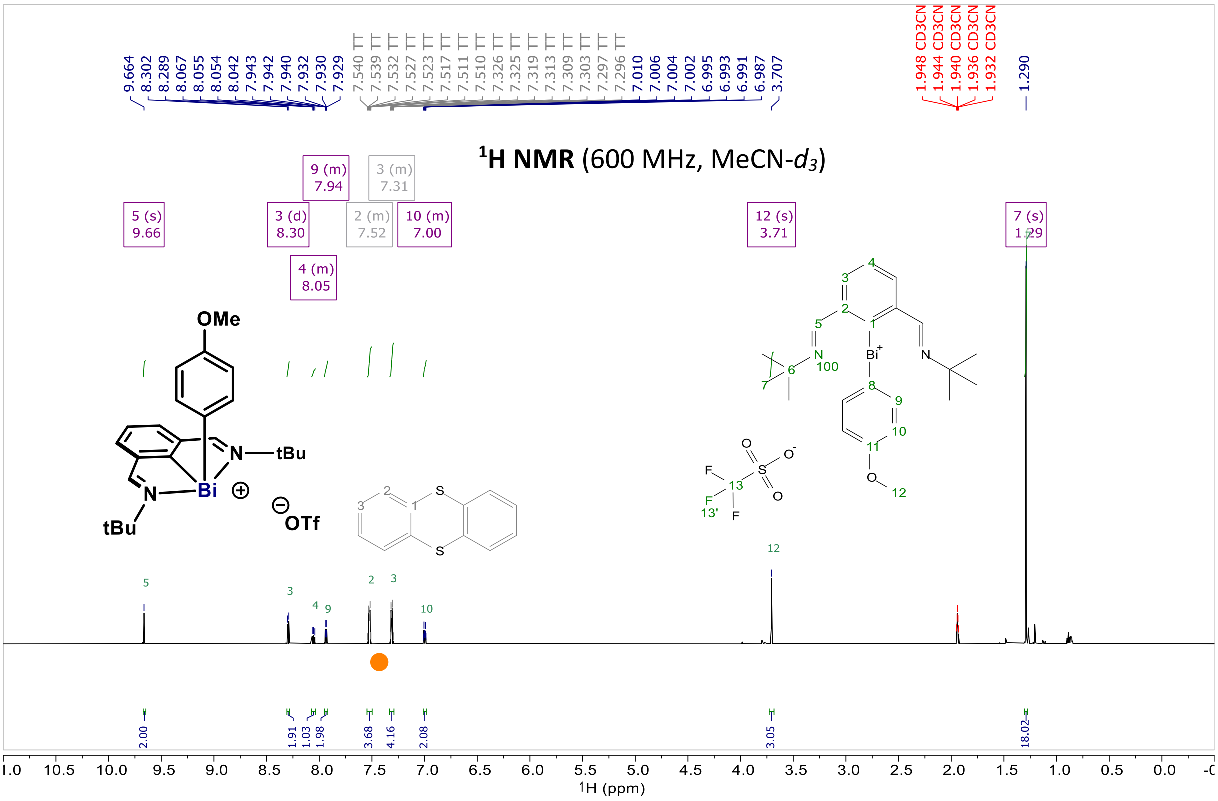

13C{1H},1D, 150.94 MHz,CD3CN,298.0K, pulse sequence: zgpg30

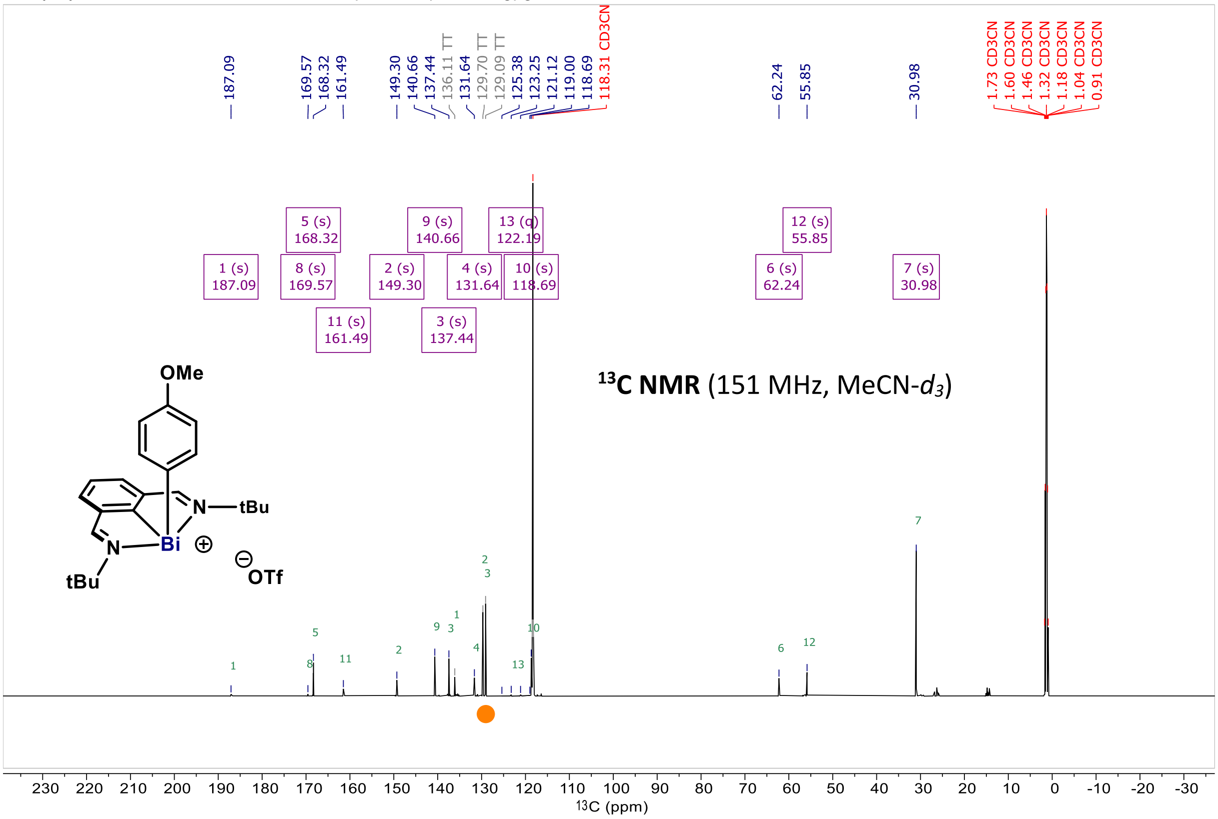

● Released thianthrene

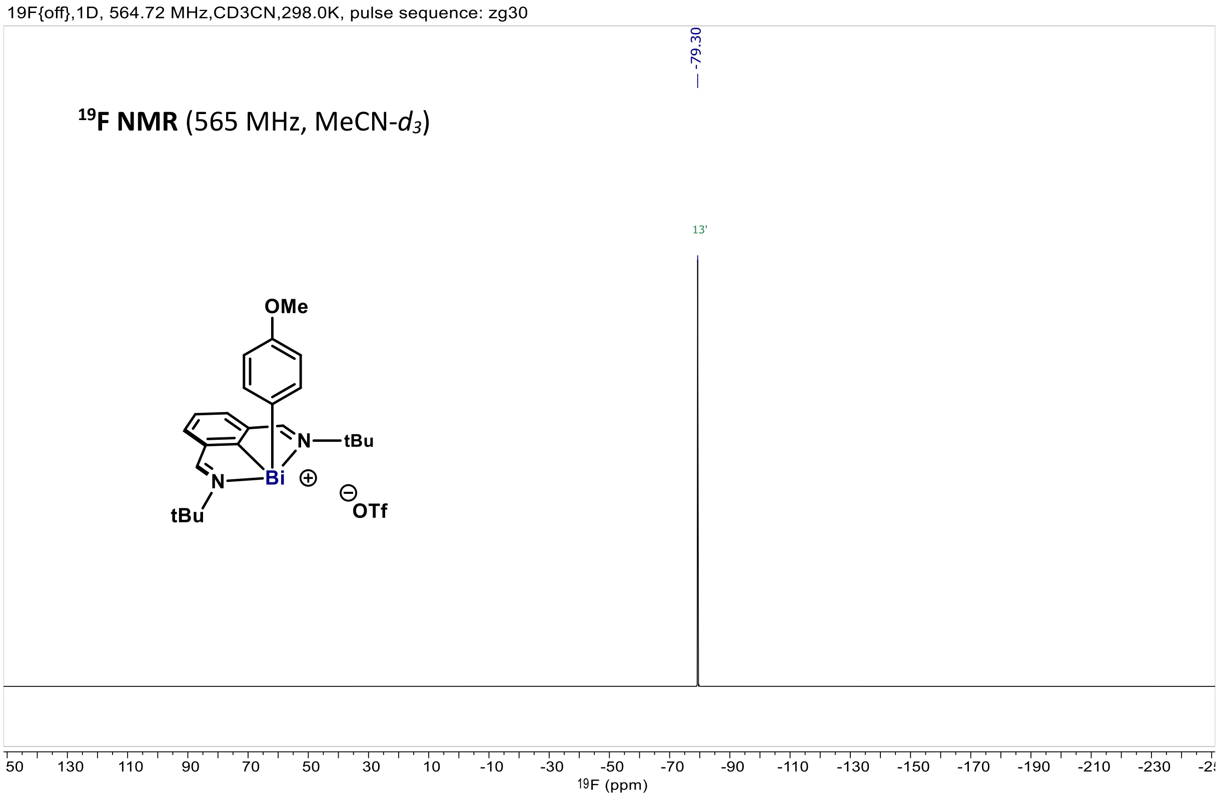
19F{off},1D, 564.72 MHz,CD3CN,298.0K, pulse sequence: zg30
-79.30
19F NMR (565 MHz, MeCN-d3)
13'
OMe
N
tBu
Bi
OTf
19F (ppm)

[(2,6-(*t*BuNCH)$_2$C$_6$H$_3$)Bi(4-(4-(2-(pyridin-2-yloxy)propoxy)phenoxy)phenyl)(tetrafluoroborate)] (7b)

1H{off,off},1D, 600.22 MHz,CD3CN,298.0K, pulse sequence: zg30

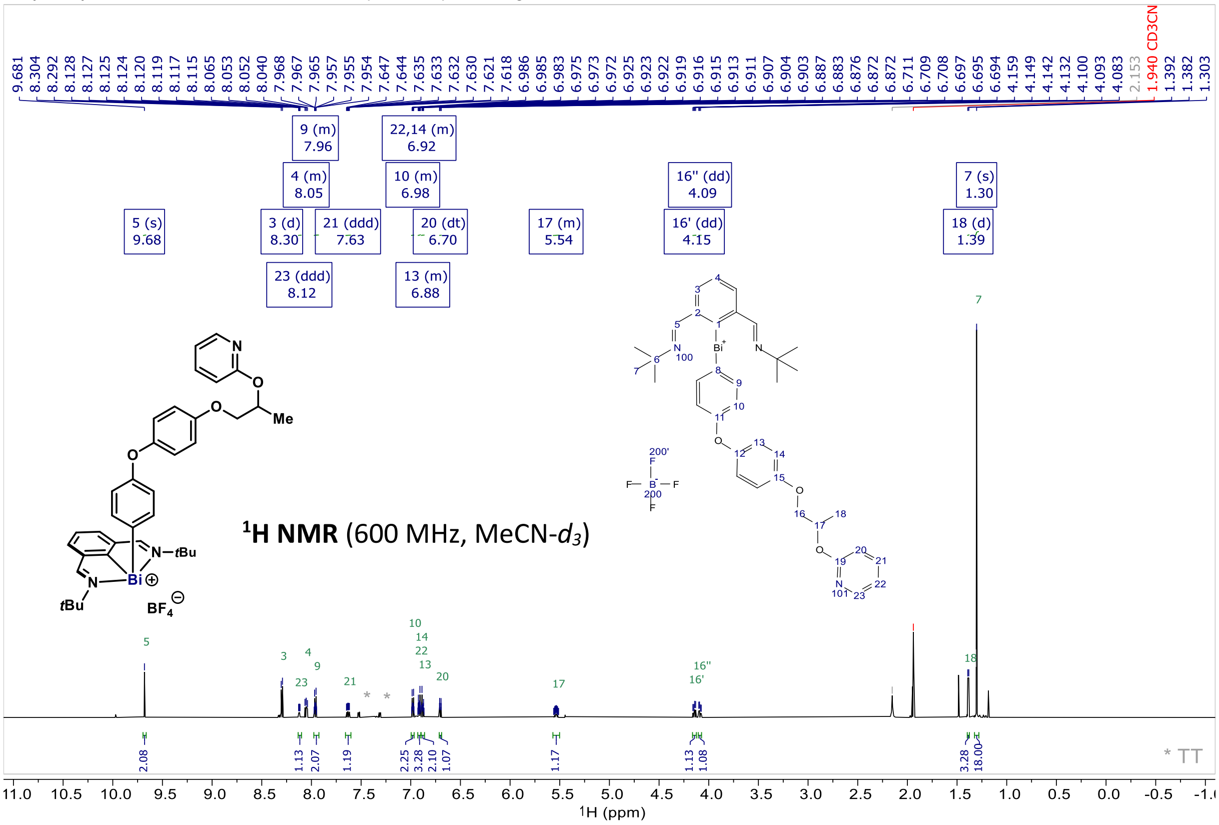

13C{1H,off},1D, 150.94 MHz,CD3CN,298.0K, pulse sequence: zgdc30

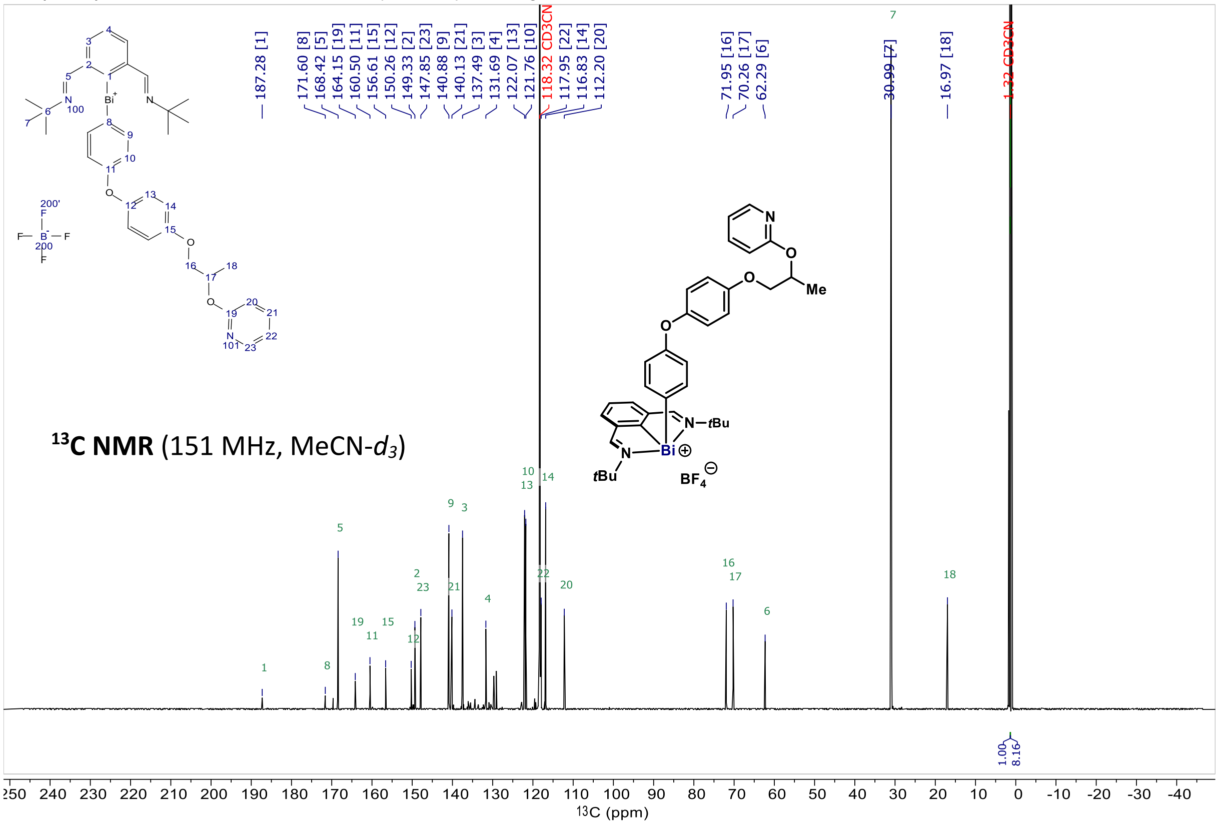

19F{1H,off},1D, 470.31 MHz,CD3CN,298.0K, pulse sequence: zg30

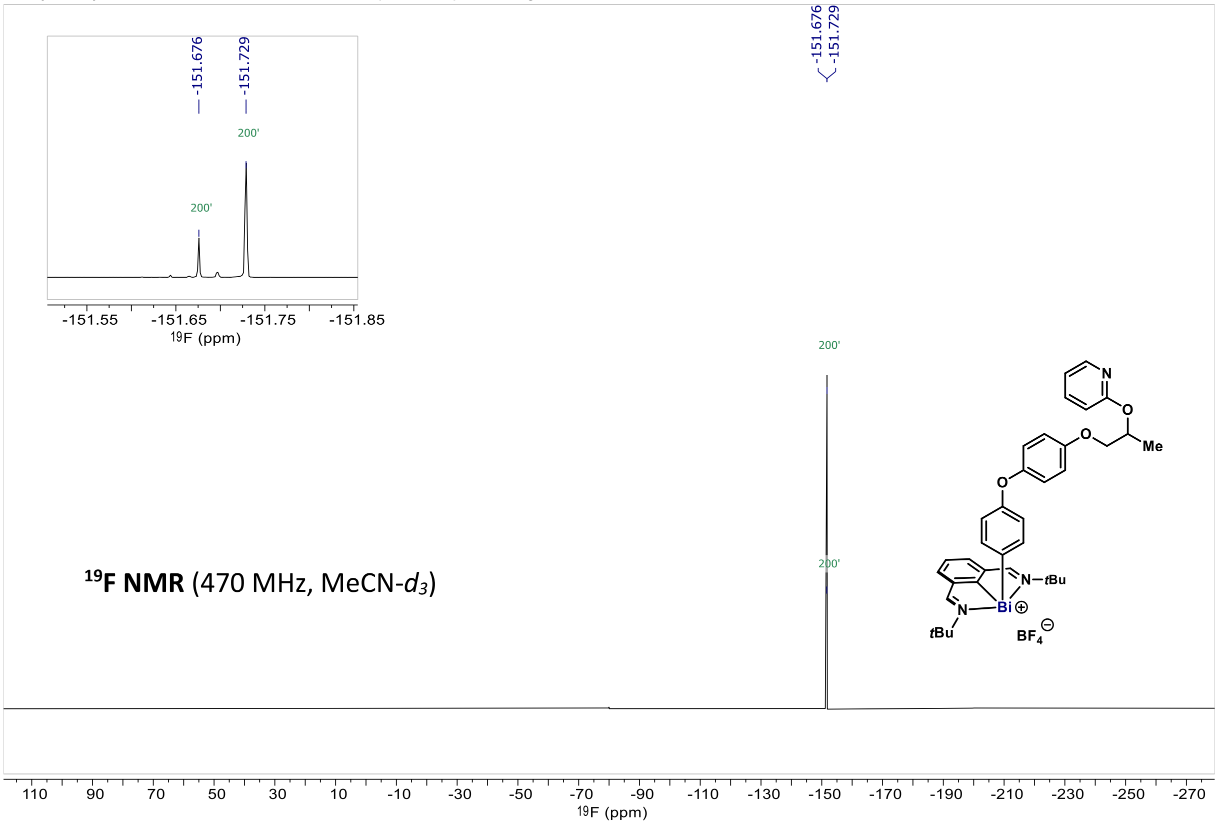

11B{1H,off},1D, 160.38 MHz,CD3CN,298.0K, pulse sequence: zgbs

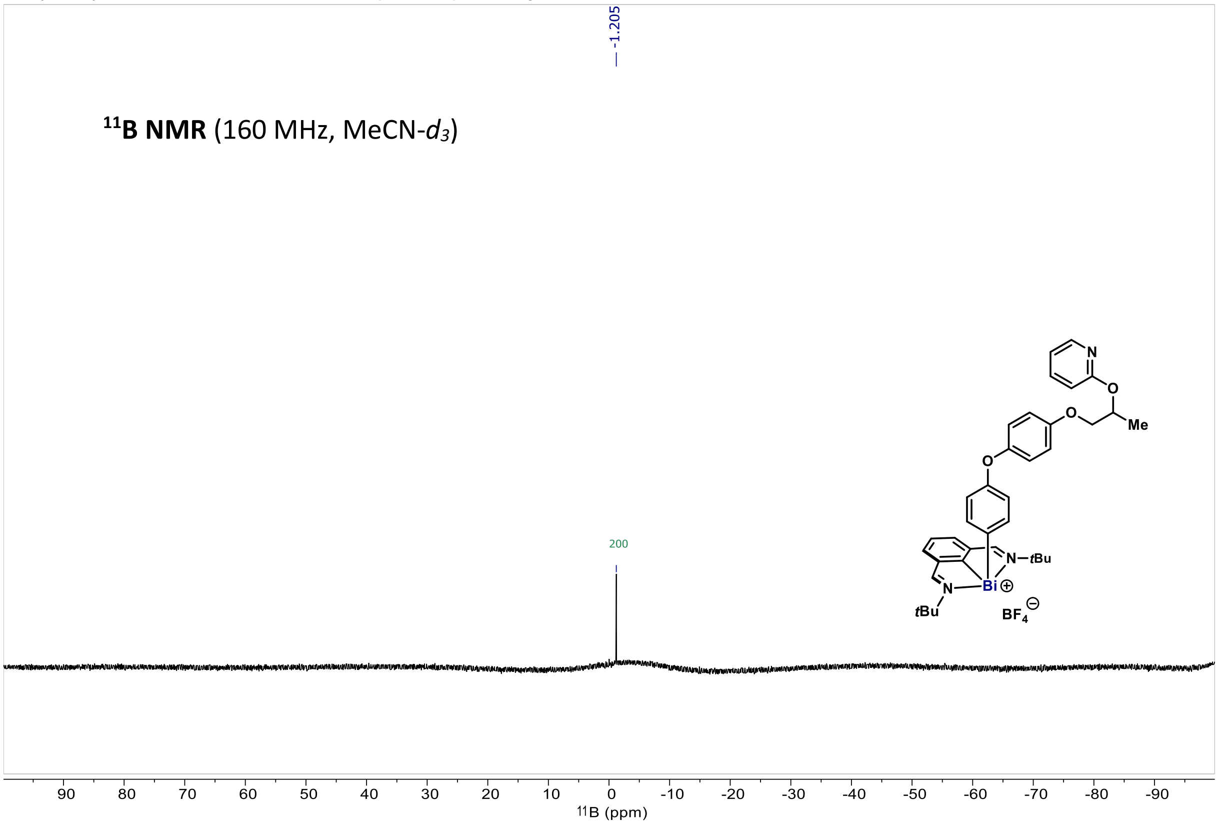

[(2,6-(*t*BuNCH)$_2$C$_6$H$_3$)Bi(4-*N*-tosyl-*N*-methyl-aniline)(tetrafluoroborate)] (7c)

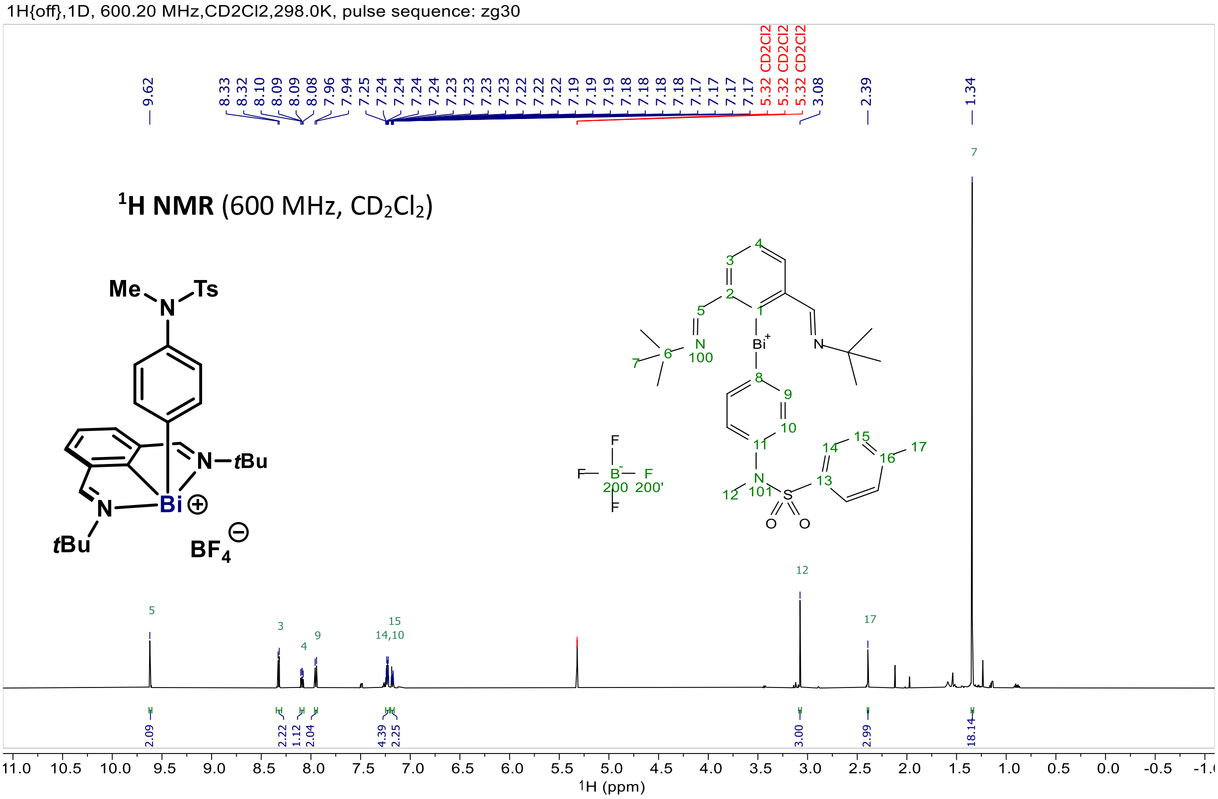

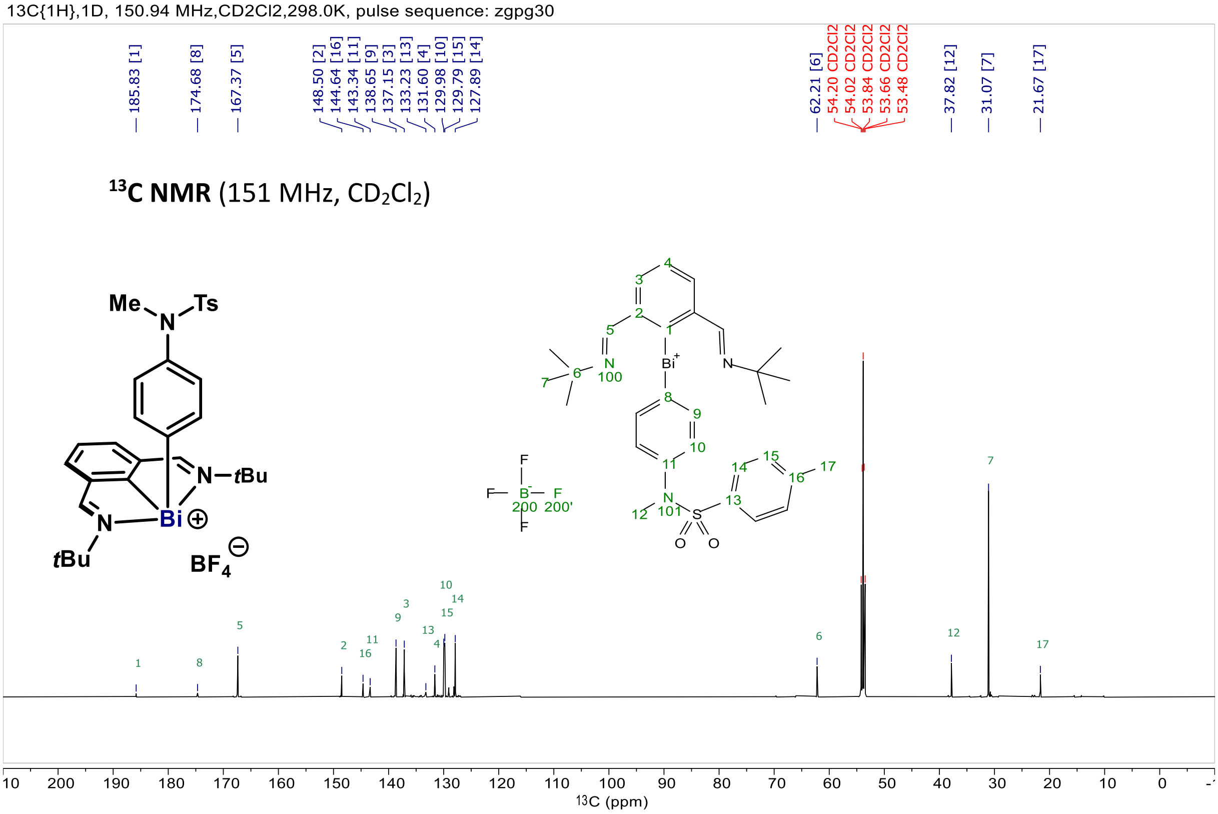

$^1H\{^{13}C\}$ HSQC

1H{13C},HSQC-EDITED, 600.20 MHz,CD2Cl2,298.0K, pulse sequence: hsqcedetgpsisp2.3

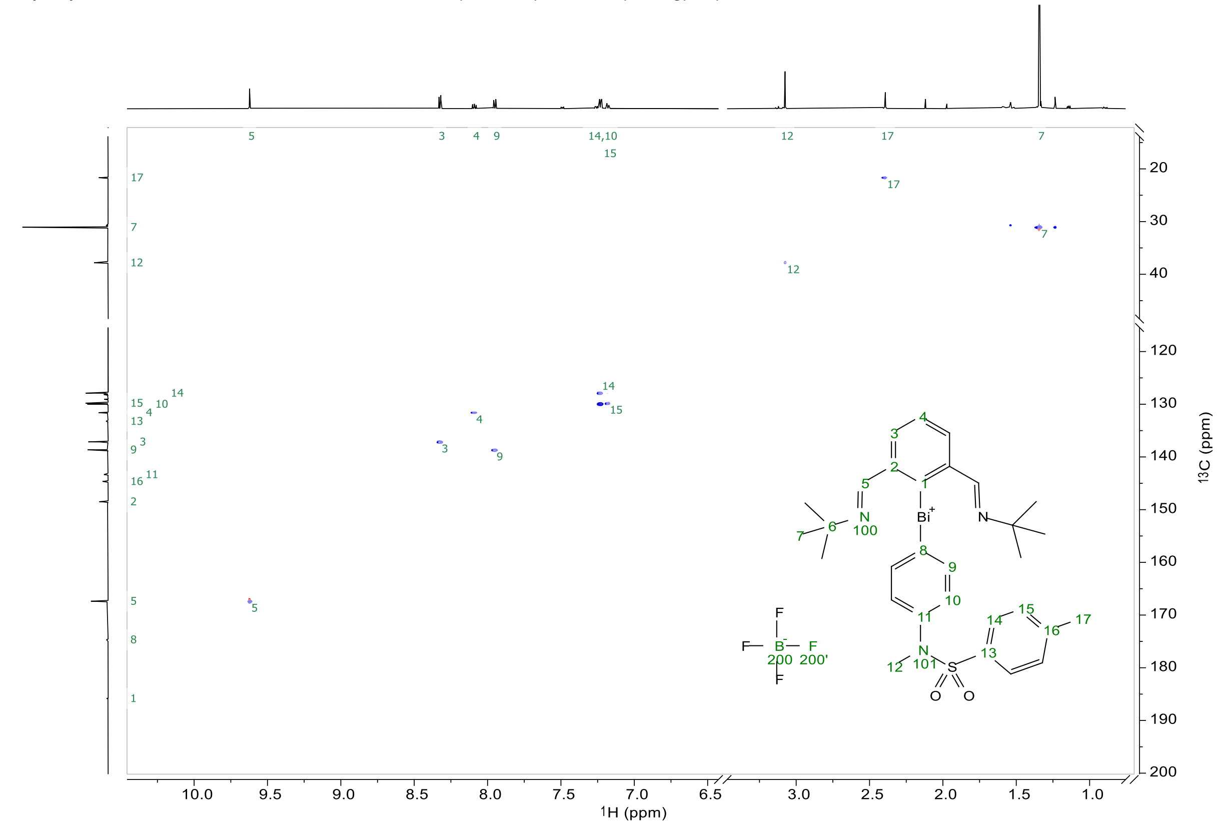

$^1H\{^{13}C\}$ HMBC

1H{13C},HMBC, 600.20 MHz,CD2Cl2,298.0K, pulse sequence: hmbcetgpl3nd

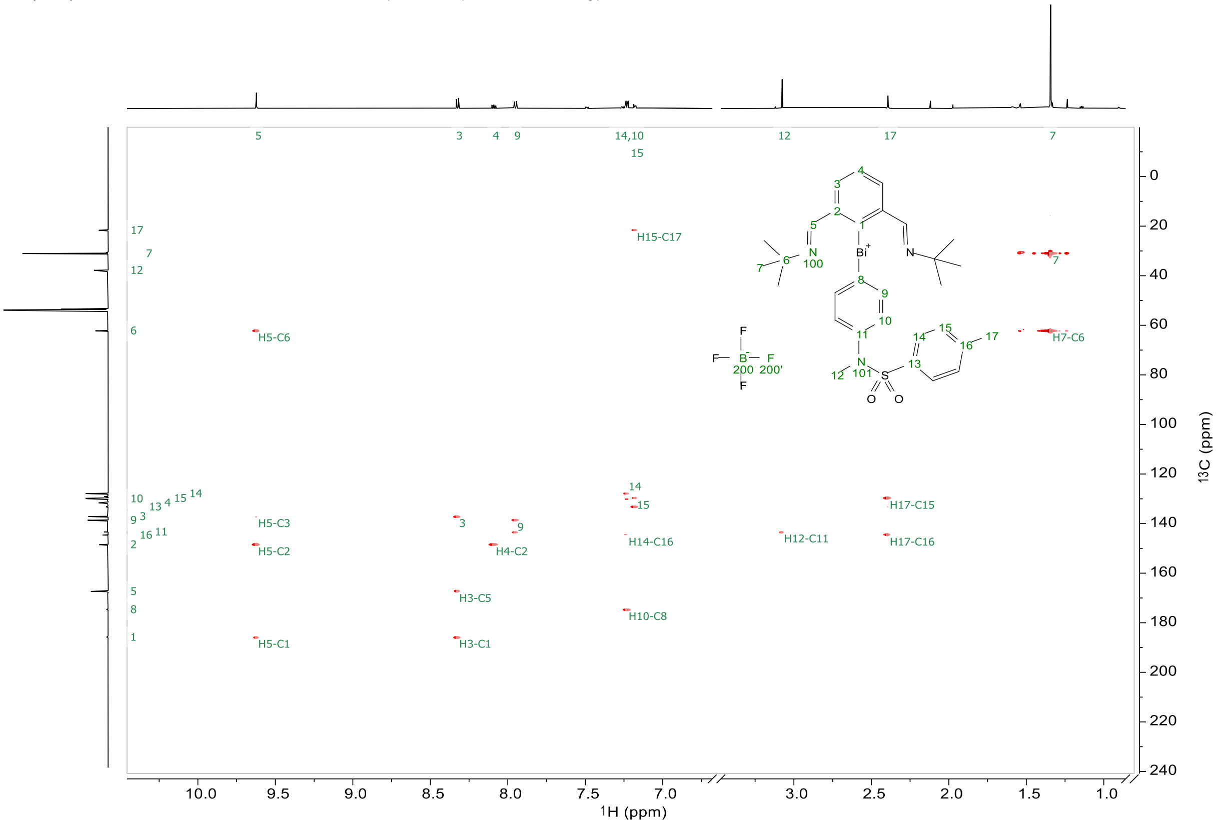

^{1}H COSY

1H{off},COSY, 600.20 MHz,CD2Cl2,298.0K, pulse sequence: cosygpppqf

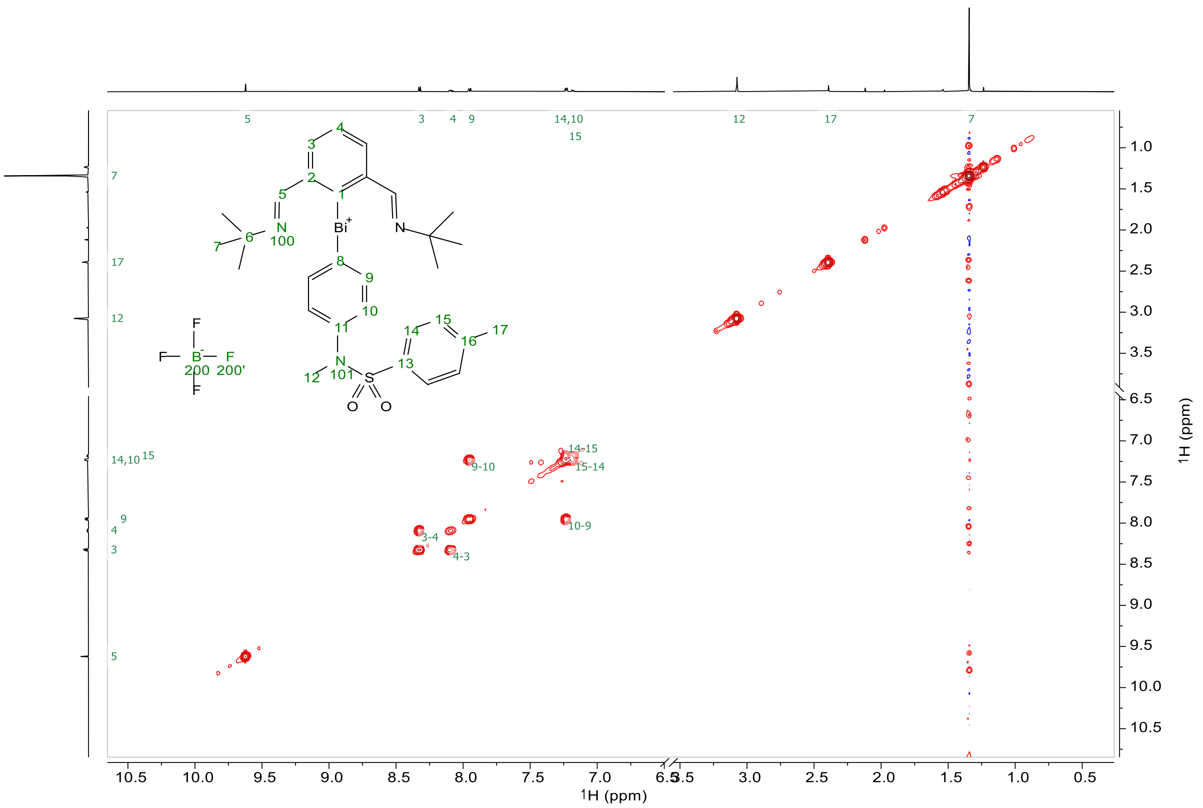

^{1}H NOESY

1H{off},NOESY, 600.20 MHz,CD2Cl2,298.0K, pulse sequence: noesygpphpp

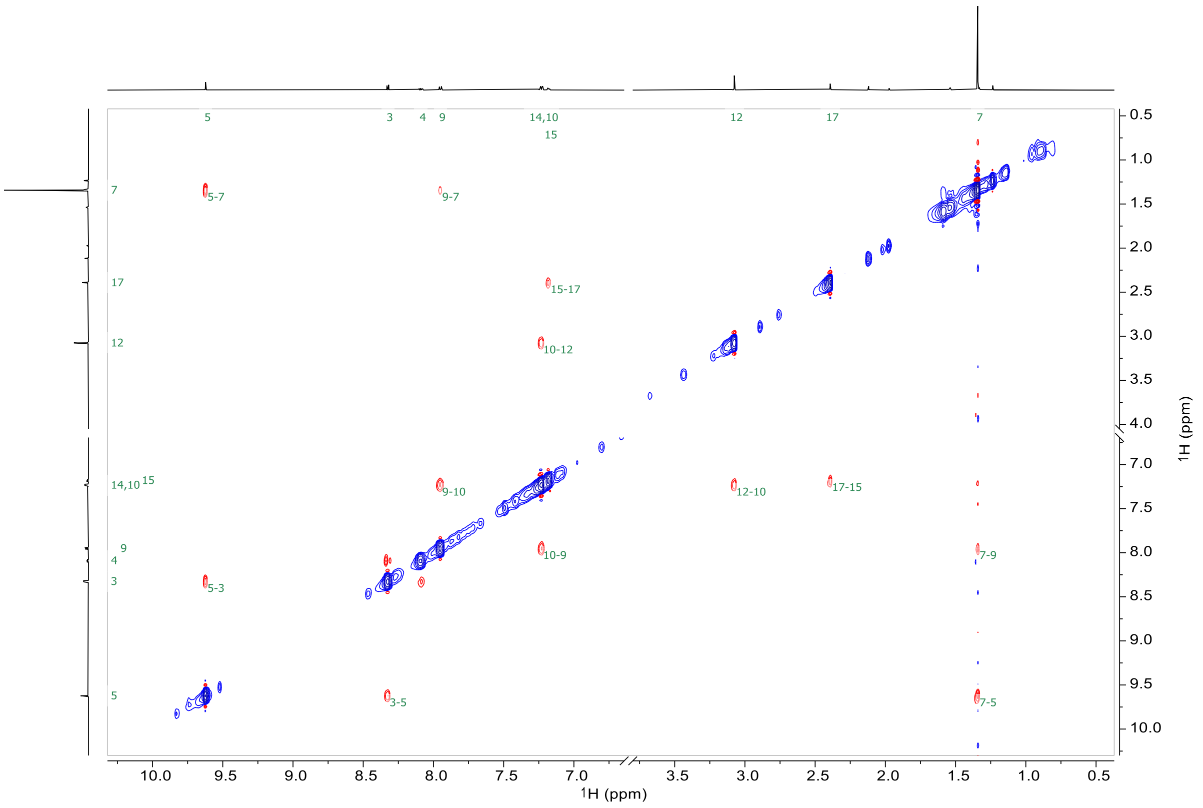

19F{off},1D, 564.72 MHz,CD2Cl2,298.0K, pulse sequence: zg30

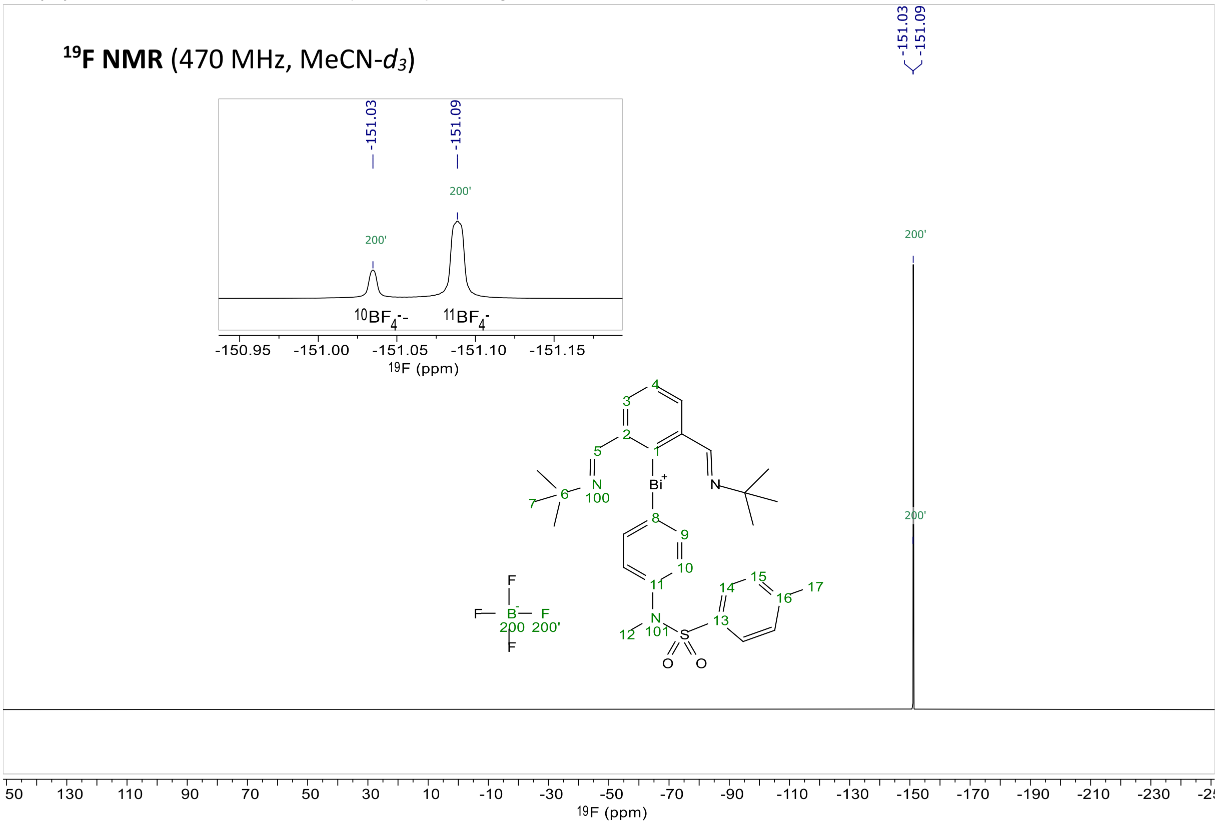

11B{off},1D, 192.57 MHz,CD2Cl2,298.0K, pulse sequence: zgbs

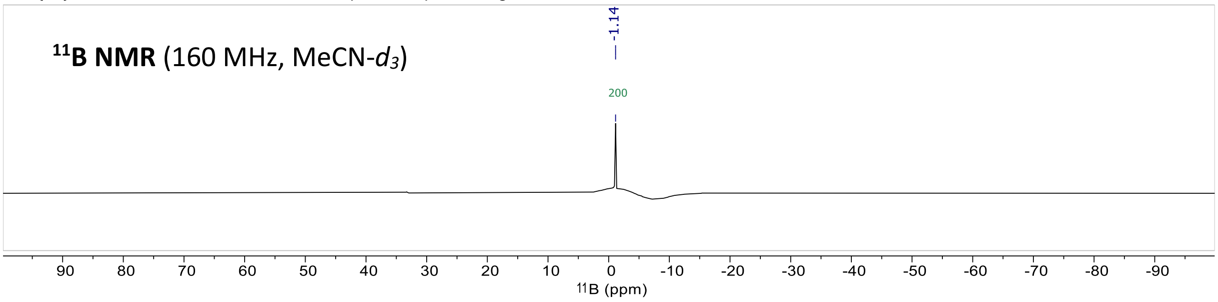

^{1}H{^{15}N} HMBC

1H{15N},HMBC, 600.20 MHz,CD2Cl2,298.0K, pulse sequence: hmbcgpndqf

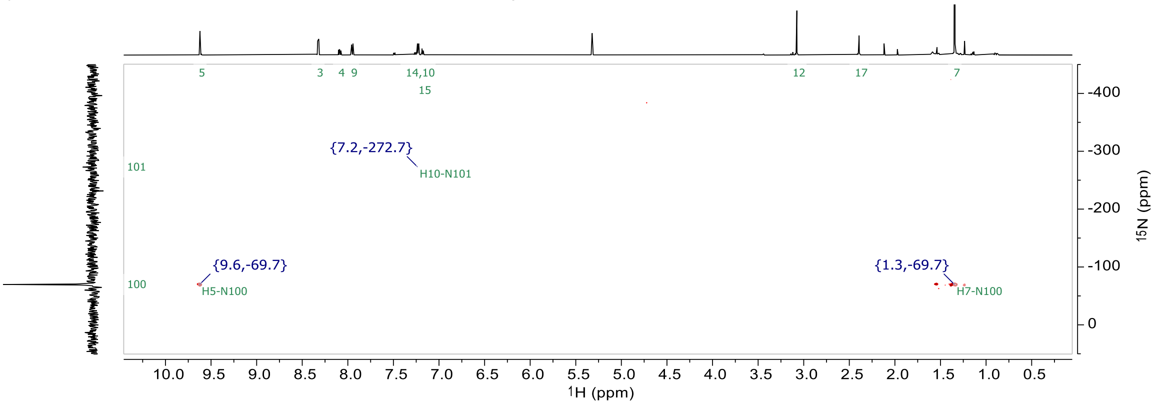

[(2,6-(*t*BuNCH)$_2$C$_6$H$_3$)Bi(4-cyanophenyl)(iodide)] (12a)

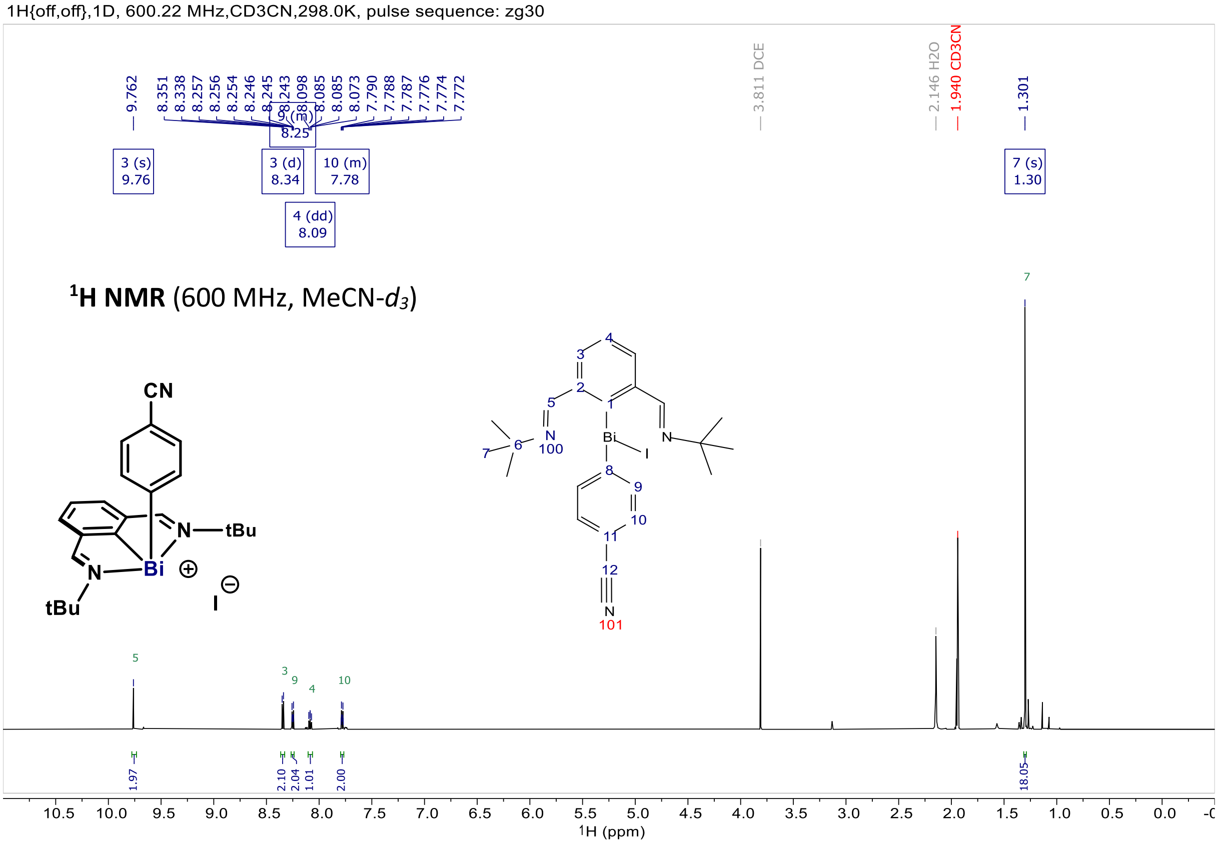

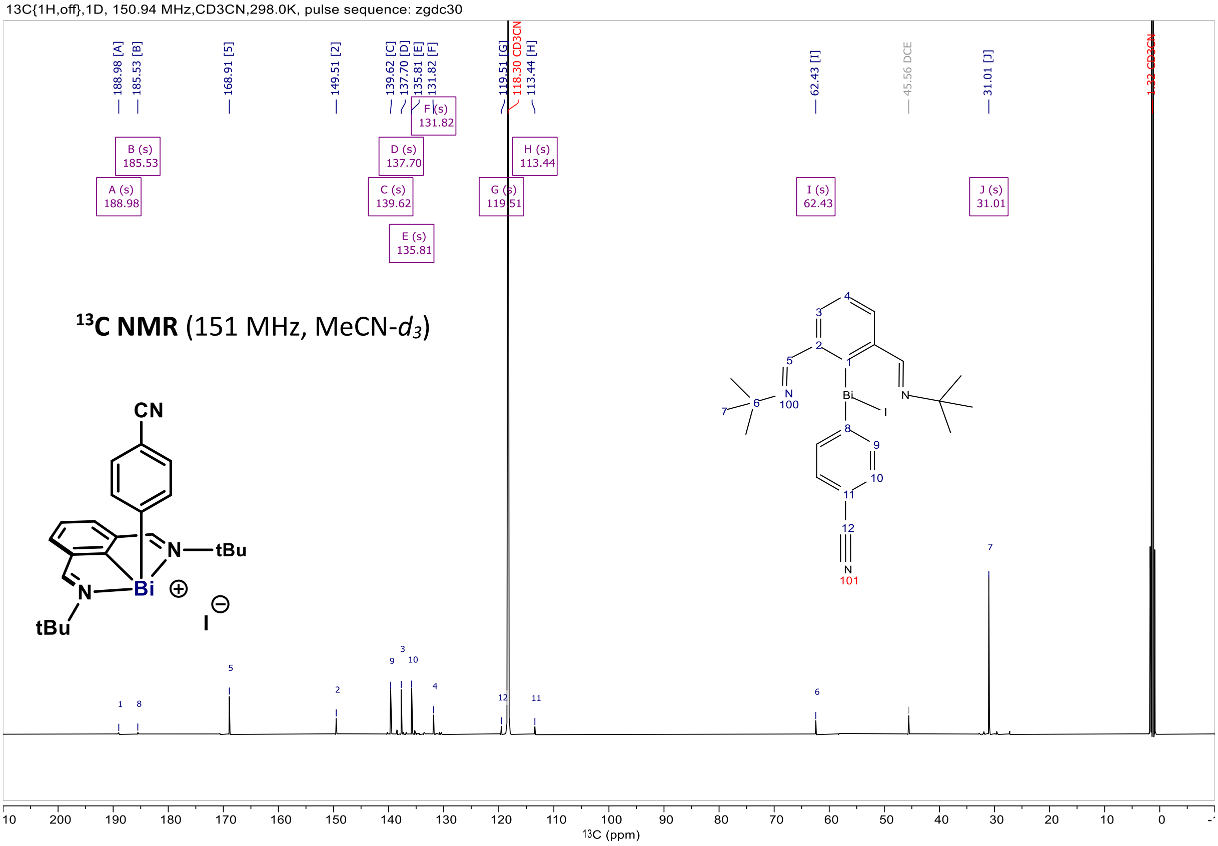

$^{1}H\{^{13}C\}$ HSQC

1H{13C,off},HSQC-EDITED, 600.22 MHz,CD3CN,298.0K, pulse sequence: hsqcedetgpsisp2.3

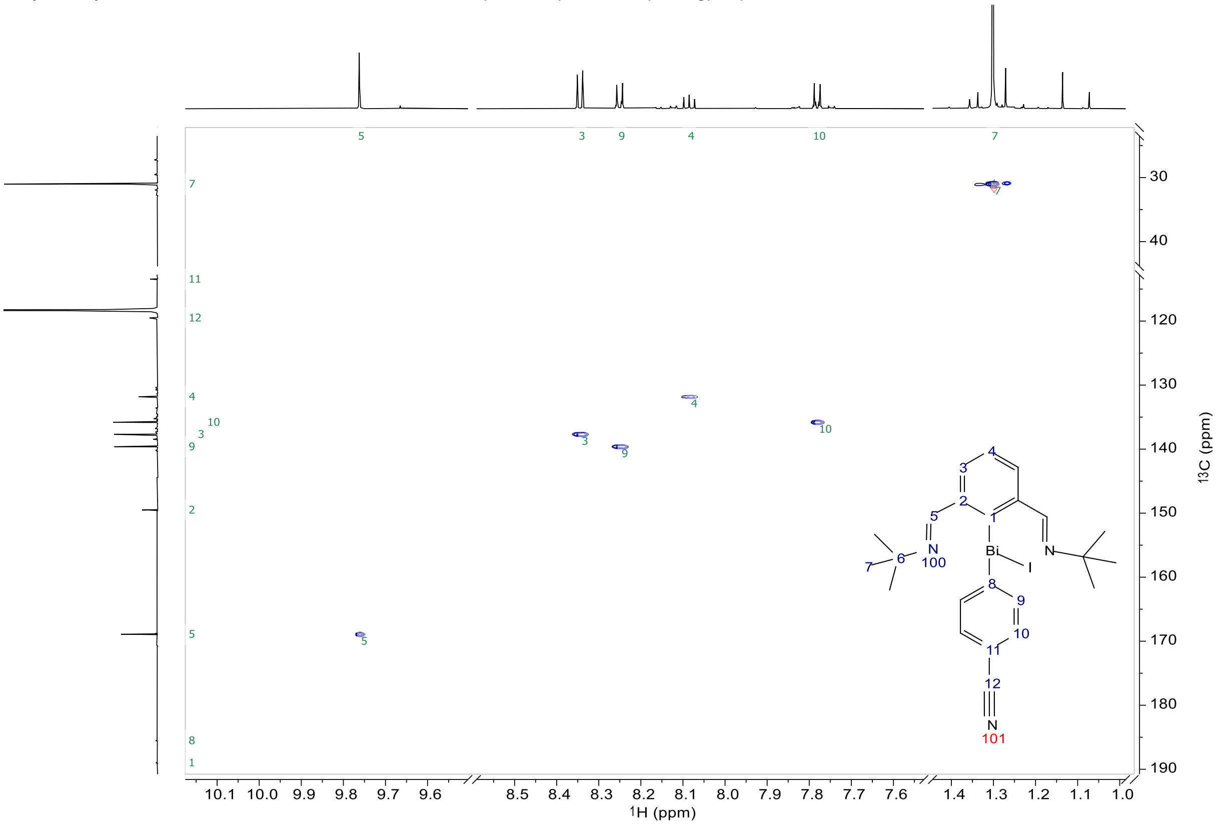

$^{1}H\{^{13}C\}$ HMBC

1H{13C,off},HMBC, 600.22 MHz,CD3CN,298.0K, pulse sequence: hmbcetgpl3nd

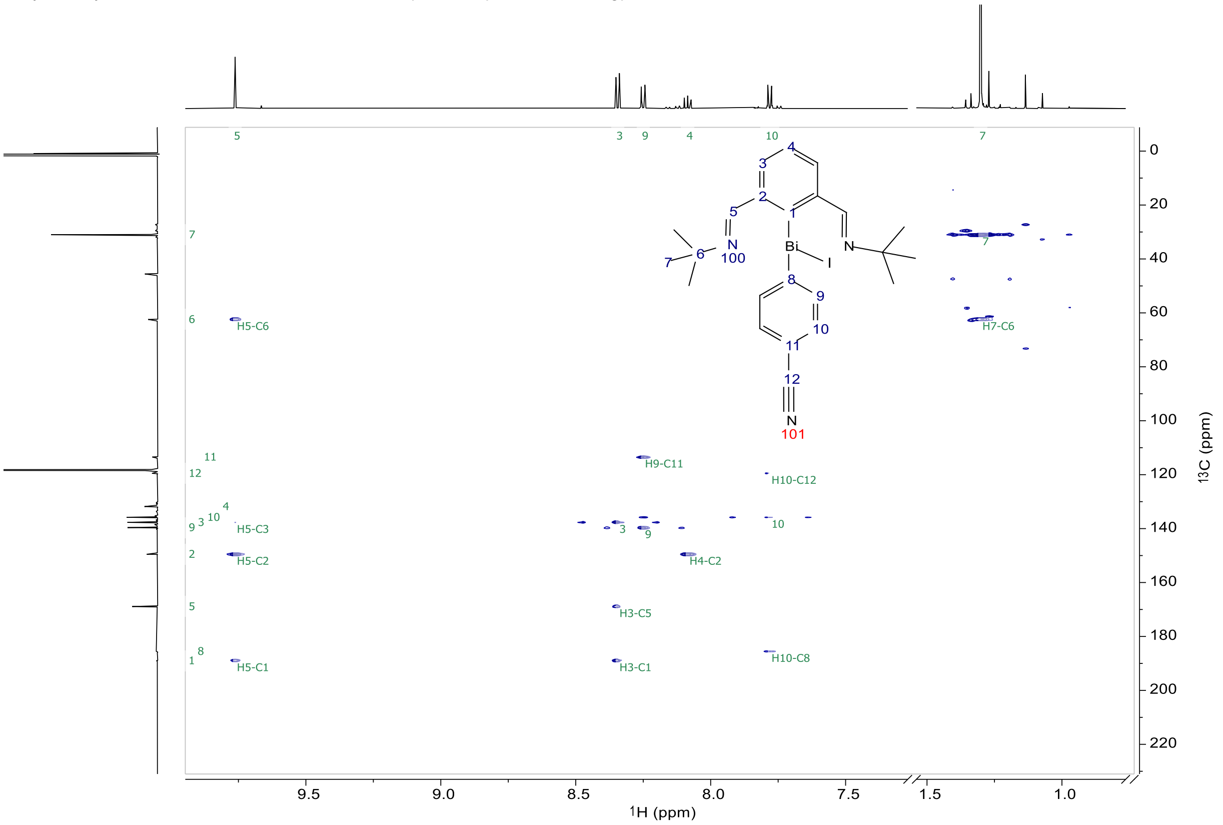

^{1}H COSY

1H{off,off},COSY, 600.22 MHz,CD3CN,298.0K, pulse sequence: cosygpppqf

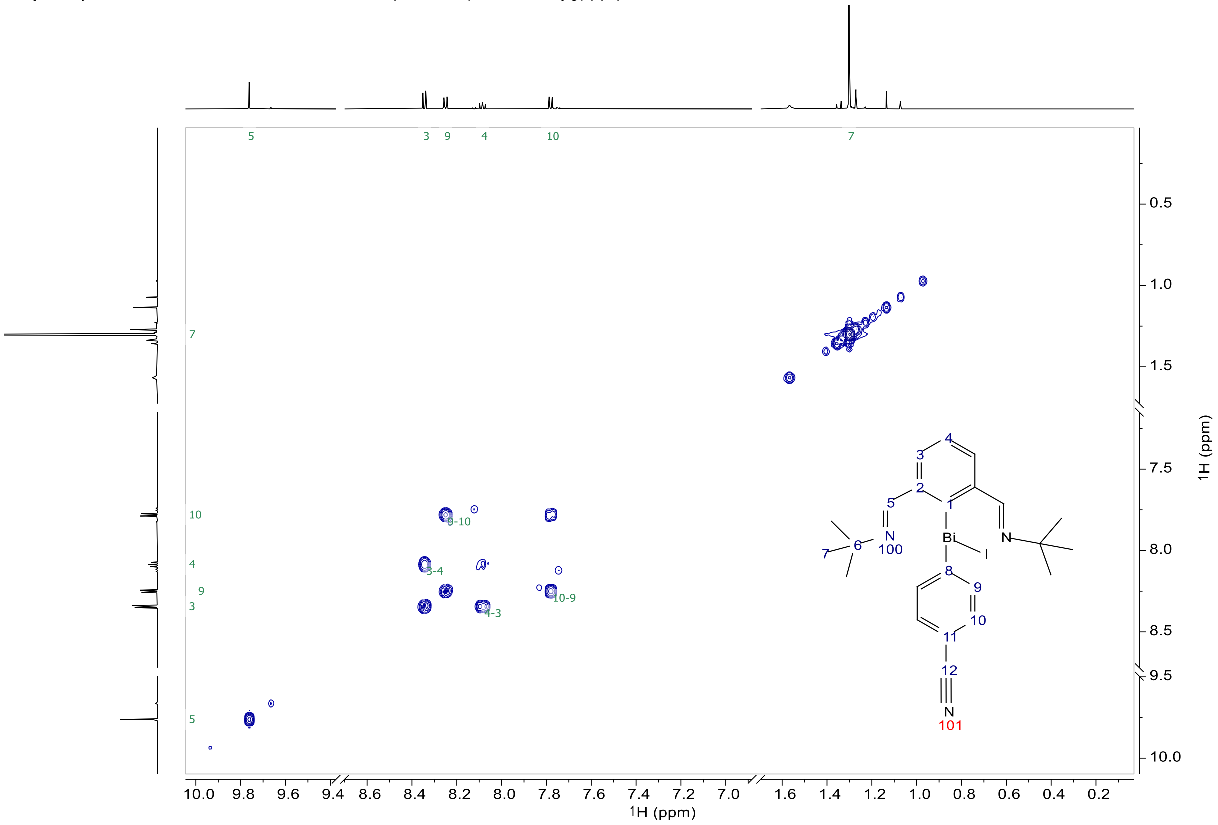

^{1}H NOESY

1H{off,off},NOESY, 600.22 MHz,CD3CN,298.0K, pulse sequence: noesygpph

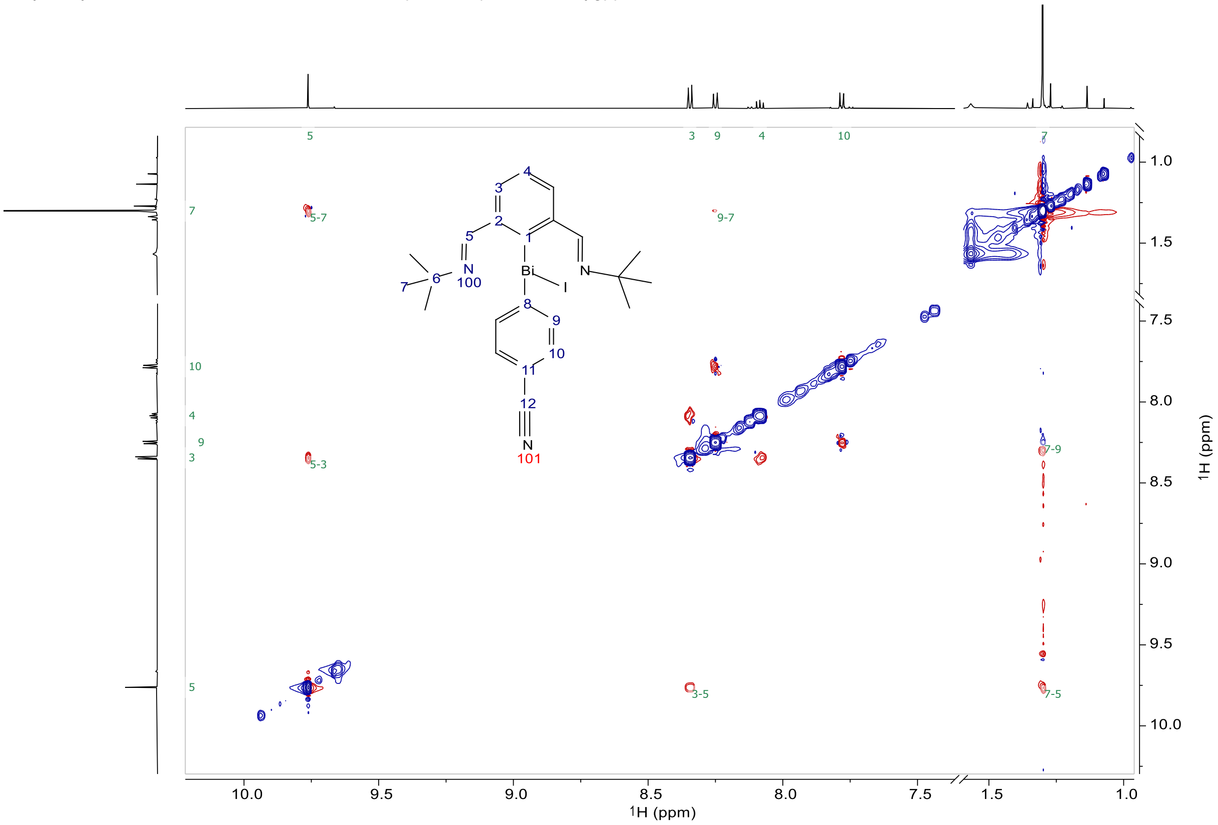

$^{1}H\{^{15}N\}$ HMBC

1H{off,15N},HMBC, 600.22 MHz,CD3CN,298.0K, pulse sequence: hmbcf3gpndqf

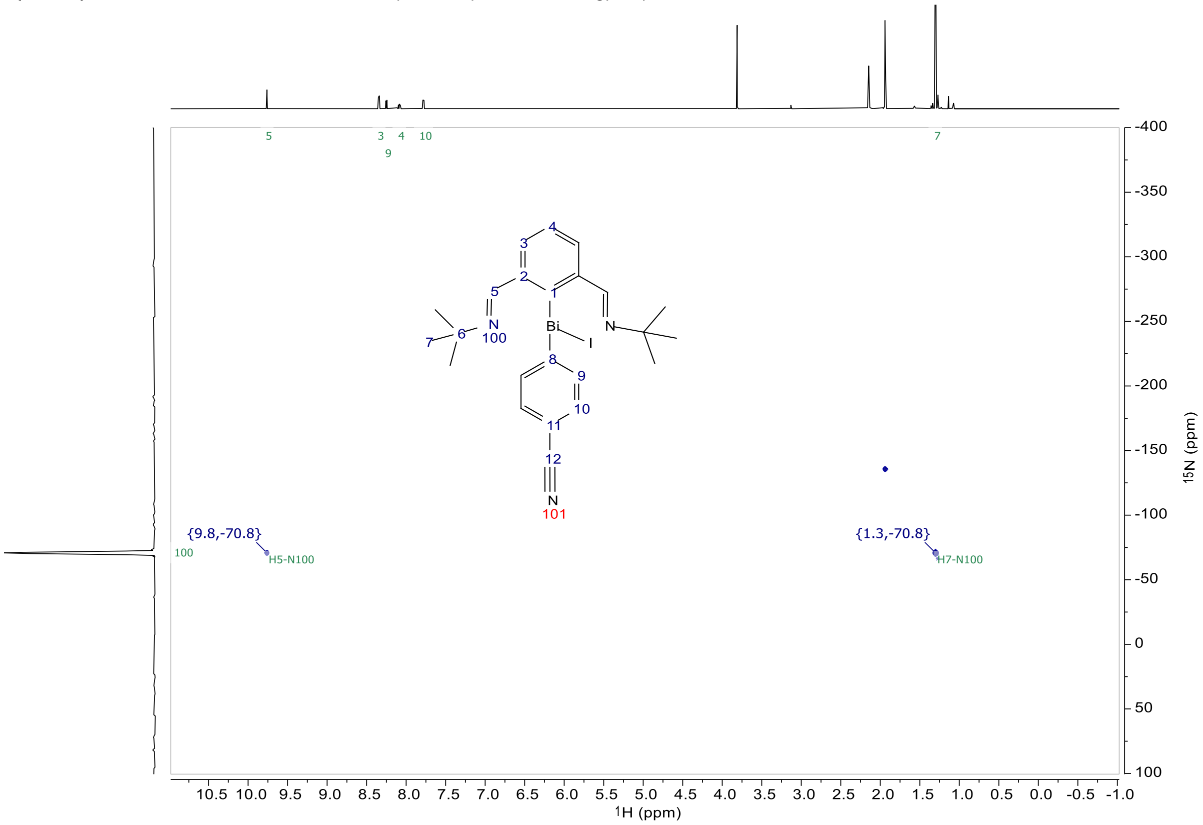

[(2,6-(*t*BuNCH)$_2$C$_6$H$_3$)Bi(1-naphthyl)(iodide)] (12b) (with 1 equiv excess unreacted Ar–I)

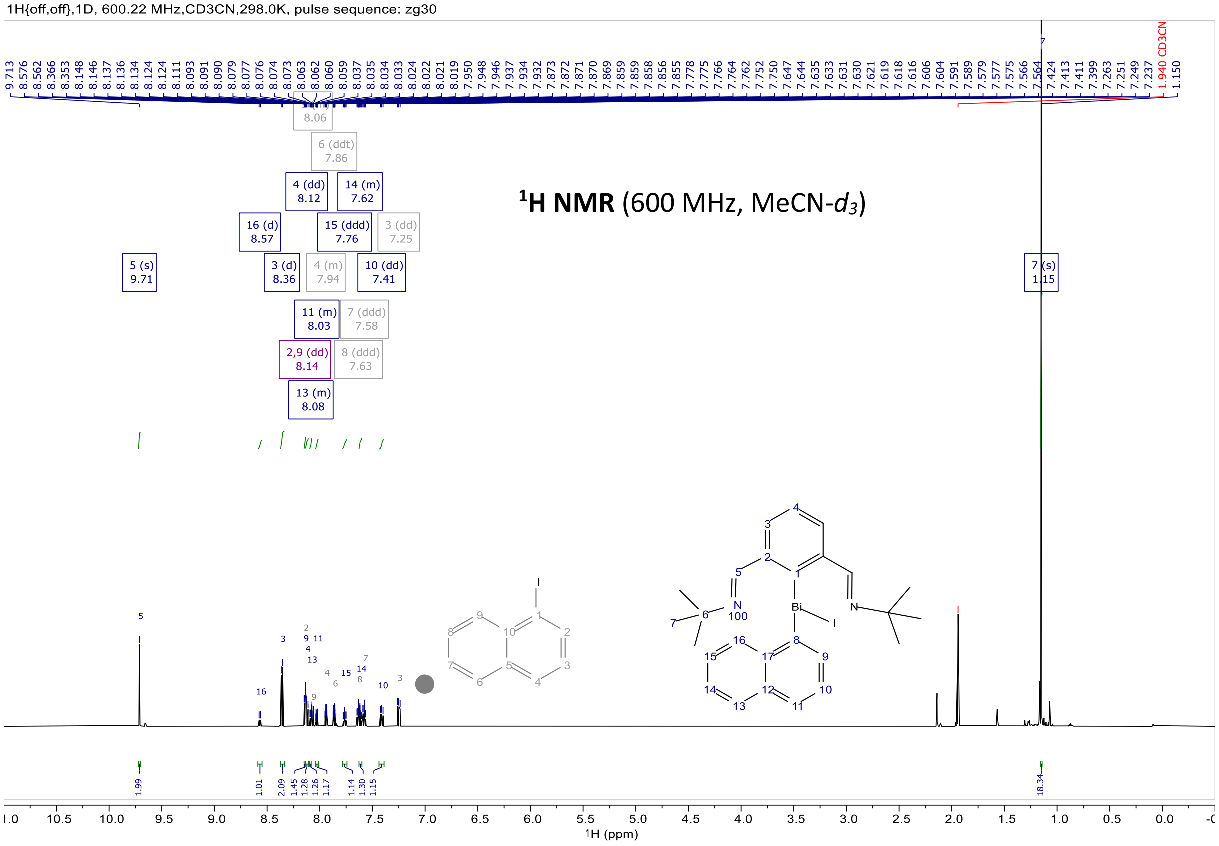

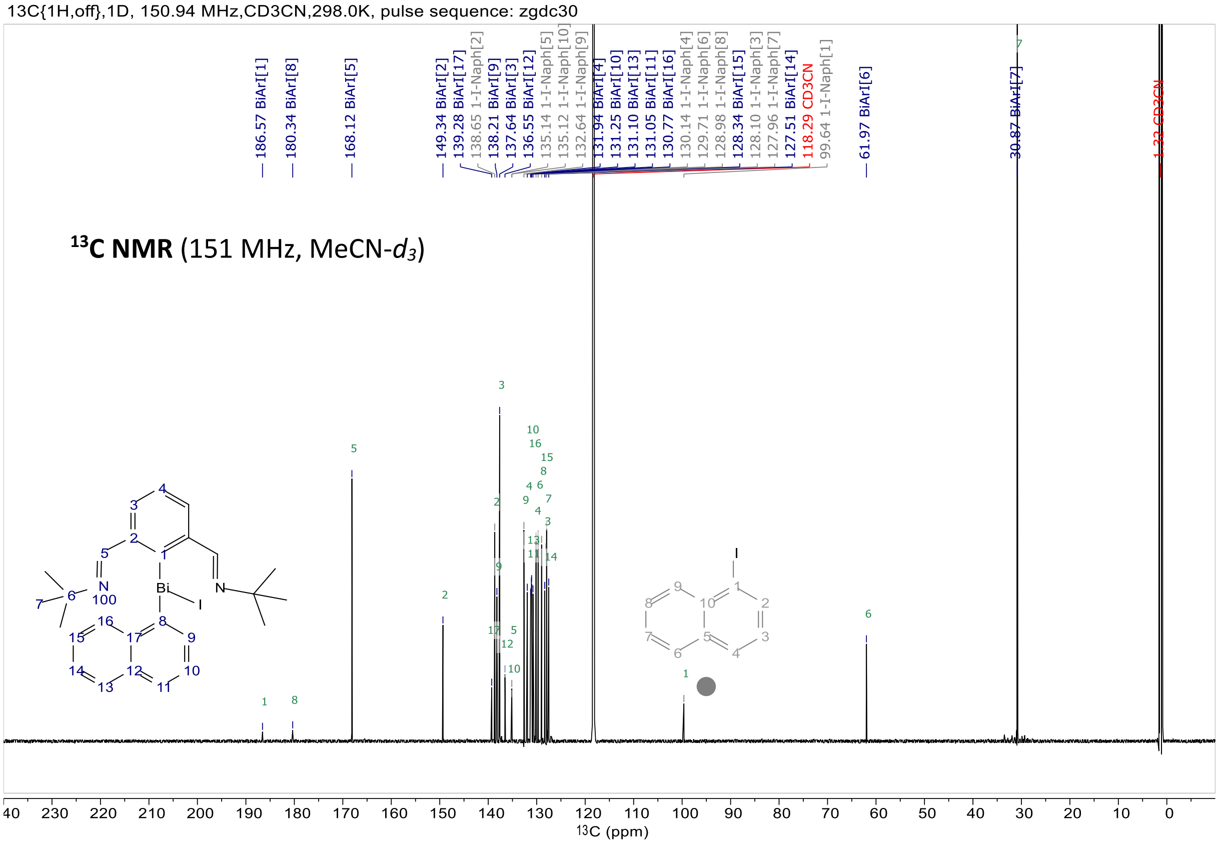

● With 1 equiv of excess 1-iodonaphthalene

[(2,6-(*t*BuNCH)$_2$C$_6$H$_3$)Bi(4-trifluoromethylphenyl)(iodide)] (12c)

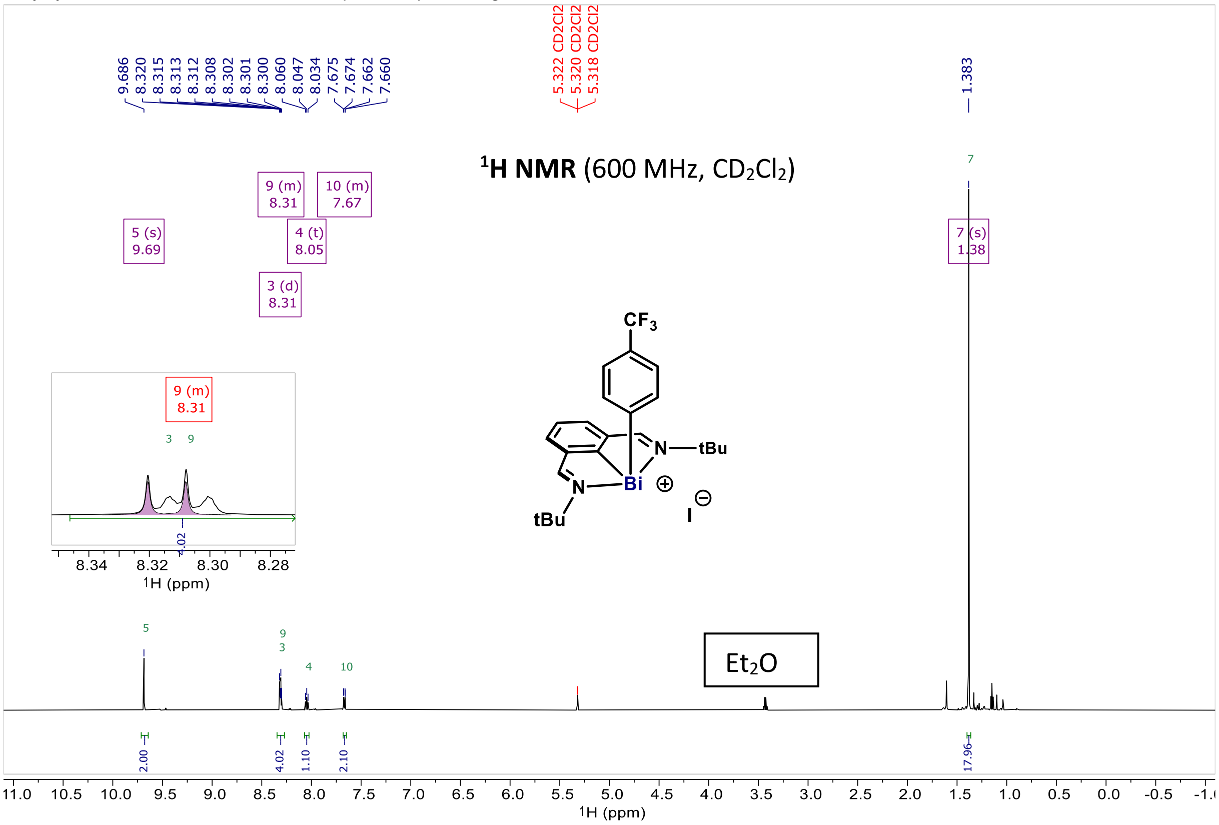

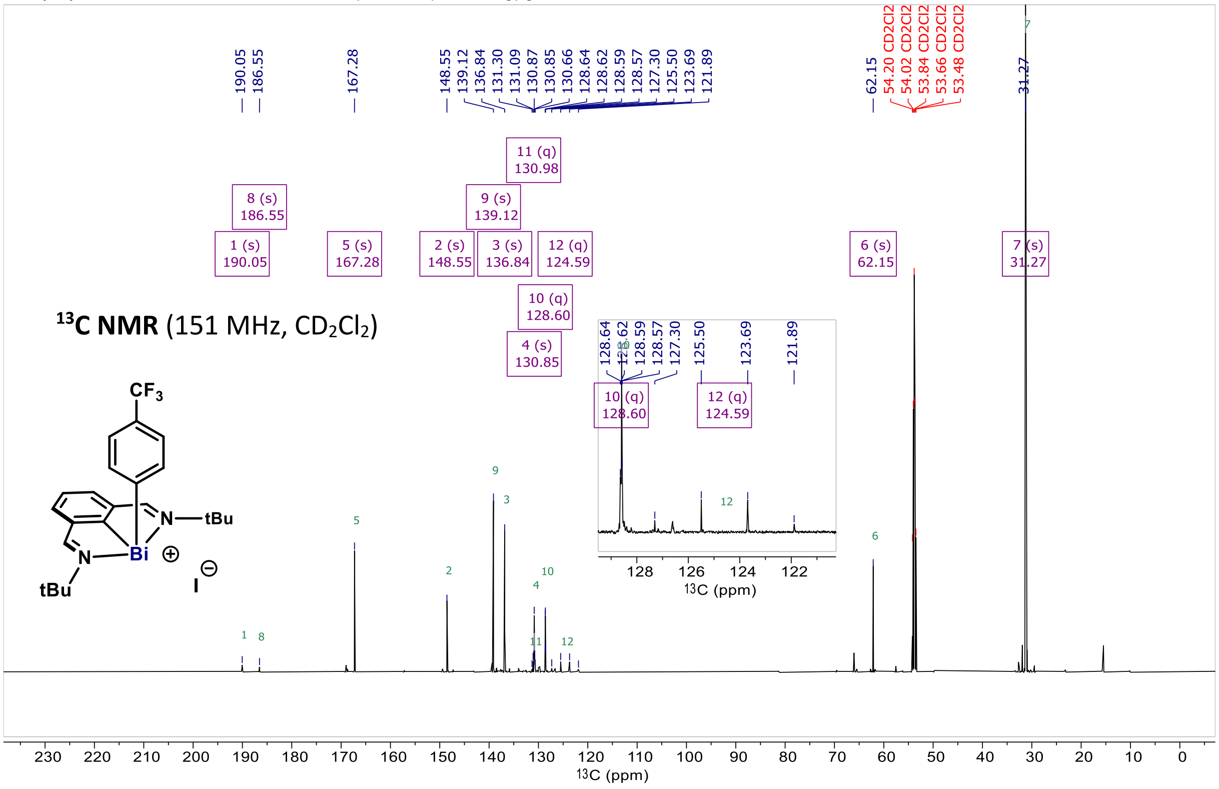

[(2,6-(*t*BuNCH)$_2$C$_6$H$_3$)Bi(4-trifluoromethylphenyl)(iodide)] (12c)

19F{off},1D, 564.72 MHz,CD2Cl2,298.0K, pulse sequence: zg30

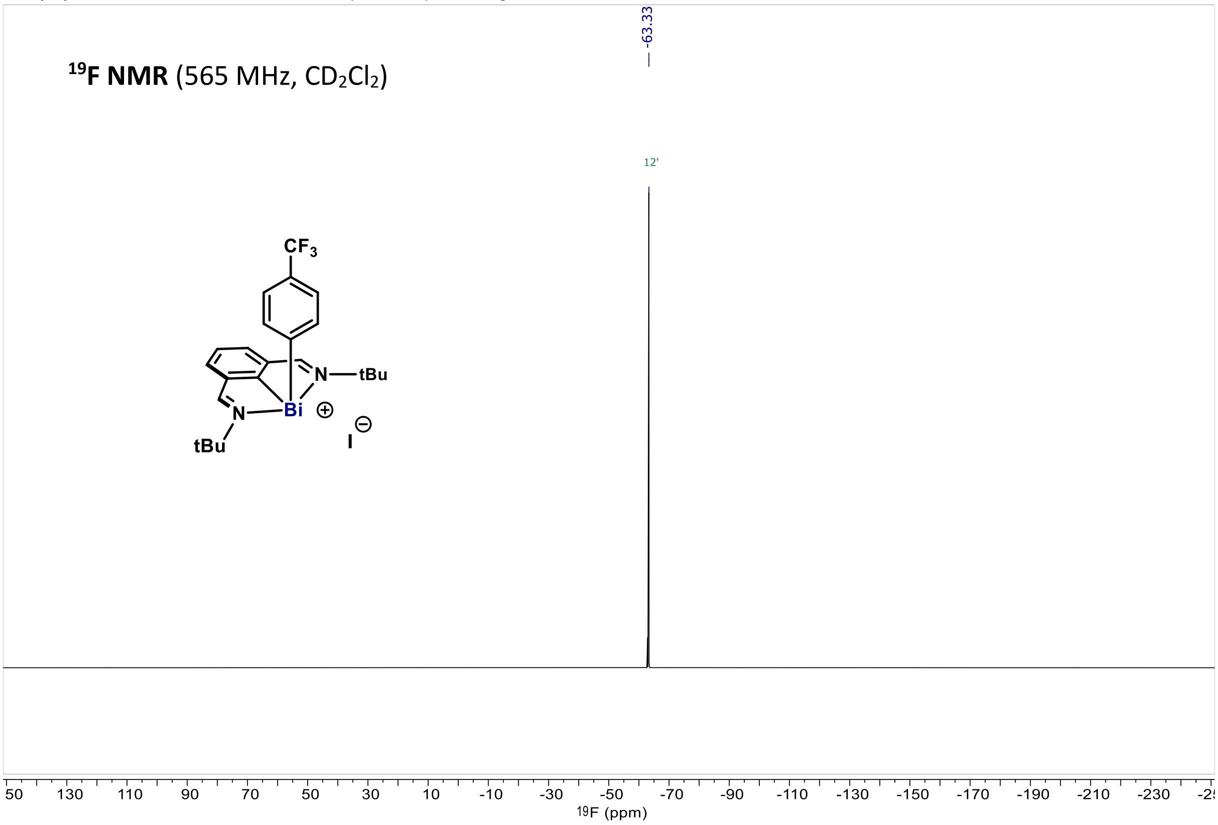

[(2,6-(*t*BuNCH)$_2$C$_6$H$_3$)Bi(4-chlorophenyl)(iodide)] (12d) (with 0.2 equiv excess unreacted Ar–I)

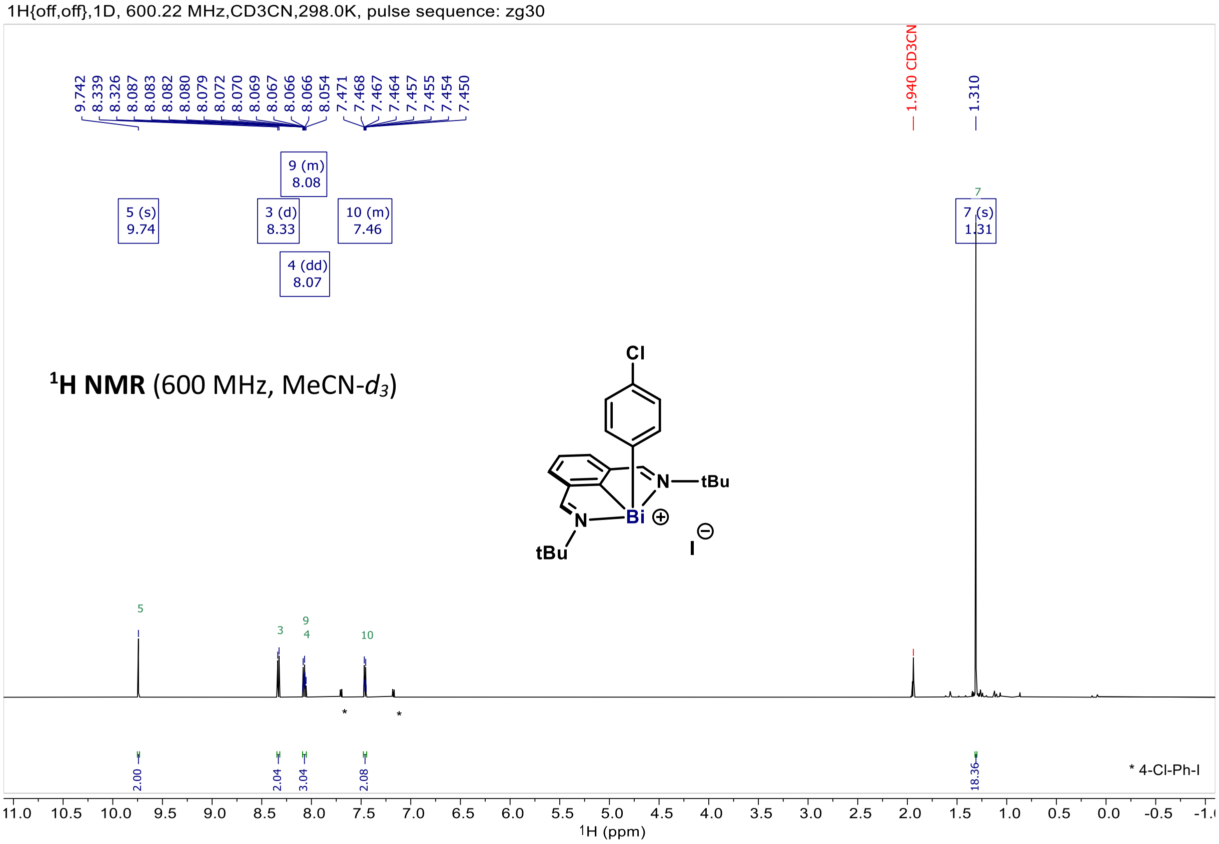

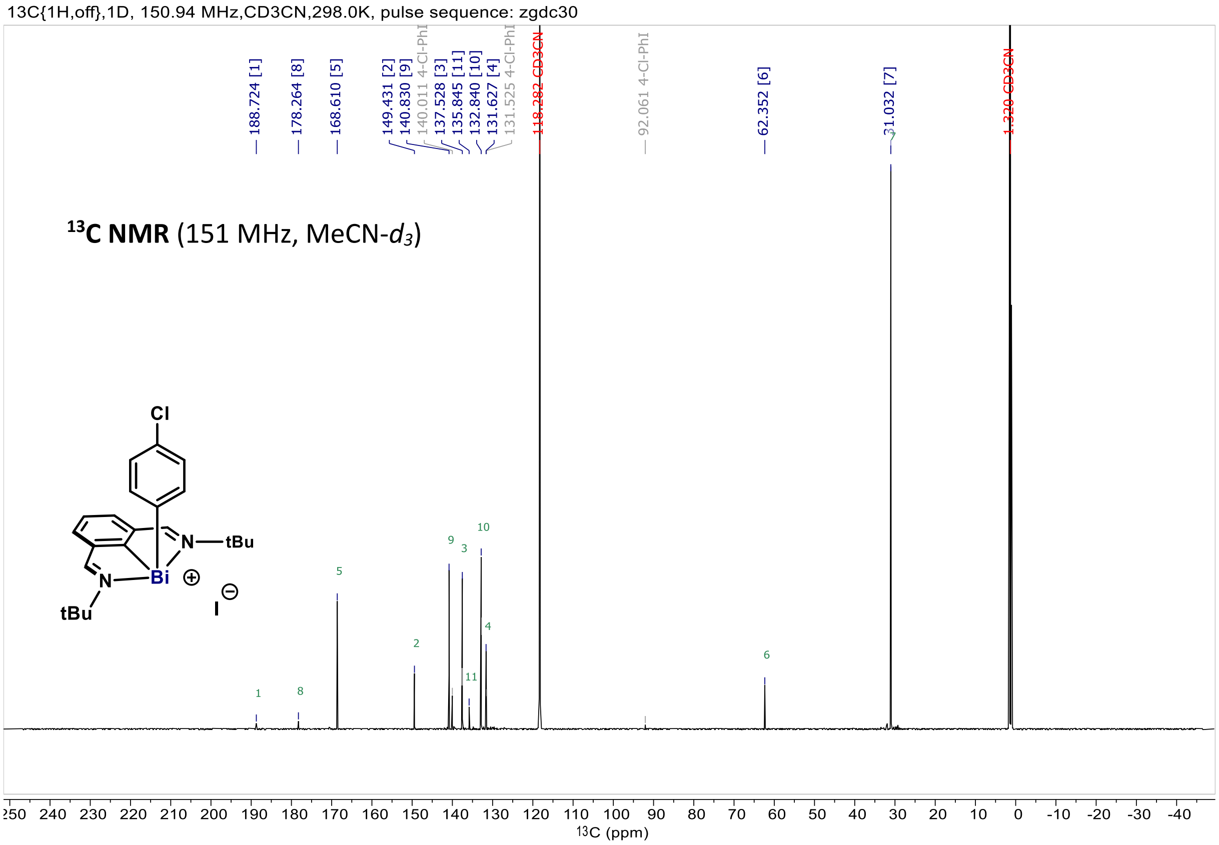

[(2,6-(*t*BuNCH)$_2$C$_6$H$_3$)Bi(4-bromophenyl)(iodide)] (**12e**) (with 0.2 equiv excess unreacted Ar–I)

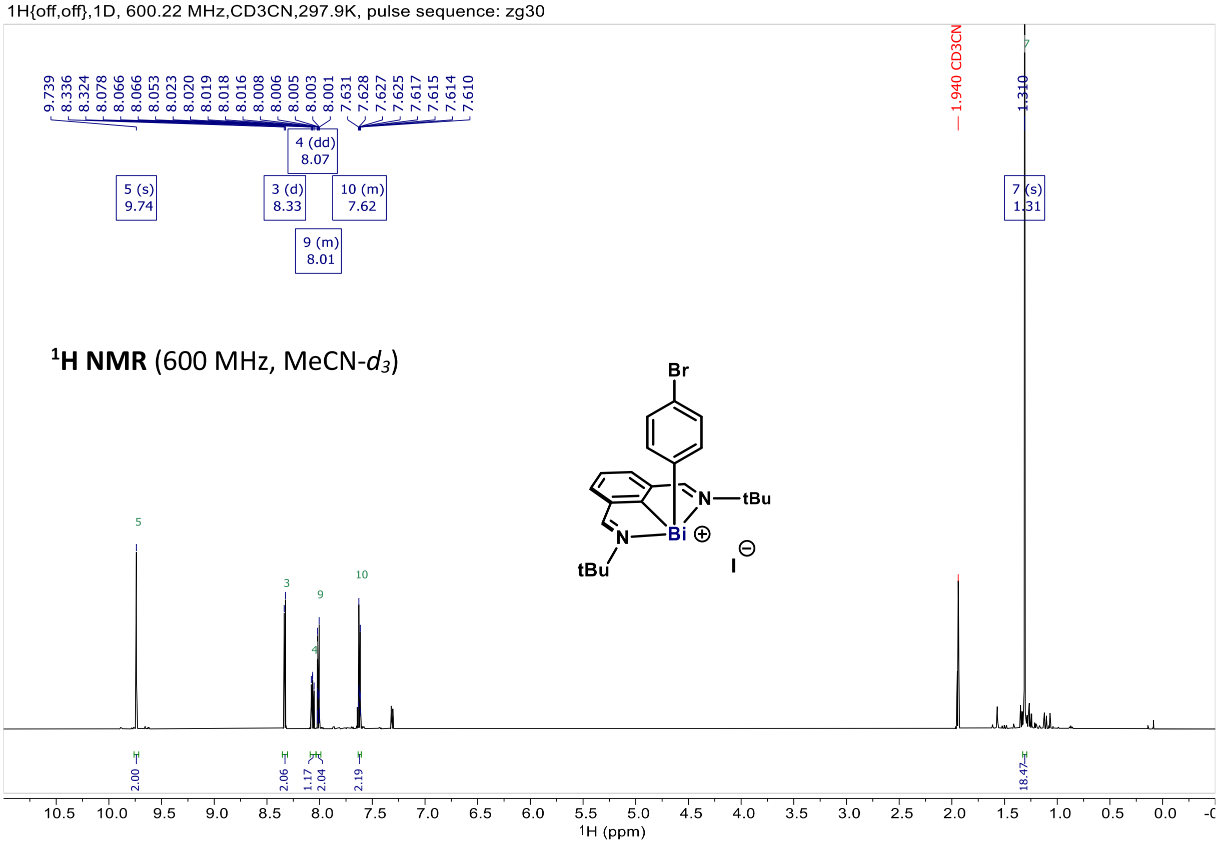

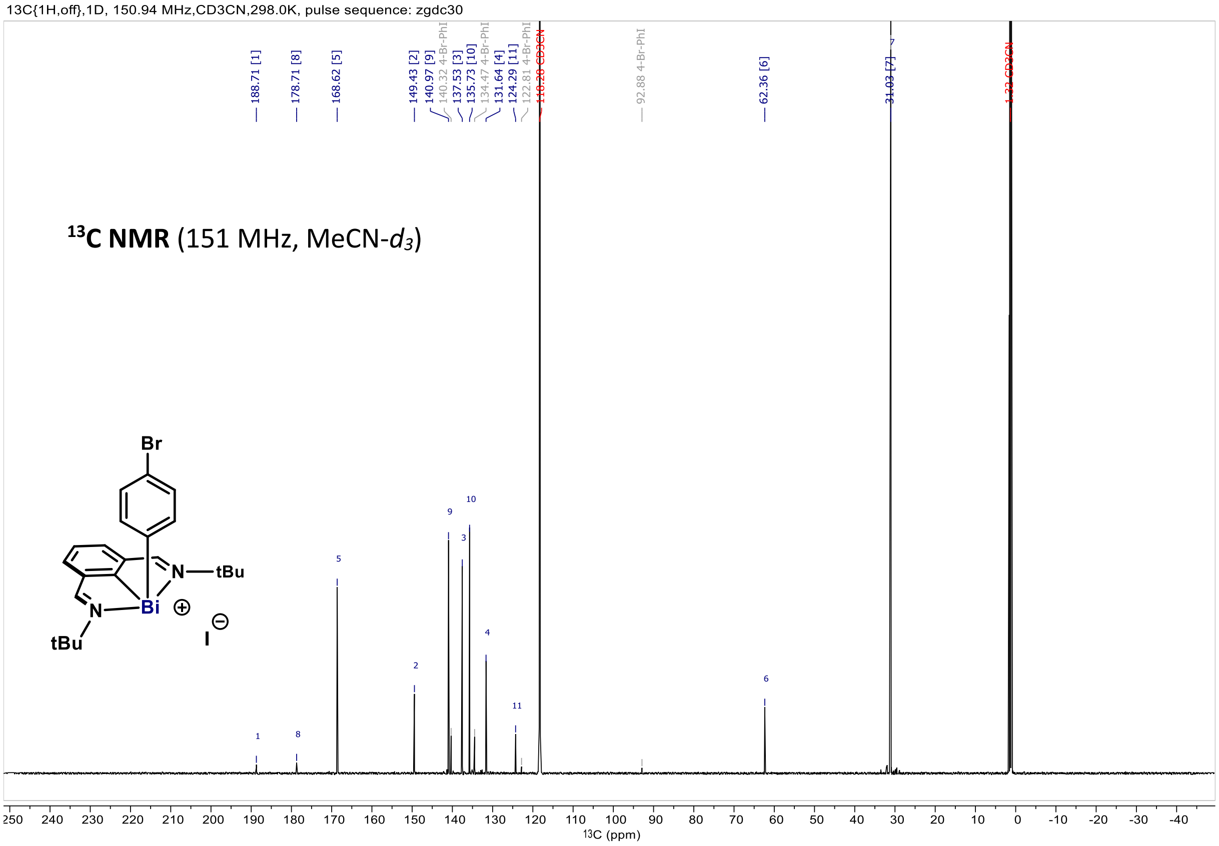

[(2,6-(*t*BuNCH)$_2$C$_6$H$_3$)Bi(4-fluorophenyl)(iodide)] (**12f**) (with 0.2 equiv excess unreacted Ar–I)

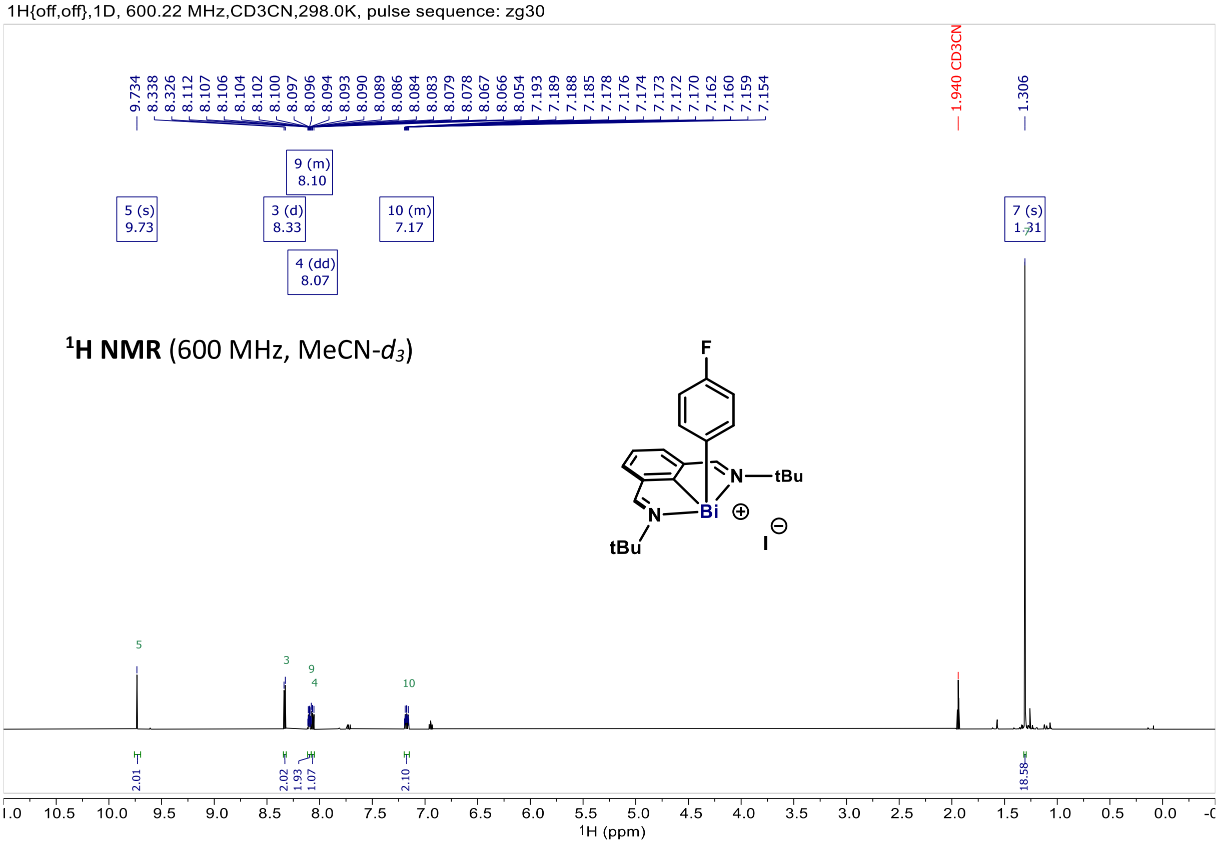

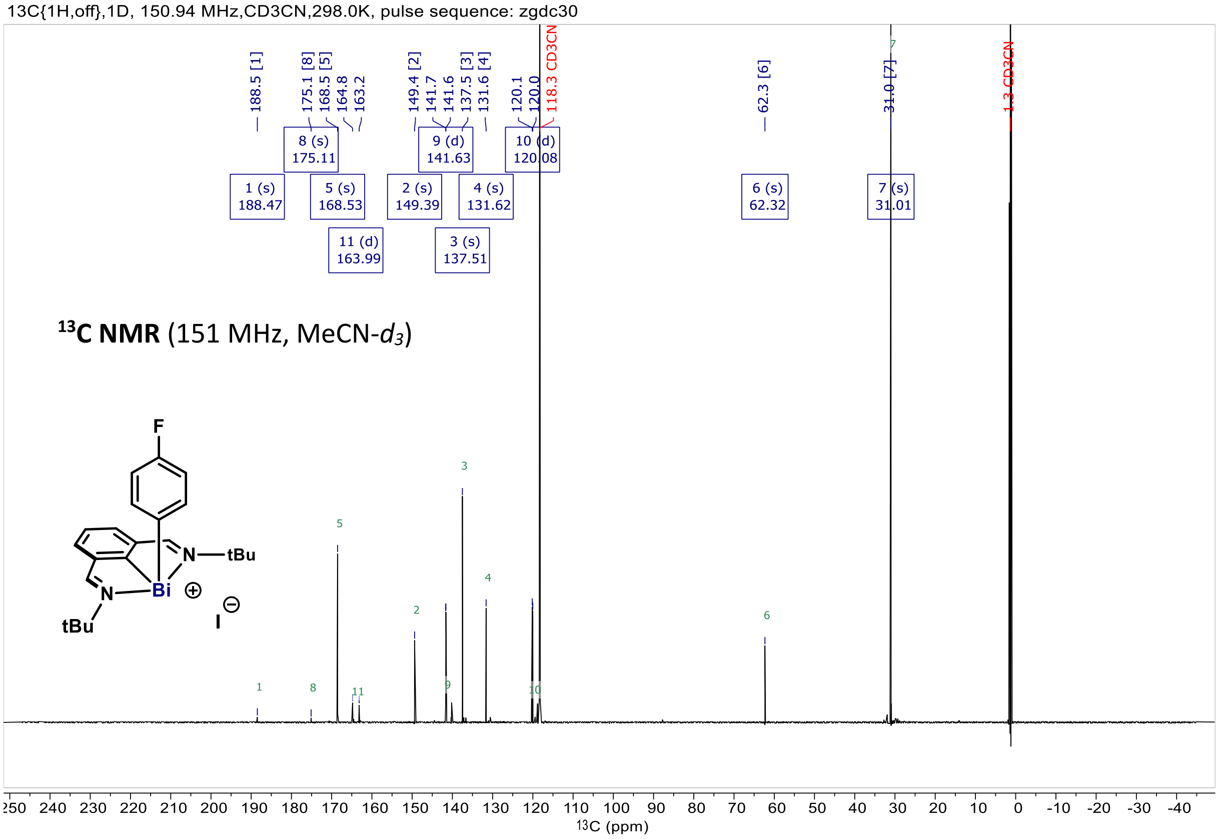

[(2,6-(tBuNCH)$_2$C$_6$H$_3$)Bi(4-fluorophenyl)(iodide)] (**12f**) (with 0.2 equiv excess unreacted Ar–I)

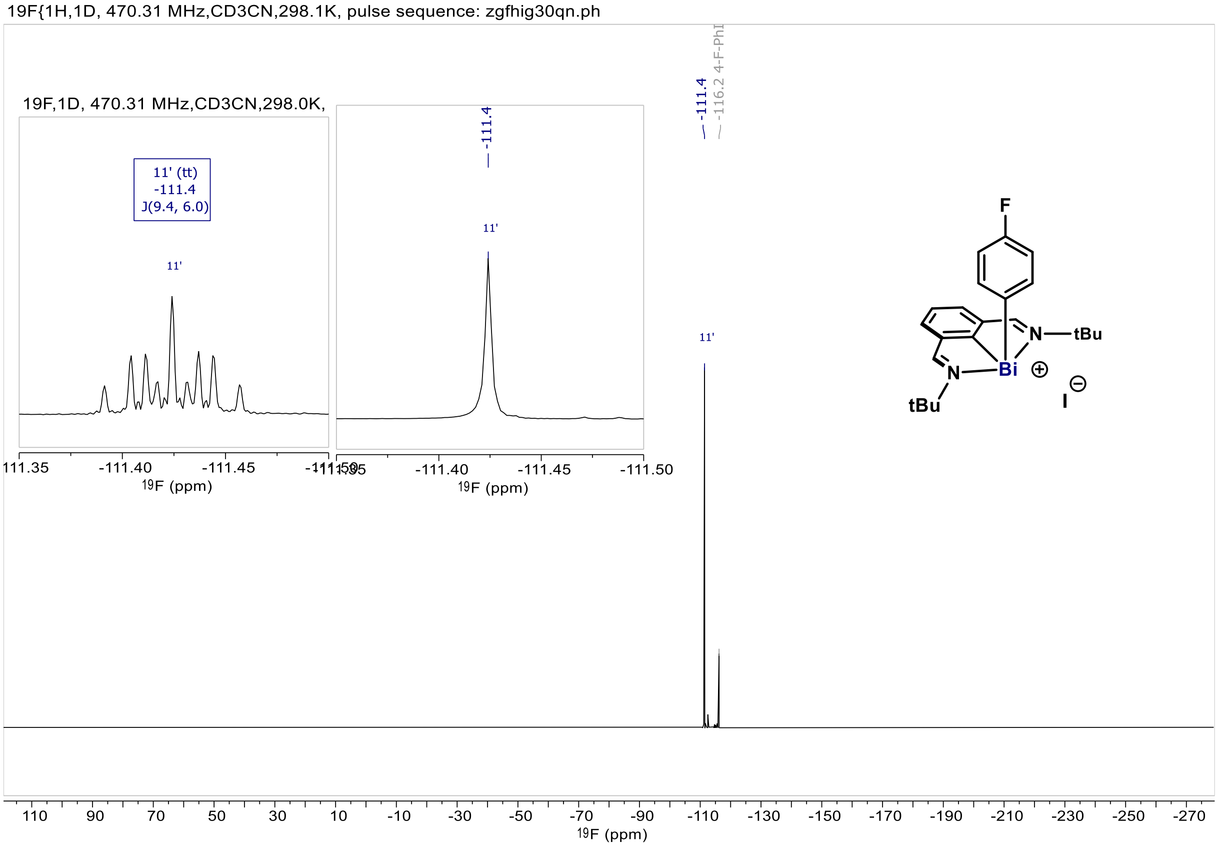

[(2,6-(*t*BuNCH)$_2$C$_6$H$_3$)Bi(4-methoxyphenyl)(iodide)] (12g) (with 0.5 equiv excess unreacted Ar–I)

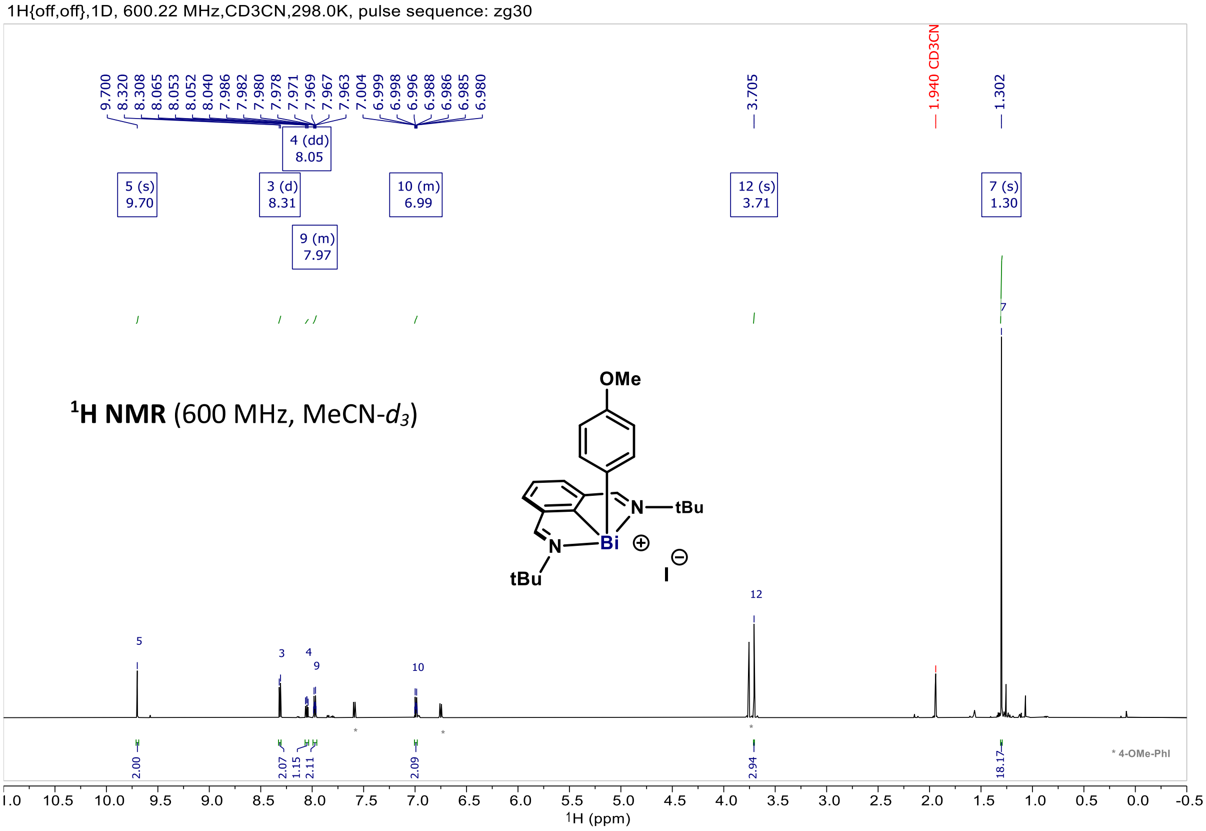

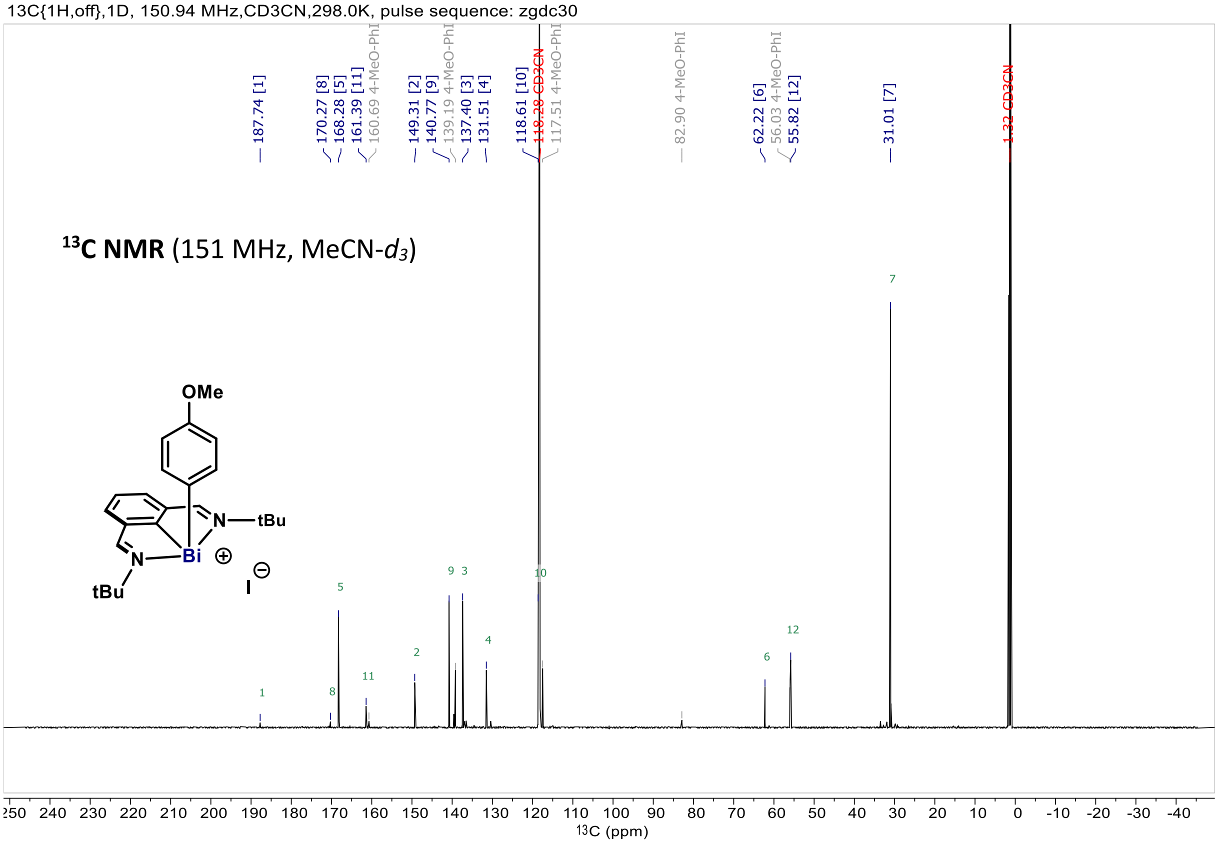

14. References

1. Vránová, I.; Alonso, M.; Lo, R.; Sedlák, R.; Jambor, R.; Růžička, A.; Dr Proft, F.; Hobza, P.; Dostál, L. From Dibismuthenes to Three- and Two-Coordinated Bismuthinidenes by Fine Ligand Tuning: Evidence for Aromatic BiC_3N Rings through a Combined Experimental and Theoretical Study. *Chem. Eur. J.* **2015**, *21*, 16917–16928.

2. Wang, F.; Planas, O.; Cornella, J. Bi(I)-Catalyzed Transfer-Hydrogenation with Ammonia-Borane. *J. Am. Chem. Soc.* **2019**, *141*, 4235–4240.

3. Pang, Y.; Leutzsch, M.; Nöthling, N.; Katzenburg, F.; Cornella, J. Catalytic Hydrodefluorination via Oxidative Addition, Ligand Metathesis, and Reductive Elimination at Bi(I)/Bi(III) Centers. *J. Am. Chem. Soc.* **2021**, *143*, 12487–12493.

4 Mato, M.; Spinnato, D.; Leutzsch, M.; Moon, H. W.; Reijerse, E. J.; Cornella, J. Bismuth radical catalysis in the activation and coupling of redox-active electrophiles. *Nat. Chem.* **2023**, DOI: 10.1038/s41557-023-01229-7.

5. (a) Ghiazza, C.; Debrauwer, V.; Monnereau, C.; Khrouz, L.; Médebielle, M.; Billard, T.; Tlili, A. Visible-Light-Mediated Metal-Free Synthesis of Trifluoromethylselenolated Arenes. *Angew. Chem. Int. Ed.* **2018**, *57*, 11781–11785. (b) Erb, W.; Hellal, A.; Albini, M.; Rouden, J.; Blanchet, J. *Chem. Eur. J.* **2014**, *20*, 6608–6612. (c) Liu, A.-D.; Wang, Z.-L.; Liu, L.; Cheng, L. Aqueous and Visible-Light-Promoted C–H (Hetero)arylation of Uracil Derivatives with Diazoniums. *J. Org. Chem.* **2021**, *86*, 16434–16447.

6. Wang, M.; Zhang, X.; Ma, M.; Zhao, B. Palladium-Catalyzed Synthesis of Esters from Arenes through C–H Thianthrenation. *Org. Lett.* **2022**, *24*, 6031–6036.

7. Lansbergen, B.; Granatino, P.; Ritter, T. Site-Selective C–H Alkylation of Complex Arenes by a Two-Step Aryl Thianthrenation-Reductive Alkylation Sequence. *J. Am. Chem. Soc.* **2021**, *143*, 7090–7914.

8 Pavlishchuk, V. V.; Addison, A. W. Conversion constants for redox potentials measured versus different reference electrodes in acetonitrile solutions at 25°C. *Inorganica Chim. Acta* **2000**, *298*, 97–102.

9 Li, J.; Chen, J.; Sang, R.; Ham, W.-S.; Plutschack, M. B.; Berger, F.; Chabbra, S.; Schnegg, A.; Genicot, C.; Ritter, T. *Nat. Chem.* **2020**, *12*, 56–62.

10 Firth, J. D.; Hammarback, L. A.; Burden, T. J.; Eastwood, J. B.; Donald, J. R.; Horbaczewskyj, C. S.; McRobie, M. T.; Tramaseur, A.; Clark, I. P.; Towrie, M.; Robinson, A.; Krieger, J.-P.; Lynam, J. M.; Fairlamb, I. J. S. Light-

and Manganese-Initiated Borylation of Aryl Diazonium Salts: Mechanistic Insight on the Ultrafast Time-Scale Revealed by Time-Resolved Spectroscopic Analysis. *Chem. Eur. J.* **2021**, *27*, 3979–3985.

11 Stoll, S.; Schweiger, A. EasySpin, a comprehensive software package for spectral simulation and analysis in EPR. *Journal of Magnetic Resonance* **2006**, *178*, 42–55.

12 Ionita, G.; Caragheorgheopol, A.; Caldararu, H.; Jones, L.; Chechik, V. Inclusion complexes of cyclodextrins with nitroxide-based spin probes in aqueous solutions. *Organic & Biomolecular Chemistry* **2009**, *7*, 598–602.

13 (a) Gannett, P. M.; Shi, X.; Lawson, T.; Kolar, C.; Toth, B. Aryl Radical Formation during the Metabolism of Arylhydrazines by Microsomes. *Chemical Research in Toxicology* **1997**, *10*, 1372–1377. (b) Reszka, K. J.; Chignell, C. F. One-electron reduction of arenediazonium compounds by physiological electron donors generates aryl radicals. An EPR and spin trapping investigation. *Chemico-Biological Interactions* **1995**, *96*, 223–234.

14 (a) Novak, M.; Brodeur, B. A. ESR spin-trapping study of the thermal decomposition of N-pivaloxyacetanilides in benzene. *The Journal of Organic Chemistry* **1984**, *49*, 1142–1144. (b) Liu, X.; Conte, M.; He, Q.; Knight, D. W.; Murphy, D. M.; Taylor, S. H.; Whiston, K.; Kiely, C. J.; Hutchings, G. J. Catalytic Partial Oxidation of Cyclohexane by Bimetallic Ag/Pd Nanoparticles on Magnesium Oxide. *Chemistry – A European Journal* **2017**, *23*, 11834–11842.

15 Kearvell, A.; Wilkinson, F. Fluorescence Quenching and External Spin-Orbit Coupling Effects. *Molecular Crystals* **1968**, *4*, 69–81.

16 (a) Neese, F.; Wennmohs, F.; Becker, U.; Riplinger, C. The ORCA quantum chemistry program package. *The Journal of Chemical Physics* **2020**, *152*, 224108. (b) Neese, F. Software update: The ORCA program system—Version 5.0. *WIREs Computational Molecular Science* **2022**, *12*, e1606.

17 (a) van Wüllen, C. Molecular density functional calculations in the regular relativistic approximation: Method, application to coinage metal diatomics, hydrides, fluorides and chlorides, and comparison with first-order relativistic calculations. *The Journal of Chemical Physics* **1998**, *109*, 392–399. (b) van Lenthe, E.; Snijders, J. G.; Baerends, E. J. The zero-order regular approximation for relativistic effects: The effect of spin–orbit coupling in closed shell molecules. *The Journal of Chemical Physics* **1996**, *105*, 6505–6516.

18 (a) Lee, C.; Yang, W.; Parr, R. G. Development of the Colle-Salvetti correlation-energy formula into a functional of the electron density. *Physical Review B* **1988**, *37*, 785–789. (b) Becke, A. D. Density-functional thermochemistry. III. The role of exact exchange. *The Journal of Chemical Physics* **1993**, *98*, 5648–5652.

19 (a) Grimme, S.; Antony, J.; Ehrlich, S.; Krieg, H. A consistent and accurate ab initio parametrization of density functional dispersion correction (DFT-D) for the 94 elements H-Pu. *The Journal of Chemical Physics* **2010**, *132*. (b) Grimme, S.; Ehrlich, S.; Goerigk, L. Effect of the damping function in dispersion corrected density functional theory. *Journal of Computational Chemistry* **2011**, *32*, 1456–1465.

20 Weigend, F.; Ahlrichs, R. Balanced basis sets of split valence, triple zeta valence and quadruple zeta valence quality for H to Rn: Design and assessment of accuracy. *Physical Chemistry Chemical Physics* **2005**, *7*, 3297–3305.

21 Pantazis, D. A.; Neese, F. All-electron scalar relativistic basis sets for the 6p elements. *Theoretical Chemistry Accounts* **2012**, *131*, 1292.

22 (a) Weigend, F. Accurate Coulomb-fitting basis sets for H to Rn. *Physical Chemistry Chemical Physics* **2006**, *8*, 1057–1065. (b) Neese, F.; Wennmohs, F.; Hansen, A.; Becker, U. Efficient, approximate and parallel Hartree–Fock and hybrid DFT calculations. A 'chain-of-spheres' algorithm for the Hartree–Fock exchange. *Chemical Physics* **2009**, *356*, 98–109. (c) Eichkorn, K.; Weigend, F.; Treutler, O.; Ahlrichs, R. Auxiliary basis sets for main row atoms and transition metals and their use to approximate Coulomb potentials. *Theoretical Chemistry Accounts* **1997**, *97*, 119–124.

23 (a) Rolfes, J. D.; Neese, F.; Pantazis, D. A. All-electron scalar relativistic basis sets for the elements Rb–Xe. *Journal of Computational Chemistry* **2020**, *41*, 1842–1849. (b) Aravena, D.; Neese, F.; Pantazis, D. A. Improved Segmented All-Electron Relativistically Contracted Basis Sets for the Lanthanides. *Journal of Chemical Theory and Computation* **2016**, *12*, 1148–1156. (c) Pantazis, D. A.; Chen, X.-Y.; Landis, C. R.; Neese, F. All-Electron Scalar Relativistic Basis Sets for Third-Row Transition Metal Atoms. *Journal of Chemical Theory and Computation* **2008**, *4*, 908–919.

24 Bykov, D.; Petrenko, T.; Izsák, R.; Kossmann, S.; Becker, U.; Valeev, E.; Neese, F. Efficient implementation of the analytic second derivatives of Hartree–Fock and hybrid DFT energies: a detailed analysis of different approximations. *Molecular Physics* **2015**, *113*, 1961–1977.

25 Kutzelnigg, W.; Liu, W. Quasirelativistic theory equivalent to fully relativistic theory. *The Journal of Chemical Physics* **2005**, *123*, 241102.

26 (a) Pollak, P.; Weigend, F. Segmented Contracted Error-Consistent Basis Sets of Double- and Triple-ζ Valence Quality for One- and Two-Component Relativistic All-Electron Calculations. *Journal of Chemical Theory and Computation* **2017**, *13*, 3696–3705. (b) Franzke, Y. J.; Treß, R.; Pazdera, T. M.; Weigend, F. Error-consistent segmented contracted all-electron relativistic basis sets of double- and triple-zeta quality for NMR shielding constants. *Physical Chemistry Chemical Physics* **2019**, *21*, 16658–16664.

27 (a) Heß, B. A.; Marian, C. M.; Wahlgren, U.; Gropen, O. A mean-field spin-orbit method applicable to correlated wavefunctions. *Chemical Physics Letters* **1996**, *251*, 365–371. (b) Neese, F. Efficient and accurate approximations to the molecular spin-orbit coupling operator and their use in molecular g-tensor calculations. *The Journal of Chemical Physics* **2005**, *122*, 034107.

28 (a) Maurer, L. A.; Pearce, O. M.; Maharaj, F. D. R.; Brown, N. L.; Amador, C. K.; Damrauer, N. H.; Marshak, M. P. Open for Bismuth: Main Group Metal-to-Ligand Charge Transfer. *Inorganic Chemistry* **2021**, *60*, 10137–10146. (b) Parke, S. M.; Narreto, M. A. B.; Hupf, E.; McDonald, R.; Ferguson, M. J.; Hegmann, F. A.; Rivard, E. Understanding the Origin of Phosphorescence in Bismoles: A Synthetic and Computational Study. *Inorganic Chemistry* **2018**, *57*, 7536–7549. (c) Imran, M.; Mix, A.; Neumann, B.; Stammler, H.-G.; Monkowius, U.; Bleckenwegner, P.; Mitzel, N. W. Synthesis, structural and photo-physical studies of bismuth(iii) complexes with Janus scorpionate and co-ligands. *Dalton Transactions* **2014**, *43*, 10956–10968. (d) Ohshita, J.; Matsui, S.; Yamamoto, R.; Mizumo, T.; Ooyama, Y.; Harima, Y.; Murafuji, T.; Tao, K.; Kuramochi, Y.; Kaikoh, T.; et al. Synthesis of Dithienobismoles as Novel Phosphorescence Materials. *Organometallics* **2010**, *29*, 3239–3241.

29 Adamo, C.; Barone, V. Toward reliable density functional methods without adjustable parameters: The PBE0 model. *J. Chem. Phys.* **1999**, *110*, 6158–6170.

30 Barone, V.; Cossi, M. Quantum Calculation of Molecular Energies and Energy Gradients in Solution by a Conductor Solvent Model. *J. Phys. Chem. A* **1998**, *102*, 11, 1995–2001.

31 Küchle, W.; Dolg, M.; Stoll, H.; Preuss, H. Ab initio pseudopotentials for Hg through Rn. *Mol. Phys.* **1991**, 74, 1245–1263.

32 Weigend, F. Accurate Coulomb-fitting basis sets for H to Rn. *Phys. Chem. Chem. Phys.* **2006**, *8*, 1057–1065.

33 Weigend, F. Hartree–Fock exchange fitting basis sets for H to Rn. *J. Comput. Chem.* **2008**, *29*, 167–175.

34 Pavlishchuk, V. V.; Addison, A. W. Conversion constants for redox potentials measured versus different reference electrodes in acetonitrile solutions at 25°C. *Inorganica Chimica Acta* **2000**, *298*, 97–102.

35 Hansch, C.; Leo, A.; Taft, R. W. A survey of Hammett substituent constants and resonance and field parameters. *Chem. Rev.* **1991**, *91*, 165–195.
